# Achieving High‐Performance Organic Long Persistent Luminescence Materials via Manipulation of Radical Cation Stability

**DOI:** 10.1002/advs.202416853

**Published:** 2025-02-22

**Authors:** Hongxin Gao, Guangming Wang, Tengyue Wang, Zi Ye, Qianqian Yan, Qianhui Chong, Chin‐Yiu Chan, Biaobing Wang, Kaka Zhang

**Affiliations:** ^1^ Jiangsu Key Laboratory of Environmentally Friendly Polymeric Materials School of Materials Science and Engineering Jiangsu Collaborative Innovation Center of Photovoltaic Science and Engineering Changzhou University 21 Gehuzhong Road Changzhou 213100 P. R. China; ^2^ State Key Laboratory of Organometallic Chemistry Key Laboratory of Synthetic and Self‐Assembly Chemistry for Organic Functional Molecules Shanghai Institute of Organic Chemistry Chinese Academy of Sciences University of Chinese Academy of Sciences 345 Lingling Road Shanghai 200032 P. R. China; ^3^ Department of Materials Science and Engineering City University of Hong Kong Tat Chee Avenue Kowloon Hong Kong SAR 999077 China

**Keywords:** difluoroboron β‐diketonate, organic afterglow, organic long persistent luminescence, radical cation, room‐temperature phosphorescence

## Abstract

Organic long persistent luminescence (OLPL) materials, with their hour‐long afterglow, hold great promise across numerous applications, yet their performance lags behind that of inorganic counterparts. A deeper understanding of the underlying photophysical mechanisms, particularly the effective control of radical intermediates, is essential for developing high‐performance OLPL materials; while systematic studies on the intrinsic stability of radical intermediates and their impact on OLPL performance remain scarce. Here biphenyl groups is introduced into a luminophore‐matrix‐donor three‐component OLPL system. By varying substituents at the ortho‐position of the biphenyl groups, the stability of radical cations is systematically modulated, and their influence on OLPL properties is investigated. Combined experimental results and theoretical calculations reveal that increased flexibility of the biphenyl bond and adjustable conformations lead to higher stability of radical cations, thereby significantly enhancing OLPL performance. Based on this understanding, a luminophore with two biphenyl groups is designed to successfully achieve remarkable afterglow brightness close to inorganic Sr_2_Al_14_O_25_/Eu^2+^, Dy^3+^ materials. Furthermore, these OLPL materials exhibit time‐encoded afterglow properties and promising applications in advanced anti‐counterfeiting, as well as background‐independent bioimaging functions. This work not only provides a novel strategy for constructing high‐performance OLPL materials but also lays a foundation for their widespread application in various fields.

## Graphical abstract

The stability of radical intermediates in the OLPL system is crucial for high‐performance materials but remains rarely explored. This study presents the first example of a systematic study of radical cation stability in the OLPL system. Results show that increased flexibility of the biphenyl bond leads to higher stability of radical cations, thereby enhancing OLPL. This work offers fundamental insights into molecular design principles for optimizing OLPL.

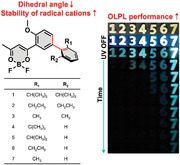

## 1 Introduction

Organic afterglow materials feature long emission durations and exhibit intriguing functions in oxygen sensing and analysis, biomedical optical imaging, anti‐counterfeiting, and optical data storage.^[^ ^1^, ^2^, ^3^, ^4^, ^5^, ^6^, ^7^, ^8^, ^9^, ^10^, ^11^, ^12^, ^13^, ^14^, ^15^, ^16^ ^]^ Such long‐lived organic emission can be divided into 1) room‐temperature phosphorescence (RTP),^[^ ^17^, ^18^, ^19^, ^20^, ^21^ ^]^ 2) TADF‐type organic afterglow (TADF represents thermally activated delayed fluorescence),^[^ ^22^, ^23^, ^24^, ^25^ ^]^ and 3) organic long persistent luminescence (OLPL).^[^ ^26^, ^27^, ^28^, ^29^, ^30^ ^]^ In recent years, OLPL materials have garnered significant attention due to their afterglow durations that can extend to hours or even longer, far exceeding the seconds or minutes‐long duration of RTP or TADF‐type afterglow; their afterglow follows power law decay with afterglow intensity being proportional to t^−m^ (m = 0–2), which is in sharp contrast to the exponential decay observed in RTP and TADF‐type afterglow systems.^[^ ^26^, ^27^, ^28^, ^29^, ^30^, ^31^, ^32^, ^33^, ^34^, ^35^, ^36^, ^37^, ^38^, ^39^, ^40^, ^41^, ^42^ ^]^ The hours‐long duration characteristic of OLPL materials, coupled with the advantages of organic materials such as ease of processing, cost‐effectiveness, and sustainability, highlights their potential for large‐scale applications.^[^ ^26^, ^27^, ^28^, ^29^, ^30^, ^31^, ^32^, ^33^, ^34^, ^35^, ^36^, ^37^, ^38^, ^39^, ^40^, ^41^, ^42^ ^]^ However, compared to inorganic long persistent luminescence (LPL) materials that have already found practical applications, organic counterparts generally exhibit lower afterglow performance metrics such as afterglow duration and afterglow brightness. The enhancement of performance metrics for OLPL materials hinges on a deeper understanding of the underlying photophysical mechanisms, for example, the charge separation and charge recombination processes, and the effective control of radical intermediates within the OLPL systems.

Early studies demonstrated that a two‐component system, comprising *N*,*N*,*N*′,*N*′‐tetramethylbenzidine (TMB) doped into polymethyl methacrylate (PMMA) matrix, could generate hours‐long OLPL via a two‐photon ionization mechanism at 20 K.^[^ ^43^ ^]^ In 2017, a two‐component material consisting of TMB donors doped into 2,8‐bis(diphenylphosphoryl)dibenzo[b,d] thiophene (PPT) acceptors was shown to emit OLPL afterglow in an inert atmosphere at room temperature; upon single‐photon excitation or normal excitation light, the material would under charge separation process to form TMB cation radicals and PPT anion radicals, followed by retarded charge recombination, generating an OLPL afterglow emission with a power‐law decay characteristic of up to several hours.^[^ ^26^ ^]^ A subsequent study in 2020 reported that TADF molecules incorporated in PPT, 1,3,5‐tris(1‐phenyl‐1H‐benzimidazol‐2‐yl)benzene (TPBi), or PMMA matrices could undergo two‐photon ionization (TPI) under high‐power irradiation, whereby the released electrons are captured by the surrounding matrix, forming charge‐separated states. The TADF molecules subsequently harvest singlet and triplet energies from charge recombination, thus exhibiting OLPL afterglow.^[^ ^28^ ^]^ Collectively, these studies, along with other reported OLPL systems,^[^ ^27^, ^29^, ^30^, ^31^, ^32^, ^33^, ^34^, ^35^, ^36^, ^37^, ^38^, ^39^, ^40^, ^41^, ^42^ ^]^ underscore the pivotal role of charge separation and recombination processes in OLPL mechanism, which are at the root of the prolonged afterglow.

The overall performance of OLPL materials is intricately linked to several critical factors. 1) The extent of charge separation is crucial. In TPI‐triggered OLPL systems, charge separation has been found to be facilitated by irradiation with high‐power excitation sources,^[^ ^28^, ^29^, ^33^, ^36^ ^]^ while in donor‐acceptor OLPL systems, the selection of components can ensure that the energy level of intermolecular charge transfer excited states is lower than the T_1_ levels of both the donor and acceptor, thereby achieving reasonable charge separation.^[^ ^26^, ^30^, ^31^, ^32^, ^35^, ^44^, ^45^, ^46^ ^]^ 2) The kinetics of charge recombination plays a vital role. The incorporation of electron traps in n‐type OLPL systems and hole traps in p‐type OLPL systems can modulate the kinetics of charge recombination, significantly extending the duration of OLPL afterglow.^[^ ^26^, ^30^ ^]^ Additionally, temperature and external fields have also been reported to modulate the charge recombination processes.^[^ ^33^, ^44^ ^]^ 3) The photoluminescence quantum yield (PLQY) of emitters is critical. The more effectively the emitter can harvest both S_1_ and T_1_ states generated during charge recombination, the higher the brightness and longer the duration of the OLPL afterglow will be. 4) The protection of radical intermediates is essential. In two or three‐component OLPL systems, there are often at least two or even three types of radical intermediates, all of which are prone to deactivation. If these radical intermediates deactivate significantly before charge recombination, the afterglow performance of the OLPL materials deteriorates markedly; conversely, if the stability of the radical intermediates is high or controllable, it becomes crucial for the development of high‐performance or responsive OLPL materials. It has been reported that inert atmospheres and encapsulation can prevent quenching of the reactive intermediates in OLPL materials by oxygen and moisture, while crystalline matrices have also been shown to protect intermediates well, enabling the realization of OLPL materials with long durations and even those dispersible in water.^[^ ^47^ ^]^ However, systematic studies on the intrinsic stability of radical intermediates and their impact on the performance of OLPL materials remain scarce.

In the research area of organic radicals, the stability of radical intermediates is one of the most critical topics, which can be categorized into thermodynamic stability and kinetic stability.^[^ ^48^, ^49^ ^]^ One aspect of thermodynamic stability pertains to molecular systems containing biphenyl groups.^[^ ^50^ ^]^ In a closed‐shell state, the dihedral angle between the two benzene rings in the biphenyl structure is relatively large; conversely, in an open‐shell state, the biphenyl radical exhibits a smaller dihedral angle, leading to a higher degree of molecular planarity and conjugation. As a result, biphenyl radical intermediates are comparatively stable. We envision that if the radical intermediates formed upon photoexcitation in OLPL systems exhibit enhanced stability, this would favorably impact the performance of OLPL materials, as it would mitigate the deactivation of these radical intermediates prior to charge recombination. Biphenyl is a common group found in many organic molecules, frequently appearing in the chemical structures of components used in OLPL materials. Therefore, studying the stability of radical intermediates in OLPL systems containing biphenyl groups, as well as their impact on OLPL afterglow properties, will deepen our understanding of OLPL systems.

**Scheme 1:**
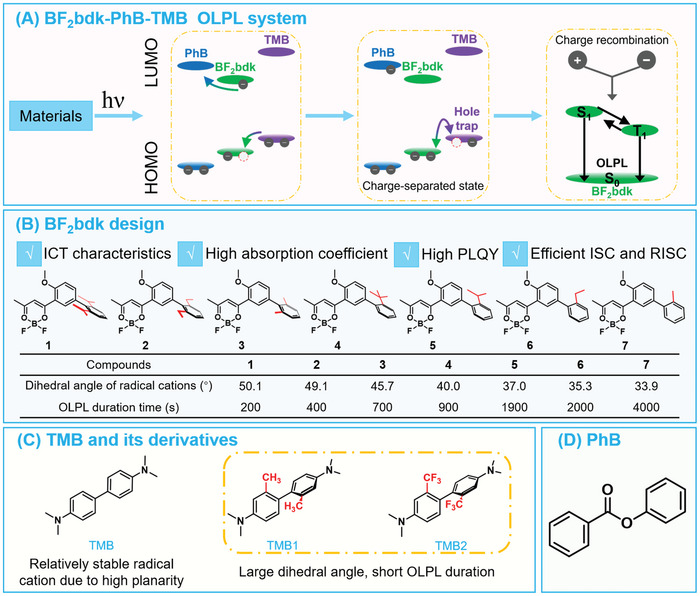
A) Photophysical processes in a **BF_2_bdk**‐PhB‐TMB three‐component OLPL model system developed in this study. B) Design of **BF_2_bdk** molecules with varied dihedral angles. C) TMB and TMB derivatives with large dihedral angles. D) Chemical structures of PhB matrix.

Given this perspective, here we develop a three‐component OLPL system based on **BF_2_bdk**‐PhB‐TMB materials, where **BF_2_bdk** represents difluoroboron β‐diketonate molecules containing biphenyl groups and PhB represents phenyl benzoate, which is further utilized as a model to investigate the impact of radical stability on OLPL afterglow properties (**Scheme** **1**A). A series of **BF_2_bdk** molecules (compounds **1**–**7**) with biphenyl groups have been designed and synthesized, whose conformation and dihedral angles between the benzene rings can be modulated through ortho‐substituents, thereby regulating the stability of the radicals (Scheme 1B). Upon losing an electron, TMB can form a relatively stable radical cation due to its increased planarity and conjugation, and TMB derivatives with the introduction of substituents at the ortho‐position of the TMB structure have also been designed to further control their radical cations stability (Scheme 1C). It has been found that in the **BF_2_bdk**‐PhB‐TMB system when the flexibility of the biphenyl bond increases and conformations can be freely adjusted, their OLPL performance can be improved significantly due to the higher stability of the radical cations, as supported by theoretical calculations. In the **7**‐PhB‐TMB derivative system, the presence of substituents on the TMB derivatives, whether electron‐donating or electron‐withdrawing, results in poorer OLPL performance; this is because the substituted TMB derivatives have restricted conformational flexibility, which hampers the planarity and stability of the radical cations. Building upon this work, **BF_2_bdk** molecules containing two biphenyl groups have been also designed, the obtained **10**‐PhB‐TMB materials exhibit excellent OLPL performance with afterglow duration exceeding 2.5 h, and their OLPL afterglow brightness is close to inorganic Sr_2_Al_14_O_25_/Eu^2+^, Dy^3+^ materials.

## 2 Results and Discussion

### 2.1 Material Design of BF_2_bdk‐PhB‐TMB Model System

**BF_2_bdk**‐PhB‐TMB three‐component OLPL system has been selected as a model (Scheme 1A). PhB has been employed as an organic matrix (Scheme 1D), because 1) its crystalline environment can protect long‐lived excited states and radical intermediates from oxygen quenching,^[^ ^22^, ^23^, ^51^, ^52^ ^]^ 2) its low conjugation degree and negligible absorption above 310 nm (Figure S2, Supporting Information) would not interfere with the light absorption of luminescent molecules, 3) it can interact with S_1_‐state **BF_2_bdk** via dipole‐dipole interaction to increase forward and reverse intersystem crossing (which can not only increase the population of long‐lived **BF_2_bdk** excited states but also facilitate **BF_2_bdk** to efficiently harvest the excitons generated by charge recombination),^[^ ^22^ ^]^ 4) its high T_1_ energy level of ≈3.5 eV (by TD‐B3LYP/6‐31g(d,p)) can suppress afterglow quenching caused by **BF_2_bdk** to PhB triplet excited state energy transfer, 5) its moderate melting point of 70 °C allows facile melt‐casting, 6) its ester group can accept electrons to form radical anions.^[^ ^36^, ^41^, ^43^, ^53^, ^54^ ^]^ **BF_2_bdk** molecules exhibit typical intramolecular charge transfer (ICT) characteristics with high molar absorption coefficients as well as high PLQYs and therefore are selected as luminescent components (Scheme 1B). Upon excitation, **BF_2_bdk** molecules in the PhB matrix can undergo intersystem crossing (ISC) and reverse intersystem crossing (RISC) processes to generate long‐lived T_1_ and S_1_ excited states.^[^ ^22^, ^55^, ^56^, ^57^ ^]^ There is a certain probability that an electron in the excited **BF_2_bdk**’s lowest unoccupied molecular orbital (LUMO) can be transferred to the PhB matrix's LUMO. Since the LUMO level of the PhB matrix is higher than that of **BF_2_bdk**, this electron transfer process is thermodynamically unfavorable, resulting in a low extent of occurrence. Fortunately, TMB can donate an electron from its highest occupied molecular orbital (HOMO) to the excited **BF_2_bdk**’s HOMO, compensating for the energy loss associated with the aforementioned thermodynamically unfavorable process (Scheme 1A). This promotes charge separation, leading to the formation of significant amounts of PhB radical anions, while **BF_2_bdk** radical cations and TMB radical cations establish an equilibrium; here TMB acts as hole traps, and charge‐separated states are consequently formed. The above charge separation process involves two electron transfer events: the electron transfer process from **BF_2_bdk**’s LUMO to the PhB matrix's LUMO, and the electron transfer process from TMB's HOMO to the **BF_2_bdk**’s HOMO; this OLPL mechanism differs from those observed in donor‐acceptor OLPL systems and TPI‐triggered OLPL systems.^[^ ^26^, ^27^, ^28^, ^29^, ^30^, ^31^, ^32^, ^33^, ^34^, ^35^, ^36^, ^37^, ^38^, ^39^, ^40^, ^41^, ^42^ ^]^ Subsequently, the retarded charge recombination occurs, resulting in the manifestation of OLPL in **BF_2_bdk**‐PhB‐TMB three‐component systems (Scheme 1A).

To investigate the influence of the stability of **BF_2_bdk** radical cations on OLPL performance, we designed a series of ICT‐type **BF_2_bdk** molecules containing biphenyl group (compounds **1**–**7** in Scheme 1B; Figures S3–S5 and Table S1, Supporting Information). Ortho‐substituents on the **BF_2_bdk** molecules allow for modulation of the conformation and dihedral angles, as supported by density functional theory (DFT) calculations and X‐ray single‐crystal structure analyses (Table S2 and Figures S6–S12, Supporting Information). By altering the substituents, the planarity of the biphenyl group in the radical cations can be modulated, thereby controlling the stability of the **BF_2_bdk** radical cations. Similarly, TMB derivatives with ortho‐substituents on their biphenyl group have been designed (Scheme 1C), and their molecular structures and high purities have been confirmed (Figure S3, Supporting Information). The TMB radical cations exhibit a high degree of planarity and conjugation, leading to greater stability; while ortho‐substituents on TMB derivatives can reduce the planarity of their radical cations. Thus, through the manipulation of ortho‐substituents, we can effectively regulate the stability of the radical cations in the TMB system.

### 2.2 Material Preparation and Characterization

**Figure 1:**
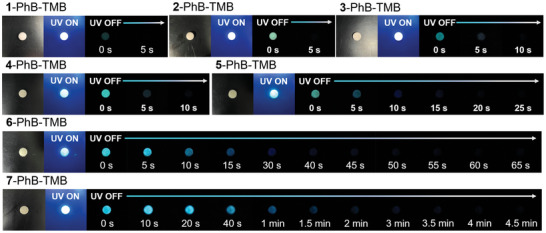
Photographs of **BF_2_bdk**‐PhB‐TMB‐0.2% samples under daylight lamp, 365 nm UV light, and after removal of the UV light.

**Figure 2:**
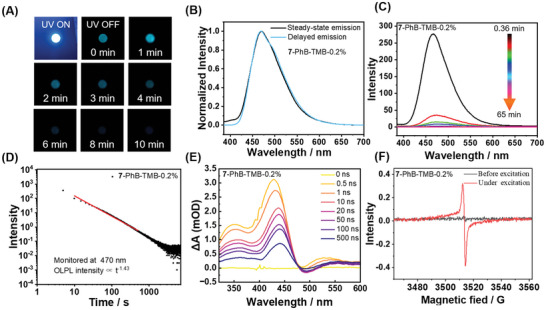
A) Photographs of **7**‐PhB‐TMB melt‐cast sample under 365 nm UV light and after removal of the UV light. B) Steady‐state and delayed emission (1 ms delay) spectra of **7**‐PhB‐TMB. C) Delayed emission spectra of the **7**‐PhB‐TMB excited at 365 nm at different delay times. D) Emission decay profiles (monitored at 470 nm) of **7**‐PhB‐TMB excited at 365 nm. E) Transient absorption spectra of **7**‐PhB‐TMB from 0 to 500 ns. F) ESR spectra of **7**‐PhB‐TMB materials.

After doping **BF_2_bdk** and TMB into PhB at a concentration of 0.2% by mass (detailed in Supporting Information), the resultant **BF_2_bdk**‐PhB‐TMB three‐component materials exhibit noticeable afterglow observable in a dark room under ambient conditions (**Figure** **1**); this observation starkly contrasts with the individual components—PhB, TMB, and **BF_2_bdk**—whose powders and dichloromethane solutions do not display any significant afterglow upon excitation at 365 or 405 nm (Figure S13, Supporting Information). Comprehensive photophysical characterizations have been performed to investigate the afterglow properties of these **BF_2_bdk**‐PhB‐TMB materials, with the data summarized in **Table** **1**. Among **1** to **7** systems, **7**‐PhB‐TMB materials demonstrate excellent afterglow performance, with blue afterglow remaining visibly detectable for 10 min after the removal of a 5 W 365 nm lamp (**Figure** **2**A); this remarkably prolonged afterglow is also detectable instrumentally. The delayed emission spectra at different delay times of **7**‐PhB‐TMB materials show that there is still a clear afterglow emission even 65 min after ceasing excitation (Figure 2C), and their afterglow intensity follows a power‐law decay trend, where afterglow intensity is proportional to t^−m^ with the m value of 1.43 (Figure 2D); such a power‐law decay is consistent with the decay characteristic of OLPL afterglows.^[^ ^26^, ^27^, ^28^, ^29^, ^30^, ^31^, ^32^, ^33^, ^34^, ^35^, ^36^, ^37^, ^38^, ^39^, ^40^, ^41^, ^42^ ^]^ Besides, the afterglow decay of **7**‐PhB‐TMB materials has also been collected to conform to the power‐law decay trend (Figure 2D), from which we can also estimate the afterglow duration to be ≈1.1 h (Table 1; Figure S14, Supporting Information).

**Table 1:** Photophysical property of **BF_2_bdk**‐PhB‐TMB‐0.2% afterglow materials.

BF_2_bdk	λ_F_ / nm	λ_DF_ / nm	λ_OLPL_ / nm	OLPL intensity^a)^	Duration^b)^ / s	*m* value
**1**	445	442	/	/	200	0.79
**2**	439	445	/	/	400	0.89
**3**	454	455	452	38	700	1.17
**4**	459	462	461	24	900	1.22
**5**	469	468	467	27	1900	1.19
**6**	470	464	470	50	2000	1.32
**7**	470	469	470	277	4000	1.43
**8**	473	474	486	484	4000	0.79
**9**	492	493	498	940	≥9000	1.34
**10**	493	495	501	386	≥9000	1.06

^a)^ obtained from OLPL spectra at ≈0.4 min;

^b)^ estimated from OLPL emission decay profiles.

Previous literature has suggested that the generation of OLPL afterglow might be due to the formation of exciplexes between donor and acceptor components after photoexcitation and excited state energy transfer.^[^ ^26^, ^27^, ^30^, ^31^, ^32^, ^35^, ^37^, ^44^, ^45^, ^46^ ^]^ However, in this work, neither **BF_2_bdk**‐PhB nor TMB‐PhB two‐component materials exhibit observable OLPL afterglow at room temperature; their afterglow lasts no more than 4 s under ambient conditions (Figures S15,S16 and S17A, Supporting Information). The OLPL energy transfer from TMB‐PhB to **BF_2_bdk** and other mechanisms can be ruled out (detailed in Text S1 and Figures S22–S24, Supporting Information).

To study TMB's role, we replace 0.2% TMB with 0.2% **BF_2_bdk** to prepare **BF_2_bdk**‐PhB‐0.4% samples (using compounds **1** to **7**) and perform photophysical measurements (Figures S18 and S19, Supporting Information). **BF_2_bdk**‐PhB‐0.4% samples have relatively bright afterglow and relatively long afterglow durations than **BF_2_bdk**‐PhB‐0.2% samples (Figures S15 and S18, Supporting Information), which can be attributed to the increased doping concentration of **BF_2_bdk**. However, **BF_2_bdk**‐PhB‐0.4% samples don't show OLPL or have very weak OLPL (Figure S20, Supporting Information). For example, the **7**‐PhB‐0.4% sample only has an OLPL intensity of 14 (estimated from OLPL spectra at ≈0.4 min), while the **7**‐PhB‐TMB‐0.2% sample shows an OLPL intensity of 277 (Table 1 and Figure 2c). These results indicate the critical role of TMB donors in the emergence of significant OLPL in **BF_2_bdk** systems.

To get more photophysical behavior of the **BF_2_bdk**‐PhB‐TMB systems, the excitation dependent OLPL measurements have been performed (Figure S21, Supporting Information). It was found that the intensity and duration of OLPL increased with the excitation power (Figure S21A,B, Supporting Information). Interestingly, OLPL signals can still be observed even at 2 µW/cm^2^ excitation power, indicating that the OLPL in the **BF_2_bdk**‐PhB‐TMB systems is not mainly caused by a two‐photon ionization mechanism. Besides, by fixing excitation power at 2.1 mW cm^−2^, it has been found that the OLPL intensity and duration slightly increase with excitation time (Figure S21C,D, Supporting Information). Significant OLPL afterglow can be achieved even after excitation as short as 5 s, which indicates the fast response of the present three‐component OLPL materials.

To further explore the OLPL mechanism in **BF_2_bdk**‐PhB‐TMB materials, transient absorption (TA) spectra have been collected to investigate the involvement of various radical intermediates, including radical cations and radical anions, in the generation process of OLPL afterglow. **7**‐PhB‐TMB materials show transient absorption bands at 300–400 nm, 400–480 nm, and 500–600 nm (Figure 2E). The 300–400 nm transient absorption band can be assigned to PhB radical anions because 1) the radical anion of ethyl benzoate has an absorption band at 310 nm^[^ ^54^ ^]^ and 2) the PhB radical anion has increased conjugation length compared to ethyl benzoate radical anion, leading to a red shift of its absorption band. In the reported study by Adachi,^[^ ^26^ ^]^ the transient absorption spectrum of the TMB/PPT sample after photo‐excitation exhibited a broad absorption band between 600 and 1400 nm, as well as an absorption band in the range of 400 to 500 nm. Besides, in other reported studies,^[^ ^58^, ^59^, ^60^ ^]^ the absorption band of TMB radical cation has been found to be located in the range of 400 to 500 nm. Therefore, the transient absorption band at 400–480 nm in the present study can be assigned to TMB radical cations (Figure 2E). The 500–600 nm band can be attributed to **7′**s radical cations or excitons, as supported by our previous study on **BF_2_bdk** with similar structures.^[^ ^36^, ^41^ ^]^ The presence of radical intermediates (g = 2.003) can be also confirmed by electron spin resonance (ESR) spectra (Figure 2F). Moreover, **7**‐PhB‐TMB materials can be excited by 405 nm light to produce OLPL afterglow (Figure S25, Supporting Information), whereas PhB‐TMB exhibits weak or no absorption at 405 nm (Figure S17, Supporting Information), suggesting that the absorption and subsequent excitation of **BF_2_bdk** drives the photophysical process leading to OLPL afterglow. We propose that the charge separation occurring in **7**‐PhB‐TMB materials proceeds through the following steps (Scheme 1A): 1) Upon photoexcitation, **BF_2_bdk** molecules are excited, accompanied by the electron transition from their HOMO to the LUMO (Table S3, Supporting Information); (2) PhB matrix (LUMO, −1.8–2.1 eV) captures an electron from the excited **BF_2_bdk**’s LUMO (−2.6–2.2 eV), while TMB serves as the hole trap, facilitating the electron transfer process from its HOMO (−4.8 eV) to **BF_2_bdk**’s HOMO (−6.4–6.0 eV) (Text S2, Supporting Information), thereby driving charge separation and the formation of PhB radical anions, **BF_2_bdk** radical cations, and TMB radical cations. It has been observed that the emission maximum in the OLPL spectrum (0.36 min delay) of **7**‐PhB‐TMB materials at 470 nm aligns closely with the emission peaks in their steady‐state and delayed emission spectra (Figure S27, Supporting Information), which also corresponds to the TADF emission peak of **7**‐PhB materials (vide infra), suggesting that the OLPL emission of **7**‐PhB‐TMB materials originates from **7′**s S_1_ emission. Therefore, we propose that the radical cations and radical anions formed by charge separation eventually undergo retarded charge recombination on the **BF_2_bdk** molecules (Scheme 1A), generating long‐lasting OLPL afterglow; because of their TADF character, **BF_2_bdk** can both harvest singlet and triplet excitons formed from charge recombination, ultimately returning to the ground state and releasing prolonged OLPL.

To further investigate the excited state properties of **BF_2_bdk**, **BF_2_bdk**‐PhB two‐component materials have also been prepared. The **BF_2_bdk**‐PhB‐0.2% materials exhibit bright room‐temperature afterglow under ambient conditions (Figures S15 and S16, Supporting Information), and their photophysical properties have been comprehensively characterized and summarized in Table S4 and Figure S28 (Supporting Information). For instance, **7**‐PhB materials display a bright sky‐blue afterglow upon the removal of a 365 nm lamp (Figure S29A, Supporting Information), with their delayed emission spectrum nearly overlapping with steady‐state emission spectrum, featuring an emission peak at 469 nm, afterglow lifetime of 228 ms, and PLQY of 51.7% (Figures S29B and S16, Supporting Information). This nearly overlapping spectral behavior is similar to previously reported **BF_2_bdk**‐PhB systems, which have been confirmed to be attributed to TADF‐type afterglow originating from the S_1_ emission of **BF_2_bdk** (detailed in Text S3, Supporting Information).^[^ ^22^, ^57^ ^]^ To support the TADF emission has a few hundred milliseconds lifetime, we collect emission decay curves of **BF_2_bdk**‐PhB‐0.2% samples at different wavelengths (Figure S30, Supporting Information). It has been found that the emission lifetimes monitored at higher‐energy regions are slightly shorter than or similar to those recorded at lower‐energy regions (Figure S30, Supporting Information). In particular, the emission decay curves monitored at 430 nm, where phosphorescence is absent as can be seen from the 77 K delayed emission spectra, show a long emission lifetime of a few hundred milliseconds (Figure S32, Supporting Information), which indicates that the TADF has lifetime > 100 ms in the present study. We understand that most of the reported TADF emitters have delayed fluorescence lifetimes of 10^−3^–10^−6^ s. Here the assignment of TADF‐type organic afterglow is supported by a series of experimental observations and analyses. 1) The similar emission color of **BF_2_bdk**‐PhB sample at room temperature under UV excitation (fluorescence color) and after ceasing UV light (afterglow color), as well as the almost identical steady‐state and delayed emission spectra of **BF_2_bdk**‐PhB sample at room temperature (for example, in the case of **7**‐PhB). 2) The absence of triplet‐to‐singlet excited state energy transfer, the absence of donor‐acceptor afterglow mechanism based on intermolecular charge transfer and retarded charge recombination, and the absence of afterglow mechanism caused by impurity (detailed in Text S3, Supporting Information). 3) The disappearance of the delayed fluorescence band at 77 K and the emergence and increase of the delayed fluorescence band upon temperature increase. 4) The insignificant contribution of triplet‐triplet annihilation at low doping concentration.

### 2.3 Relationship Between Radical Cation Stability and OLPL Performance

**Figure 3:**
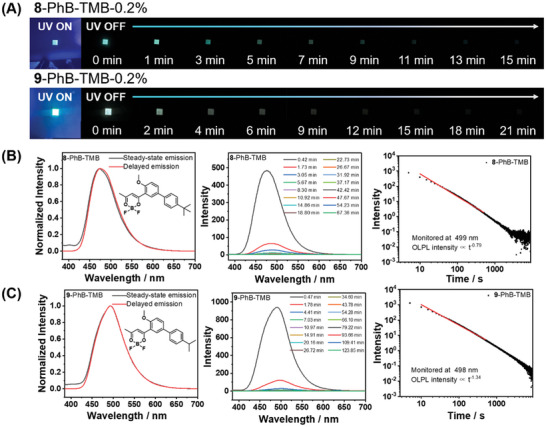
A) Photographs of **8, 9**‐PhB‐TMB‐0.2% afterglow materials under a 365 nm UV light and after the removal of the UV light. B, C) Room temperature steady‐state emission spectra, delayed emission (1 ms delay) spectra, OLPL emission spectra at different delay times, and emission decay profiles of **8**‐PhB‐TMB‐0.2% (B) and **9**‐PhB‐TMB‐0.2% (C).

Based on the above understanding of the OLPL mechanism, the variations in OLPL afterglow performance among the **BF_2_bdk**‐PhB‐TMB materials have been further investigated. To facilitate the comparison of the afterglow performance of these OLPL materials, afterglow performance metrics such as the OLPL afterglow duration time and initial OLPL afterglow brightness are summarized in Table 1; afterglow duration time is estimated based on the time point where the afterglow lifetime decay curve flattens, while the initial OLPL afterglow intensity is obtained from the delayed emission spectra with a delay time of ≈0.4 min. The comparative results indicate that OLPL afterglow performance follows the order: 1 < 2 < 3 < 4 < 5 < 6 < 7 (Table 1). To elucidate the observed trend in OLPL afterglow performance, we further studied the stability of the **BF_2_bdk** radical cations via theoretical calculations. TD‐DFT calculations reveal that the dihedral angle of the biphenyl groups of the optimized **BF_2_bdk** radical cations is in the following order: 1 > 2 > 3 > 4 > 5 > 6 > 7, which is in accordance with the OLPL afterglow performance (Scheme 1B); since smaller dihedral angles between biphenyl groups lead to better planarity of the biphenyl radical cations, thereby enhancing the stability of the **BF_2_bdk** radical cations as well as the OLPL afterglow performance. The relationship between the stability of the **BF_2_bdk** radical cations and the dihedral angle can also be assessed through the electronic energy or free energy of the radical cations. Accordingly, we used the optimized dihedral angle of each **BF_2_bdk** radical cation and removed the alkyl substituents on biphenyl groups for ease of calculation and comparison of the electronic energy or free energy of the radical cations. The results are summarized in Table S5 (Supporting Information), indicating that as the dihedral angle increases, both the electronic energy and free energy of **BF_2_bdk** radical cations significantly increase, which is detrimental to its thermodynamic stability. Therefore, a reduction in the number of substituents and steric hindrance favors increased torsional freedom around the biphenyl single bond, enhancing the stability of the **BF_2_bdk** radical cations. This stabilization is responsible for the favorable OLPL afterglow performance in a **7**‐PhB‐TMB system with higher torsional freedom. One may argue that the alkyl substituents could influence the molecular packing in the PhB matrix, potentially affecting some photophysical processes and leading to changes in OLPL properties. To address this, the bulky groups in compounds **4** and **5** have been relocated to the para‐position of the biphenyl group to obtain compounds **8** and **9**, which serve as controls for further investigation. Significantly, the resultant **8**‐PhB‐TMB and **9**‐PhB‐TMB materials exhibit notably better OLPL afterglow performance (from both aspects of afterglow durations and OLPL intensity at ≈0.4 min delayed time) compared to **4**‐PhB‐TMB materials and **5**‐PhB‐TMB materials, as well as **7**‐PhB‐TMB materials (**Figure** **3** and Table 1); this indicates that the inferior OLPL afterglow performance in **4**‐PhB‐TMB and **5**‐PhB‐TMB materials is primarily due to the ortho‐substituents affecting the stability of **BF_2_bdk** radical cations, rather than caused by the change of molecular packing of **BF_2_bdk** within the materials.

Interestingly, the OLPL afterglow of **BF_2_bdk**‐PhB‐TMB materials also exhibits temperature‐dependent characteristics (Figure S34, Supporting Information). In the case of **7**‐PhB‐TMB materials, the OLPL brightness and durations at 77 K are weaker and shorter than those at room temperature (Figure S34A,C,D, Supporting Information). This is understandable because the rate of charge recombination usually increases with temperature in conventional OLPL systems.^[^ ^33^, ^39^, ^44^ ^]^ Actually, we also find a significant enhancement of OLPL intensity of the **7**‐PhB‐TMB system during transferring the materials from 77 K to room temperature (Figure S35, Supporting Information), indicating the enhanced charge recombination at elevated temperature. Notably, unlike the sky‐blue OLPL exhibited at room temperature, **7**‐PhB‐TMB materials show a green OLPL at 77 K (Figure S34A, Supporting Information). Their OLPL spectra at different delay times at 77 K (Figure S34C, Supporting Information) indicate that **7**‐PhB‐TMB materials exhibit a maximum emission peak at 505 nm and a minor emission peak at 465 nm, which can be attributed to **7′**s T_1_ and S_1_ emission, respectively (vide supra). After charge recombination, 25% of excitons exist in the form of S_1_ states and 75% in T_1_ states, which can interconvert through ISC/RISC processes at room temperature. At 77 K, thermally activated RISC is suppressed while ISC remains active, so the OLPL of **7**‐PhB‐TMB materials primarily manifests as T_1_ emission at 77 K.

The temperature‐sensitive emission behavior of the **1**‐PhB‐TMB system is very different from that of the **7**‐PhB‐TMB system. As mentioned above, the **1**‐PhB‐TMB sample has very weak OLPL at room temperature. By placing a **1**‐PhB‐TMB sample at 77 K, one can find the emergence of significant OLPL as revealed by its 77 K emission spectra at different delay times (Figure S34B,E,F, Supporting Information). Such behavior is unusual for conventional OLPL materials but it precisely proves the essential role of radical cation stability for high‐performance OLPL materials; at low temperatures, the radical cation of compound **1** would be relatively stable (compared to that at room temperature), accounting for its significant OLPL at 77 K. In this specific case, the suppressed charge recombination at low temperatures is no longer the main factor in determining OLPL intensity.

It's interesting thing that the effects of substituents on the stability of radical cations and OLPL afterglow performance are not limited to the luminescent **BF_2_bdk**, but also to the electron donor TMB, as the TMB molecular structure contains biphenyl groups as well. Two different TMB derivatives, TMB1 and TMB2, have also been designed and synthesized by introducing CH_3_ or CF_3_ substituents onto the ortho‐position of TMB's biphenyl groups. Upon doping into the PhB matrix, the resultant **7**‐PhB‐TMB1 and **7**‐PhB‐TMB2 materials can also exhibit OLPL afterglow under ambient conditions (Figure S36, Supporting Information), but their OLPL afterglow intensity and duration time are far inferior to those of the **7**‐PhB‐TMB materials (Figure S36C,D, Supporting Information), which aligns with theoretical calculations. TD‐DFT calculations reveal that the dihedral angle of the biphenyl groups in the TMB derivatives is notably larger than that in TMB, and their electronic energy and free energy are also higher (Table S5, Supporting Information). These indicate that the substituents on the TMB derivatives hinder the planarity of the biphenyl groups, regardless of whether they are electron‐withdrawing or electron‐donating, leading to poor stability of the TMB derivative radical cations and, consequently, suboptimal OLPL afterglow performance of the resultant three‐component materials. In contrast, TMB radical cations have relatively high stability, which accounts for the favorable OLPL afterglow performance observed in the **BF_2_bdk**‐PhB‐TMB materials.

**Figure 4:**
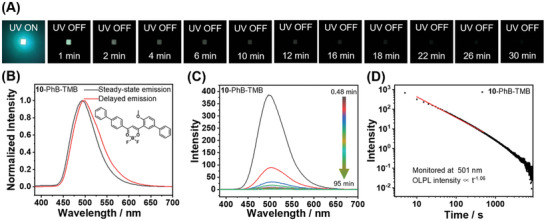
A) Photographs of **10**‐PhB‐TMB melt‐cast sample under 365 nm UV light and after removal of the UV light. B–D) Steady‐state and delayed emission (1 ms delay) spectra (B), OLPL spectra excited at 365 nm at different delay times (C), and emission decay profiles (monitored at 501 nm) of **10**‐PhB‐TMB excited at 365 nm (D).

The above experimental results and analyses highlight the importance of radical cation stability for OLPL afterglow properties. Additionally, it is noteworthy that both **BF_2_bdk** and TMB in this work possess biphenyl groups, while biphenyl groups with rotational freedom have been demonstrated to be beneficial for enhancing OLPL afterglow performance. To construct high‐performance OLPL materials, we designed and synthesized a novel **BF_2_bdk** molecule featuring two biphenyl groups, identified as compound **10**. Similar to other **BF_2_bdk** molecules, **10** also exhibits excellent photophysical properties. The UV‐vis spectrum of **10** in dichloromethane solution shows strong absorption peaks at 373 and 424 nm, with molar absorption coefficients of 3.66 and 3.22 × 10^4^ M^−1^ cm^−1^, respectively, while its steady‐state emission spectrum displays a maximum emission peak at 542 nm (Figure S37, Supporting Information). Upon doping into the PhB matrix, the resultant **10**‐PhB two‐component materials exhibit bright cyan fluorescence with a PLQY of 95.7%, along with a cyan TADF‐type afterglow that lasts for ≈4 s under ambient conditions (Text S4 and Figure S38, Supporting Information). Furthermore, TMB was incorporated into **10**‐PhB materials to promote the formation of radical intermediates and to extend the afterglow duration (**Figure** **4**). Significantly, the resultant **10**‐PhB‐TMB materials exhibit a remarkable cyan‐green OLPL afterglow upon removing excitation light, observable to the naked eye for ≈30 min (Figure 4A). The afterglow emission decay curves of **10**‐PhB‐TMB materials indicate that their OLPL could still be detected instrumentally even 9000 s after the removal of excitation (Figure 4D and Table 1), demonstrating an exceptionally prolonged OLPL duration. The OLPL emission spectrum (0.48 min delay) of **10**‐PhB‐TMB materials shows an emission peak at 501 nm (Figure 4C), which is similar to their delayed emission spectrum as well as the steady‐state emission spectrum (Figure 4B); this emission peak is almost identical to the TADF‐type afterglow emission at 504 nm of **10**‐PhB materials (Figure S38, Supporting Information), and can be attributed to **10′**s S_1_ state emission. It is noteworthy that the afterglow emission decay curve of **10**‐PhB‐TMB materials exhibits two distinct decay modes: one within the first 2 s after the cessation of excitation and the other after 2 s (Figure S40, Supporting Information). The first part of the decay curve (within the first 2 s post‐excitation) follows the exponential decay pattern, similar to the afterglow emission decay curve of **10**‐PhB materials, and can be attributed to TADF‐type afterglow; while the second part of the decay curve (after 2 s) follows a power‐law decay relationship, characteristic of the OLPL afterglow. The PLQY of **10**‐PhB‐TMB material is 50.7%, as measured using an integrating sphere system. This absolute PLQY allows us to estimate the efficiency of the first part (0–2 s) of conventional fluorescence plus TADF‐type afterglow to be 50.7%. However, due to the extended duration of the OLPL afterglow, which can last for several minutes to hours, current PLQY measurement instruments are unable to detect this component. Given the limited discussion of OLPL efficiency in reported studies on OLPL materials, we propose a method to estimate OLPL efficiency based on the specific properties of **BF_2_bdk**‐PhB‐TMB materials in this work (Text S5, Supporting Information). Using the proposed method, the OLPL efficiency can be estimated based on the proportion of the first‐part emission (82.2%) and the second‐part OLPL afterglow (17.8%) (Figure S40, Supporting Information). By applying the afterglow efficiency value of the first‐part emission (50.7%), we estimate the OLPL efficiency of the **10**‐PhB‐TMB‐0.2% materials to be 11.0%. Besides, the **10**‐PhB‐TMB‐0.2% materials have been found to show OLPL signals at 2 µW cm^−2^ UV excitation (Figure S21B, Supporting Information) and can be fast activated by 365 nm UV light at 2.1 mW cm^−2^ (Figure S21D, Supporting Information). Moreover, **10**‐PhB‐TMB materials exhibit excellent visible‐light‐excitable OLPL properties under ambient conditions, with OLPL afterglow properties at 420 nm excitation that are similar to those at 365 nm, including afterglow color and afterglow duration (Figure S41, Supporting Information). Notably, the OLPL afterglow brightness and duration of **10**‐PhB‐TMB materials are comparable to inorganic Sr_2_Al_14_O_25_/Eu^2+^, Dy^3+^ materials (Figure S42, Supporting Information), which further emphasizes the importance of the stability of radical cations for fabricating high‐performance OLPL materials.

### 2.4 Material Function

**Figure 5:**
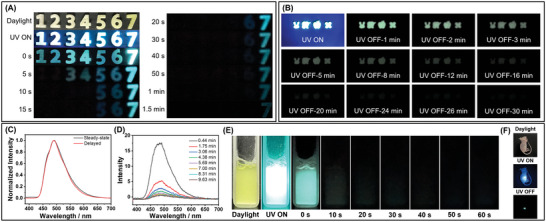
A) The afterglow photographs of **BF_2_bdk**‐PhB‐TMB materials, where each number corresponds to the name of the compound. B) Materials with various shapes of **10**‐PhB‐TMB exhibiting persistent afterglow at room temperature. C) Steady‐state emission spectra and delayed emission (1 ms delay) spectra, D) OLPL emission spectra, and E) the afterglow photographs of an aqueous afterglow suspension prepared from **10**‐PhB‐TMB under 365 nm excitation. F) Photographs of mice injected with **10**‐PhB‐TMB suspension under daylight, 365 nm UV lamp, and removal of 365 nm UV lamp.

Organic afterglow materials have been reported for promising applications in the fields of anti‐counterfeiting and information encryption, as the development of materials with higher levels of information security is an important research topic.^[^ ^61^, ^62^ ^]^ Given that compounds **1**–**7** systems exhibit distinctly different OLPL afterglow durations at room temperature, we combined them to construct devices that display varying numbers of afterglow objects at different delay times, which represents a novel time‐encoded anti‐counterfeiting material (**Figure** **5**). Due to the moderate melting point of the PhB matrix, these **BF_2_bdk**‐PhB‐TMB materials exhibit excellent processability. They can be melted into a moldable liquid upon heating and subsequently cooled in variously shaped silicone molds to obtain organic afterglow materials with different shapes of numbers, where each number corresponds to the compound's designation (Figure 5A). The combined afterglow pattern displays “1234567” in yellow under daylight, in blue under 365 nm light, and in blue afterglow immediately after the excitation source is removed (Figure 5A). At a delay time of 5 s, it shows “34567”; at 10 s, “567”; at 20 s, “67”; and after 40 s, only “7” remains (Figure 5A). The differential afterglow durations of these number‐shaped materials result in varying afterglow patterns and information over different time scales, demonstrating their potential for time‐encoded anti‐counterfeiting applications. Besides, other **BF_2_bdk**‐PhB‐TMB materials also exhibit excellent processability. For instance, **10**‐PhB‐TMB materials can be processed into various afterglow shapes and still show long‐lasting cyan afterglow at room temperature, with a duration of ≈30 min (Figure 5B). Additionally, **10**‐PhB‐TMB materials can also be prepared as the aqueous afterglow suspension using a melt‐droplet method with the aid of surfactant F127. The steady‐state and delayed emission spectra of **10**‐PhB‐TMB afterglow suspension are similar to those of the solid **10**‐PhB‐TMB materials (Figure 5C), indicating that the afterglow mechanism is not affected by the suspension form. Remarkably, **10**‐PhB‐TMB suspension also exhibits significant OLPL afterglow under ambient conditions (Figure 5D,E), with the OLPL afterglow signal remaining detectable in the OLPL emission spectrum even 10 min after the excitation source is removed (Figure 5E). Furthermore, after further injection into the tissue of mice, **10**‐PhB‐TMB suspensions can still show bright green afterglow with a clean background (Figure 5F), suggesting their potential for high‐contrast bioimaging applications.

## 3 Conclusion

In this study, we systematically investigated the effect of the stability of radical cations on room‐temperature OLPL property, focusing on the **BF_2_bdk**‐PhB‐TMB three‐component systems. The comprehensive photophysical characterizations reveal that the OLPL afterglow in these materials arises from the synergistic interaction between **BF_2_bdk**, PhB, and TMB, rather than from exciplex formation or energy transfer processes. Specifically, the **BF_2_bdk**‐PhB‐TMB materials exhibit remarkable OLPL afterglow performance, with **7**‐PhB‐TMB materials displaying a bright sky‐blue afterglow observable for 35 min post‐excitation. Further analysis of the radical cation stability in **BF_2_bdk** and TMB derivatives highlights the pivotal role of the rotational freedom of the biphenyl group in enhancing the stability of radical cations, which is critical for achieving high‐performance OLPL materials. The stability of radical cations is related to the charge recombination processes; the quenching of the radical cations due to their instability can be considered as nonradiative recombination in the system. With the introduction of two sterically unhindered biphenyl groups, the obtained **10**‐PhB‐TMB materials exhibit excellent OLPL performance with afterglow duration exceeding 2.5 h, and their OLPL afterglow even close to that of inorganic LPL materials, underscoring the importance of molecular design in optimizing these properties. During the fast development of OLPL systems in the past years,^[^ ^26^, ^27^, ^28^, ^29^, ^30^, ^31^, ^32^, ^33^, ^34^, ^35^, ^36^, ^37^, ^38^, ^39^, ^40^, ^41^, ^42^ ^]^ one may find that the involvement of biphenyl groups, aromatic amine groups, and their derivatives would be helpful for the enhancement of OLPL performance; this can be explained by the stability of the intermediate radical cations. Overall, this work provides fundamental insights into the molecular design principles governing the OLPL afterglow and paves the way for the development of high‐performance organic afterglow materials with tailored properties for diverse technological applications. Regarding the important topic of how to enhance OLPL performance, strong light absorption, efficient charge separation, high stability of radical intermediates, and improved harvesting of excitons generated by charge recombination are all very important for achieving such high‐performance OLPL systems.

CCDC 2391572, 2391573, 2391574, 2391575, 2391576, 2391577, and 2391578 contains the supplementary crystallographic data for this paper. These data can be obtained free of charge from The Cambridge Crystallographic Data Centre via www.ccdc.cam.ac.uk/data_request/cif.

## Conflict of Interest

The authors declare no conflict of interest.

## Supporting information



Supporting Information

## Data Availability

The data that support the findings of this study are available from the corresponding author upon reasonable request.
