# Supplementary material for: Achieving High‐Performance Organic Long Persistent Luminescence Materials via Manipulation of Radical Cation Stability
Source: Adv Sci (Weinh). 2025 Feb 22;12(15):2416853. doi: 10.1002/advs.202416853 (PMC12005741; doi:10.1002/advs.202416853)
Supplement: Supplementary file 1 — Supporting Information [file ADVS-12-2416853-s001.pdf]

ADVANCED SCIENCE
Open Access

Supporting Information

for *Adv. Sci.*, DOI 10.1002/advs.202416853

Achieving High-Performance Organic Long Persistent Luminescence Materials via Manipulation of Radical Cation Stability

Hongxin Gao, Guangming Wang, Tengyue Wang, Zi Ye, Qianqian Yan, Qianhui Chong, Chin-Yiu Chan, Biaobing Wang* and Kaka Zhang**

Supporting Information

Achieving High-Performance Organic Long Persistent Luminescence Materials via Manipulation of Radical Cation Stability

Hongxin Gao,[1,2] Guangming Wang,[2] Tengyue Wang,[2] Zi Ye,[2] Qianqian Yan,[2] Qianhui Chong,[1] Chin-Yiu Chan,[3,*] Biaobing Wang,[1,*] and Kaka Zhang[2,*]

[1]Jiangsu Key Laboratory of Environmentally Friendly Polymeric Materials School of Materials Science and Engineering, Jiangsu Collaborative Innovation Center of Photovoltaic Science and Engineering, Changzhou University, Changzhou, China.

*E-mail: biaobing@cczu.edu.cn

[2]State Key Laboratory of Organometallic Chemistry and Shanghai Hongkong Joint Laboratory in Chemical Synthesis, Key Laboratory of Synthetic and Self-Assembly Chemistry for Organic Functional Molecules, Ningbo Zhongke Creation Center of New Materials, Shanghai Institute of Organic Chemistry, University of Chinese Academy of Sciences, Chinese Academy of Sciences, 345 Lingling Road, Shanghai 200032, People's Republic of China.

*E-mail: zhangkaka@sioc.ac.cn;

[3]Department of Materials Science and Engineering, Department of Chemistry, City University of Hong Kong, Tat Chee Avenue, Kowloon, Hong Kong SAR, China.

*E-mail: chinychan2@cityu.edu.hk.

Table of Contents

Materials

Synthesis of compounds **1-10**, **TMB1** and **TMB2**

Physical measurements and instrumentation

TD-DFT calculations

Text S1. Ruling out TMB-PhB exciplex OLPL to **BF_2bdk** energy transfer mechanism and other mechanism

Text S2. About the electron transfer process from TMB's HOMO to **BF_2bdk**'s HOMO.

Text S3. TADF-type afterglow in **BF_2bdk**-PhB two-component system.

Text S4. About the TADF-type afterglow of **10**-PhB materials.

Text S5. About the methods for calculating or estimating OLPL efficiency.

Text S6. The selection guideline of organic matrix.

Figure S1. Examples of afterglow materials with OLPL property.

Figure S2. UV-vis absorption spectrum of PhB matrix and TMB.

Figure S3. HPLC profile of compounds **1-10**, **TMB1**, **TMB2.**

Figure S4. UV-vis spectra of compounds **1-7** in DCM and their steady-state emission spectra.

Figure S5. The fluorescence decay profiles of compounds **1-7** in dichloromethane solution excited at 365 nm.

Table S1. Photophysical data of compounds **1-7** in DCM at room temperature.

Table S2. Biphenyl dihedral angles of the ground states of compounds **1-7** obtained through TD-DFT calculations and single crystal analysis.

Figure S6-S12. Iso-surface maps of electron-hole density difference of compounds **1-7**'s excited states calculated at B3LYP/def2-TZVP(-f) level, where blue and green iso-surfaces correspond to hole and electron distributions, and excitation energies, oscillator strengths and spin–orbit coupling matrix element (SOCME) values.

Figure S13. Photos of compounds **1-7**, TMB, and PhB in solid state and dichloromethane solution under 365 nm light source or 405 nm light and after removal of light.

Figure S14. Steady-state and delayed emission spectra (1 ms delay), OLPL emission spectra (at different delay time) and OLPL emission decay profiles of **BF$_2$bdk**-PhB-TMB-0.2% afterglow materials excited at 365 nm.

Figure S15. Photographs of **BF$_2$bdk**-PhB-0.2% afterglow materials under 365 nm UV excitation and after the removal of the UV excitation.

Figure S16. Emission decay of **BF$_2$bdk**-PhB-0.2% samples excited at 365 nm and monitored at different wavelengths.

Figure S17. (A) Room-temperature delayed emission spectra (0.36 min delay) of **7**-PhB-TMB-0.2% (excited at 365 nm) and TMB-PhB-0.2% samples (excited at 320 nm). (B) UV-vis absorption spectra of TMB-COP-0.2% and TMB-PhB-0.2% samples, where COP represents cyclo olefin polymer. (C) Room-temperature steady-state emission spectra of TMB-COP-0.2% and TMB-PhB-0.2% samples. (D, E, F) Steady-state and delayed emission spectra (1 ms delay) and emission decay profile of TMB-COP-0.2% at 77 K excited at 320 nm. (G, H, I) Steady-state and delayed emission spectra (1 ms delay) and emission decay profile of TMB-PhB-0.2% at 77 K excited at 320 nm.

Figure S18. Photographs of **BF$_2$bdk**-PhB-0.4% afterglow materials under 365 nm UV excitation and after the removal of the UV excitation.

Figure S19. (A) Room temperature steady-state emission and delayed emission (1 ms delay) spectra of **BF$_2$bdk**-PhB-0.4% materials under ambient conditions. (B) Emission decay of **BF$_2$bdk**-PhB-0.4% samples excited at 365 nm and monitored at different wavelengths.

Figure S20. Delayed emission spectra (at different delay time) of **BF$_2$bdk**-PhB-0.4% afterglow materials excited at 365 nm.

Figure S21. (A) Room-temperature emission decay profile (monitored at 470 nm) of **7**-TMB-PhB-0.2% excited by 365 nm of different excitation powers for 30 s. (B) Room-temperature emission decay profile (monitored at 501 nm) of **10**-TMB-PhB-

0.2% excited by 365 nm of different excitation powers for 30 s. (C) Room-temperature emission decay profile (monitored at 470 nm) of **7**-PhB-TMB-0.2% excited by 365 nm UV light at 2.1 mW/cm^2. (D) Room-temperature emission decay profile (monitored at 501 nm) of **10**-PhB-TMB-0.2% excited by 365 nm light at 2.1 mW/cm^2.

Figure S22. Chemical structures of 48, 56, 4CzIPN and coumarin emitter dopants, and room-temperature delayed emission spectra (0.36 min delay) of **7**-PhB-TMB-0.2% (excited at 365 nm) and 48/56/4CzIPN/coumarin-PhB-TMB-0.2% samples (excited at 320 nm).

Figure S23. HPLC profile of further purified TMB and purified PhB, denoted as TMB' and PhB', respectively.

Figure S24. Steady-state and delayed emission spectra (1 ms delay) and emission decay profile (monitored at 470 nm) of **7**-PhB-TMB-0.2% and **7**-PhB'-TMB'-0.2% afterglow materials excited at 365 nm.

Figure S25. (A) Photographs of **7**-PhB-TMB under 405 nm UV light and after removal of the UV light. (B) Steady-state and delayed emission (1 ms delay) spectra of **7**-PhB-TMB excited at 405 nm. (C) Emission decay profiles (1 ms delay, monitored at 470 nm) of **7**-PhB-TMB excited at 405 nm.

Table S3. The HOMO and LUMO levels obtained by DFT calculation at B3LYP/6-31g(d,p) level.

Figure S26. (A) Chemical structures of fluorene and tetracyanoethylene (TCNE). (B) Photographs of melt-cast samples of fluorene-PhB, fluorene-TCNE-PhB and TCNE-PhB under room light. (C) Photographs under room light of fluorene-PhB and TCNE-PhB samples before mixing, and fluorene-TCNE-PhB samples after mixing fluorene-PhB and TCNE-PhB samples by mechanical grinding.

Figure S27. Steady-state spectra, delayed emission spectra, and OLPL spectra (0.36 min delay) of **7**-PhB-TMB material at 470 nm.

Table S4. Photophysical property of **BF$_2$bdk**-PhB-0.2% afterglow materials.

Figure S28. Room temperature steady-state emission and delayed emission (1 ms delay) spectra of **BF$_2$bdk**-PhB-0.2% material under ambient conditions.

Figure S29. (A) Photographs of **7**-PhB under 365 nm UV light and after removal of the UV light. (B) Steady-state and delayed emission (1 ms delay) spectra of **7**-PhB at room temperature, as well as delayed emission (1 ms delay) spectrum of **7**-PhB at 77 K. (C) Schematic diagram of TADF afterglow mechanism of **7**-PhB materials.

Figure S30. The delayed emission decay of fresh-prepared **BF_2bdk**-PhB-0.2% samples excited at 365 nm and monitored at different wavelengths.

Figure S31. The steady-state and delayed emission spectra, emission decay spectra of **1**/**4**/**7**-PhB-0.001% (A) and **1**/**4**/**7**-PhB-0.01% (B) afterglow materials excited at 365 nm.

Figure S32. Steady-state and delayed emission (1 ms delay) spectra and phosphorescence decay of **BF_2bdk**-PhB-0.2% samples at 77 K excited at 365 nm.

Figure S33. (A) Steady-state and delayed emission (1 ms delay) spectra, emission decay profiles (monitored at 488/497 nm) of **8**/**9**-PhB-0.2% samples. (B) Steady-state and delayed emission (1 ms delay) spectra and phosphorescence decay of **8**/**9**-PhB-0.2% samples at 77 K excited at 365 nm.

Table S5. Dihedral angle, electronic energy, and free energy of radical cations in this work.

Figure S34. (A, B) Photographs of **7**-PhB-TMB and **1**-PhB-TMB materials at 77 K under daylight lamp, 365 nm ultraviolet light, and after removal of ultraviolet light. (C, E) Delayed emission spectra of **7**-PhB-TMB and **1**-PhB-TMB materials excited at 365 nm at 77 K. (D, F) Logarithmic plot of OLPL emission attenuation curve of **7**-PhB-TMB material at 77 K (monitored at 505 nm) and **1**-PhB-TMB material at 77 K (monitored at 501 nm).

Figure S35. Photographs of the afterglow property change of upon transferring **7**-PhB-TMB materials from liquid nitrogen to room temperature.

Figure S36. (A, B) Photographs of 7-PhB-**TMB1** and 7-PhB-**TMB2** under daylight lamp, 365 nm UV light and after removal of the UV light. (C) Delayed emission spectra (0.36 delay) of the 7-PhB-**TMB**, 7-PhB-**TMB1** and 7-PhB-**TMB2** materials excited at 365 nm. (D) Emission decay profiles (monitored at 470 nm) of 7-PhB-**TMB**, 7-PhB-**TMB1** and 7-PhB-**TMB2** materials excited at 365 nm.

Figure S37. (A) Molar absorption coefficient and (B) steady-state emission spectrum of compound **10** in dichloromethane (DCM).

Figure S38. (A) Photographs of **10**-PhB sample under 365 nm UV light and after removal of the UV light. (B) Steady-state and delayed emission (1 ms delay) spectra of **10**-PhB sample. (C) Emission decay profiles (monitored at 494 nm) of **10**-PhB sample excited at 365 nm.

Figure S39. (A) Variable-temperature delayed emission spectra (1 ms delay) of **10**-PhB materials excited at 365 nm. (B) Emission decay profiles (monitored at 542 nm) of **10**-PhB material at 77 K.

Figure S40. Percentage of different luminescent components obtained from emission decay profile (fluorescence plus TADF afterglow, and OLPL afterglow) in **10**-PhB-TMB-0.2% materials.

Figure S41. (A) Photographs of **10**-PhB-TMB melt-cast sample under 420 nm lamp and after removal of the lamp. (B) Steady-state and delayed emission (1 ms delay) spectra of **10**-PhB-TMB excited at 420 nm. (C) Delayed emission decay profiles (monitored at 497 nm) of **10**-PhB-TMB excited at 420 nm.

Figure S42. (A) Afterglow photographs of **10**-PhB-TMB materials and inorganic $Sr_2Al_{14}O_{25}/Eu^{2+}$, Dy^{3+} materials. (B) Emission decay profiles (monitored at 501 nm) of **10**-PhB-TMB materials and emission decay profiles (monitored at 515 nm) of inorganic $Sr_2Al_{14}O_{25}/Eu^{2+}$, Dy^{3+} materials.

Figure S43-S119. NMR, FT-IR, HRMS, single crystal structure of compounds **1-10**, **TMB1** and **TMB2**

Table S6-S26. Single crystal analysis data of compounds **2-5**, **7-9**.

1. Experimental Section

Materials

4-Methoxyphenylboronic acid (98%, Innochem), 2-bromotoluene (99%, Innochem), 2-bromoethylbenzene (99%, Adamas), 1-bromo-2-isopropylbenzene (RG, Adamas), 1-bromo-2-(tert-butyl) benzene (95%, Bide Pharmatech), 2,6-dimethylbromobenzene (98%, Innochem), 2-bromo-1,3-diethylbenzene (95%, Bide Pharmatech), 2-bromo-1,3-diisopropylbenzene (96%, Innochem), 4-methoxybiphenyl (98%, Adamas), 1-bromo-4-tert-butylbenzene (97%, Adamas), 4-bromocumene (RG, Adamas), palladium acetate (98%, Bide Pharmatech), potassium carbonate anhydrous (AR, Shanghai Dahe Chemical), *N*,*N*-dimethylformamide (99.5%, Acmec), acetic anhydride (AR, Sinopharm Chemical Reagent), boron trifluoride diethyl etherate (98%, TCI), acetyl chloride (98%, Aladdin), aluminum chloride (99%, Energy Chemical), sodium hydride (60%, Aladdin), methyl [1,1'-biphenyl]-4-carboxylate (95%, Bide Pharmatech), 2,2'-dimethyl[1,1'-biphenyl]-4,4'-diamine (RG, Adamas), 2,2'-bis(trifluoromethyl)benzidine (98%, Adamas), dimethyl sulfate (RG, Adamas), *N*,*N*,*N*',*N*'-tetramethylbenzidine (98%, Bide Pharmatech), phenyl benzoate (PhB) (99%, Energy Chemical).

Preparation of two- or three-component afterglow materials by melt casting method

For the preparation of **BF_2bdk**-PhB-0.2% melt-cast samples, 400 μL **BF_2bdk** in dichloromethane (1.0 mg/mL) and 200 mg phenyl benzoate (PhB) were added into a glass bottle (3 mL). After heating and solvent evaporation at 100 °C, the mixture was cooled to room temperature to obtain **BF_2bdk**-PhB-0.2% melt-cast samples that show afterglow properties. TMB-PhB-0.2% melt-cast samples were obtained by the same preparation method with TMB doping concentration of 0.2 wt%.

For the preparation of **BF_2bdk**-PhB-TMB-0.2% melt-cast samples, 400 μL **BF_2bdk** in dichloromethane (1.0 mg/mL), 200 μL TMB in dichloromethane (2.0 mg/mL) and 200 mg phenyl benzoate (PhB) were added into a glass bottle (3 mL).

After heating and solvent evaporation at 100 °C, the mixture was cooled to room temperature to obtain **BF_2bdk**-PhB-TMB-0.2% melt-cast samples that show afterglow properties were obtained. **BF_2bdk**-PhB-TMB1-0.2% and **BF_2bdk**-PhB-TMB2-0.2% melt-cast samples were obtained by the same preparation method.

For the preparation of TMB-PhB-dopant-0.2% melt-cast samples, 400 μL dopant emitters in dichloromethane (1.0 mg/mL), 200 μL TMB in dichloromethane (2.0 mg/mL) and 200 mg phenyl benzoate (PhB) were added into a glass bottle (3 mL). After heating and solvent evaporation at 100 °C, the mixture was cooled to room temperature to obtain TMB-PhB-dopant-0.2% melt-cast samples that show afterglow properties were obtained.

These melt-cast samples are used for photophysical measurements and others, where PhB matrices of these samples are in crystalline state.

Synthesis

Synthesis of compound 1 via Suzuki-Miyaura cross coupling reaction and cascade reaction

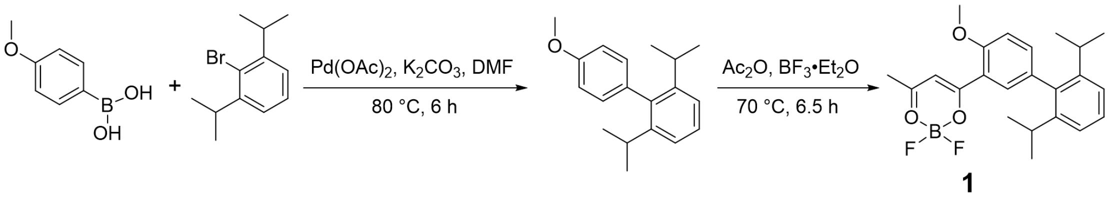

Into a round bottom flask were added 4-methoxyphenylboronic acid (303.7 mg, 2.0 mmol), 2-bromo-1,3-diisopropylbenzene (424.4 mg, 1.8 mmol), palladium acetate (4.9 mg, 0.02 mmol), potassium carbonate anhydrous (824.0 mg, 6 mmol) and *N*,*N*-dimethylformamide (2.5 mL). The reaction mixture was stirred at 80 °C for 6 h. Then, the reaction mixture was washed by saturated sodium chloride solution and extracted by dichloromethane. The crude product was purified by column chromatography over silica gel using petroleum ether/dichloromethane (3:1) as eluent to give 182.7 mg product (yield, 38.7%). The obtained product (182.7 mg) was further reacted with acetic anhydride (1.7 mL) and boron trifluoride diethyl etherate (255 μL) at 70 °C for 6.5 h. Then the reaction was quenched by adding the reaction mixture dropwise into

cold water. The mixed solution was washed with a saturated sodium chloride solution and then extracted with dichloromethane. The crude product was purified by column chromatography over silica gel using petroleum ether/dichloromethane (3:2) as eluent to give yellow solids with an isolation yield of 68.5% (186.3 mg). Compound **1** was further purified by three cycles of recrystallization in spectroscopic grade dichloromethane/*n*-hexane. ^{1}H NMR (400 MHz, Chloroform-*d*) δ 7.99 (d, *J* = 2.3 Hz, 1H), 7.41 (dd, *J* = 8.5, 2.3 Hz, 1H), 7.35 (d, *J* = 7.7 Hz, 1H), 7.20 (d, *J* = 7.8 Hz, 2H), 7.12 – 7.03 (m, 2H), 4.04 (s, 3H), 2.59 – 2.47 (m, 2H), 2.40 (s, 3H), 1.09 (d, *J* = 6.8 Hz, 6H), 1.06 (d, *J* = 6.9 Hz, 6H). ^{13}C NMR (101 MHz, Chloroform-*d*) δ 192.14, 180.78, 159.69, 147.07, 137.72, 137.38, 133.65, 132.57, 128.45, 122.80, 120.05, 111.78, 102.67, 56.08, 30.43, 24.94, 24.28, 24.07. ^{19}F NMR (376 MHz, Chloroform-*d*) δ -139.17, -139.23. ^{11}B NMR (128 MHz, Chloroform-*d*) δ -0.07. FT-IR (KBr, cm^{-1}): ν 3158.1, 3064.6, 2960.2, 2924.8, 2865.6, 1613.3, 1594.3, 1533.0, 1504.3, 1467.6, 1441.8, 1411.8, 1369.4, 1329.7, 1281.8, 1265.1, 1250.4, 1207.5, 1166.9, 1104.2, 1081.6, 1050.1, 1033.1, 1016.6, 978.7, 905.5, 834.3, 821.3, 807.2, 762.2, 660.8, 649.2, 632.7, 614.5, 578.1, 558.8, 510.7. HRMS m/z found (calcd for $C_{23}H_{27}BF_2O_3NH_4^+$ $[M+NH_4]^+$): 418.2329 (418.2360).

Synthesis of compound 2 via Suzuki-Miyaura cross coupling reaction and cascade reaction

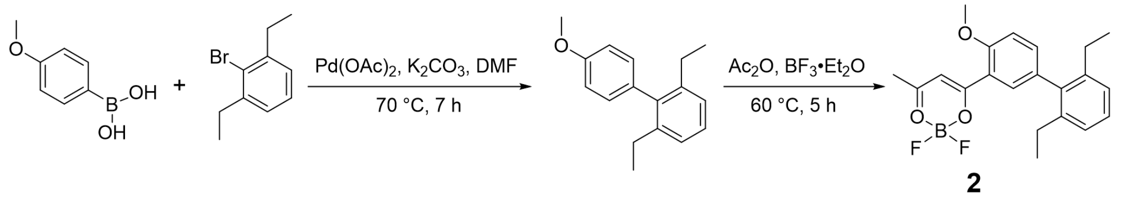

2

Into a Schlenk flask were added 4-methoxyphenylboronic acid (485.7 mg, 3.2 mmol), 2-bromo-1,3-diethylbenzene (648.3 mg, 3.1 mmol), palladium acetate (9.4 mg, 0.04 mmol), potassium carbonate anhydrous (1208.6 mg, 8.7 mmol) and *N*,*N*-dimethylformamide (2.5 mL). The solution was degassed by the freeze-pump-thaw technique. The reaction mixture was stirred at 70 °C for 7 h. The reaction mixture was washed by saturated sodium chloride solution and then extracted by dichloromethane.

The crude product was purified by column chromatography over silica gel using petroleum ether/dichloromethane (1:1) as eluent to give 170.4 mg product (yield, 23.3%). The obtained product (170.4 mg) was further reacted with acetic anhydride (3.5 mL) and boron trifluoride diethyl etherate (500 μL) at 60 °C for 5 h. Then the reaction was quenched by adding the reaction mixture dropwise into cold water. The mixed solution was washed with a saturated sodium chloride solution and then extracted with dichloromethane. The crude product was purified by column chromatography over silica gel using petroleum ether/dichloromethane (1:1) as eluent to give yellow solids with an isolation yield of 46.4% (122.5 mg). Compound **2** was further purified by three cycles of recrystallization in spectroscopic grade dichloromethane/*n*-hexane. ^{1}H NMR (400 MHz, Chloroform-*d*) δ 8.01 (d, J = 2.3 Hz, 1H), 7.42 (d, J = 2.3 Hz, 1H), 7.32 – 7.26 (m, 1H), 7.14 (d, J = 7.6 Hz, 2H), 7.11 – 7.06 (m, 2H), 4.03 (s, 3H), 2.40 (s, 3H), 2.29 (m, 4H), 1.01 (t, J = 7.6 Hz, 6H). ^{13}C NMR (101 MHz, Chloroform-*d*) δ 192.16, 180.71, 159.73, 142.46, 138.57, 137.81, 133.55, 132.79, 128.18, 125.82, 120.12, 111.86, 102.60, 56.08, 27.01, 24.95, 15.53. ^{19}F NMR (376 MHz, Chloroform-*d*) δ -139.10, -139.16. ^{11}B NMR (128 MHz, Chloroform-*d*) δ -0.07. FT-IR (KBr, cm^{-1}): ν 3162.1, 3060.6, 2963.4, 2932.7, 2869.2, 1614.1, 1593.6, 1530.4, 1456.4, 1413.0, 1368.8, 1335.5, 1268.8, 1251.1, 1209.3, 1165.6, 1103.9, 1049.0, 1021.1, 977.3, 905.3, 839.4, 829.7, 812.7, 763.0, 658.9, 626.3, 573.0, 552.3, 511.8. HRMS m/z found (calcd for $C_{21}H_{23}BF_2O_3NH_4^+$ $[M+NH_4]^+$): 390.2022 (390.2047).

Synthesis of compound 3 via Suzuki-Miyaura cross coupling reaction and cascade reaction

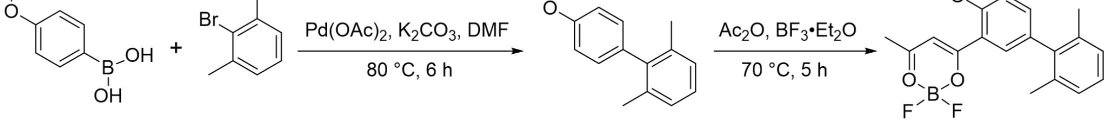

3

Into a round bottom flask were added 4-methoxyphenylboronic acid (460.1 mg, 3.0 mmol), 2,6-dimethylbromobenzene (556.9 mg, 3 mmol), palladium acetate (7.1

mg, 0.03 mmol), potassium carbonate anhydrous (1243.0 mg, 9 mmol) and *N*,*N*-dimethylformamide (3 mL). The reaction mixture was stirred at 80 °C for 6 h. Then, the reaction mixture was washed by saturated sodium chloride solution and then extracted by dichloromethane. The crude product was purified by column chromatography over silica gel using petroleum ether/dichloromethane (2:1) as eluent to give 298.6 mg product (yield, 46.9%). The obtained product (298.6 mg) was further reacted with acetic anhydride (3.5 mL) and boron trifluoride diethyl etherate (525 μL) at 70 °C for 5 h. Then the reaction was quenched by adding the reaction mixture dropwise into cold water. The mixed solution was washed with a saturated sodium chloride solution and then extracted with dichloromethane. The crude product was purified by column chromatography over silica gel using petroleum ether/dichloromethane (3:1) as eluent to give yellow solids with an isolation yield of 66.7% (322.8 mg). Compound **3** was further purified by three cycles of recrystallization in spectroscopic grade dichloromethane/hexane. ^{1}H NMR (400 MHz, Chloroform-*d*) δ 7.98 (d, *J* = 2.3 Hz, 1H), 7.40 (dd, *J* = 8.6, 2.3 Hz, 1H), 7.20 – 7.14 (m, 1H), 7.14 – 7.05 (m, 4H), 4.03 (s, 3H), 2.40 (s, 3H), 2.01 (s, 6H). ^{13}C NMR (101 MHz, Chloroform-*d*) δ 192.19, 180.67, 159.74, 139.66, 137.48, 136.29, 134.26, 132.52, 127.68, 127.58, 120.41, 112.25, 102.57, 56.10, 24.96, 21.01. ^{19}F NMR (376 MHz, Chloroform-*d*) δ -139.02, -139.08. ^{11}B NMR (128 MHz, Chloroform-*d*) δ -0.07. FT-IR (KBr, cm^{-1}): ν 3165.4, 2955.7, 2917.5, 2853.5, 1614.7, 1596.8, 1531.4, 1465.9, 1444.8, 1409.2, 1363.6, 1336.5, 1265.8, 1253.5, 1161.3, 1103.6, 1079.2, 1054.6, 1042.0, 977.0, 903.5, 816.0, 832.9, 778.7, 657.5, 640.2, 626.2, 610.8, 575.8, 536.9, 507.5. HRMS m/z found (calcd for $C_{19}H_{19}BF_2O_3NH_4^+$ $[M+NH_4]^+$): 362.1718 (362.1734).

Synthesis of compound 4 via Suzuki-Miyaura cross coupling reaction and cascade reaction

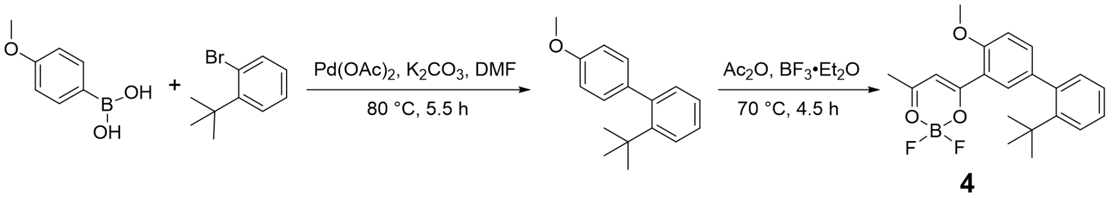

Into a round bottom flask were added 4-methoxyphenylboronic acid (305.2 mg, 2.0 mmol), 1-bromo-2-(tert-butyl) benzene (420.0 mg, 2 mmol), palladium acetate (5.1 mg, 0.02 mmol), potassium carbonate anhydrous (830.7 mg, 6 mmol) and *N*,*N*-dimethylformamide (2.5 mL). The reaction mixture was stirred at 80 °C for 5.5 h. Then, the reaction mixture was washed by saturated sodium chloride solution and then extracted by dichloromethane. The crude product was purified by column chromatography over silica gel using petroleum ether/dichloromethane (2:1) as eluent to give 240.2 mg product (yield, 50.7%). The obtained product (197.7 mg) was further reacted with acetic anhydride (2.5 mL) and boron trifluoride diethyl etherate (370 μL) at 70 °C for 4.5 h. Then the reaction was quenched by adding the reaction mixture dropwise into cold water. The mixed solution was washed with a saturated sodium chloride solution and then extracted with dichloromethane. The crude product was purified by column chromatography over silica gel using petroleum ether/dichloromethane (3:2) as eluent to give yellow solids with an isolation yield of 46.6% (142.6 mg). Compound **4** was further purified by three cycles of recrystallization in spectroscopic grade dichloromethane/*n*-hexane. ^{1}H NMR (400 MHz, Chloroform-*d*) δ 8.09 (d, *J* = 2.4 Hz, 1H), 7.53 (dd, *J* = 8.1, 1.3 Hz, 1H), 7.50 (dd, *J* = 8.5, 2.4 Hz, 1H), 7.35 – 7.29 (m, 1H), 7.17 (td, *J* = 7.4, 1.3 Hz, 1H), 7.09 (s, 1H), 7.00 (d, *J* = 8.6 Hz, 1H), 6.95 (dd, *J* = 7.5, 1.7 Hz, 1H), 4.03 (s, 3H), 2.39 (s, 3H), 1.18 (s, 9H). ^{13}C NMR (101 MHz, Chloroform-*d*) δ 192.13, 180.71, 159.69, 148.07, 139.81, 138.49, 137.98, 132.98, 132.80, 127.88, 126.97, 125.29, 119.19, 110.92, 102.63, 56.11, 36.61, 32.81, 24.94. ^{19}F NMR (376 MHz, Tetrachloroethane-d_2, 80 °C) δ -138.09. ^{11}B NMR (128 MHz, Chloroform-*d*) δ -0.08. FT-IR (KBr, cm^{-1}): ν 3168.4, 3064.5, 2963.9, 2841.4, 1613.0, 1530.2, 1482.0, 1463.1, 1434.9, 1411.4, 1365.3, 1336.5, 1272.7, 1252.9, 1179.1, 1104.5, 1081.6, 1052.7, 1017.7, 977.8, 905.7, 834.2,

761.4, 672.8, 653.2, 630.2, 613.4, 571.1, 538.3, 493.1. HRMS m/z found (calcd for $C_{21}H_{23}BF_2O_3NH_4^+$ $[M+NH_4]^+$): 390.2033 (390.2047).

Synthesis of compound 5 via Suzuki-Miyaura cross coupling reaction and cascade reaction

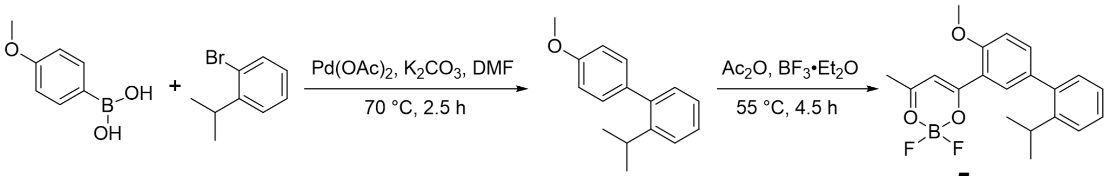

5

Into a round bottom flask were added 4-methoxyphenylboronic acid (457.2 mg, 3.0 mmol), 1-bromo-2-isopropylbenzene (596.1 mg, 3 mmol), palladium acetate (8.4 mg, 0.04 mmol), potassium carbonate anhydrous (1243.4 mg, 9 mmol) and *N*,*N*-dimethylformamide (2.5 mL). The reaction mixture was stirred at 70 °C for 2.5 h. Then, the reaction mixture was washed by saturated sodium chloride solution and then extracted by dichloromethane. The crude product was purified by column chromatography over silica gel using petroleum ether/dichloromethane (1:1) as eluent to give 525.7 mg product (yield, 77.6%). The obtained product (380.3 mg) was further reacted with acetic anhydride (5 mL) and boron trifluoride diethyl etherate (740 μL) at 55 °C for 4.5 h. Then the reaction was quenched by adding the reaction mixture dropwise into cold water. The mixed solution was washed with a saturated sodium chloride solution and then extracted with dichloromethane. The crude product was purified by column chromatography over silica gel using petroleum ether/dichloromethane (3:2) as eluent to give yellow solids with an isolation yield of 82.8% (498.3 mg). Compound **5** was further purified by three cycles of recrystallization in spectroscopic grade dichloromethane/*n*-hexane. ^{1}H NMR (400 MHz, Chloroform-*d*) δ 8.12 (d, J = 2.4 Hz, 1H), 7.53 (dd, J = 8.6, 2.4 Hz, 1H), 7.37 (dd, J = 8.7, 1.8 Hz, 2H), 7.24 – 7.18 (m, 1H), 7.16 – 7.11 (m, 1H), 7.11 – 7.03 (m, 2H), 4.03 (s, 3H), 2.99 – 2.90 (m, 1H), 2.40 (s, 3H), 1.16 (d, J = 6.8 Hz, 6H). ^{13}C NMR (101 MHz, Chloroform-*d*) δ 192.24, 180.63, 159.86, 146.49, 138.90, 137.39, 135.28, 132.56, 130.08, 128.29, 125.81, 125.64, 119.98, 111.73, 102.58, 56.15, 29.60,

24.97, 24.30. ^{19}F NMR (376 MHz, Chloroform-*d*) δ -139.05, -139.11. ^{11}B NMR (128 MHz, Chloroform-*d*) δ -0.05. FT-IR (KBr, cm^{-1}): ν 3162.8, 3014.4, 2968.8, 2870.0, 1613.8, 1526.5, 1484.4, 1471.1, 1441.4, 1413.4, 1362.1, 1339.3, 1275.9, 1266.6, 1204.2, 1180.8, 1159.0, 1145.4, 1104.2, 1080.6, 1057.4, 1048.3, 1017.7, 978.4, 918.9, 908.8, 835.5, 814.7, 770.1, 691.5, 655.5, 625.8, 613.0, 578.2, 559.0, 532.9, 491.6, 466.9. HRMS m/z found (calcd for $C_{20}H_{21}BF_2O_3NH_4^+$ $[M+NH_4]^+$): 376.1876 (376.1891).

Synthesis of compound 6 via Suzuki-Miyaura cross coupling reaction and cascade reaction

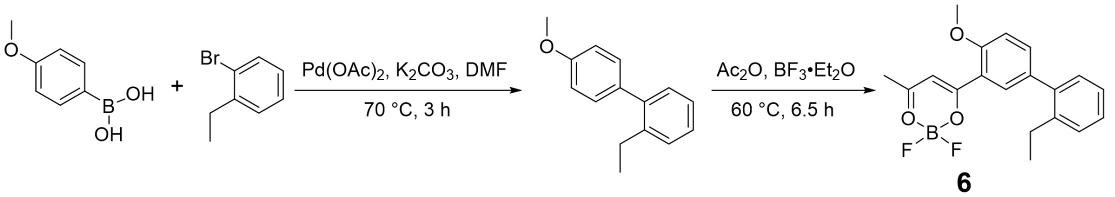

6

Into a round bottom flask were added 4-methoxyphenylboronic acid (454.3 mg, 3.0 mmol), 2-bromoethylbenzene (530.1 mg, 2.9 mmol), palladium acetate (6.7 mg, 0.03 mmol), potassium carbonate anhydrous (1241.3 mg, 9 mmol) and *N*,*N*-dimethylformamide (3 mL). The reaction mixture was stirred at 70 °C for 3 h. Then, the reaction mixture was washed by saturated sodium chloride solution and then extracted by dichloromethane. The crude product was purified by column chromatography over silica gel using petroleum ether/dichloromethane (1:1) as eluent to give 504.0 mg product (yield, 82.9%). The obtained product (504.0 mg) was further reacted with acetic anhydride (5.7 mL) and boron trifluoride diethyl etherate (850 μL) at 60 °C for 6.5 h. Then the reaction was quenched by adding the reaction mixture dropwise into cold water. The mixed solution was washed with a saturated sodium chloride solution and then extracted with dichloromethane. The crude product was purified by column chromatography over silica gel using petroleum ether/dichloromethane (3:2) as eluent to give yellow solids with an isolation yield of 55.9% (442.3 mg). Compound **6** was further purified by three cycles of recrystallization in spectroscopic grade dichloromethane/*n*-hexane. ^{1}H NMR (400

MHz, Chloroform-*d*) δ 8.14 (d, *J* = 2.4 Hz, 1H), 7.55 (dd, *J* = 8.6, 2.4 Hz, 1H), 7.36 – 7.27 (m, 2H), 7.23 (ddd, *J* = 7.4, 6.0, 2.6 Hz, 2H), 7.16 (dd, *J* = 7.3, 1.2 Hz, 1H), 7.11 – 7.02 (m, 2H), 4.03 (s, 3H), 2.56 (q, *J* = 7.5 Hz, 2H), 2.40 (s, 3H), 1.10 (t, *J* = 7.6 Hz, 3H). ^{13}C NMR (101 MHz, Chloroform-d) δ 192.25, 180.62, 159.87, 141.74, 139.47, 137.31, 135.21, 132.52, 130.06, 128.83, 128.11, 125.89, 119.98, 111.80, 102.58, 56.15, 26.26, 24.97, 15.64. ^{19}F NMR (376 MHz, Chloroform-*d*) δ -139.02, -139.08. ^{11}B NMR (128 MHz, Chloroform-*d*) δ -0.05. FT-IR (KBr, cm^{-1}): ν 3165.4, 3013.2, 2966.3, 2931.0, 2873.2, 1614.3, 1527.9, 1483.4, 1471.6, 1433.5, 1413.1, 1359.6, 1342.2, 1285.7, 1269.4, 1181.0, 1156.4, 1140.4, 1104.7, 1082.7, 1059.5, 1017.7, 977.8, 916.4, 906.2, 875.5, 836.6, 816.8, 765.8, 692.3, 654.5, 623.7, 613.2, 574.7, 543.1, 493.8, 470.4, 447.4, 436.8, 415.3. HRMS m/z found (calcd for $C_{19}H_{19}BF_2O_3NH_4^+$ $[M+NH_4]^+$): 362.1728 (362.1734).

Synthesis of compound 7 via Suzuki-Miyaura cross coupling reaction and cascade reaction

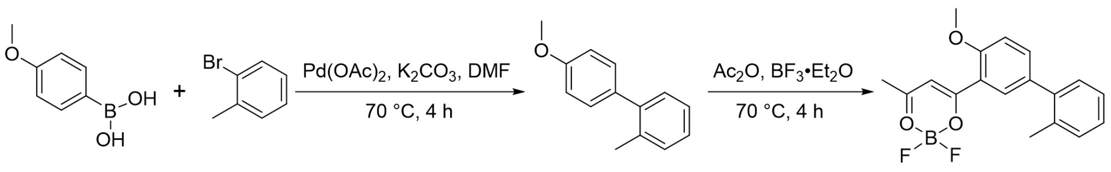

7

Into a round bottom flask were added 4-methoxyphenylboronic acid (460.2 mg, 3.0 mmol), 2-bromotoluene (525.8 mg, 3.1 mmol), palladium acetate (7.9 mg, 0.04 mmol), potassium carbonate anhydrous (1242.3 mg, 9 mmol) and *N*,*N*-dimethylformamide (3.5 mL). The reaction mixture was stirred at 70 °C for 4 h. Then, the reaction mixture was washed by saturated sodium chloride solution and then extracted by dichloromethane. The crude product was purified by column chromatography over silica gel using petroleum ether/dichloromethane (1:1) as eluent to give 580.7 mg product (yield, 97.7%). The obtained product (447.5 mg) was further reacted with acetic anhydride (5 mL) and boron trifluoride diethyl etherate (740 μL) at 70 °C for 4 h. Then the reaction was quenched by adding the reaction mixture dropwise into cold water. The mixed solution was washed with a saturated sodium

chloride solution and then extracted with dichloromethane. The crude product was purified by column chromatography over silica gel using petroleum ether/dichloromethane (4:1) as eluent to give yellow solids with an isolation yield of 64.0% (476.7 mg). Compound **7** was further purified by three cycles of recrystallization in spectroscopic grade dichloromethane/*n*-hexane. ^{1}H NMR (400 MHz, Chloroform-*d*) δ 8.14 (d, *J* = 2.4 Hz, 1H), 7.59 – 7.53 (m, 1H), 7.27 (d, *J* = 1.4 Hz, 2H), 7.25 – 7.17 (m, 2H), 7.10 – 7.03 (m, 2H), 4.02 (s, 3H), 2.42 – 2.38 (m, 3H), 2.25 (s, 3H). ^{13}C NMR (101 MHz, Chloroform-*d*) δ 192.26, 180.62, 159.86, 139.81, 137.31, 135.43, 135.22, 132.50, 130.56, 129.83, 127.86, 126.09, 120.04, 111.83, 102.56, 56.15, 24.96, 20.51. ^{19}F NMR (376 MHz, Chloroform-*d*) δ -138.99, -139.05. ^{11}B NMR (128 MHz, Chloroform-*d*) δ -0.05. FT-IR (KBr, cm^{-1}): ν 3451.2, 3173.4, 2946.0, 2845.6, 1614.9, 1528.2, 1482.3, 1456.1, 1414.2, 1362.3, 1340.6, 1303.8, 1280.6, 1261.9, 1182.0, 1106.1, 1057.9, 1018.9, 977.5, 904.5, 831.4, 813.8, 760.5, 728.1, 692.1, 653.0, 615.1, 573.5, 508.2. HRMS m/z found (calcd for $C_{18}H_{17}BF_2O_3NH_4^+$ $[M+NH_4]^+$): 348.1578 (348.1578).

Synthesis of compound 8 via Suzuki-Miyaura cross coupling reaction and cascade reaction

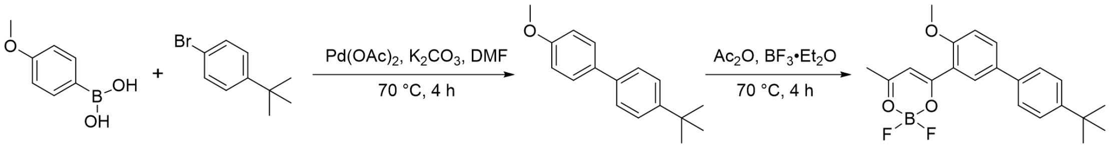

Into a round bottom flask were added 4-methoxyphenylboronic acid (470.6 mg, 3.1 mmol), 1-bromo-4-tert-butylbenzene (634.0 mg, 3 mmol), palladium acetate (7.9 mg, 0.04 mmol), potassium carbonate anhydrous (1246.6 mg, 9 mmol) and *N*,*N*-dimethylformamide (4 mL). The reaction mixture was stirred at 70 °C for 4 h. Then, the reaction mixture was washed by saturated sodium chloride solution and then extracted by dichloromethane. The crude product was purified by column chromatography over silica gel using petroleum ether/dichloromethane (1:3) as eluent to give 497.6 mg product (yield, 69.6%). The obtained product (497.6 mg) was further

reacted with acetic anhydride (5.5 mL) and boron trifluoride diethyl etherate (745 μL) at 70 °C for 4 h. Then the reaction was quenched by adding the reaction mixture dropwise into cold water. The mixed solution was washed with a saturated sodium chloride solution and then extracted with dichloromethane. The crude product was purified by column chromatography over silica gel using petroleum ether/dichloromethane (1:1) as eluent to give yellow solids with an isolation yield of 69.5% (535.8 mg). Compound **8** was further purified by three cycles of recrystallization in spectroscopic grade dichloromethane/*n*-hexane. ^{1}H NMR (400 MHz, Chloroform-*d*) δ 8.38 (d, *J* = 2.5 Hz, 1H), 7.81 (dd, *J* = 8.7, 2.5 Hz, 1H), 7.54 – 7.49 (m, 2H), 7.49 - 7.44 (m, 2H), 7.07 (t, *J* = 4.4 Hz, 2H), 4.00 (s, 3H), 2.39 (s, 3H), 1.36 (s, 9H). ^{13}C NMR (101 MHz, Chloroform-*d*) δ 192.25, 180.64, 160.09, 150.77, 136.20, 134.95, 134.42, 130.16, 126.52, 125.98, 120.47, 112.50, 102.57, 56.16, 34.65, 31.41, 24.94. ^{19}F NMR (376 MHz, Chloroform-*d*) δ -138.61, -138.67. ^{11}B NMR (128 MHz, Chloroform-*d*) δ -0.01. FT-IR (KBr, cm^{-1}): ν 3169.9, 3039.5, 2961.0, 2870.5, 2840.4, 1612.8, 1529.7, 1495.1, 1464.8, 1424.0, 1397.4, 1365.2, 1340.2, 1268.2, 1183.5, 1162.2, 1104.9, 1080.9, 1052.8, 1022.1, 978.1, 904.0, 844.7, 815.0, 742.7, 660.3, 644.8, 608.7, 574.0, 550.2, 521.7, 492.6, 470.0. HRMS m/z found (calcd for $C_{21}H_{23}BFO_3$ [M-F]$^{+}$): 353.1742 (353.1719).

Synthesis of compound 9 via Suzuki-Miyaura cross coupling reaction and cascade reaction

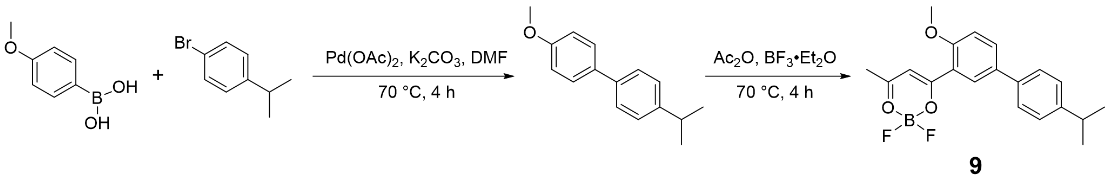

9

Into a round bottom flask were added 4-methoxyphenylboronic acid (468.6 mg, 3.1 mmol), 4-bromocumene (598.5 mg, 3 mmol), palladium acetate (8.9 mg, 0.04 mmol), potassium carbonate anhydrous (1237.9 mg, 9 mmol) and *N*,*N*-dimethylformamide (4 mL). The reaction mixture was stirred at 70 °C for 4 h. Then, the reaction mixture was washed by saturated sodium chloride solution and then

extracted by dichloromethane. The crude product was purified by column chromatography over silica gel using petroleum ether/dichloromethane (3:1) as eluent to give 378.1 mg product (yield, 55.6%). The obtained product (378.1 mg) was further reacted with acetic anhydride (4.5 mL) and boron trifluoride diethyl etherate (620 μL) at 70 °C for 4 h. Then the reaction was quenched by adding the reaction mixture dropwise into cold water. The mixed solution was washed with a saturated sodium chloride solution and then extracted with dichloromethane. The crude product was purified by column chromatography over silica gel using petroleum ether/dichloromethane (1:1) as eluent to give yellow solids with an isolation yield of 66.4% (397.4 mg). Compound **9** was further purified by three cycles of recrystallization in spectroscopic grade dichloromethane/*n*-hexane. ^{1}H NMR (400 MHz, Chloroform-*d*) δ 8.36 (d, *J* = 2.4 Hz, 1H), 7.79 (dd, *J* = 8.7, 2.5 Hz, 1H), 7.52 – 7.46 (m, 2H), 7.30 (d, *J* = 8.2 Hz, 2H), 7.10 – 7.02 (m, 2H), 3.99 (s, 3H), 2.94 (m, 1H), 2.38 (s, 3H), 1.28 (d, *J* = 6.9 Hz, 6H). ^{13}C NMR (101 MHz, Chloroform-*d*) δ 192.24, 180.63, 160.08, 148.51, 136.60, 134.96, 134.52, 130.14, 127.11, 126.79, 120.45, 112.50, 102.58, 56.16, 33.89, 24.95, 24.07. ^{19}F NMR (376 MHz, Chloroform-*d*) δ -138.61, -138.67. ^{11}B NMR (128 MHz, Chloroform-*d*) δ -0.01. FT-IR (KBr, cm^{-1}): ν 3170.0, 2962.3, 2871.1, 1613.5, 1537.3, 1496.0, 1461.7, 1429.6, 1401.0, 1377.2, 1361.4, 1340.5, 1290.3, 1263.9, 1178.0, 1155.2, 1105.7, 1080.8, 1056.4, 1045.0, 1018.2, 977.7, 905.6, 839.3, 818.6, 667.1, 652.2, 609.6, 574.2, 555.0, 541.8. HRMS m/z found (calcd for $C_{20}H_{21}BFO_3$ [M-F]$^{+}$): 339.1567 (339.1563).

Synthesis of compound 10 via acetylation reaction and Claisen condensation reaction

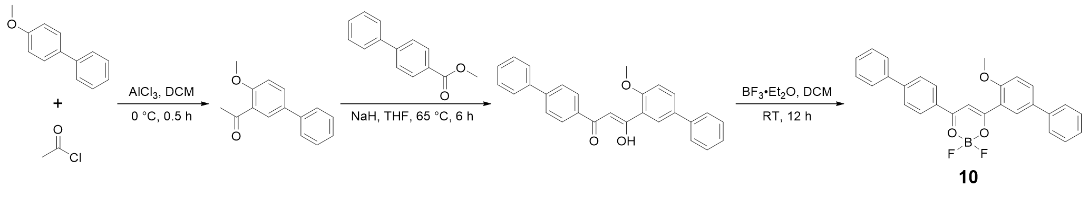

Into a round bottom flask were added 4-methoxybiphenyl (2.41 g, 13.1 mmol), acetyl chloride (1.0 mL, 13.1 mmol), and dichloromethane (15 mL). The reaction

mixture was stirred at 0 °C for 10 minutes, followed by the addition of aluminum trichloride (1.76 g, 13.2 mmol). The mixture was then allowed to continue to react for 30 minutes. Then, the reaction was quenched with dilute hydrochloric acid solution. The resulting mixture was washed with saturated sodium chloride solution, and the organic phase was extracted with dichloromethane. The crude product was purified by column chromatography over silica gel using petroleum ether/dichloromethane (1:1) as eluent to give 861.2 mg product (yield, 29.1%). The obtained product (452.4 mg, 2 mmol) was dissolved in anhydrous tetrahydrofuran (5 mL) along with sodium hydride (300 mg). After stirring at 0 °C for 30 minutes, methyl [1,1'-biphenyl]-4-carboxylate (510.5 mg, 2.4 mmol) was added. The reaction mixture was then heated to 65 °C under oil bath conditions for 6 hours. Subsequently, the reaction was quenched with saturated ammonium chloride solution. The mixture was washed with saturated sodium chloride solution, and the organic layer was extracted with dichloromethane. The reaction solution was concentrated to approximately 10 mL using a rotary evaporator. Following this, 1.5 mL of boron trifluoride etherate was added dropwise to the concentrated solution, and the reaction was allowed to proceed at room temperature for 12 hours. The resulting mixture was washed with saturated sodium chloride solution, and the organic layer was extracted with dichloromethane. The crude product was purified by column chromatography on silica gel using a petroleum ether/dichloromethane (3:1) eluent, yielding an orange solid with a separation yield of 40.0% (363.7 mg). Compound **10** was further purified by three cycles of recrystallization in spectroscopic grade dichloromethane/*n*-hexane. ^{1}H NMR (500 MHz, Chloroform-*d*) δ 8.51 (d, *J* = 3.0 Hz, 1H), 8.26 – 8.23 (m, 2H), 7.87 (d, *J* = 15.8 Hz, 2H), 7.84 – 7.80 (m, 2H), 7.71 (d, *J* = 7.5 Hz, 2H), 7.66 – 7.62 (m, 2H), 7.53 (d, *J* = 8.1 Hz, 2H), 7.51 (d, *J* = 13.8 Hz, 2H), 7.48 (s, 1H), 7.41 (d, *J* = 7.2 Hz, 1H), 7.18 (d, *J* = 8.5 Hz, 1H), 4.14 (s, 3H). ^{13}C NMR (101 MHz, Chloroform-*d*) δ 181.34, 179.45, 159.09, 146.67, 138.29, 138.05, 133.74, 133.53, 130.11, 129.20, 128.58, 128.10, 127.93, 127.77, 126.62, 126.55, 126.31, 125.79, 120.26, 111.47, 97.74, 55.32. ^{19}F NMR (376 MHz, Chloroform-*d*) δ -139.47, -139.53. ^{11}B NMR (128 MHz, Chloroform-*d*) δ 0.35. FT-IR (KBr, cm^{-1}): ν 3421.8, 3031.4, 1603.9, 1566.4, 1537.1,

1484.7, 1409.1, 1352.6, 1312.9, 1297.4, 1264.0, 1208.3, 1181.8, 1163.7, 1095.7, 1071.4, 1037.8, 1005.8, 955.8, 859.4, 825.4, 765.0, 743.7, 698.0, 653.3, 622.9, 605.4, 580.8, 563.3, 493.7. HRMS m/z found (calcd for $C_{28}H_{21}BFO_3$ [M-F]$^+$): 435.1579 (435.1568).

Synthesis of TMB1

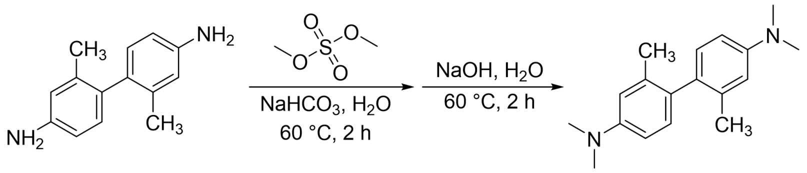

Into a round bottom flask were added 2,2'-dimethyl[1,1'-biphenyl]-4,4'-diamine (1.34 g, 5.0 mmol), dimethyl sulfate (3 mL, 30 mmol), sodium hydrogen carbonate (4.0 g) and deionized water (15 mL). The reaction mixture was stirred at 60 °C for 2 h, then 25 mL of 60% sodium hydroxide aqueous solution was added and the reaction continued for a further 2 h. Then, the reaction mixture was washed by deionized water and extracted by dichloromethane. The crude product was purified by column chromatography over silica gel using ethyl acetate/dichloromethane (50:1) as eluent to give 230.0 mg product (yield, 17.0%). **TMB1** was further purified by three cycles of recrystallization in spectroscopic grade dichloromethane/n-hexane. ^{1}H NMR (400 MHz, Chloroform-*d*) δ 6.99 (d, *J* = 8.2 Hz, 2H), 6.65 (d, *J* = 2.7 Hz, 2H), 6.62 (dd, *J* = 8.3, 2.7 Hz, 2H), 2.97 (s, 12H), 2.07 (s, 6H). ^{13}C NMR (101 MHz, Chloroform-*d*) δ 149.63, 137.22, 130.83, 130.55, 113.91, 110.07, 40.85, 20.66. HRMS m/z found (calcd for $C_{18}H_{25}N_2^+$ [M+H]$^+$): 269.20108 (269.2013).

Synthesis of TMB2

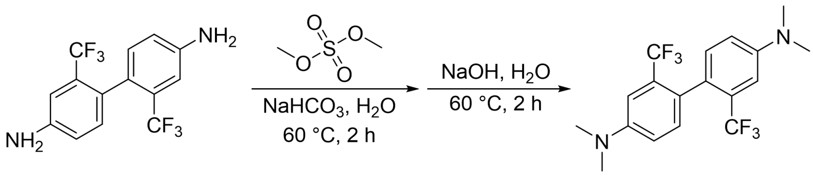

Into a round bottom flask were added 2,2'-bis(trifluoromethyl)benzidine (1.6 g, 5.0 mmol), dimethyl sulfate (3 mL, 30 mmol), sodium hydrogen carbonate (4.0 g) and

deionized water (15 mL). The reaction mixture was stirred at 60 °C for 2 h, then 25mL of 60% sodium hydroxide aqueous solution was added and the reaction continued for a further 2 h. Then, the reaction mixture was washed by deionized water and extracted by dichloromethane. The crude product was purified by column chromatography over silica gel using ethyl acetate/dichloromethane (100:1) as eluent to give 91.0 mg product (yield, 4.8%). **TMB2** was further purified by three cycles of recrystallization in spectroscopic grade dichloromethane/n-hexane. ^{1}H NMR (400 MHz, Chloroform-*d*) δ 7.11 (d, J = 8.6 Hz, 2H), 6.99 (d, J = 2.8 Hz, 2H), 6.80 (dd, J = 8.6, 2.8 Hz, 2H), 3.02 (s, 12H). ^{13}C NMR (101 MHz, Chloroform-*d*) δ 148.31, 132.17, 128.71, 128.42, 124.71, 124.06, 121.98, 112.63, 107.97, 39.30. ^{19}F NMR (376 MHz, Chloroform-*d*) δ -58.31. HRMS m/z found (calcd for $C_{18}H_{19}F_6N_2^+$ $[M+H]^+$): 377.14397 (377.1447).

Physical measurements and instrumentation

Nuclear magnetic resonance (NMR) spectra were recorded on a JEOL Fourier-transform NMR spectrometer (400 MHz), including ^{1}H NMR, ^{13}C{^{1}H} NMR, ^{19}F{^{1}H} NMR, and ^{11}B{^{1}H} NMR. High-resolution mass spectrometry (HRMS) analyses were performed by Agilent Technologies 5973N and Thermo Fisher Scientific LTQ FT Ultra mass spectrometer operated in positive electrospray ionization (ESI) mode. UV-vis absorption spectra were recorded on a Shimadzu UVmini1285 UV-vis spectrophotometer. The steady-state and delayed emission spectra were collected by Hitachi FL-4700 fluorescence spectrometer equipped with chopping systems; the delayed emission spectra were obtained with a delay time of approximately 1 ms. The OLPL spectra at different delay times were recorded in the luminescence mode by Hitachi FL-4700 fluorescence spectrometer. The excited state decay profiles in millisecond to second region were collected by Hitachi FL-4700 fluorescence spectrometer equipped with chopping systems. For solid-state sample, photoluminescence quantum yield was measured by a Hamamatsu absolute PL quantum yield measurement system based on a standard protocol (*Adv. Mater.* **1997**,

9, 230). For solution sample, photoluminescence quantum yield was measured by relative method using appropriate reference. HPLC measurement was performed on a waters ACQUITY UPLC H-Class system by using MeCN/H_2O (volume ratio 95:5) as eluent. The ns-TA spectrum was performed using the EDINBURGH-LP880 transient absorption spectrometer, which uses a 290 nm pump laser and a high-energy xenon lamp as the detection light source, generated by a commercial optical parametric amplifier (TOPAS-PRIME). ESR spectrum was collected on a Bruker EMX plus 6/1 spectrometer equipped with an Oxford Instrument ESR900 liquid He cryostat using an Oxford ITC 503 temperature controller. Photographs and videos were captured by HUAWEI P60 camera and iPhone 12 camera. All animal procedures were reviewed and approved by the Institutional Animal Care and Use Committee at Chinese Academy of Sciences and are in accordance with the Guide for the Care and Use of Laboratory Animals of Chinese Academy of Sciences.

TD-DFT calculations

TD-DFT calculations were performed to study the photophysical properties of molecularly dispersed **BF$_2$bdk** in the solid state. Since the afterglow properties are originated from the excited states of molecularly dispersed **BF$_2$bdk** in the rigid PhB matrices where intermolecular rotation and vibration are largely restricted, the ground-state geometries of **BF$_2$bdk** compounds were used for all the TD-DFT calculations. The ground-state geometries of **BF$_2$bdk** compounds and TMB as well as TMB derivatives were optimized by a DFT calculation using B3LYP functional and 6-311+G(d, p) basis set, while the optimized geometries of their radical cations were further obtained by a DFT calculation using B3LYP functional and 6-311+G(d, p) basis set. The singlet excited states and triplet excited states were calculated on ORCA 4.2.1 program with B3LYP functional and def2-TZVP(-f) basis set and analyzed by Multiwfn software. Spin-orbit coupling (SOC) matrix elements between the singlet excited states and triplet excited states were calculated with spin-orbit mean-field (SOMF) methods on ORCA 4.2.1 program with B3LYP functional and

def2-TZVP(-f) basis set. The obtained electronic structures were analyzed by Multiwfn software. All isosurface maps to show the electron distribution and electronic transitions were rendered by Visual Molecular Dynamics (VMD) software based on the exported files from Multiwfn. Based on the optimized ground-state geometries, the radical-cation geometries of **BF_2bdk** compounds and TMB as well as TMB derivatives were further optimized by a DFT calculation using B3LYP functional and 6-311+G(d, p) basis set, and their electronic and free energies were analyzed by Shermo software. (Neese F, *Wiley Interdiscip. Rev. Comput. Mol. Sci.* **2018**, *8*: 1327-1332; Becke AD, *Phys. Rev. A* **1988**, *38*: 3098-3100; Lee C, Yang W, Parr RG, *Phys. Rev. B.* **1988**, *37*: 785-789; Miehlich B, Savin A, Stoll H, Preuss H, *Chem. Phys. Lett.* **1989**, *157*: 200-206; Roy LE, Hay PJ, Martin RL, *J. Chem. Theory Comput.* **2008**, *4*: 1029–1031; Weigend F, Ahlrichs R, *Phys. Chem. Chem. Phys.* **2005**, *7*: 3297-3305; Lu T, Chen F, *J. Comput. Chem.* **2012**, *33*: 580-592; Humphrey W, Dalke A, Schulten K, *J. Mol. Graphics* **1996**, *14*: 33-38; T. Lu, Q Chen, *Comput. Theor. Chem.* **2021**, *1200*, 113249).

2. Supporting Results and Discussion

Text S1. Ruling out TMB-PhB exciplex OLPL to **BF_2bdk** energy transfer mechanism and other mechanism

To investigate the possibility of exciplex formation between TMB and PhB, TMB-PhB and TMB-COP materials have been prepared by incorporating TMB into PhB and cyclic olefin polymer (COP), respectively (Figure S17). The results show that the UV-vis absorption spectrum of TMB-PhB materials is similar to that of TMB-COP materials, without any additional red-shifted exciplex absorption signals (Figure S17B). Similarly, the steady-state emission spectra of both TMB-PhB and TMB-COP materials are quite comparable, with a slight red shift in the maximum emission signal due to the increased dipole interactions in the PhB matrix (Figure S17C). It is noteworthy that the emission signal around 500 nm in the steady-state emission spectrum of TMB-PhB materials can be attributed to the phosphorescence emission of TMB (Figure S17C). This can be validated through the steady-state emission spectra of both TMB-PhB and TMB-COP materials at 77 K, which display a more pronounced emission signal at 500 nm compared to room temperature (Figure S17D and S17G); this emission signal is also consistent with the phosphorescence emission observed in the delayed spectra (Figure S17C), as well as the previously reported phosphorescence emission of TMB (*Nature* **2017**, *550*, 384). Unlike COP, which allows oxygen to permeate under ambient conditions, the crystalline environment of the PhB matrix can effectively protect the T_1 states of TMB from oxygen quenching. Consequently, the steady-state emission spectrum of TMB-PhB materials exhibit a pronounced phosphorescence emission signal of TMB, whereas the steady-state emission spectrum of TMB-COP materials show only a negligible phosphorescence emission signal at room temperature (Figure S17C). Therefore, the mechanism involving exciplex formation between TMB and PhB leading to OLPL afterglow could be ruled out in this work. Furthermore, various dopant emitters, which have been reported to act as energy acceptors for exciplexes to achieve various colored OLPL afterglows, have also been introduced into TMB-PhB system. However, the resultant TMB-PhB-dopant materials do not exhibit any noticeable OLPL afterglow at

room temperature, especially in comparison to **7**-PhB-TMB materials (Figure S22); this further excludes the mechanism of exciplex-to-**BF_2bdk** energy transfer leading to the OLPL afterglow of **BF_2bdk**-PhB-TMB three-component systems.

On the other hand, recent study reported that multi-resonance (MR)-TADF molecule *v*-DABNA, when excited by visible light, can transfer its excited-state energy to the *m*-MTDATA-3TPYMB donor-acceptor pair, sensitizing the charge separation process and enabling the three-component system to exhibit visible-light-excitable OLPL properties (*Adv. Mater.* **2024**, *36*, 2400158). Notably, the intensity of this OLPL afterglow is comparable to that achieved through ultraviolet excitation of the donor-acceptor pair. However, in present work, TMB-PhB two-component samples show insignificant OLPL afterglow after the removal of UV excitation. Therefore, the aforementioned sensitization mechanism cannot fully account for the observed OLPL phenomena in our system.

Notably, **BF_2bdk** molecules used in this work have been purified several times by recrystallization before use, with its high purity confirmed by HPLC measurements (Figure S3), while TMB and PhB are commercially sourced. In order to rule out the impact of impurities on the OLPL afterglow of the **BF_2bdk**-PhB-TMB materials, we also purified TMB and PhB through recrystallization, and the resultant TMB' and PhB' were confirmed to be of high purity *via* HPLC measurements (Figure S23). It has been found that the steady-state and delayed emission spectra as well as afterglow emission decay curves of **7**-PhB-TMB and **7**-PhB'-TMB' materials are almost identical (Figure S24), indicating that impurities have a negligible effect on the OLPL afterglow of the **7**-PhB-TMB materials.

Text S2. About the electron transfer process from TMB's HOMO to **BF_2bdk**'s HOMO

Notably, the doping concentrations of **BF_2bdk** and TMB in **BF_2bdk**-PhB-TMB materials are as low as 0.2 wt%, which results in a significant spatial distance between these molecules dispersed within the PhB matrix. This raises questions about the feasibility of electron transfer processes between them. To address this, conventional

donor-acceptor pairs (fluorene and tetracyanoethylene (TCNE)) have been selected as a model to investigate the electron transfer process at a low concentration (0.2 wt%) incorporated into the PhB matrix. The results indicate that the color of the fluorene-TCNE-PhB-0.2% three-component materials under daylight is a deeper brown compared to fluorene-PhB-0.2% (white color) and TCNE-PhB-0.2% (white color) two-component materials (Figure S26), which suggests that intermolecular charge transfer can occur effectively even at a doping concentration as low as 0.2 wt%. The above model experiments suggest that the electron transfer process between **BF_2bdk** and TMB in **BF_2bdk**-PhB-TMB materials is indeed feasible, supporting the proposed charge separation mechanism.

Text S3. TADF-type afterglow in **BF_2bdk**-PhB two-component system

To validate TADF-type afterglow mechanism in **BF_2bdk**-PhB two-component system, other possible mechanisms such as excited state energy transfer mechanism, donor-acceptor mechanism, and impurity mechanism have been thoroughly discussed and ruled out.

Room-temperature afterglow can arise from excited state energy transfer from RTP donors to luminescent acceptors (*Nat. Commun*. **2020**, *11*, 4802; *Angew. Chem. Int. Ed*. **2020**, *59*, 9393), while the excited state energy transfer process occurs only when the donors are excited at their absorption maximum or near the maximum. The obtained **BF_2bdk**-PhB afterglow materials in present study can be excited by 365 nm UV, even 405 nm laser and PhB matrices show negligible UV-vis absorption at 365 nm and 405 nm (Figure S2). Therefore, the possibility of room-temperature phosphorescence originating from energy transfer from PhB matrices to **BF_2bdk** can be ruled out.

Recent research has also demonstrated that the triplet excited states of organic matrices can act as mediators for singlet-to-triplet intersystem crossing (ISC) of phosphorescence dopants when their energy levels fall between the S_1 and T_1 states of the dopants, resulting in the emergence of organic room-temperature afterglow (*Angew. Chem. Int. Ed*. **2020**, *59*, 16054). However, this mediation of ISC does not

apply to the $\mathbf{BF_2bdk}$-PhB system in present study, as the T_1 level of the PhB matrices is significantly higher than both the S_1 and T_1 states of $\mathbf{BF_2bdk}$ molecules. Additionally, the high T_1 levels of organic matrices play a key role in preventing afterglow quenching caused by triplet-to-triplet energy transfer from luminescent dopants to organic matrices, thus enabling the construction of high-performance afterglow materials (*Adv. Funct. Mater.* **2013**, *23*, 3386; *Angew. Chem. Int. Ed.* **2021**, *60*, 17138).

Within organic materials, the donor-acceptor mechanism can induce charge-separation states, which have been observed in some organic systems (*Nature* **2017**, *550*, 384). The long persistent luminescence observed in organic systems is attributed to delayed charge recombination in rigid solid matrices. For organic donor-acceptor afterglow systems, the formation of charge-separated states through intermolecular charge transfer between the donor and acceptor molecules is a prerequisite for long persistent luminescent properties. However, in this study, the negligible intermolecular charge transfer between the $\mathbf{BF_2bdk}$ dopant and organic matrices such as PhB, due to the latter's lower HOMO and higher LUMO levels, precludes the possibility of the long persistent luminescence mechanism within this system.

Recent reports suggest that impurities may also be a possible cause of organic room-temperature phosphorescence (*Nat. Mater*. **2021**, *20*, 175). In this study, the $\mathbf{BF_2bdk}$ compounds and TMB have been carefully purified using column chromatography and recrystallized three times in spectroscopic grade dichloromethane/n-hexane to ensure its purity. Additionally, HPLC measurements confirm their high purity (Figure S3). The resulting high-purity $\mathbf{BF_2bdk}$ powders exhibit no afterglow properties at room temperature, but the $\mathbf{BF_2bdk}$-PhB afterglow materials obtained by doping $\mathbf{BF_2bdk}$ into PhB matrices display remarkable afterglow properties under ambient conditions. The experimental and analytical results presented here exclude the possibility that room-temperature afterglow properties of $\mathbf{BF_2bdk}$-PhB-0.2% materials is due to impurities.

Additionally, low-temperature experiments have been further conducted to explore the excited state properties (Figure S32). For instance, the emission maximum

of **7**-PhB materials at 77 K in its steady-state emission spectrum is nearly identical to that at room temperature (Figure S28 and S32), with the maximum emission peak at 470 nm, allowing us to estimate the S_1 level of compound **7** at 2.638 eV; whereas their delayed emission spectrum at 77 K shows a significant red shift compared to that at room temperature (Figure S29B), with the maximum phosphorescence emission peak at 506 nm, from which the T_1 level and ΔE_{ST} of compound **7** can be estimated to be 2.450 eV and 0.188 eV, respectively. Such a low ΔE_{ST} can significantly facilitate the RISC process, allowing for the simultaneous capture of both singlet and triplet excitons, thereby leading to TADF-type afterglow emission. Besides, the afterglow decay curve monitored at different emission wavelengths of **7**-PhB materials at room temperature have been collected (Figure S30), from which it can be seen that the effect of phosphorescence emission on TADF-type afterglow is negligible, and the accurate TADF-type afterglow lifetime can be fitted into exponential decay of 254 ms (Figure S30), leading to the estimation of k_{RISC} to be in the range of 10^0-10^1 s^{-1}; since the RISC process is the rate-determining step in TADF afterglow emission, and k_{nr} and k_q are greatly suppressed by the crystalline PhB matrix. Therefore, compound **7** exhibits TADF afterglow properties in PhB matrix, which is also supported by theoretical calculations. TD-DFT calculations indicate that compound **7** possesses multiple T_n excited states with different symmetries from the S_1 state (Figure S12), which according to the EI-Sayed rule favor ISC or RISC processes. Moreover, the spin-orbit coupling matrix elements (SOCMEs) between these T_n states and S_1 are relatively large, such as 0.26 cm^{-1} for S_1-T_1, 0.27 cm^{-1} for S_1-T_2, and 0.63 cm^{-1} for S_1-T_3; such large SOCME values can facilitate multiple ISC or RISC channels between T_n and S_1. The presence of multiple T_n-S_1 channels with large SOCMEs, along with a moderate ΔE_{ST}, are the key reasons why compound **7** exhibits TADF-type afterglow with moderate k_{RISC} in the PhB matrix. Similar to **7**-PhB materials, other **BF$_2$bdk**-PhB materials also exhibit TADF afterglow at room temperature (Figure S28). It should be noted that some **BF$_2$bdk**-PhB materials, such as **1**-PhB and **2**-PhB materials, possess TADF/RTP dual emission characteristics; their RTP emission signals at room temperature align with the phosphorescence emission signals at 77 K (Figure S28 and

S32), originating from the T_1 emission of **BF_2bdk**.

Regarding whether the TADF emission has a few hundred milliseconds lifetime, we collect emission decay curves of **BF_2bdk**-PhB-0.2% samples at different wavelengths (Figure S30). It has been found that the emission lifetimes monitored at higher-energy region are slightly shorter than or similar to those recorded at lower-energy region (Figure S30). In particular, the emission decay curves monitored at 430 nm, where phosphorescence is absent as can be seen from the 77 K delayed emission spectra, show long emission lifetime of a few hundred milliseconds (Figure S32), which strongly support that the TADF has lifetime > 100 ms in the present study. We understand that most of the reported TADF emitters have delayed fluorescence lifetimes of 10^{-3} ~ 10^{-6} s. Here the assignment of TADF-type organic afterglow is supported by a series of experimental observations and analyses. (1) The similar emission color of **BF_2bdk**-PhB sample at room temperature under UV excitation (fluorescence color) and after ceasing UV light (afterglow color), as well as the almost identical steady-state and delayed emission spectra of **BF_2bdk**-PhB sample at room temperature (for example, in the case of **7**-PhB). (2) The absence of triplet-to-singlet excited state energy transfer, the absence of donor-acceptor afterglow mechanism based on intermolecular charge transfer and retarded charge recombination, and the absence of afterglow mechanism caused by impurity. (3) The disappearance of delayed fluorescence band at 77 K and the emergence and increase of delayed fluorescence band upon temperature increase. (4) The insignificant contribution of triplet-triplet annihilation at low doping concentration. (5) The long emission lifetime (> 100 ms) obtained by emission decay profiles at higher-energy region where phosphorescence signals are absent. On the other hand, it is noteworthy that with the efforts of our group in this specific research direction of TADF-type organic afterglow (lifetimes > 100 ms), many TADF-type afterglow samples have been obtained (*Angew. Chem. Int. Ed.* **2021**, *60*, 17138; *Adv. Funct. Mater.* **2021**, 2110207; *Chem. Eng. J.* **2022**, *431*, 134197; *J. Mater. Chem. C,* **2022**, *10*, 4795; *Sci. China Chem.* **2023**, *66*, 1120; *J. Mater. Chem. C,* **2023**, *11*, 2291; *Chem. Eur. J.* **2023**, *29*, e202203670; *Adv. Funct. Mater.* **2023**, 2214960; *Chem. Eng. J.* **2023**, *460*, 141916;

Chem. Sci. **2023**, *14*, 8180; *Chem. Mater.* **2024**, *36*, 3000). There are also reported studies on TADF afterglow by other research groups (*Angew. Chem. Int. Ed.* **2023**, *62*, e202217616; *Angew. Chem. Int. Ed.* **2023**, *62*, e202304020). TADF-type organic afterglow systems feature moderate k_{RISC} of 10^0-10^1 s^{-1}. Given that many organic compounds have phosphorescence rate around 10^0 s^{-1} or smaller, the moderate k_{RISC} is enough to open TADF afterglow (lifetimes around 100 ms or longer) in organic systems where nonradiative decay (k_{nr}) and oxygen quenching (k_q) of triplet excited states are sufficiently suppressed by rigid organic matrices. TADF-type afterglow systems with emission lifetimes > 100 ms do exist.

Text S4. About the TADF-type afterglow of **10**-PhB materials

The steady-state emission spectrum of **10**-PhB materials almost overlaps with its delayed emission spectrum, showing a maximum emission peak at 494 nm with afterglow lifetime of 203 ms (Figure S38). This overlapping spectral behavior, similar to other **BF_2bdk**-PhB systems, can be attributed to TADF-type afterglow from S_1 state of compound **10**. In addition, the low-temperature and temperature-dependent delayed emission spectra of **10**-PhB materials reveal that the maximum emission peak at 494 nm decreases as the temperature decreases (Figure S39), consistent with the characteristic of TADF emitters. Conversely, the red-shifted emission peak at 542 nm increases with decreasing temperature and becomes dominant at 77 K, exhibiting a long afterglow lifetime of 1072 ms (Figure S39); this red-shifted emission can be attributed to the phosphorescence emission from T_1 state of compound **10**. Therefore, the resultant **10**-PhB two-component materials exhibits highly-efficiency TADF-type afterglow under ambient conditions, but with a short afterglow duration lasting only for a few seconds.

Text S5. About the methods for calculating or estimating OLPL efficiency

For estimating OLPL efficiency, we perform literature survey. For OLPL materials that are sensitive to air (*Nature* **2017**, *550*, 384), the PLQY of the materials in nitrogen and in air can be measured. The difference in PLQY between materials in

nitrogen and in air can be roughly considered as the efficiency of triplet and charge-separation-related component of emission (*Nature* **2017**, *550*, 384). In several other reported studies, OLPL afterglow efficiency has been rarely discussed (*Nat. Commun.* **2020**, *11*, 191; *Nat. Mater.* **2022**, *21*, 338). In some reported studies especially in two-photon ionization system (*Adv. Mater.* **2020**, *32*, 2003911), the relationship between OLPL afterglow brightness and excitation light intensity is not linear. In polymer-based OLPL systems and n-type OLPL systems, the OLPL afterglow intensity would be also dependent on excitation time (because of the presence of oxygen and other factors). Besides, OLPL materials usually show minutes-long or hour-long duration, so that the PLQY values obtained using integrating sphere in absolute PLQY measurement systems don't contain the contribution of OLPL afterglow. As described in *Nat. Commun.* **2020**, *11*, 191, "the quantum efficiency of LPL emission is difficult to define, because the charge accumulation and release processes are slow and complicated in contrast to those of long-lived phosphorescence". In the present study, the property of **10**-PhB-TMB materials is relatively simple. Its OLPL property can be obtained by short-term and low-power 365 nm UV excitation; the crystalline PhB matrix can well protect organic triplets and radical intermediates under ambient conditions. The **10**-PhB-TMB materials can be readily excited by 365 nm UV lights of Hitachi FL-4700 fluorescence spectrometer to exhibit emission decay profile with long durations (Figure 4 in the main text); because of its strong UVA absorption capability, compound **10** in the materials can be sufficiently excited by 365 nm UV lights to initiate the subsequently photophysical processes in the **10**-PhB-TMB afterglow system. Given the fact that the present **10**-PhB-TMB afterglow materials are relatively simple that can be readily excited by UVA lights, we propose the calculation or estimation of OLPL afterglow efficiency in the present **10**-PhB-TMB system. From the emission decay profile in Figure S40, the percentages of the first part (0-2 s, prompt fluorescence and TADF afterglow) and the second part (2-500 s, OLPL afterglow) can be calculated to be 82.2% and 17.8%, respectively; the reported study in *Adv. Mater.* **2024**, 2312439 also use decay profile to obtain percentage of each component. The PLQY of **10**-PhB-TMB materials obtained by a Hamamatsu

absolute PLQY measurement system is 50.7%, which can be approximately refer to emission from the first part (0-2 s) of the decay profile (Figure S40); because of its hour-long duration, the PLQY values obtained from the absolute PLQY measurement systems don't contain the contribution of OLPL afterglow. Based on these, the OLPL afterglow efficiency can be roughly estimated to be 11.0% (50.7% × 17.8% / 82.2% = 11.0%). Here we fully consider the specific property of the present **10**-PhB-TMB afterglow system and propose this method to calculate or estimate OLPL afterglow efficiency.

Text S6. The selection guideline of organic matrix.

In the early stage of the studies on **BF_2bdk**-matrix systems, we prepare high-performance afterglow materials by screening **BF_2bdk** compounds and organic matrices. During the studies in recent two years, we do accumulate some knowledge on the selection guideline of the organic matrices in our **BF_2bdk**-matrix systems. (1) Organic matrices with relatively large dipole moments can interact with **BF_2bdk** excited states via dipole-dipole interactions, reduce S_1 levels of **BF_2bdk** (T_1 levels of **BF_2bdk** is relatively insensitive to medium or environment), and thus decrease ΔE_{ST} values and enhance both ISC and RISC. Because of their relatively large dipole moments, benzophenone derivatives can be used as suitable organic matrices for the fabrication of **BF_2bdk**-matrix afterglow materials in some circumstance. (2) To suppress afterglow quenching caused by excited state energy transfer from **BF_2bdk** triplets to organic matrices, organic matrices with higher T_1 levels than **BF_2bdk** is necessary for the fabrication of high-performance afterglow materials in our **BF_2bdk**-matrix systems. In the case of **BF_2bdk** with low T_1 levels, it is found that benzophenone derivatives such as BP and MeOBP can be suitable organic matrices. When the **BF_2bdk** dopants possess high T_1 levels, phenyl benzoate (PhB) can be suitable matrices due to their higher T_1 levels (benzophenone derivatives is not suitable as reported in *Angew. Chem. Int. Ed.* **2021**, *60*, 17138 and *Chem. Commun.* **2021**, *57*, 8794). (3) It is also found that organic matrices such as phenyl benzoate and benzophenone derivatives can protect **BF_2bdk** triplets from oxygen quenching and

largely suppress nonradiative decay of **BF_2bdk** triplets, especially in the melt-cast dopant-matrix afterglow materials (*Adv. Opt. Mater*. **2021**, 2100353; *Chem. Commun.* **2021**, *57*, 8794). Based on the above knowledge, it is understandable that, since the **BF_2bdk** in the present study possess relatively high T_1 levels, PhB matrices, rather than benzophenone derivatives, are selected for the fabrication of afterglow materials. These, together with PhB's property of being transparent in UVA region and being able to accept electrons to form radical anions, are the reasons of matrix selection in this study.

3. Figures and Tables

Components	Mechanism	Performance index	References
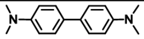<br>**Donor**<br>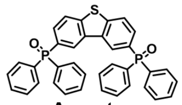<br>**Acceptor**	D-A mechanism	• 526 nm, **in N_2**<br>• PLQY = 21%(7% in air), Φ_{OLPL} = 14%<br>• Decay time > 5000 s<br>• Duration time > 1 h	*Nature* **2017**, *550*, 384.
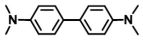<br>**Donor**<br>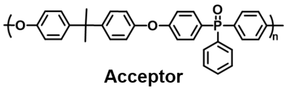<br>**Acceptor**	D-A mechanism	• 526 nm, **in N_2**<br>• PLQY = 3%<br>• Decay time ~ 300 s<br>• Duration time > 7 min	*Adv. Mater.* **2018**, *30*, 1803713.
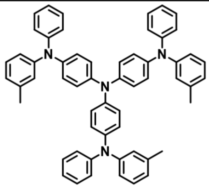<br>**Donor**<br>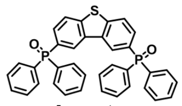<br>**Acceptor**	D-A mechanism	• ~520 nm, **in N_2**<br>• PLQY = 31%<br>• Decay time > 1000 s<br>• Duration time = -	*Chem. Lett.* **2019**, *48*, 270.
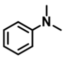 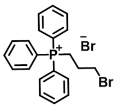<br>**Donor** **Acceptor**	D-A mechanism	• 500 nm, **under ambient conditions**<br>• PLQY = -<br>• Decay time = 7 h<br>• Duration time = 7 h	*Adv. Mater.* **2020**, *32*, 2001026.
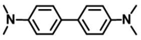<br>**Donor 1**<br>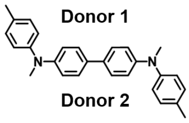<br>**Donor 2**<br>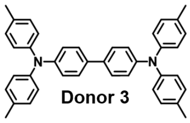<br>**Donor 3**<br>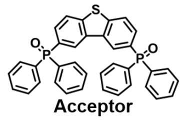<br>**Acceptor**	D-A mechanism	**D1+A:**<br>• 530 nm, **in N_2**<br>• PLQY = 24%<br>• Decay time ~ 1000 s<br>**D2+A:**<br>• ~540 nm, **in N_2**<br>• PLQY = 27%<br>• Decay time ~ 800 s<br>**D3+A:**<br>• ~530 nm, **in N_2**<br>• PLQY = 28%<br>• Decay time ~ 400 s	*Nat. Commun.* **2020**, *11*, 191.
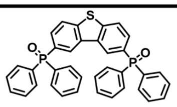<br>**Host 1**<br>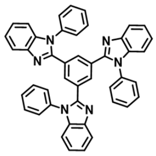 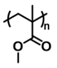<br>**Host 2** **Host 3**<br>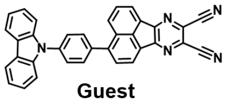<br>**Guest**	TPI mechanism	**H1+G:**<br>• 586 nm, **in N_2**<br>• PLQY = 49.9%, in air<br>• Decay time ~ 4500 s<br>• Duration time > 1000 s<br>**H2+G:**<br>• 578 nm, **in N_2**<br>• PLQY = 46.2%, in air<br>• Decay time ~ 1500 s<br>• Duration time > 1000 s<br>**H3+G:**<br>• 560nm, **in N_2**<br>• PLQY = 29.1%, in air<br>• Decay time = ~400 s<br>• Duration time > 400 s	*Adv. Mater.* **2020**, *32*, 2003911.

Components	Mechanism	Performance index	References
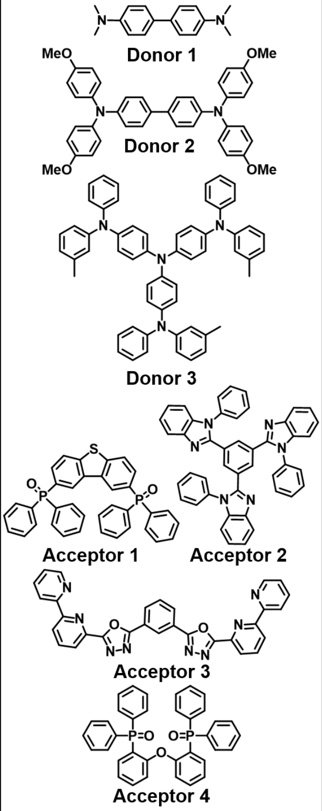	D-A mechanism	**D1+A1:**<br>• 527 nm, **in N_2**<br>• PLQY = 6.5%<br>• Decay time = 868 s<br>**D2+A1:**<br>• 524 nm, **in N_2**<br>• PLQY = 9.3%<br>• Decay time = 1073 s<br>**D3+A1:**<br>• 523 nm, **in N_2**<br>• PLQY = 35.2%<br>• Decay time = 1352 s<br>**D3+A2:**<br>• 557 nm, **in N_2**<br>• PLQY = 16.6%<br>• Decay time = 110 s<br>**D3+A3:**<br>• 587 nm, **in N_2**<br>• PLQY = 10.9%<br>• Decay time = 47 s<br>**D3+A4:**<br>• 471 nm, **in N_2**<br>• PLQY = 7.6%<br>• Decay time = 229 s	*Adv. Funct. Mater.* **2020**, *30*, 2000795.
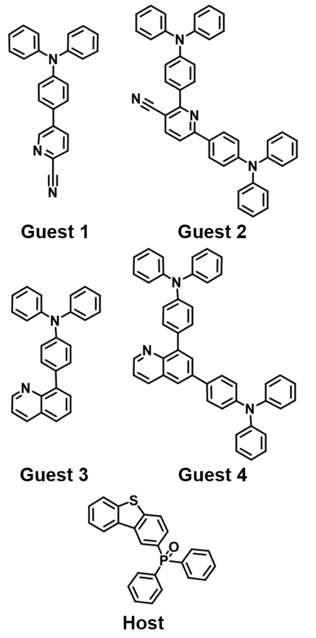	TPI mechanism	**G1+H:**<br>• 532 nm<br>• PLQY = -<br>• Decay time ~ 2400 s<br>• Duration time ＞ 1200 s<br>**G2+H:**<br>• 538 nm<br>• PLQY = -<br>• Decay time ~ 2500 s<br>• Duration time ＞ 2400 s<br>**G3+H:**<br>• 550 nm<br>• PLQY = -<br>• Decay time ~ 3000 s<br>• Duration time ＞ 2400 s<br>**G4+H:**<br>• 577 nm<br>• PLQY = -<br>• Decay time ~ 2300 s<br>• Duration time ＞ 1200 s	*Angew. Chem. Int. Ed.* **2021**, *60*, 16984.
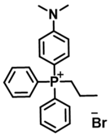	TPI mechanism	• 450 nm, **in air**<br>• PLQY = 3.3%<br>• Decay time ~ 20 min<br>• Duration time ~ 12 min	*J. Am. Chem. Soc.* **2022**, *144*, 3050.

Components	Mechanism	Performance index	References
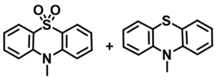	/	• ~497 nm, **in air**<br>• $\Phi_{phos.}$ = 20%<br>• Decay time ~ 1600 s<br>• Duration time > 25 min	*Adv. Mater.* **2021**, *33,* 2007811.
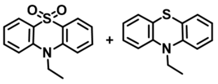		• ~503 nm, **in air**<br>• $\Phi_{phos.}$ = 43%<br>• Decay time ~ 1400 s<br>• Duration time > 25 min	
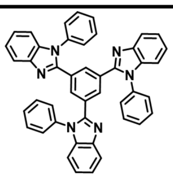<br>**Donor 1**<br>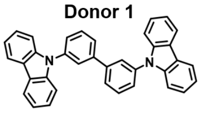<br>**Donor 2**<br>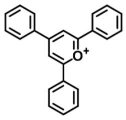<br>**Acceptor 1**<br>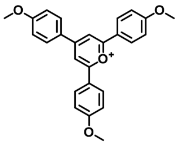<br>**Acceptor 2**<br>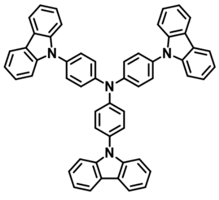<br>**Trap 1**<br>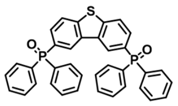<br>**Trap 2**<br>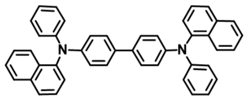<br>**Trap 3**	D-A mechanism	**D1+A1:**<br>• 603 nm, **in N_2**<br>• PLQY = 7%<br>• Decay time = 1830 s<br>• Decay time in air = 160 s<br>**D2+A1:**<br>• 731 nm, **in N_2**<br>• PLQY = 1%<br>• Decay time = 33 s<br>• Decay time in air = -<br>**D1+A2:**<br>• - nm, **in N_2**<br>• PLQY = 1%<br>• Decay time = -<br>• Decay time in air = -<br>**D2+A2:**<br>• 624 nm, **in N_2**<br>• PLQY = 7%<br>• Decay time = 610 s<br>• Decay time in air = -<br>**D1+A1+T1:**<br>• 601 nm, **in N_2**<br>• PLQY = 2%<br>• Decay time = 14550 s<br>• Decay time in air = 1685 s<br>**D1+A1+T2:**<br>• 597 nm, **in N_2**<br>• PLQY = 9%<br>• Decay time = 850 s<br>• Decay time in air = 415 s<br>**D1+A1+D2:**<br>• 600 nm, **in N_2**<br>• PLQY = 8%<br>• Decay time = 970 s<br>• Decay time in air = 285 s<br>**D1+A1+T3:**<br>• 601 nm, **in N_2**<br>• PLQY = 1%<br>• Decay time = 12640 s<br>• Decay time in air = 1060 s	*Nat. Mater.* **2022**, *21,* 338.
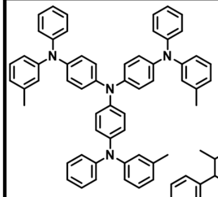<br>**Donor**<br>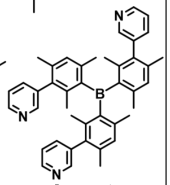<br>**Acceptor**	D-A mechanism	• 525 nm, **in N_2**<br>• PLQY = -<br>• Decay time ~ 120 s<br>• Duration time > 100 s	*Small Struct.* **2023**, *4*, 2300052.

Components	Mechanism	Performance index	References
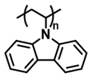 **Donor**<br>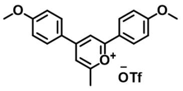 **Acceptor 1**<br>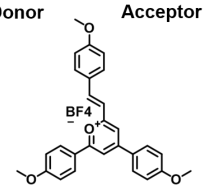 **Acceptor 2**<br>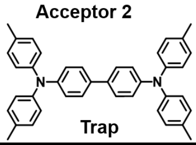 **Trap**	D-A mechanism	**D+A1:**<br>• 606 nm, **under ambient conditions**<br>• PLQY = -<br>• Decay time ~ 5000 s<br>**D+A2:**<br>• 650 nm, **under ambient conditions**<br>• PLQY = -<br>• Decay time ~ 3000 s<br>**D+A1+T:**<br>• 606 nm, **under ambient conditions**<br>• PLQY = -<br>• Decay time > 10000 s	*Chem. Sci.* **2023**, *14*, 8180.
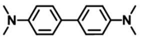 **Donor 1**<br>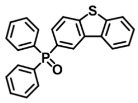 **Acceptor 1**<br>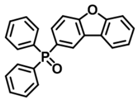 **Acceptor 2**<br>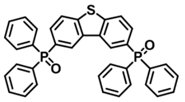 **Acceptor 3**<br>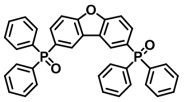 **Acceptor 4**	D-A mechanism	**D+A1:**<br>• 512 nm, **in N_2**<br>• PLQY = 1.3%<br>• Decay time ~ 8000 s<br>• Duration time = 5163 s<br>• Decay time under high-power excitation > 180 min<br>• Duration time under high-power excitation = 120 min<br>**D+A2:**<br>• - nm, **in N_2**<br>• PLQY = 6.3%<br>• Decay time ~ 10 s<br>• Duration time = 24 s<br>• Decay time under high-power excitation = -<br>• Duration time under high-power excitation = 30 min<br>**D+A3:**<br>• - nm, **in N_2**<br>• PLQY = 2.9%<br>• Decay time ~ 2500 s<br>• Duration time = 2017 s<br>• Decay time under high-power excitation = -<br>• Duration time under high-power excitation = 5 min<br>**D+A4:**<br>• - nm, **in N_2**<br>• PLQY = 4.2%<br>• Decay time ~ 100 s<br>• Duration time = 288 s<br>• Decay time under high-power excitation = -<br>• Duration time under high-power excitation = 1 min	*Sci. China. Mater.* **2023**, *66*, 4756.
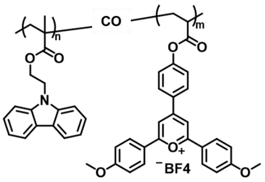 CP1: m/n = 0.003 CP2: m/n = 0.005<br>CP3: m/n = 0.012 CP4: m/n = 0.090	D-A mechanism	**CP1:**<br>• 690 nm, **in N_2**<br>• PLQY = 3.5%<br>• Decay time ~ 4000 s<br>• Decay time in air < 10 min<br>**CP2:**<br>• 698 nm, **in N_2**<br>• PLQY = 3.1%<br>• Decay time ~ 4000 s (~ 400 s in air)<br>• Decay time in air < 10 min	*Angew.Chem. Int.Ed.* **2024**, *63*,e202314500.

Components	Mechanism	Performance index	References
		**CP3:**<br>• 714 nm, **in N_2**<br>• PLQY = 1.3%<br>• Decay time ~ 1000 s<br>• Decay time in air < 10 min<br>**CP4:**<br>• 732 nm, **in N_2**<br>• PLQY = 0.7%<br>• Decay time ~ 10 s<br>• Decay time in air < 10 min	
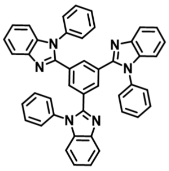<br>**Host 1**<br>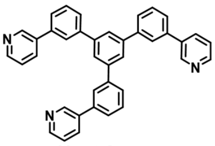<br>**Host 2**<br>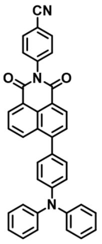<br>**Guest 1**<br>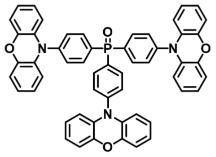<br>**Guest 2**<br>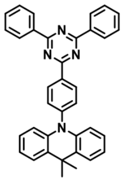<br>**Guest 3**<br>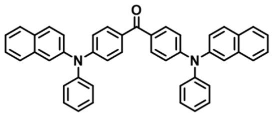<br>**Guest 4**<br>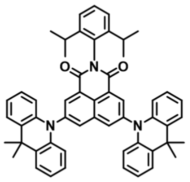<br>**Guest 5**<br>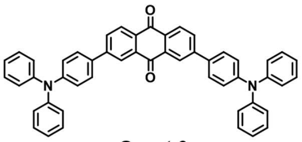<br>**Guest 6**	/	**H1+G1:**<br>• 585 nm, **in N_2**<br>• PLQY = -<br>• Decay time ~ 100000 s<br>• Decay time > 24 h<br>**H1+G2:**<br>• 507 nm, **in N_2**<br>• PLQY = -<br>• Decay time ~ 4000 s<br>• Decay time = 1.2 h<br>**H1+G3:**<br>• 530 nm, **in N_2**<br>• PLQY = -<br>• Decay time ~ 5000 s<br>• Decay time = 2.1 h<br>**H1+G4:**<br>• 517/552 nm, **in N_2**<br>• PLQY = -<br>• Decay time ~ 3500 s<br>• Decay time = 1.06 h<br>**H1+G5:**<br>• 640 nm, **in N_2**<br>• PLQY = -<br>• Decay time > 40000 s<br>• Decay time > 12 h<br>**H1+G6:**<br>• 669 nm, **in N_2**<br>• PLQY = -<br>• Decay time ~ 2000 s<br>• Decay time = 0.86 h<br>**H2+G1:**<br>• 594 nm, **in N_2**<br>• PLQY = -<br>• Decay time ~ 4500 s<br>• Decay time = 1.6 h	*Nat.Photonics.* **2 024,** *18*, 350-356.

Components	Mechanism	Performance index	References
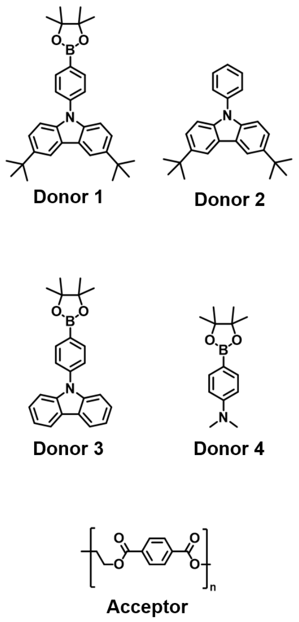	D-A mechanism	**D1+A:**<br>• 500 nm, **under ambient conditions**<br>• Φ_{OLPL} = 21.3%<br>• Decay time ~ 10000 s<br>• Duration time = 11 h<br>**D2+A:**<br>• 500 nm, **under ambient conditions**<br>• Φ_{OLPL} = -<br>• Decay time ~ 5000 s<br>• Duration time = 5000 s<br>**D3+A:**<br>• 488 nm, **under ambient conditions**<br>• Φ_{OLPL} = -<br>• Decay time ~ 3000 s<br>• Duration time = 3000 s<br>**D4+A:**<br>• 500 nm, **under ambient conditions**<br>• Φ_{OLPL} = -<br>• Decay time ~ 200 s<br>• Duration time = 200 s	*Adv. Mater.* **2024**, *36*, 2312439.
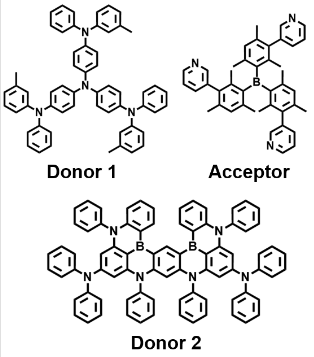	D-A mechanism	**D1+D2+A:**<br>• 550 nm<br>• Duration time = 15452 s	*Adv. Mater.* **2024**, *36*, 2400158.

Figure S1. Examples of afterglow materials with OLPL property (D-A mechanism: OLPL system based on donor and acceptor pairs; TPI mechanism: OLPL system based on two-photon ionization; decay time: afterglow duration obtained from emission decay profiles; duration time: afterglow duration obtained by cameras or observed by human eyes).

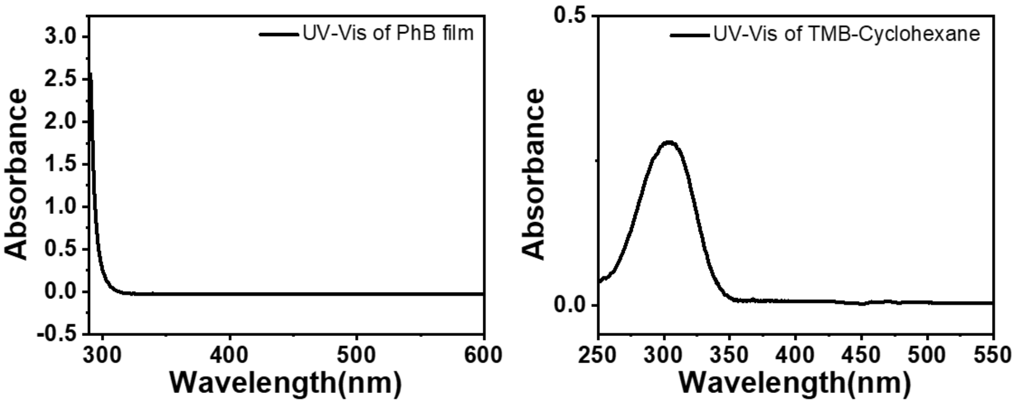

Figure S2. UV-vis absorption spectra of PhB matrix and TMB. Both PhB matrix and TMB show negligible UV-vis absorption at 365 nm and 405 nm.

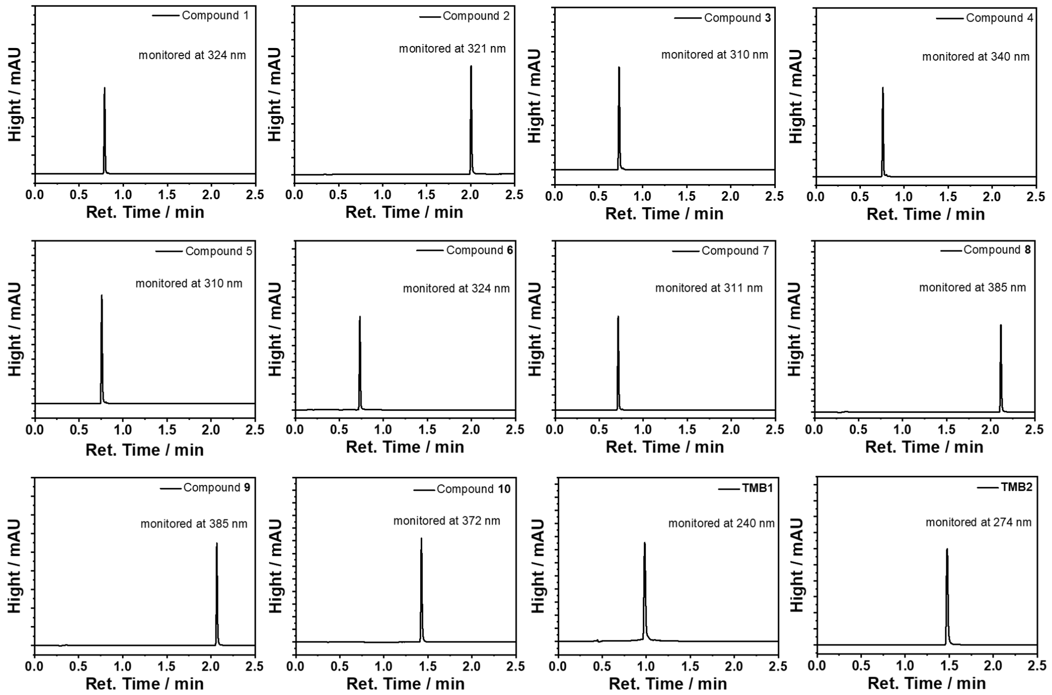

Figure S3. HPLC profile of compounds **1~10**, **TMB1, TMB2**.

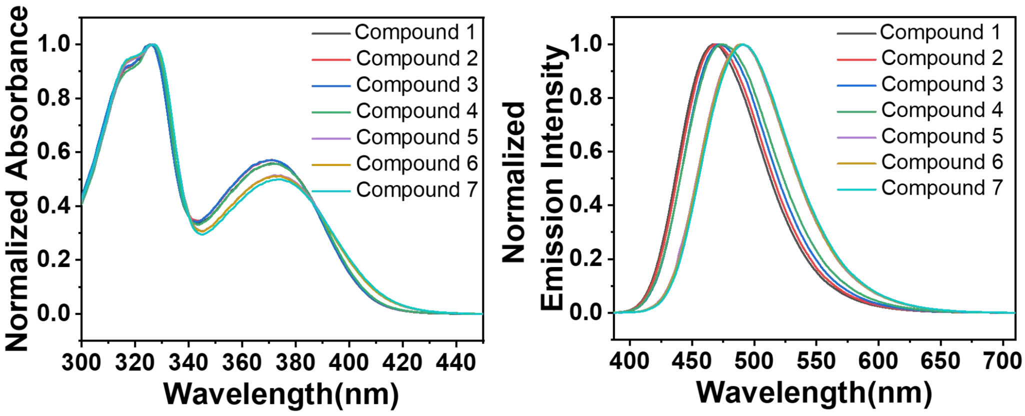

Figure S4. UV-vis spectra of compounds **1-7** in DCM and their steady-state emission spectra.

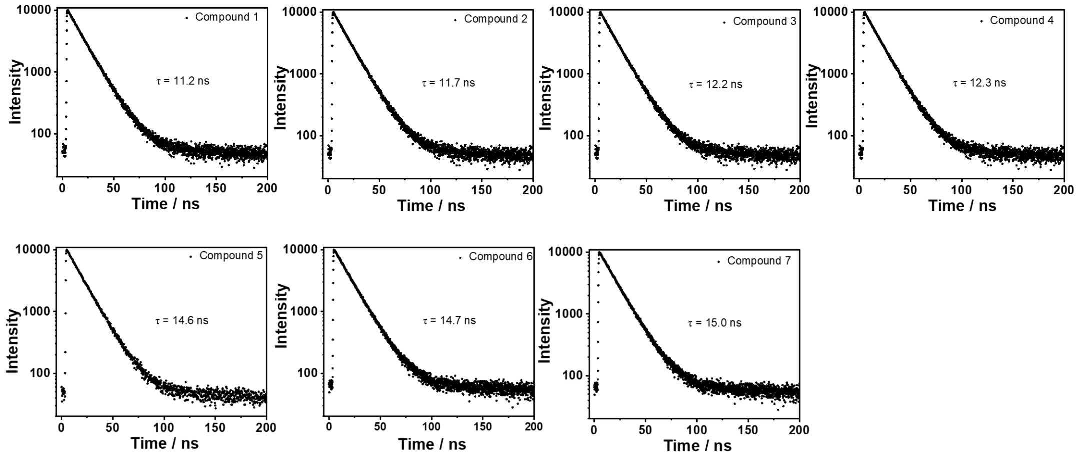

Figure S5. The fluorescence decay profiles of compounds **1-7** in dichloromethane solution excited at 365 nm.

Table S1. Photophysical data of compounds **1-7** in DCM at room temperature.

Compounds	λ_{abs} / nm	ε / (10^4 L mol^{-1} cm^{-1})	λ_F / nm	τ_F / ns	Φ / %
**1**	316.5<br>326<br>371	1.04<br>1.14<br>0.64	467.2	11.2	56.90
**2**	317<br>325.5<br>371	1.25<br>1.37<br>0.78	469.0	11.7	57.67
**3**	317<br>325.5<br>371.5	1.40<br>1.52<br>0.87	472.8	12.2	55.00
**4**	317<br>327<br>372	1.24<br>1.37<br>0.76	473.6	12.3	54.70
**5**	317<br>327<br>374.5	1.35<br>1.45<br>0.74	489.6	14.6	52.52
**6**	317<br>327<br>374	1.09<br>1.16<br>0.59	489.4	14.7	50.45
**7**	317<br>326.5<br>374	1.37<br>1.45<br>0.72	491.0	15.0	52.20

Table S2. Biphenyl dihedral angles of the ground states of compounds **1-7** obtained through TD-DFT calculations and single crystal analysis

**Dihedral angle (°)**	**1**	**2**	**3**	**4**	**5**	**6**	**7**
DFT ground state	89.9	89.0	87.6	88.1	61.7	60.5	57.3
single crystal	/	86.5	80.5	84.0	56.4	/	40.8

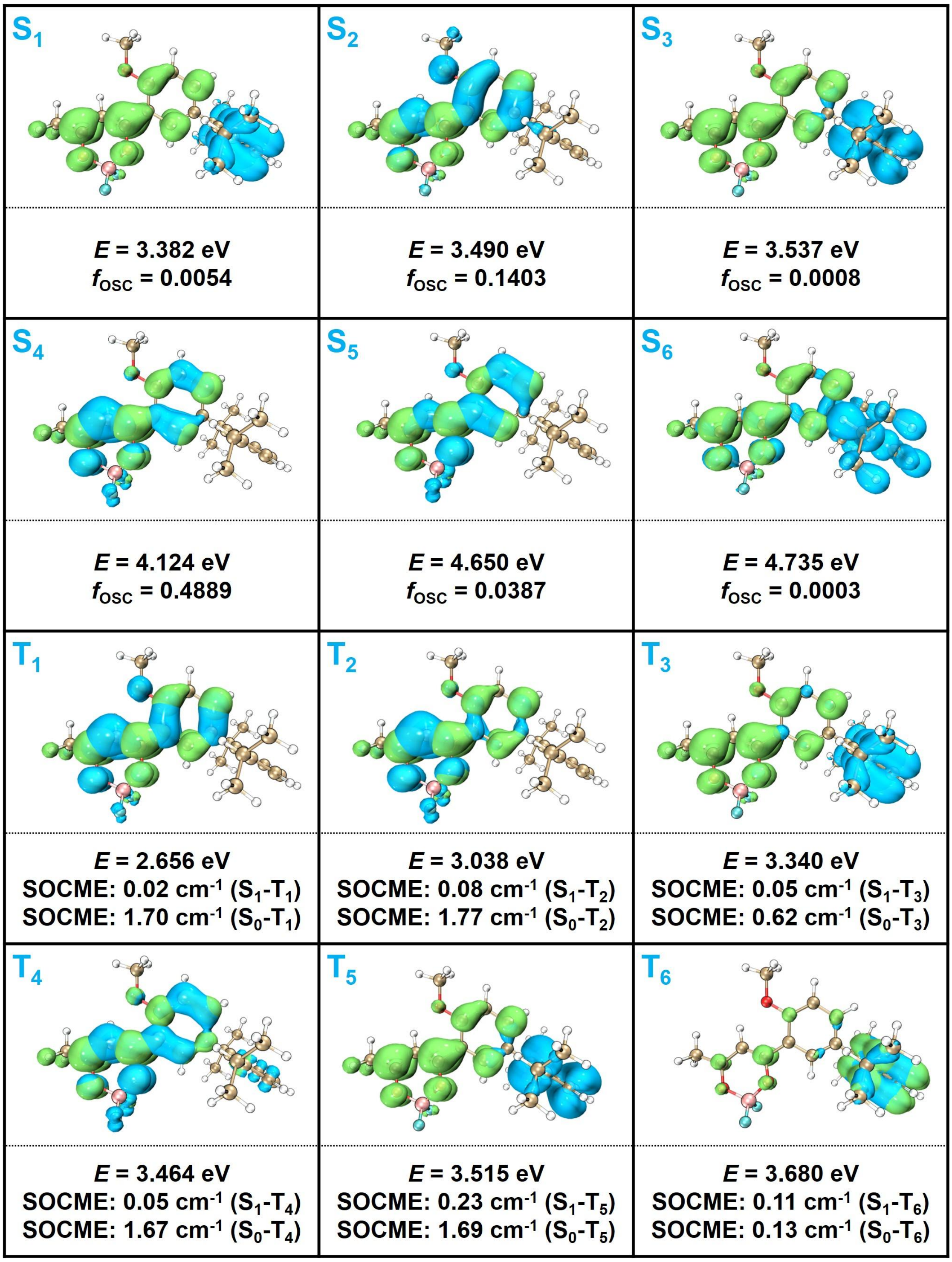

Figure S6. Iso-surface maps of electron-hole density difference of compound **1**'s excited states calculated at B3LYP/def2-TZVP(-f) level, where blue and green iso-surfaces correspond to hole and electron distributions, and excitation energies, oscillator strengths and spin–orbit coupling matrix element (SOCME) values.

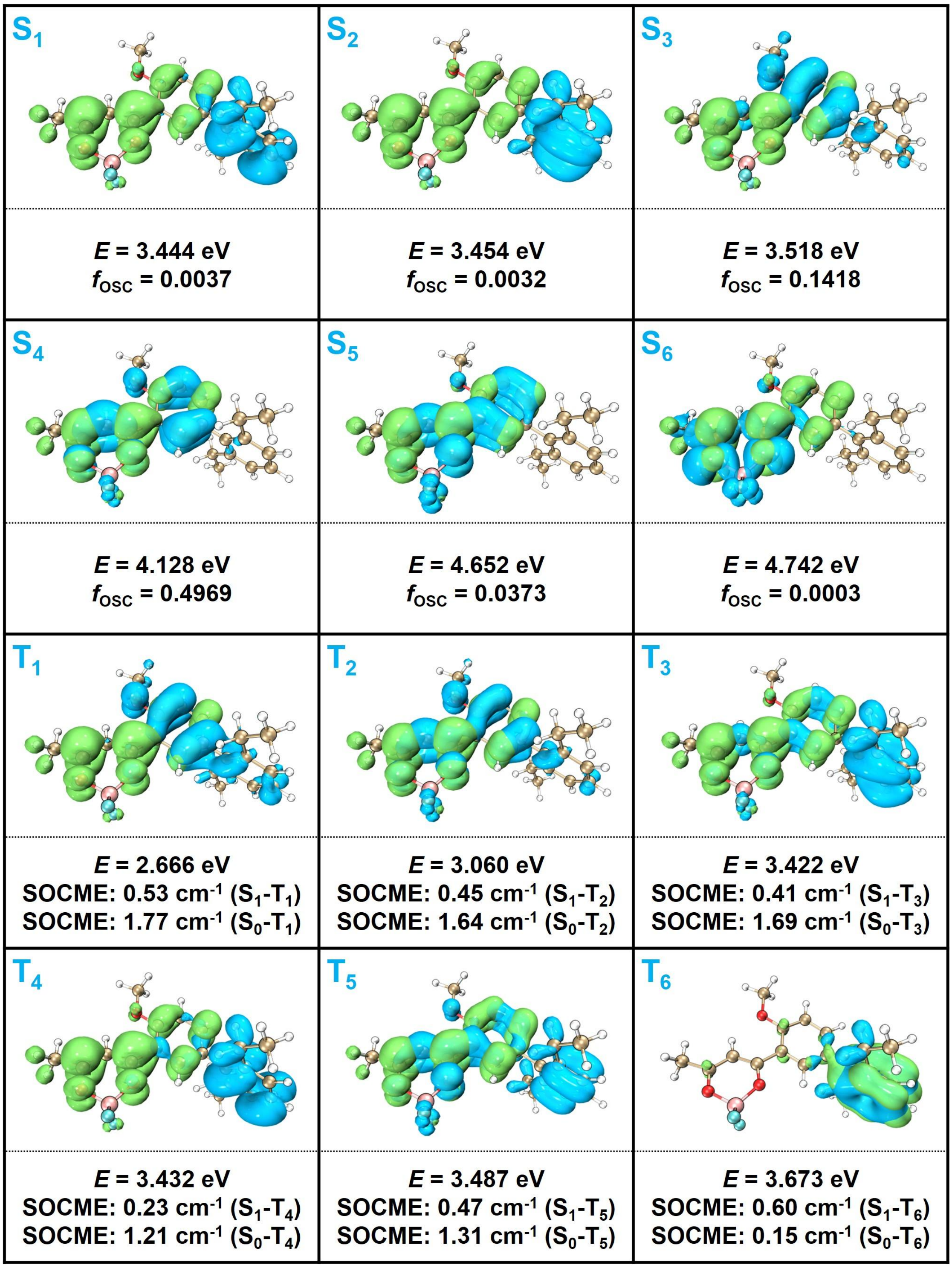

Figure S7. Iso-surface maps of electron-hole density difference of compound **2**'s excited states calculated at B3LYP/def2-TZVP(-f) level, where blue and green iso-surfaces correspond to hole and electron distributions, and excitation energies, oscillator strengths and spin–orbit coupling matrix element (SOCME) values.

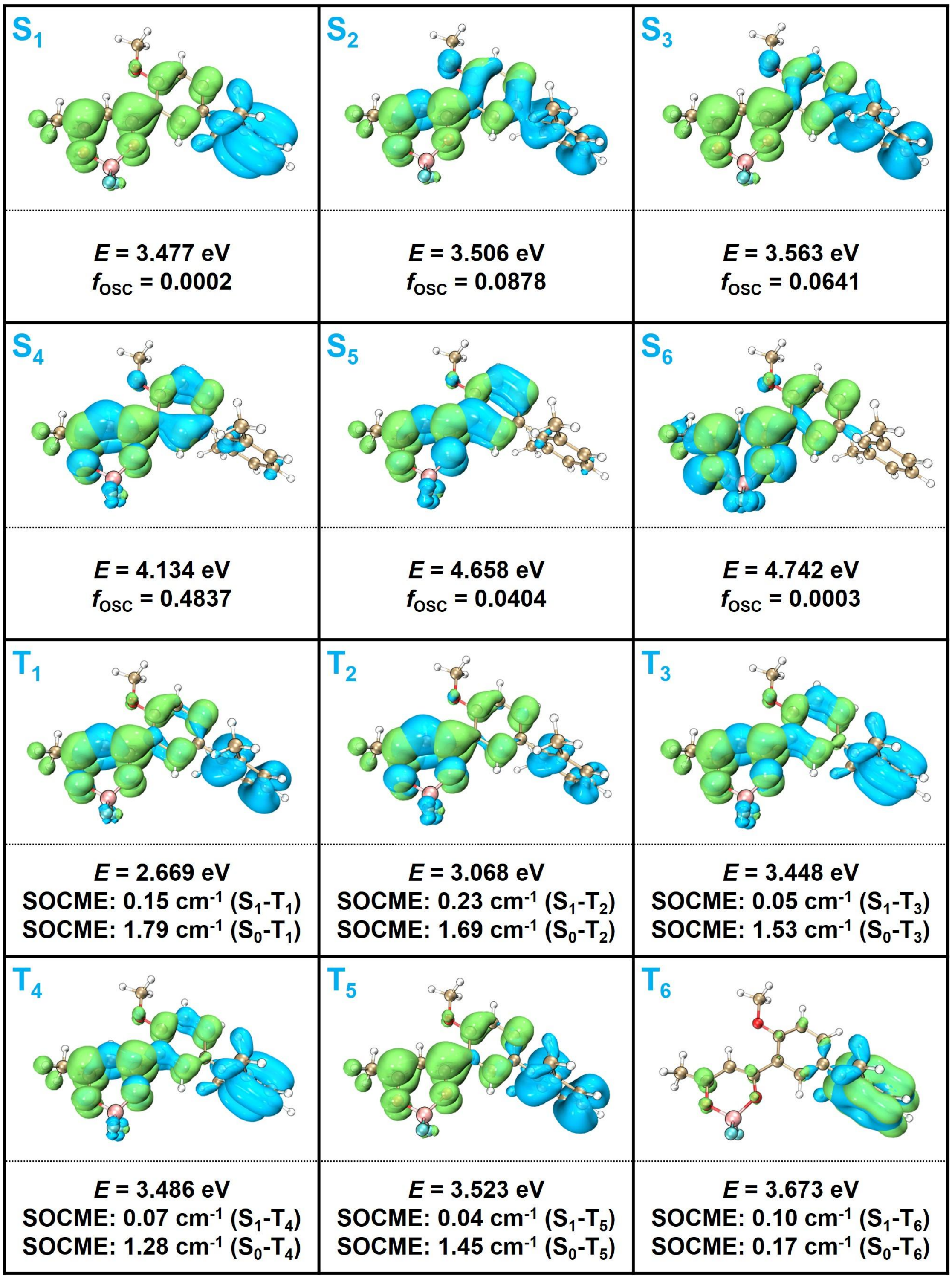

Figure S8. Iso-surface maps of electron-hole density difference of compound **3**'s excited states calculated at B3LYP/def2-TZVP(-f) level, where blue and green iso-surfaces correspond to hole and electron distributions, and excitation energies, oscillator strengths and spin–orbit coupling matrix element (SOCME) values.

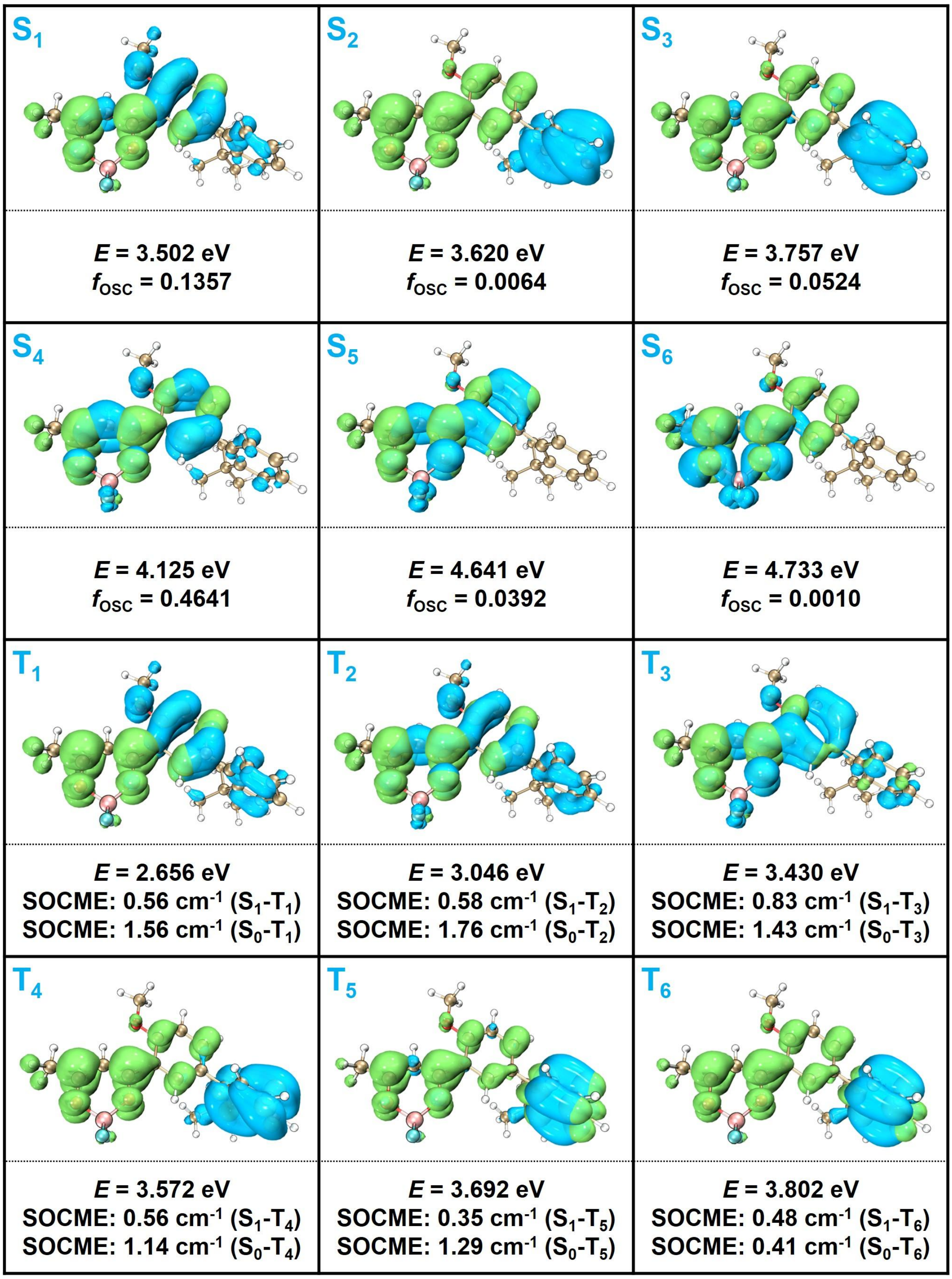

Figure S9. Iso-surface maps of electron-hole density difference of compound **4**'s excited states calculated at B3LYP/def2-TZVP(-f) level, where blue and green iso-surfaces correspond to hole and electron distributions, and excitation energies, oscillator strengths and spin–orbit coupling matrix element (SOCME) values.

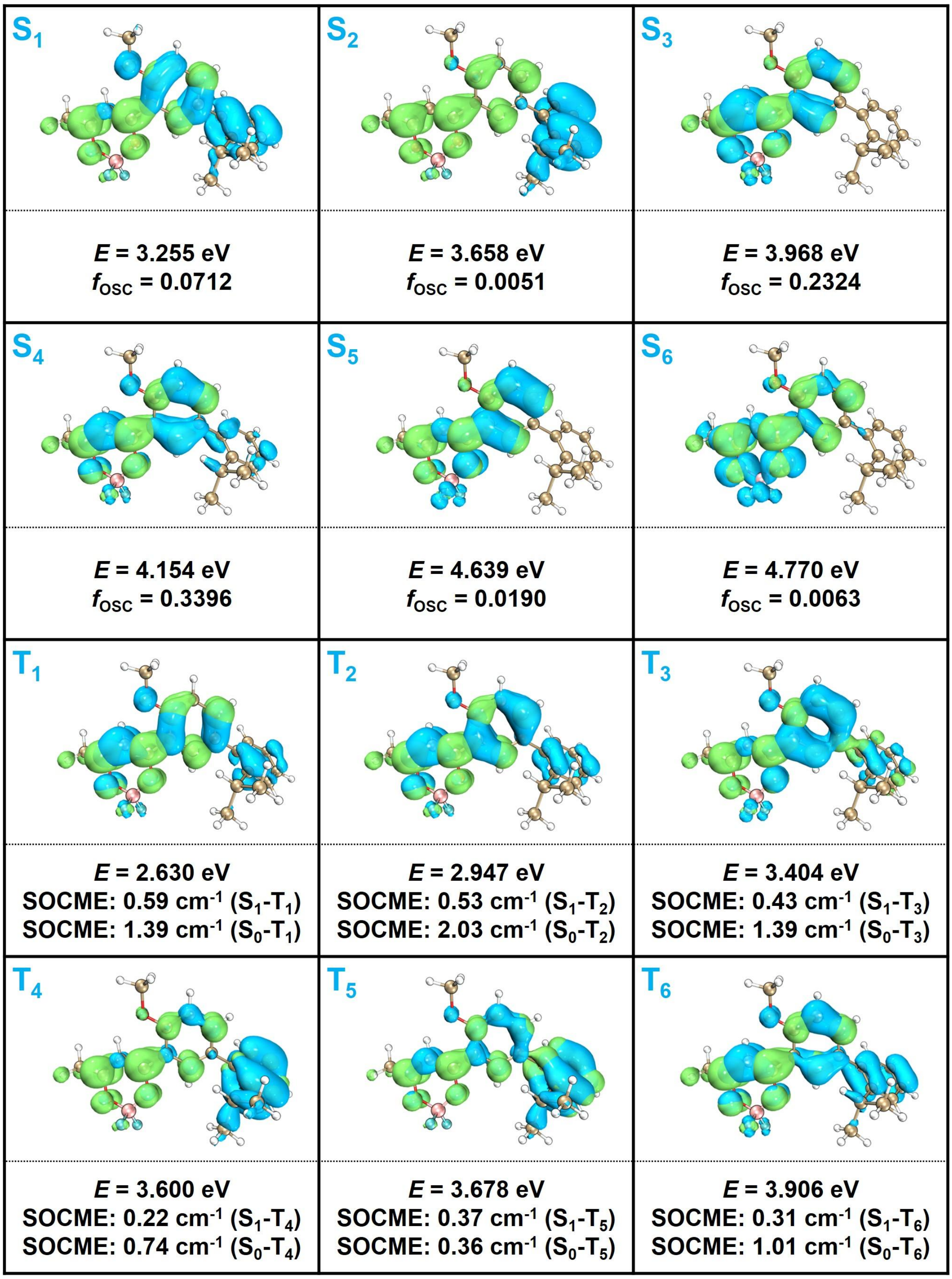

Figure S10. Iso-surface maps of electron-hole density difference of compound **5**'s excited states calculated at B3LYP/def2-TZVP(-f) level, where blue and green iso-surfaces correspond to hole and electron distributions, and excitation energies, oscillator strengths and spin–orbit coupling matrix element (SOCME) values.

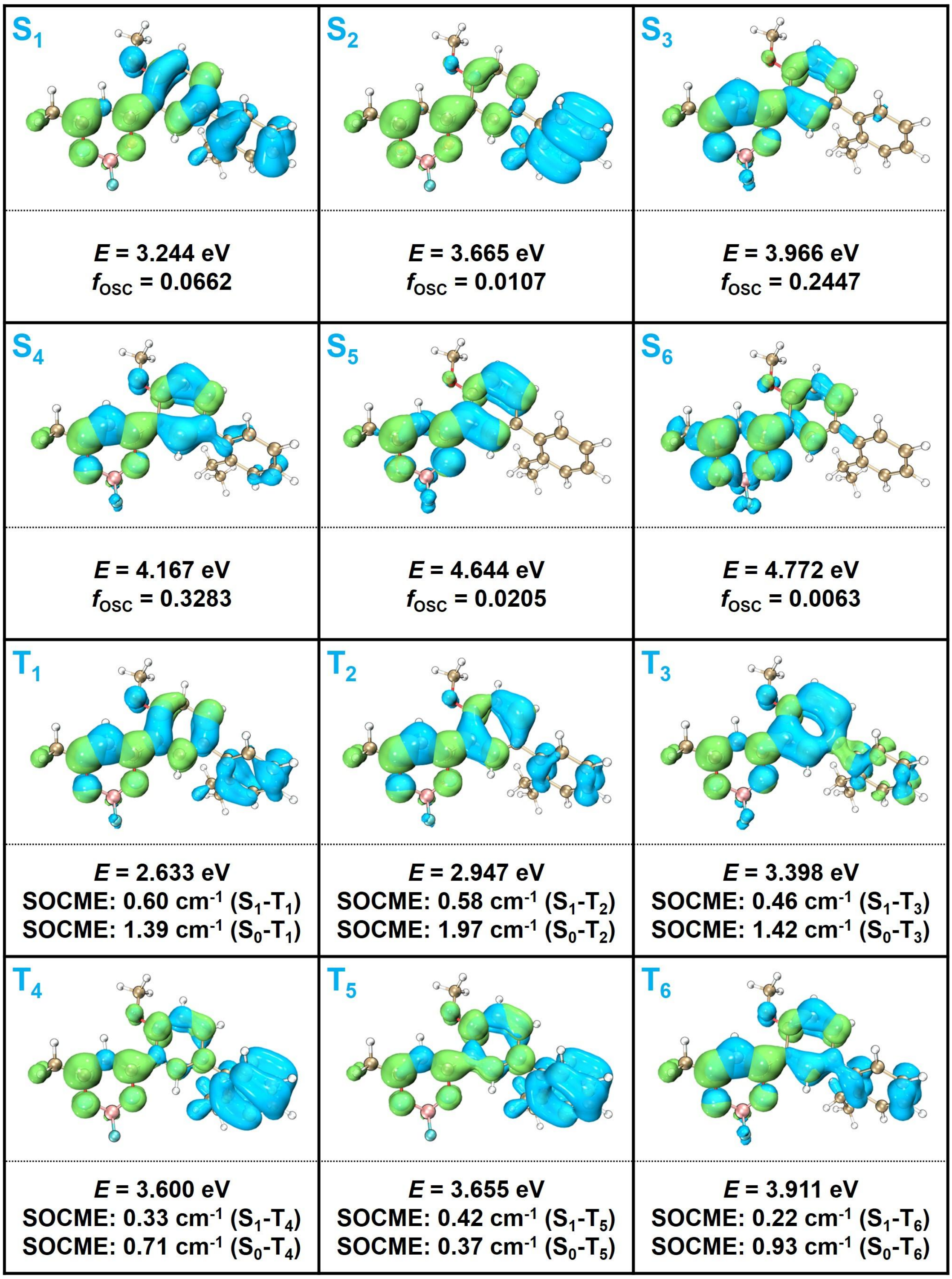

Figure S11. Iso-surface maps of electron-hole density difference of compound **6**'s excited states calculated at B3LYP/def2-TZVP(-f) level, where blue and green iso-surfaces correspond to hole and electron distributions, and excitation energies, oscillator strengths and spin–orbit coupling matrix element (SOCME) values.

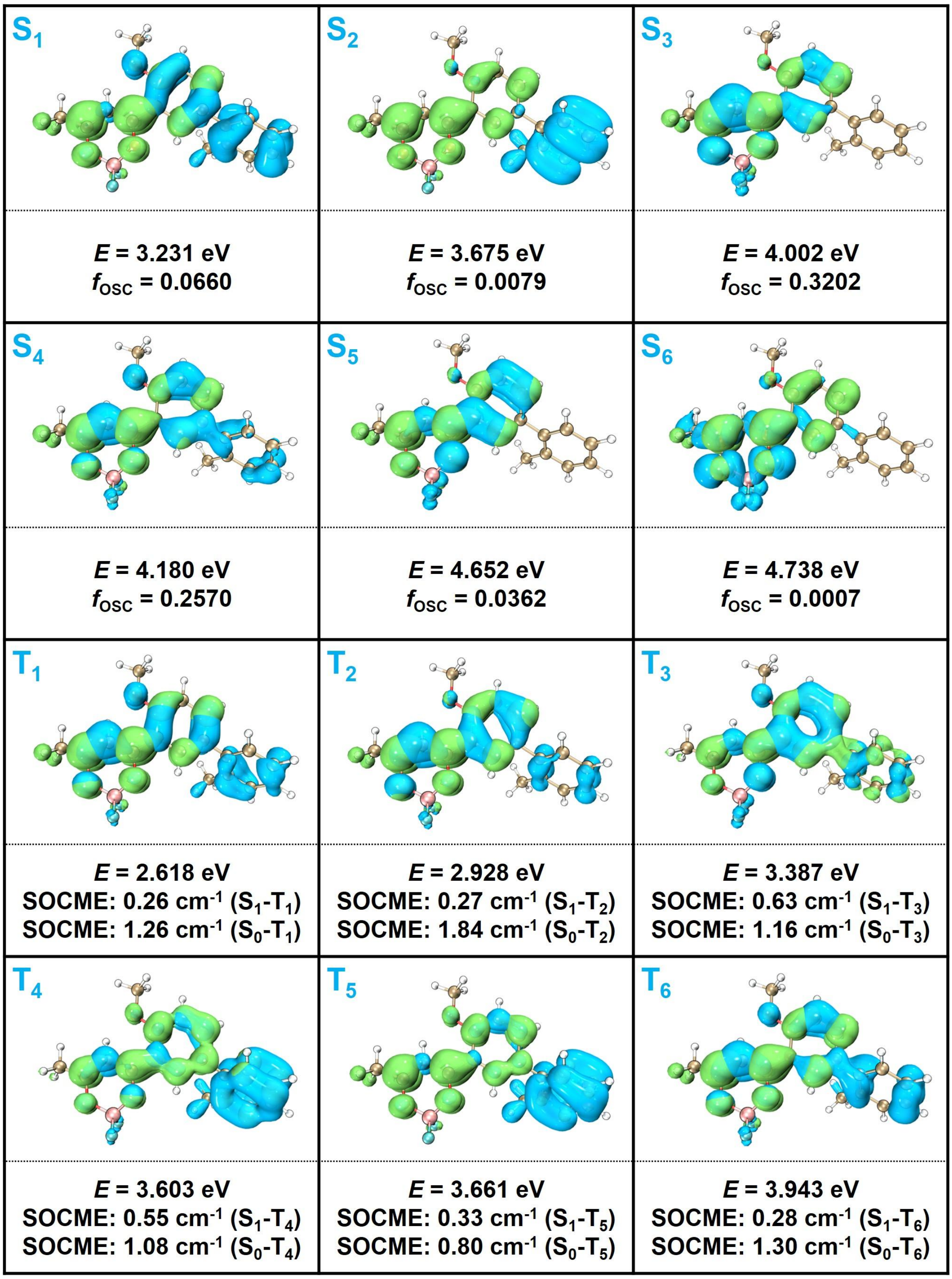

Figure S12. Iso-surface maps of electron-hole density difference of compound **7**'s excited states calculated at B3LYP/def2-TZVP(-f) level, where blue and green iso-surfaces correspond to hole and electron distributions, and excitation energies, oscillator strengths and spin–orbit coupling matrix element (SOCME) values.

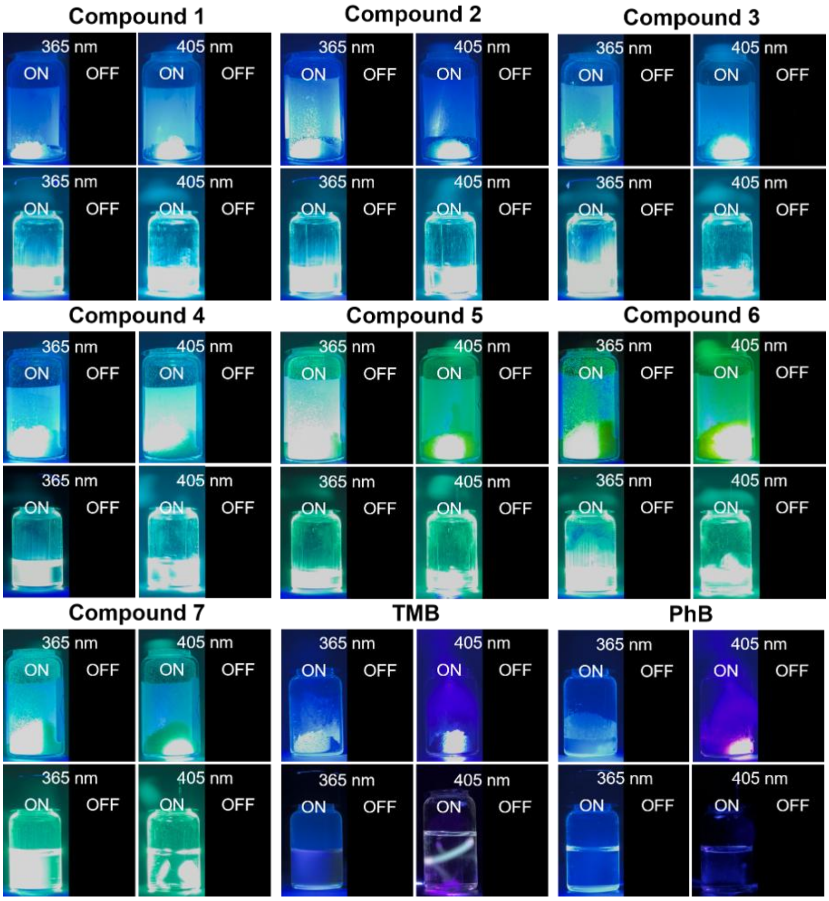

Figure S13. Photos of compounds **1-7**, TMB, and PhB in solid state and dichloromethane solution under 365 nm light source or 405 nm light and after removal of light.

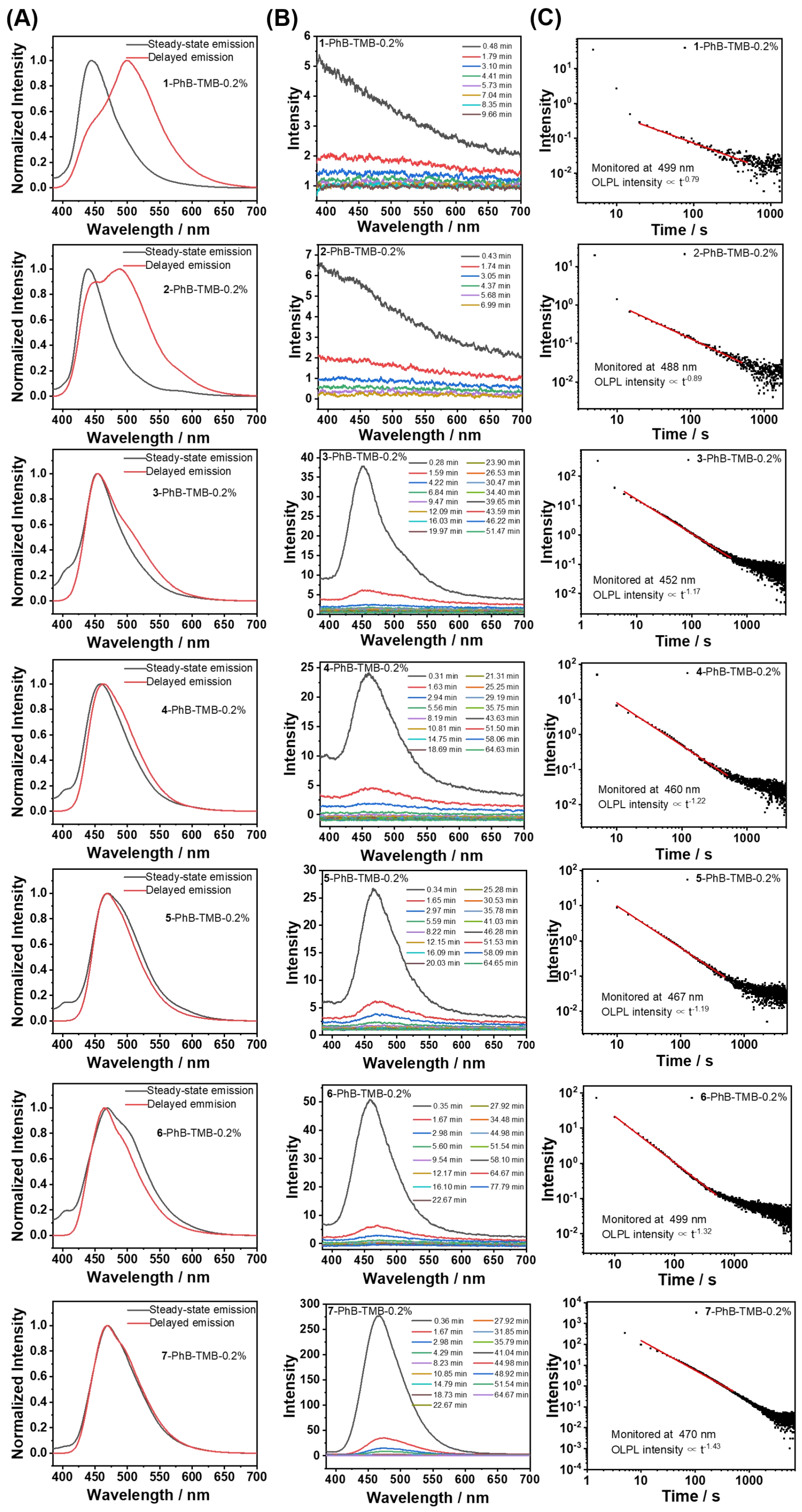

Figure S14. (A) The steady-state and delayed emission spectra (1 ms delay) of **BF₂bdk**-PhB-TMB-0.2% afterglow materials excited at 365 nm. (B) The OLPL emission spectra (at different delay time) of **BF₂bdk**-PhB-TMB-0.2% afterglow materials excited at 365 nm. (C) The OLPL emission decay profiles of **BF₂bdk**-PhB-TMB-0.2% afterglow materials excited at 365 nm.

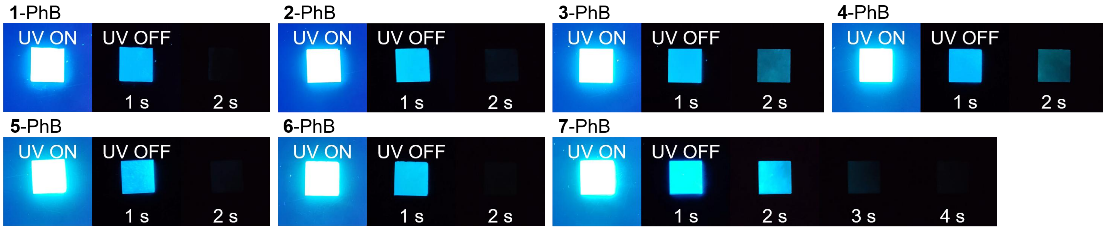

Figure S15. Photographs of **BF$_2$bdk**-PhB-0.2% afterglow materials under 365 nm UV excitation and after the removal of the UV excitation.

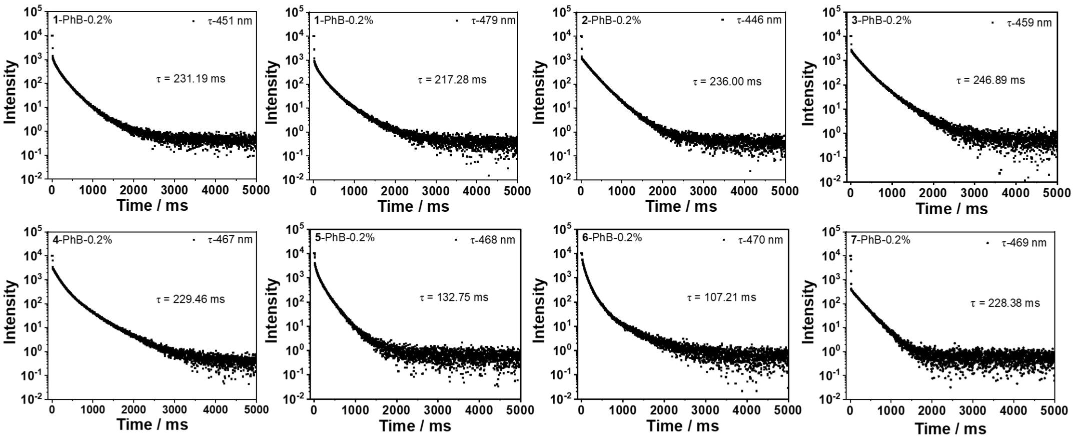

Figure S16. Emission decay of **BF$_2$bdk**-PhB-0.2% samples excited at 365 nm and monitored at different wavelengths. For the Hitachi FL-4700 instrument used in the present study, the baseline intensity is on the order of 10^{-2} ~ 10^{-1} (for instance, in Figure 2D, Figure 3 and Figure 4D). In this figure, one can find that the emission intensity in the range of 3 s to 5 s is on the order of 10^{-1} ~ 10^{0}, slightly larger than the baseline intensity. These very weak and long-persistent signals can be attributed to third lifetime components. According to our previous study (*Chem. Eng. J.* **2023**, *460*, 141916), **BF$_2$bdk**-PhB samples have been reported to show significant OLPL property after being excited by high-power UV lamp. Here the power of 365 nm excitation source from Hitachi FL-4700 instrument is much weaker than high-power UV lamp, so it is understandable that the **BF$_2$bdk**-PhB samples in the present study show very weak and long-persistent signals. Fortunately, such very weak signals have insignificant or very small influence on the fitting of emission decay profiles to obtain phosphorescence or TADF lifetimes. Regarding the shape of the emission decay profiles, we perform additional experiments to explain these observations. First,

considering that the complicated emission decay may be caused by molecular aggregation, we collect the emission decay profiles of **BF_2bdk**-PhB samples at low doping concentrations (Figure S31). The **7**-PhB sample still shows an excellent multi-exponential decay compared to other samples (Figure S31), which suggest molecular aggregation may be not suitable to explain the complicated decay in the present study. Second, in view of that the complicated decay may originate from RTP/TADF dual emission, we monitor the emission decay of **BF_2bdk**-PhB samples at different wavelengths (Figure S30). In particular, the emission decay at 430 nm only contains the contribution of TADF. However, the **7**-PhB sample monitored at 430 nm still shows an excellent multi-exponential decay compared to other samples. This suggests that the complicated emission decay is unlikely to be caused by RTP/TADF dual emission. Third, the phosphorescence decay of **BF_2bdk**-PhB samples at 77 K exhibit clear exponential decay (Figure S32), which suggest the complicated decay at room temperature should not be caused by the heterogeneous microenvironment for **BF_2bdk**'s T_1 states. Based on our understanding on the dipole-dipole interaction between S_1-state **BF_2bdk** and ground-state PhB to facilitate reverse intersystem crossing (RISC) in our previous study (*Adv. Opt. Mater.* **2021**, 2101909; *Angew. Chem. Int. Ed.* **2021**, *60*, 17138; *Adv. Funct. Mater.* **2021**, 2110207), we propose that the complicated emission decay should be caused by the heterogeneous microenvironment of dipole-dipole interaction. Specifically, the increase of the size of alkyl substituents would complicate the mode of molecular packing of **BF_2bdk** in PhB matrix. Different packing mode would lead to different extent of dipole-dipole interaction between S_1-state **BF_2bdk** and ground-state PhB and consequently heterogeneous microenvironment for k_{RISC}. It is known that k_{RISC} is the rate-determine step for TADF afterglow, so **1**-PhB to **6**-PhB systems with larger substituents have complicated decay profiles. In contrast, **7**-PhB system with a small methyl substituent shows the clear multi-exponential decay.

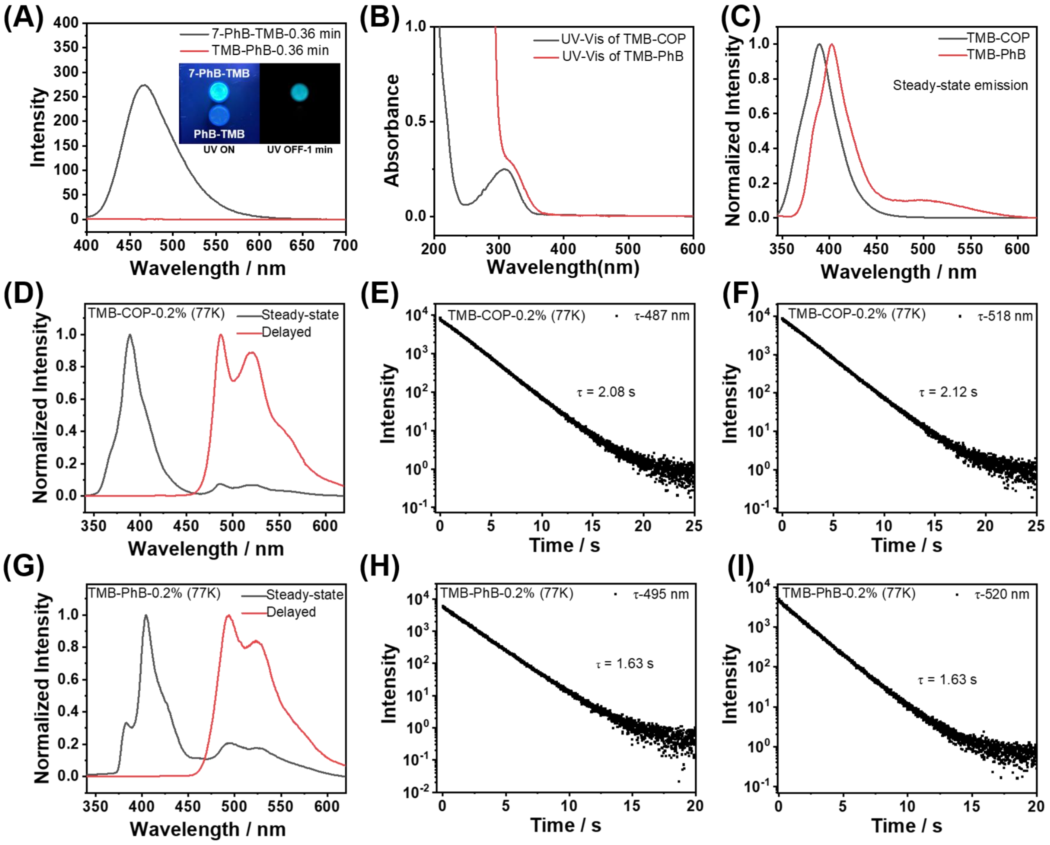

Figure S17. (A) Room-temperature delayed emission spectra (0.36 min delay) of **7-**PhB-TMB-0.2% (excited at 365 nm) and TMB-PhB-0.2% samples (excited at 320 nm). (B) UV-vis absorption spectra of TMB-COP-0.2% and TMB-PhB-0.2% samples, where COP represents cyclo olefin polymer. (C) Room-temperature steady-state emission spectra of TMB-COP-0.2% and TMB-PhB-0.2% samples. (D, E, F) Steady-state and delayed emission spectra (1 ms delay) and emission decay profile of TMB-COP-0.2% at 77 K excited at 320 nm. (G, H, I) Steady-state and delayed emission spectra (1 ms delay) and emission decay profile of TMB-PhB-0.2% at 77 K excited at 320 nm.

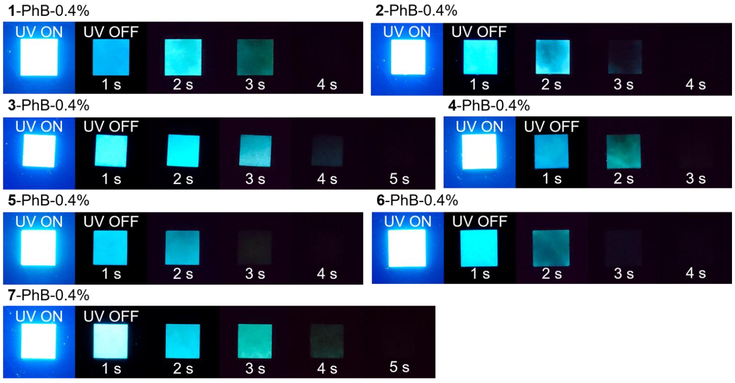

Figure S18. Photographs of **BF₂bdk**-PhB-0.4% afterglow materials under 365 nm UV excitation and after the removal of the UV excitation.

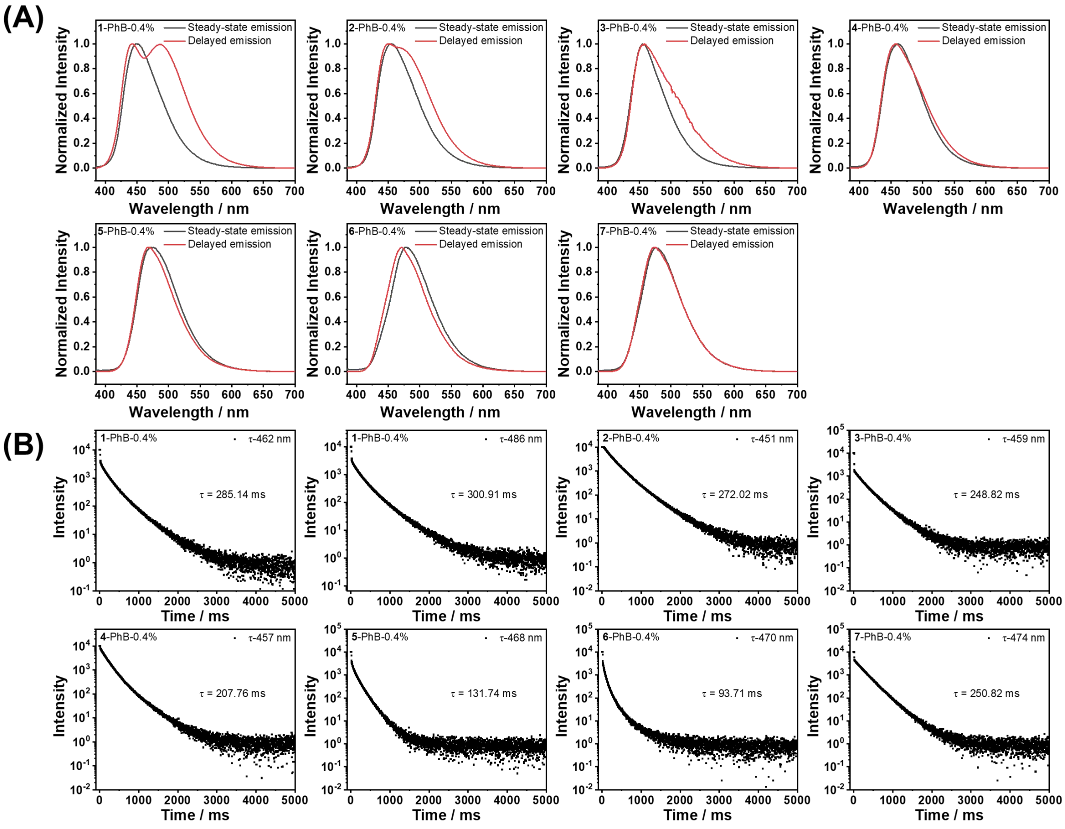

Figure S19. (A) Room temperature steady-state emission and delayed emission (1 ms delay) spectra of **BF₂bdk**-PhB-0.4% materials under ambient conditions. (B) Emission decay of **BF₂bdk**-PhB-0.4% samples excited at 365 nm and monitored at different wavelengths.

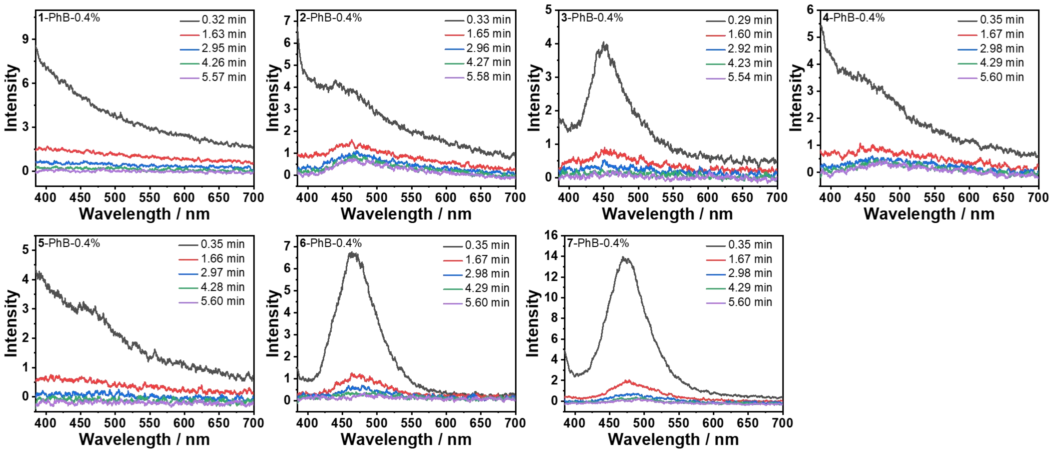

Figure S20. Delayed emission spectra (at different delay time) of **BF$_2$bdk**-PhB-0.4% afterglow materials excited at 365 nm.

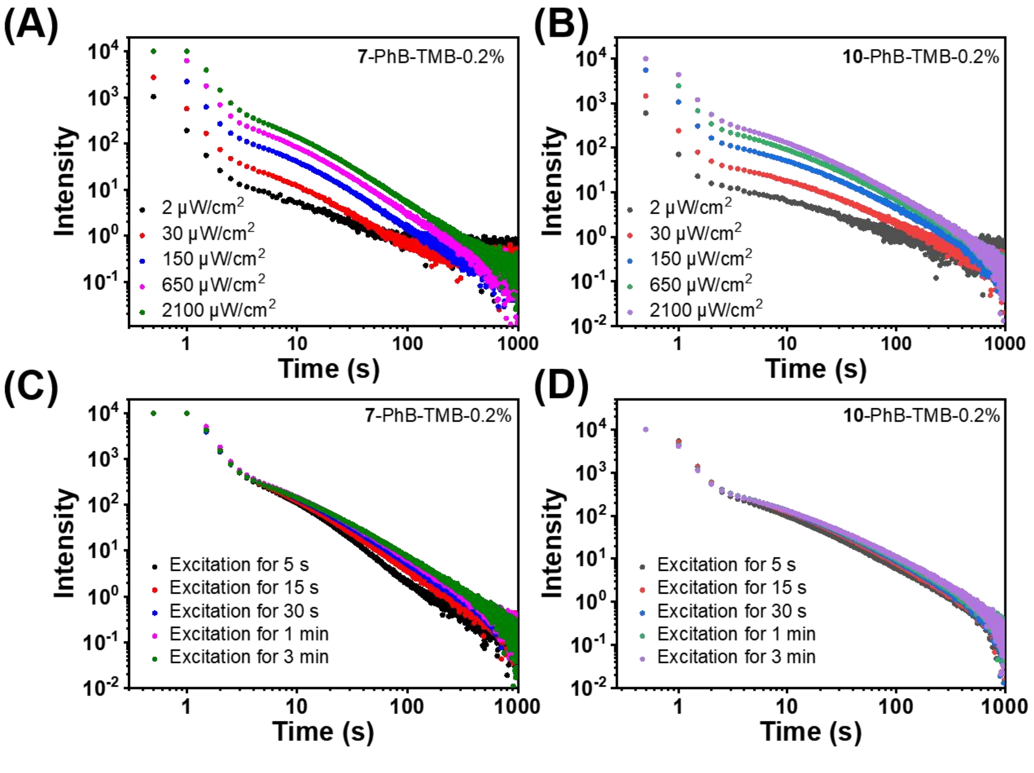

Figure S21. (A) Room-temperature emission decay profile (monitored at 470 nm) of **7**-TMB-PhB-0.2% excited by 365 nm of different excitation powers for 30 s. (B) Room-temperature emission decay profile (monitored at 501 nm) of **10**-TMB-PhB-0.2% excited by 365 nm of different excitation powers for 30 s. (C) Room-temperature emission decay profile (monitored at 470 nm) of **7**-PhB-TMB-0.2% excited by 365 nm UV light at 2.1 mW/cm^2. (D) Room-temperature emission decay

profile (monitored at 501 nm) of **10**-PhB-TMB-0.2% excited by 365 nm light at 2.1 mW/cm^2.

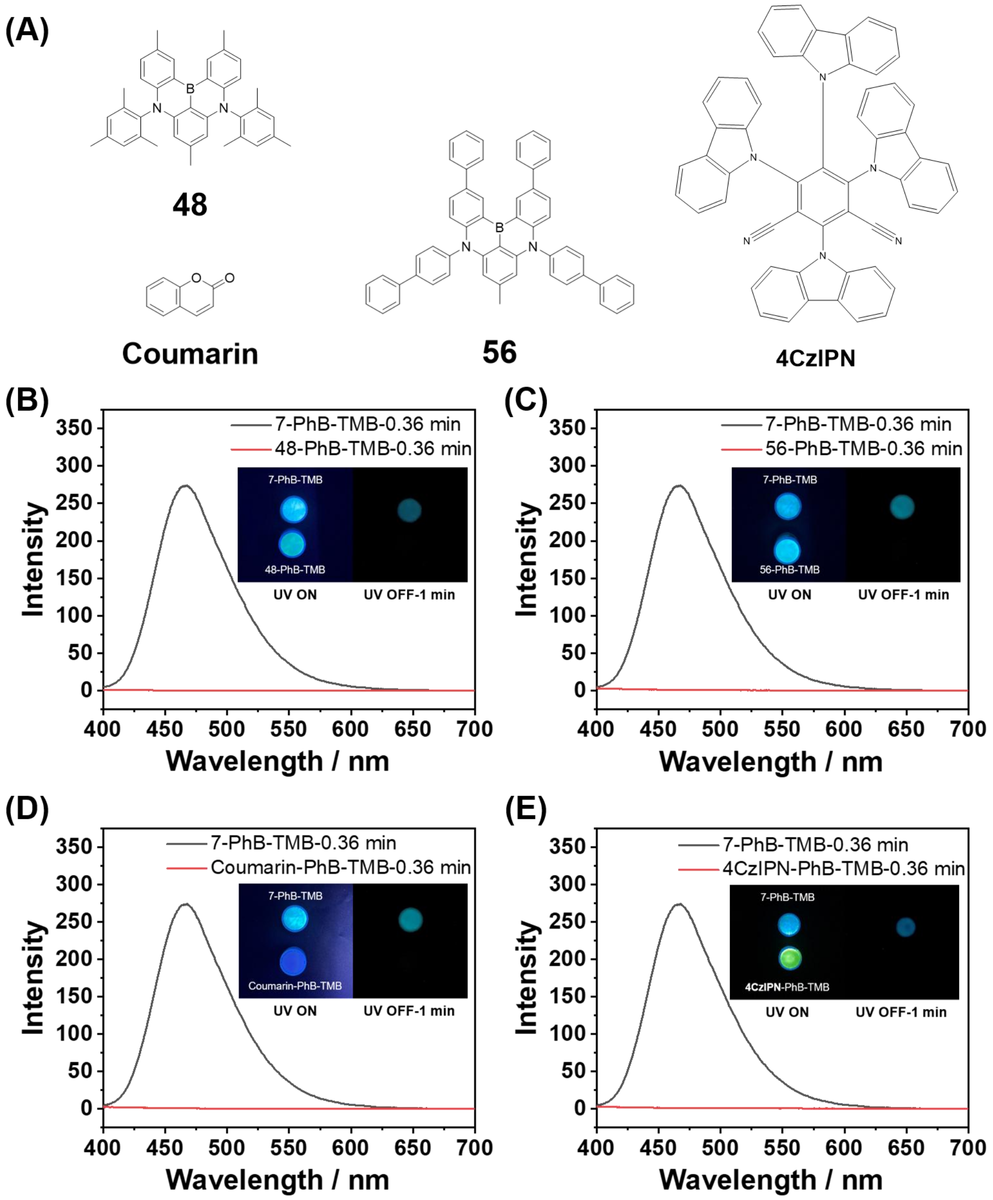

Figure S22. (A) Chemical structures of 48, 56, 4CzIPN and coumarin emitter dopants. (B) Room-temperature delayed emission spectra (0.36 min delay) of **7**-PhB-TMB-0.2% (excited at 365 nm) and 48-PhB-TMB-0.2% samples (excited at 320 nm). (C) Room-temperature delayed emission spectra (0.36 min delay) of **7**-PhB-TMB-0.2% (excited at 365 nm) and 56-PhB-TMB-0.2% samples (excited at 320 nm). (D) Room-temperature delayed emission spectra (0.36 min delay) of **7**-PhB-TMB-0.2% (excited at 365 nm) and 4CzIPN-PhB-TMB-0.2% samples (excited at 320 nm). (E) Room-temperature delayed emission spectra (0.36 min delay) of **7**-PhB-TMB-0.2% (excited

at 365 nm) and coumarin-PhB-TMB-0.2% samples (excited at 320 nm). Given that charge separation is the prerequisite for OLPL (the population of long-lived excited states and the energy level structures in the three-component systems should be important for the charge-separating process), the absence of OLPL in Figure S22 should be either caused by less population of long-lived excited states or the mismatch of HOMO/LUMO energy level structures.

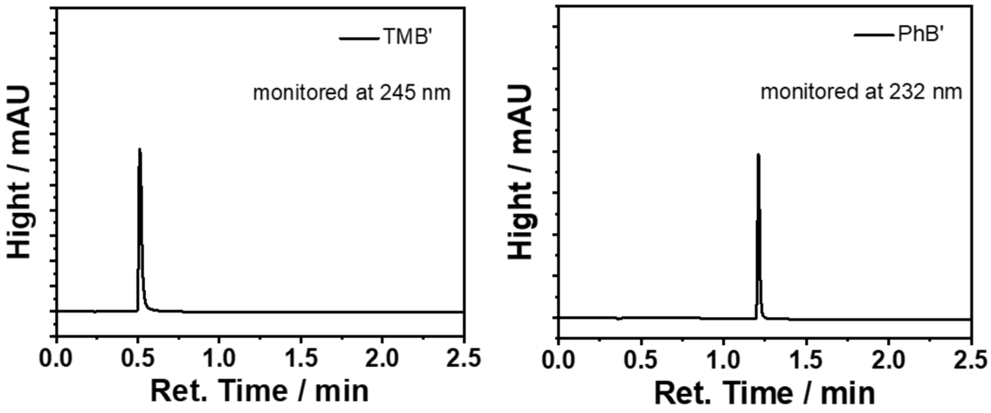

Figure S23. HPLC profile of further purified TMB and purified PhB, denoted as TMB' and PhB', respectively.

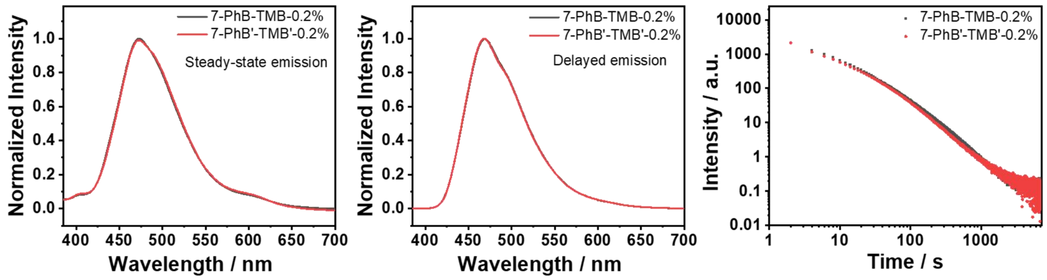

Figure S24. Steady-state and delayed emission spectra (1 ms delay) and emission decay profile (monitored at 470 nm) of **7**-PhB-TMB-0.2% and **7**-PhB'-TMB'-0.2% afterglow materials excited at 365 nm, where PhB' and TMB' represents the further purified PhB and TMB.

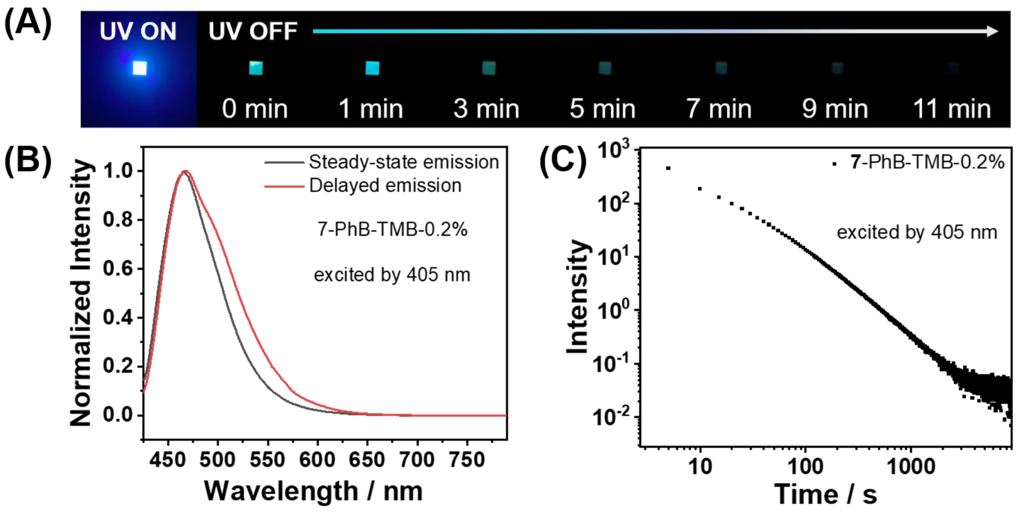

Figure S25. (A) Photographs of **7**-PhB-TMB under 405 nm UV light and after removal of the UV light. (B) Steady-state and delayed emission (1 ms delay) spectra of **7**-PhB-TMB excited at 405 nm. (C) Emission decay profiles (1 ms delay, monitored at 470 nm) of **7**-PhB-TMB excited at 405 nm.

Table S3. The HOMO and LUMO levels obtained by DFT calculation at B3LYP/6-31g(d,p) level.

Compounds	LUMO / eV	HOMO / eV
1	-2.24	-6.33
2	-2.24	-6.31
3	-2.24	-6.35
4	-2.24	-6.33
5	-2.22	-6.15
6	-2.23	-6.19
7	-2.23	-6.12[a]
8	-2.30	-5.94
9	-2.31	-5.95
10	-2.65	-5.99[a]

[a] It has been found that the HOMO levels of compounds **7** and **10** (-6.14 eV and -6.06 eV) by cyclic voltammetry agree well with the results by DFT (-6.12 eV and -5.99 eV).

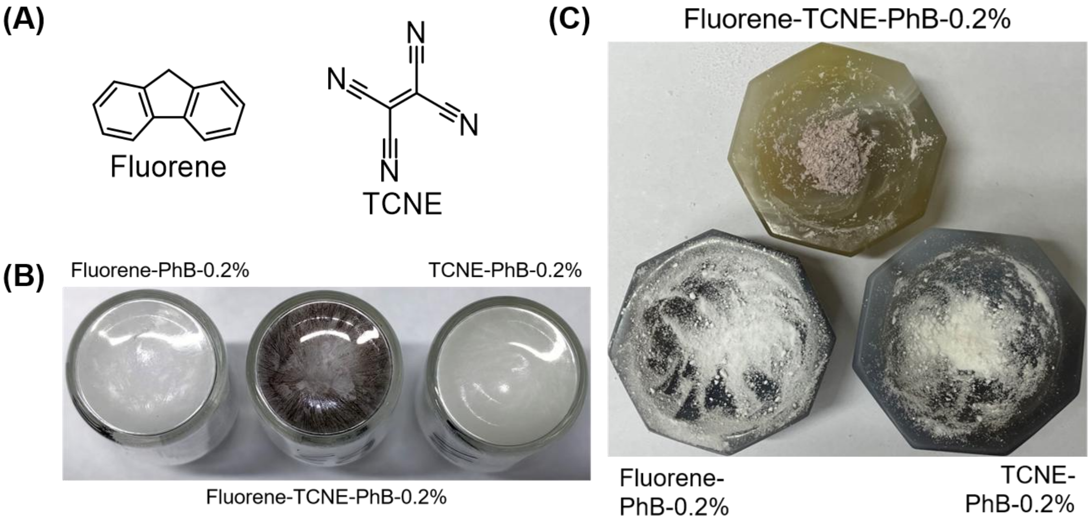

Figure S26. (A) Chemical structures of fluorene and tetracyanoethylene (TCNE). (B) Photographs of melt-cast samples of fluorene-PhB, fluorene-TCNE-PhB and TCNE-PhB under room light. (C) Photographs under room light of fluorene-PhB and TCNE-PhB samples before mixing, and fluorene-TCNE-PhB samples after mixing fluorene-PhB and TCNE-PhB samples by mechanical grinding.

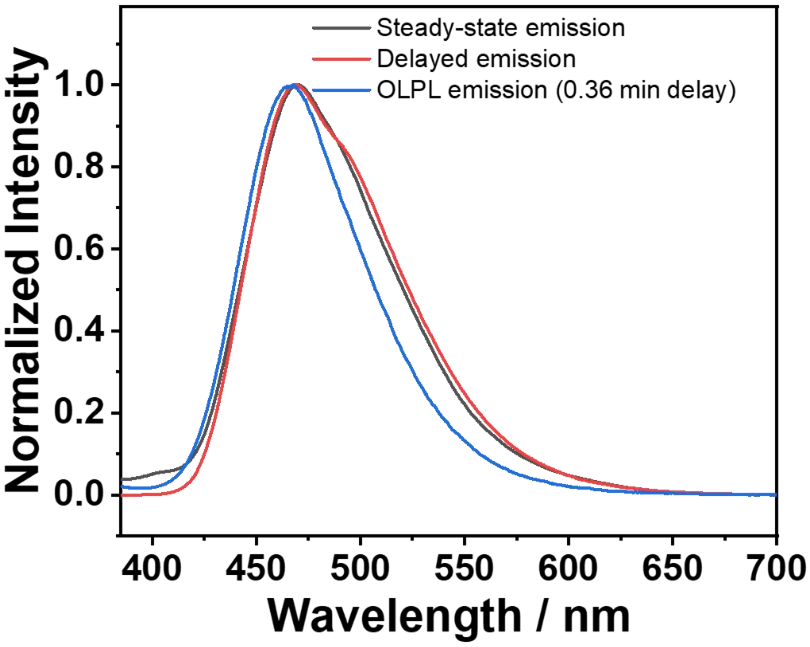

Figure S27. Steady-state spectra, delayed emission spectra, and OLPL spectra (0.36 min delay) of **7**-PhB-TMB material at 470 nm.

Table S4. Photophysical property of BF_2bdk-PhB-0.2% afterglow materials.

BF_2bdk	λ_F / nm	λ_{DF} / nm	τ_{DF} / ms	$\lambda_F{}^a$ / nm	$\lambda_P{}^a$ / nm	$\tau_P{}^a$ / ms	$\Delta E_{ST}{}^b$ / eV	PLQY / %
1	459	451	231	451	515	1021	0.342	33.6
2	444	446	236	446	515	1044	0.373	51.4
3	463	459	247	449	519	1039	0.372	44.1
4	469	467	229	453	501	1121	0.262	34.7
5	469	468	133	469	527	1210	0.291	48.6
6	468	470	107	466	501	1098	0.186	48.2
7	469	468	228	470	506	1087	0.188	51.7
8	490	488	105	488	538	1549	0.236	61.3
9	497	502	90	506	542	1464	0.163	57.3
10	494	494	203	501	542	1072	0.187	95.7

[a]at 77 K; [b]estimated from fluorescence and phosphorescence maxima at 77 K.

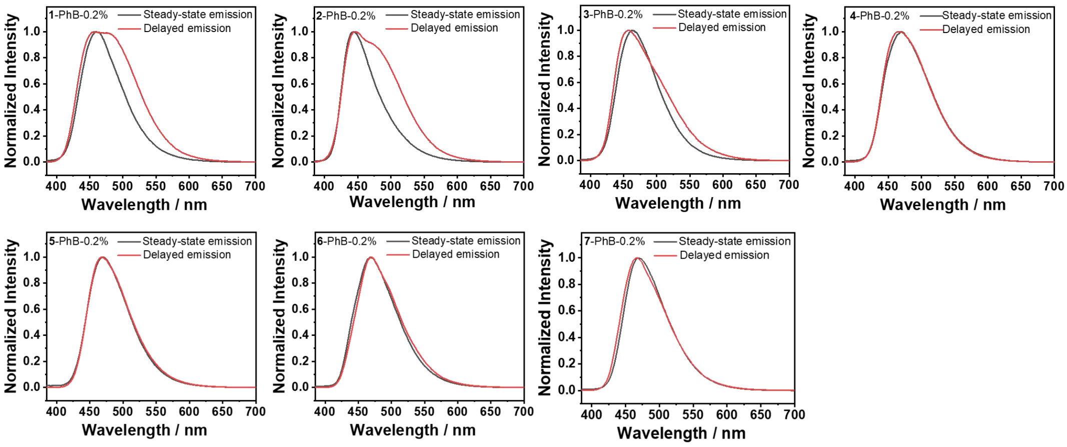

Figure S28. Room temperature steady-state emission and delayed emission (1 ms delay) spectra of **BF_2bdk**-PhB-0.2% material under ambient conditions.

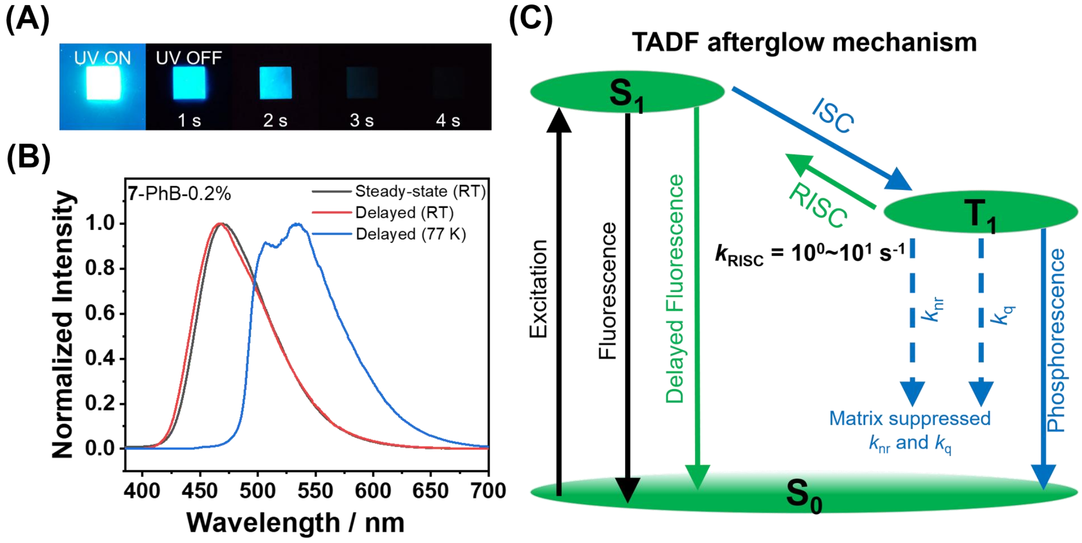

Figure S29. (A) Photographs of **7**-PhB under 365 nm UV light and after removal of the UV light. (B) Steady-state and delayed emission (1 ms delay) spectra of **7**-PhB at room temperature, as well as delayed emission (1 ms delay) spectrum of **7**-PhB at 77 K. (C) Schematic diagram of TADF afterglow mechanism of **7**-PhB materials.

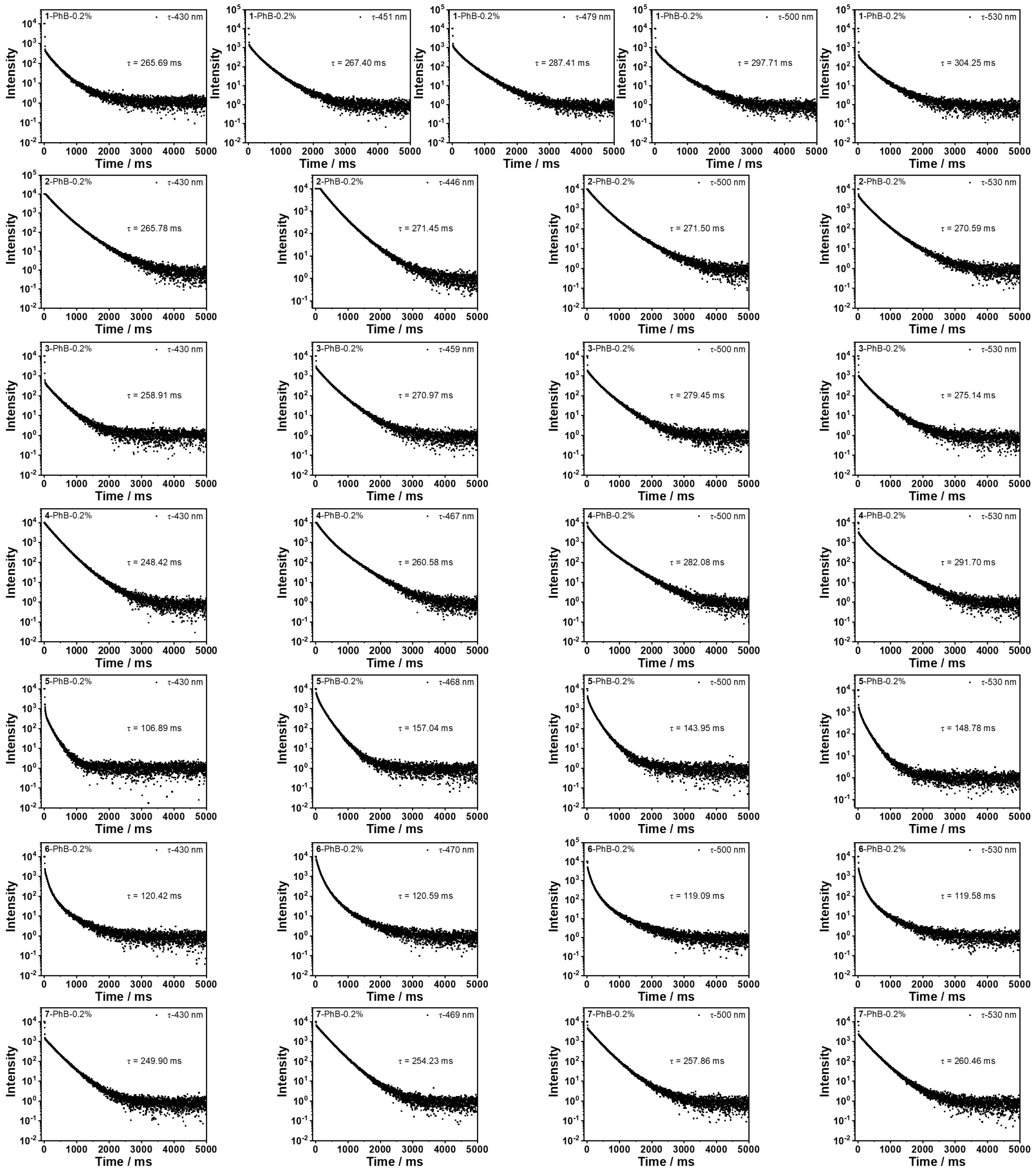

Figure S30. The delayed emission decay of fresh-prepared **BF_2bdk**-PhB-0.2% samples excited at 365 nm and monitored at different wavelengths.

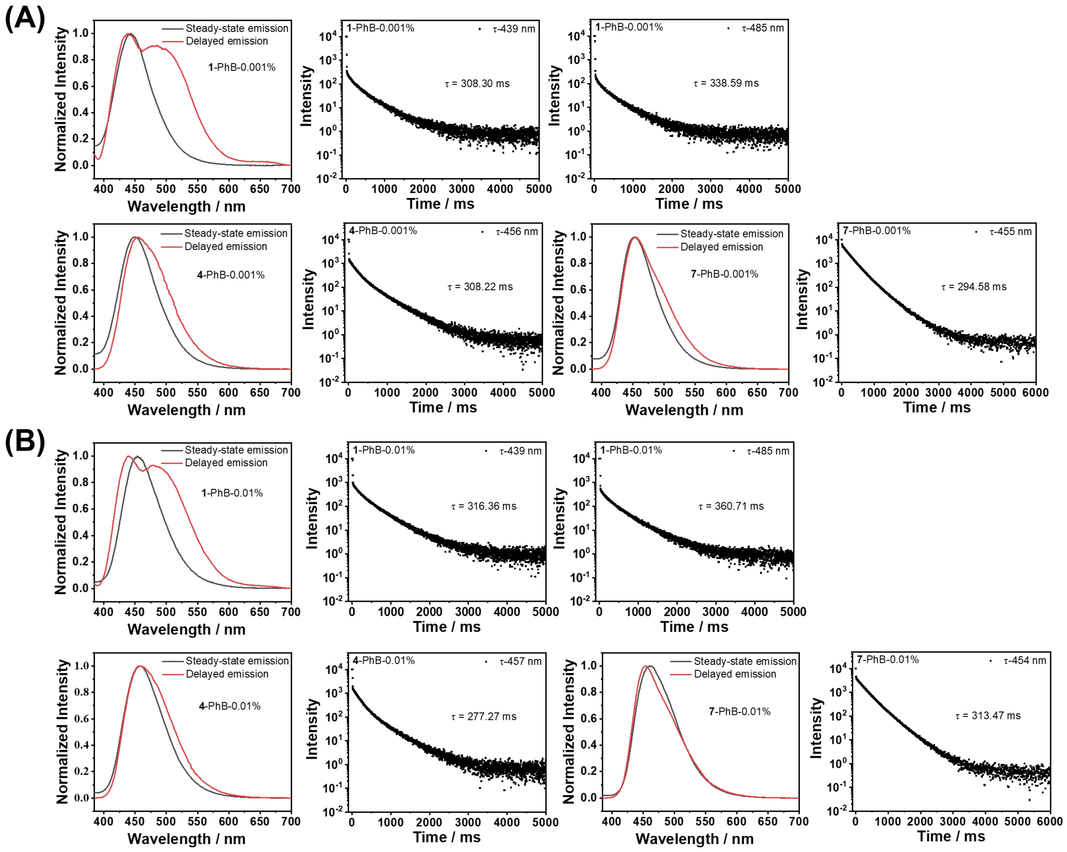

Figure S31. The steady-state and delayed emission spectra, emission decay spectra of **1**/**4**/**7**-PhB-0.001% (A) and **1**/**4**/**7**-PhB-0.01% (B) afterglow materials excited at 365 nm.

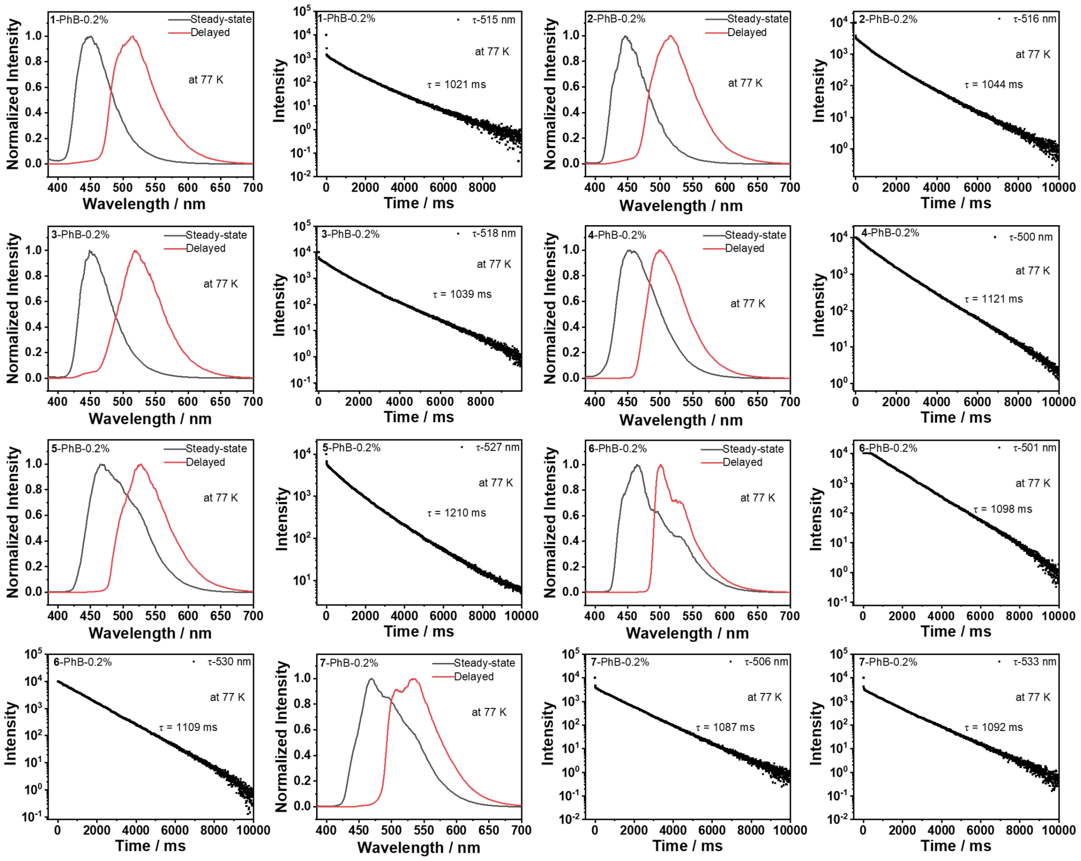

Figure S32. Steady-state and delayed emission (1 ms delay) spectra and phosphorescence decay of **BF_2bdk**-PhB-0.2% samples at 77 K excited at 365 nm.

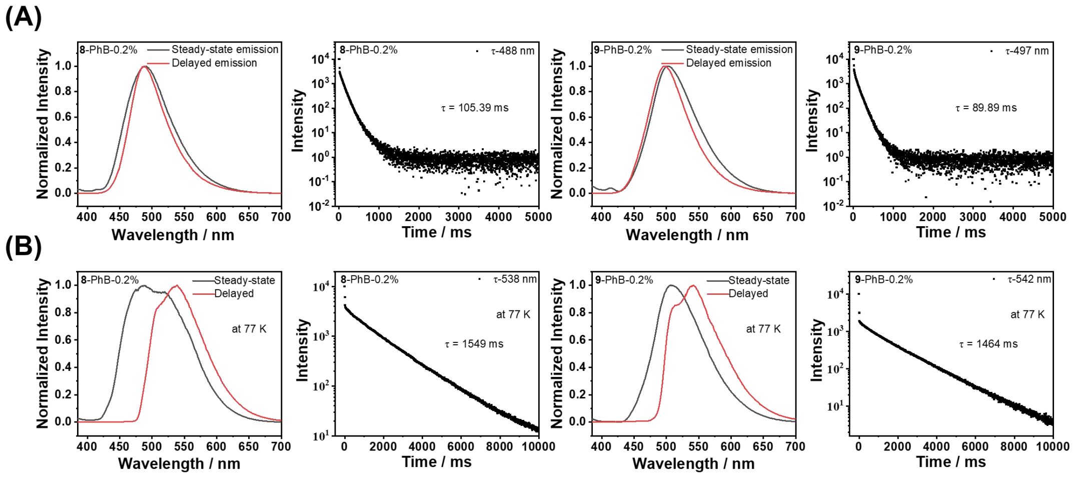

Figure S33. (A) Steady-state and delayed emission (1 ms delay) spectra, emission decay profiles (monitored at 488/497 nm) of **8**/**9**-PhB-0.2% samples. (B) Steady-state and delayed emission (1 ms delay) spectra and phosphorescence decay of **8**/**9**-PhB-0.2% samples at 77 K excited at 365 nm.

Table S5. Dihedral angle, electronic energy, and free energy of radical cations in this work.

Compounds	Dihedral angle of radical cations (°)	electronic energy (Hartree)	electronic energy (kcal / mol) relative to 7 or TMB	electronic energy	free energy (kcal / mol) relative to 7 or TMB
1	50.1	-1107.26067356	3.358407	-1107.0203182	3.079379
2	49.1	-1107.26112833	3.073035	-1107.0207395	2.815009
3	45.7	-1107.26247132	2.230295	-1107.0219961	2.026480
4	40.0	-1107.26441423	1.011100	-1107.0237745	0.910517
5	37.0	-1107.26526684	0.476079	-1107.0245443	0.427460
6	35.3	-1107.26571371	0.195664	-1107.0249457	0.175577
7	33.9	-1107.26602552	0	-1107.0252255	0
TMB	17.0	-731.211162295	0	-730.9276155	0
TMB1	45.7	-731.206947377	2.644902	-730.9238224	2.380207
TMB2	53.4	-731.204738219	4.031170	-730.9217578	3.675764

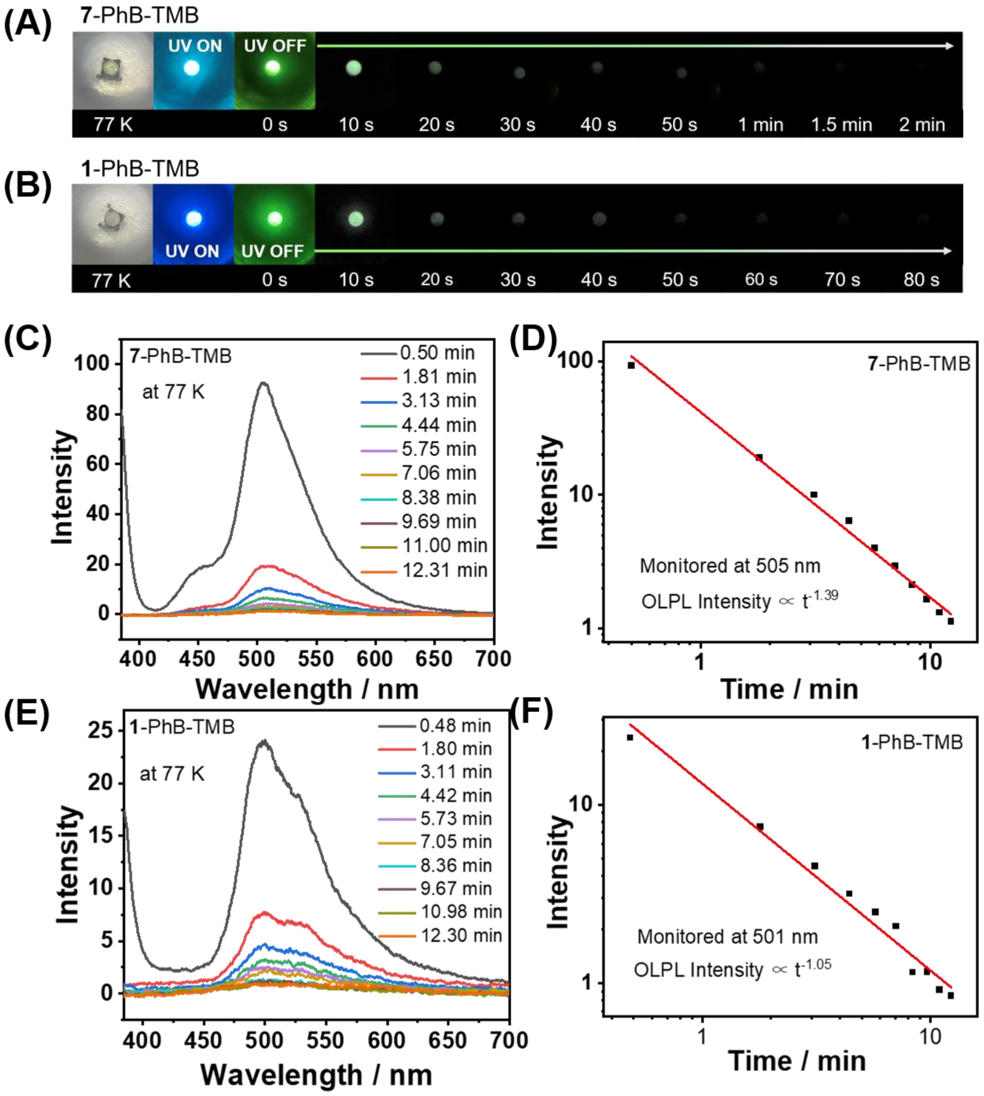

Figure S34. (A, B) Photographs of (A) **7**-PhB-TMB and (B) **1**-PhB-TMB materials at 77 K under daylight lamp, 365 nm ultraviolet light, and after removal of ultraviolet light. (C, E) Delayed emission spectra of (C) **7**-PhB-TMB and (E) **1**-PhB-TMB materials excited at 365 nm at 77 K. (D, F) Logarithmic plot of OLPL emission attenuation curve of (D) **7**-PhB-TMB material at 77 K (monitored at 505 nm) and (F) **1**-PhB-TMB material at 77 K (monitored at 501 nm).

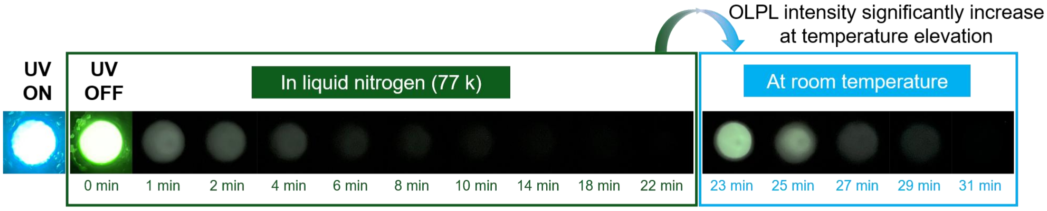

Figure S35. Photographs of the afterglow property change of upon transferring **7**-PhB-TMB materials from liquid nitrogen to room temperature.

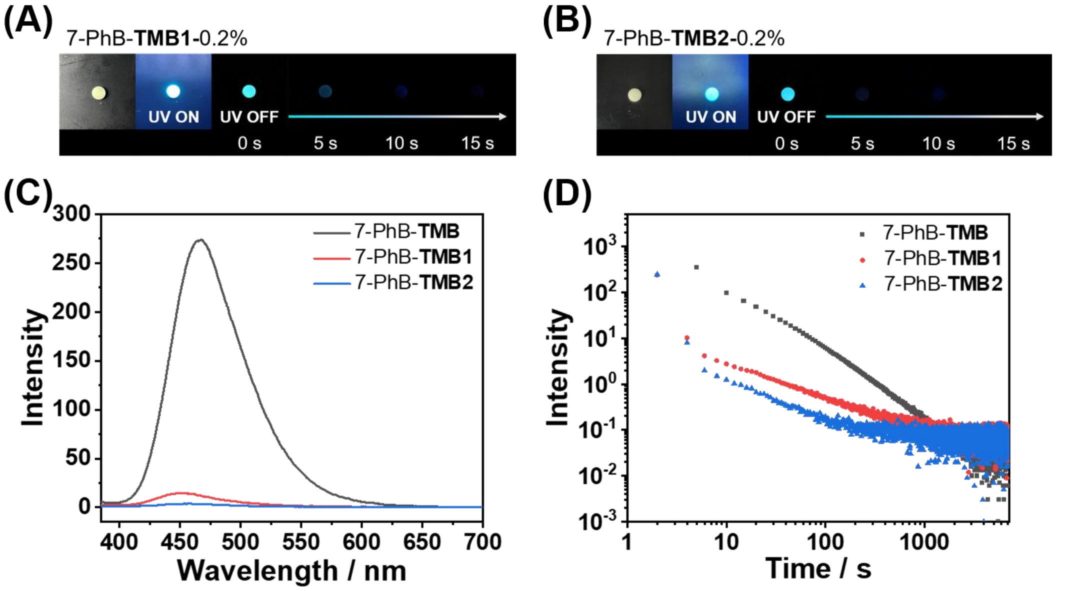

Figure S36. (A, B) Photographs of (A) 7-PhB-**TMB1** and (B) 7-PhB-**TMB2** under daylight lamp, 365 nm UV light and after removal of the UV light. (C) Delayed emission spectra (0.36 delay) of the 7-PhB-**TMB**, 7-PhB-**TMB1** and 7-PhB-**TMB2** materials excited at 365 nm. (D) Emission decay profiles (monitored at 470 nm) of 7-PhB-**TMB**, 7-PhB-**TMB1** and 7-PhB-**TMB2** materials excited at 365 nm.

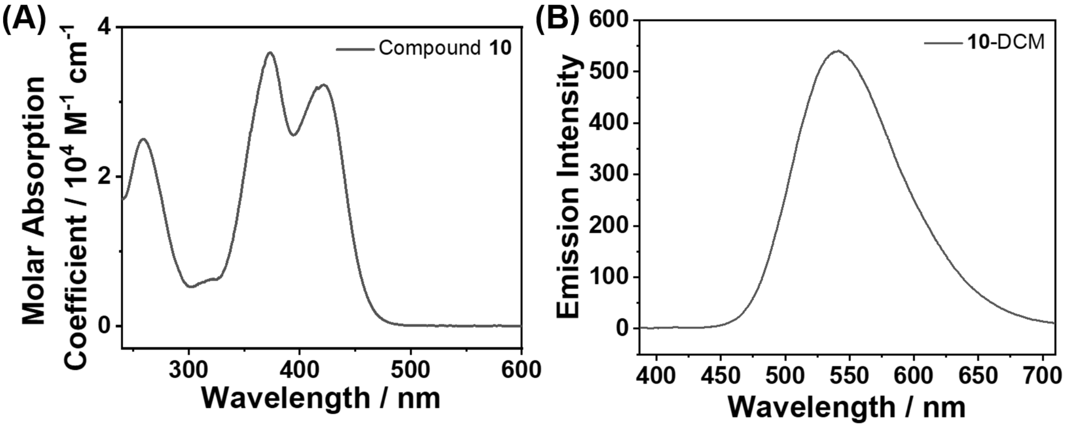

Figure S37. (A) Molar absorption coefficient and (B) steady-state emission spectrum of compound **10** in dichloromethane (DCM).

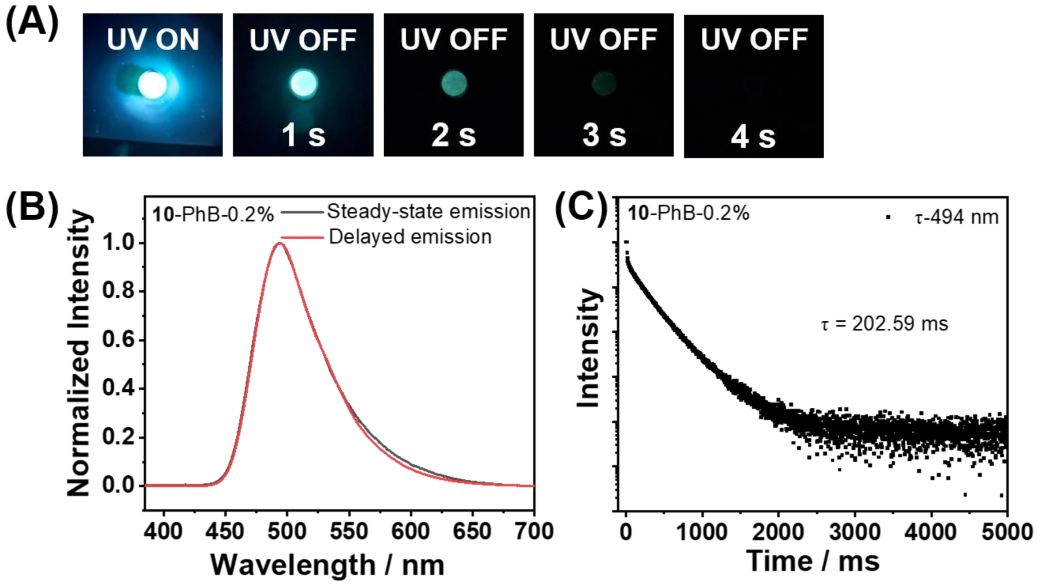

Figure S38. (A) Photographs of **10**-PhB sample under 365 nm UV light and after removal of the UV light. (B) Steady-state and delayed emission (1 ms delay) spectra of **10**-PhB sample. (C) Emission decay profiles (monitored at 494 nm) of **10**-PhB sample excited at 365 nm.

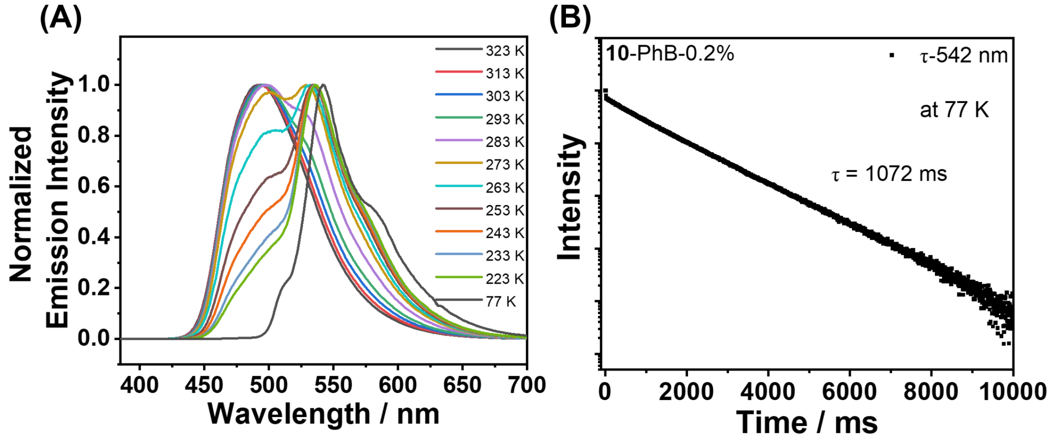

Figure S39. (A) Variable-temperature delayed emission spectra (1 ms delay) of **10**-PhB materials excited at 365 nm. (B) Emission decay profiles (monitored at 542 nm) of **10**-PhB material at 77 K.

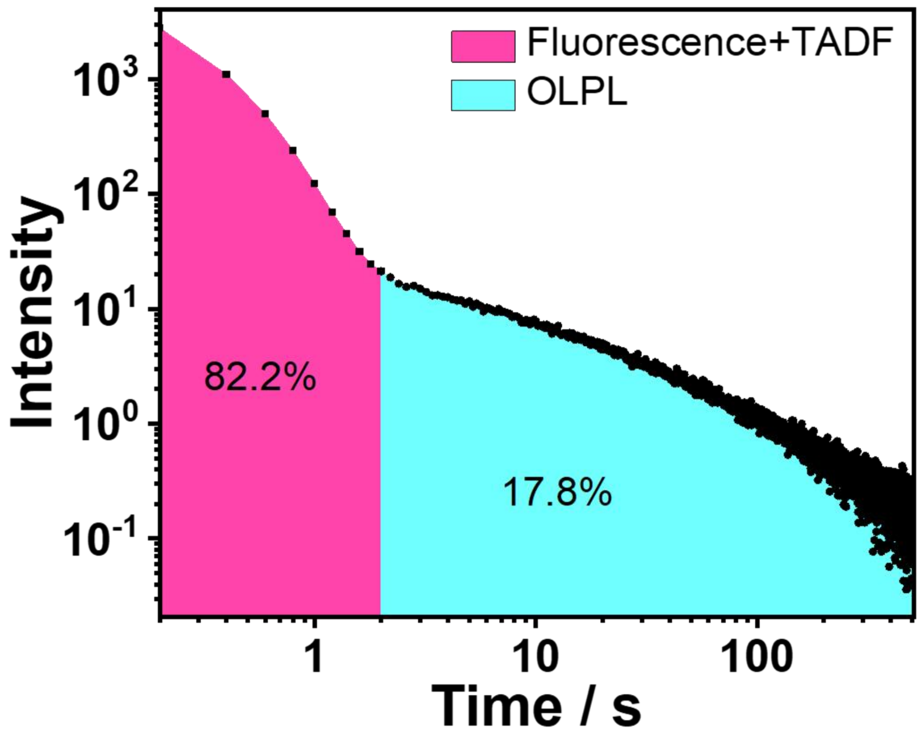

Figure S40. Percentage of different luminescent components obtained from emission decay profile (fluorescence plus TADF afterglow, and OLPL afterglow) in **10**-PhB-TMB-0.2% materials.

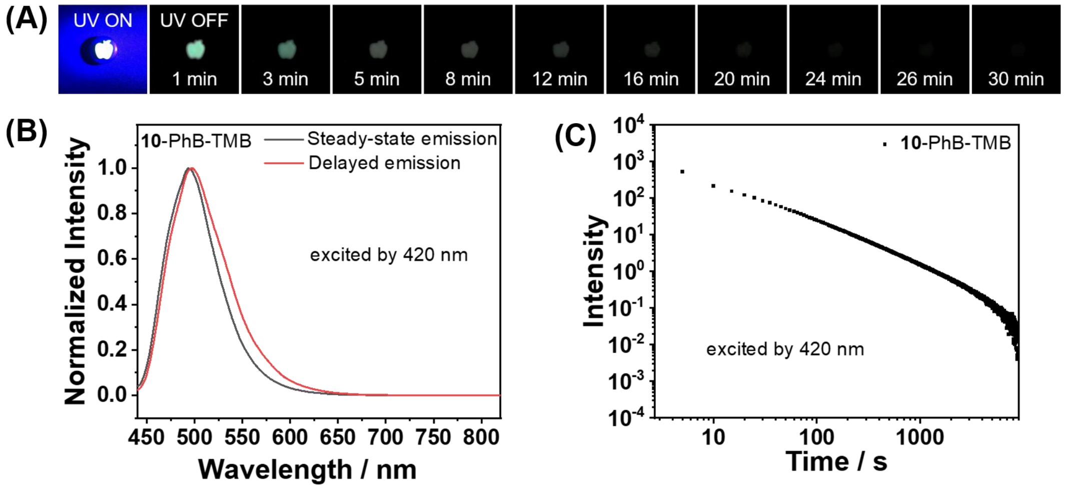

Figure S41. (A) Photographs of **10**-PhB-TMB melt-cast sample under 420 nm lamp and after removal of the lamp. (B) Steady-state and delayed emission (1 ms delay) spectra of **10**-PhB-TMB excited at 420 nm. (C) Delayed emission decay profiles (monitored at 497 nm) of **10**-PhB-TMB excited at 420 nm.

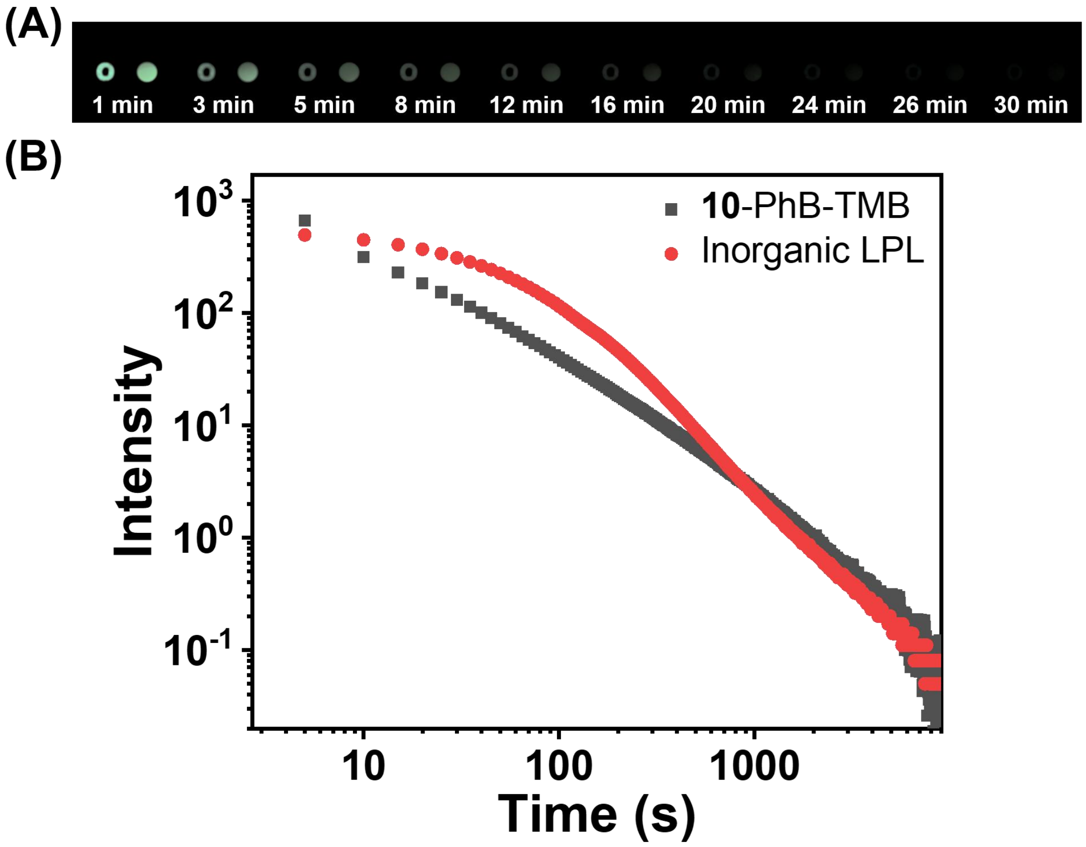

Figure S42. (A) Afterglow photographs of **10**-PhB-TMB materials (left) and inorganic $Sr_2Al_{14}O_{25}/Eu^{2+}$, Dy^{3+} materials (right). (B) Emission decay profiles (monitored at 501 nm) of **10**-PhB-TMB materials and emission decay profiles (monitored at 515 nm) of inorganic $Sr_2Al_{14}O_{25}/Eu^{2+}$, Dy^{3+} materials (produced by Obligatory Meteor Jewelry Co., Ltd).

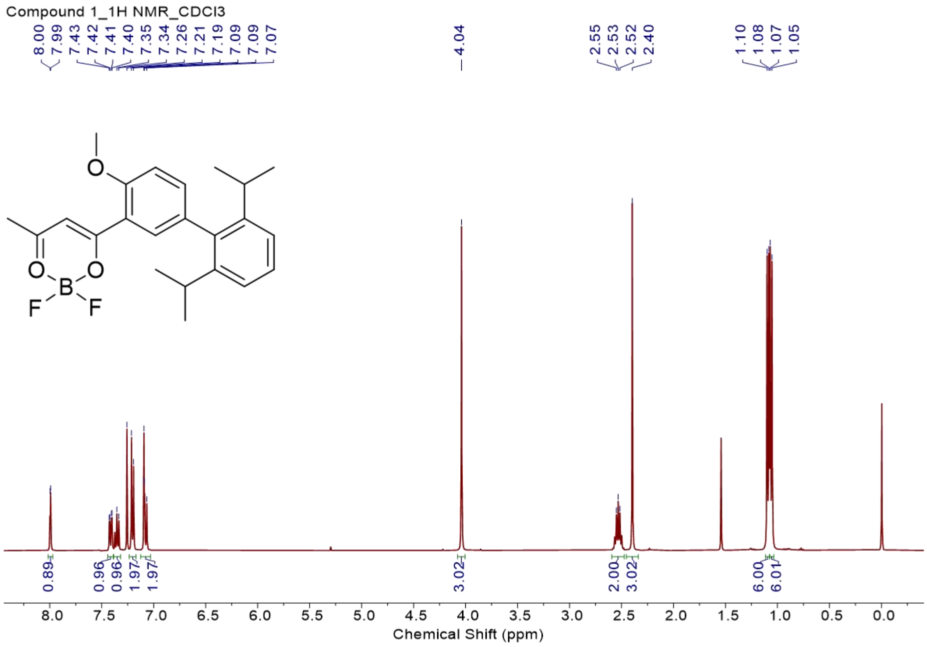

Figure S43. ^{1}H NMR spectra of compound **1**.

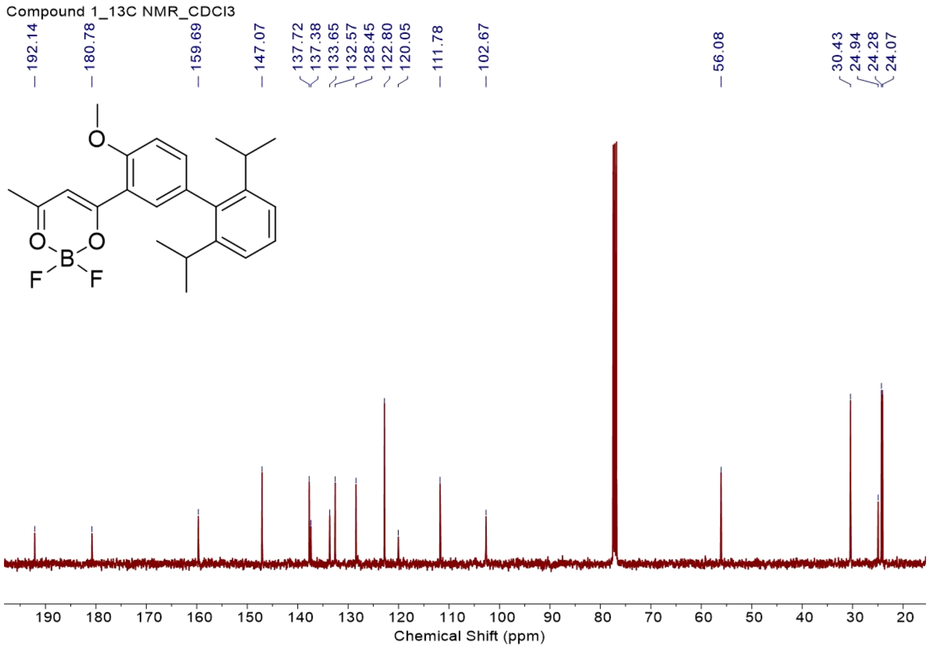

Figure S44. ^{13}C NMR spectra of compound **1**.

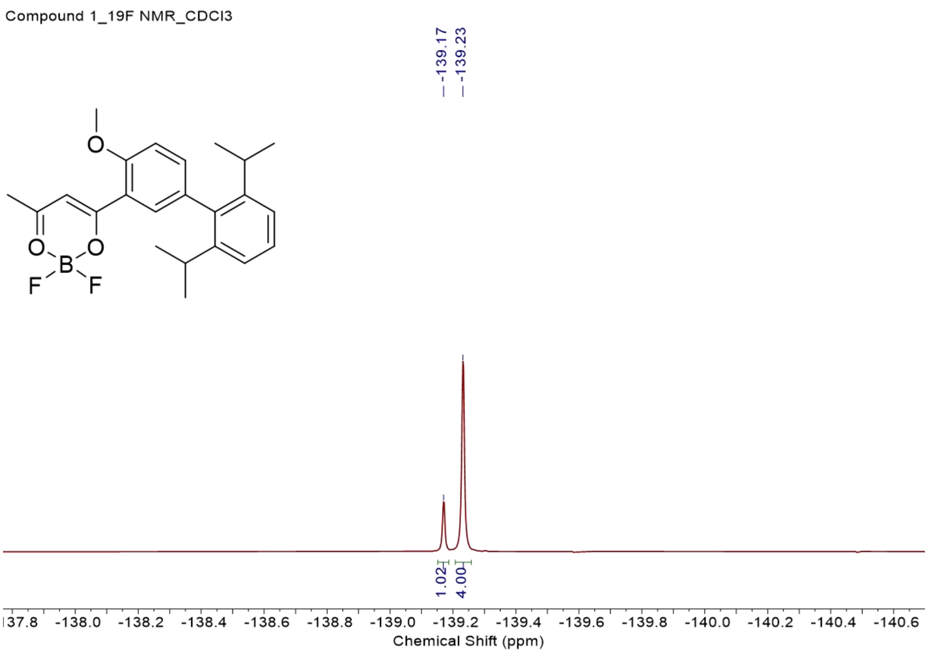

Figure S45. ^{19}F NMR spectra of compound **1**.

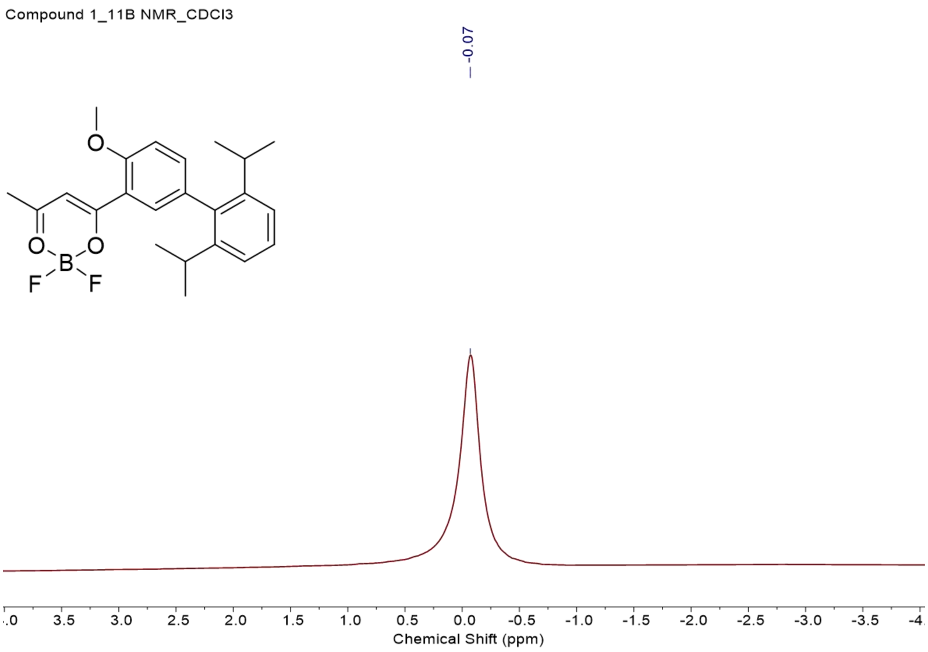

Figure S46. ^{11}B NMR spectra of compound **1**.

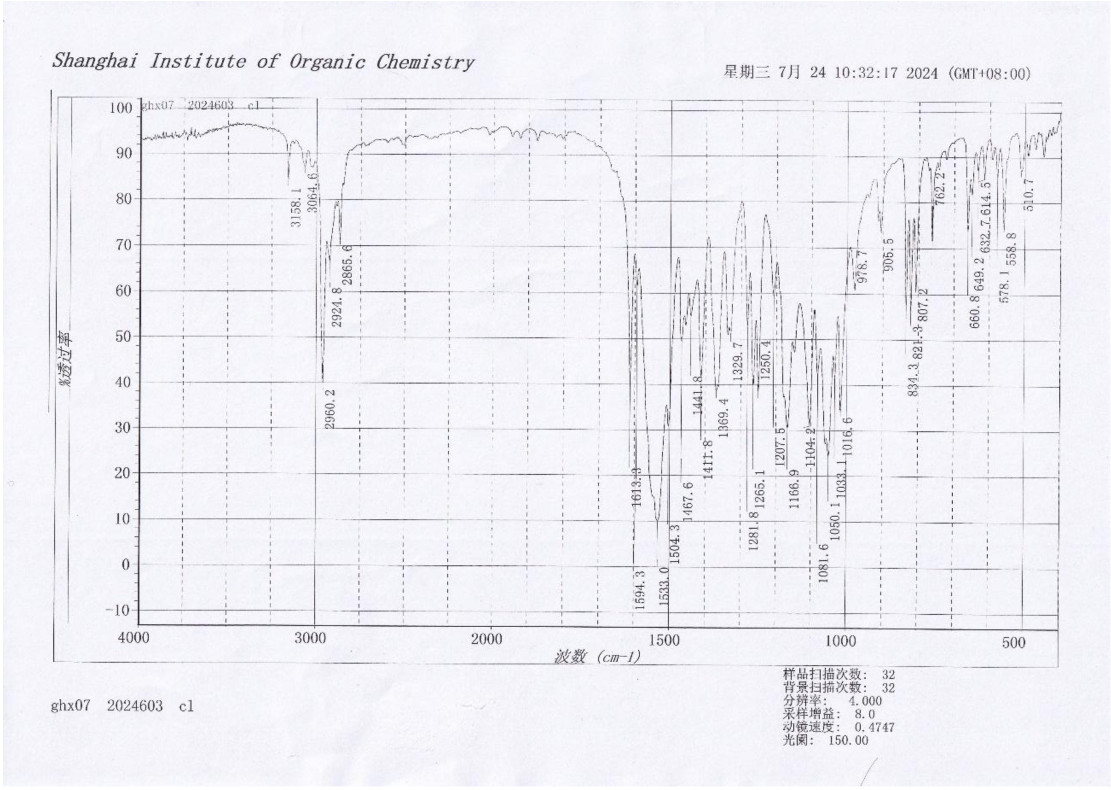

Figure S47. FT-IR spectrum of compound **1**.

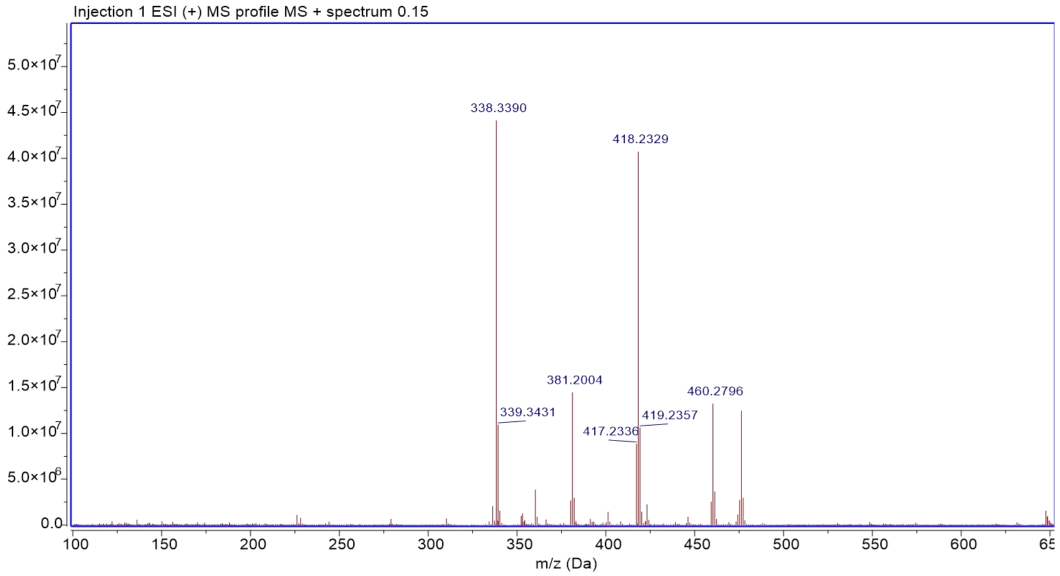

Figure S48. HRMS spectra of compound **1**. The additional signal peaks that are 42 units higher than the $[M+NH_4]^+$ peak can be assigned to $[M+NH_4]^+$ with acetonitrile.

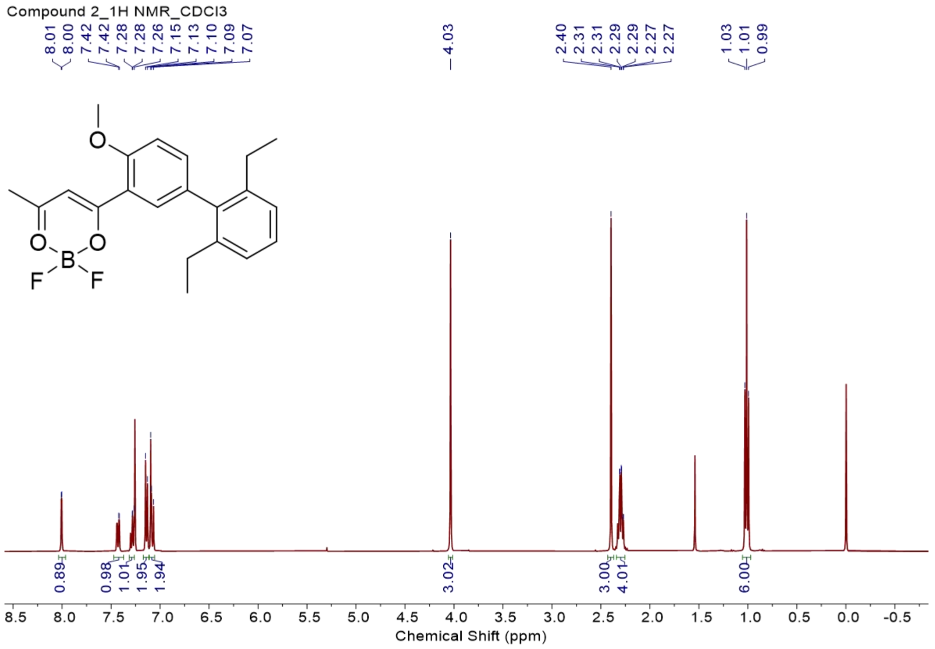

Figure S49. ^{1}H NMR spectra of compound **2**.

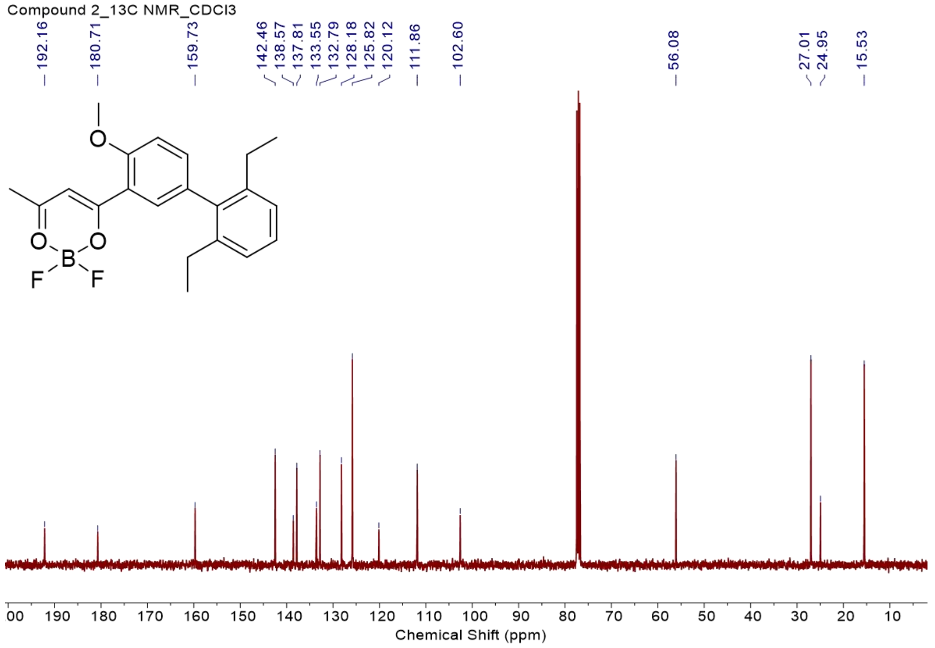

Figure S50. ^{13}C NMR spectra of compound **2**.

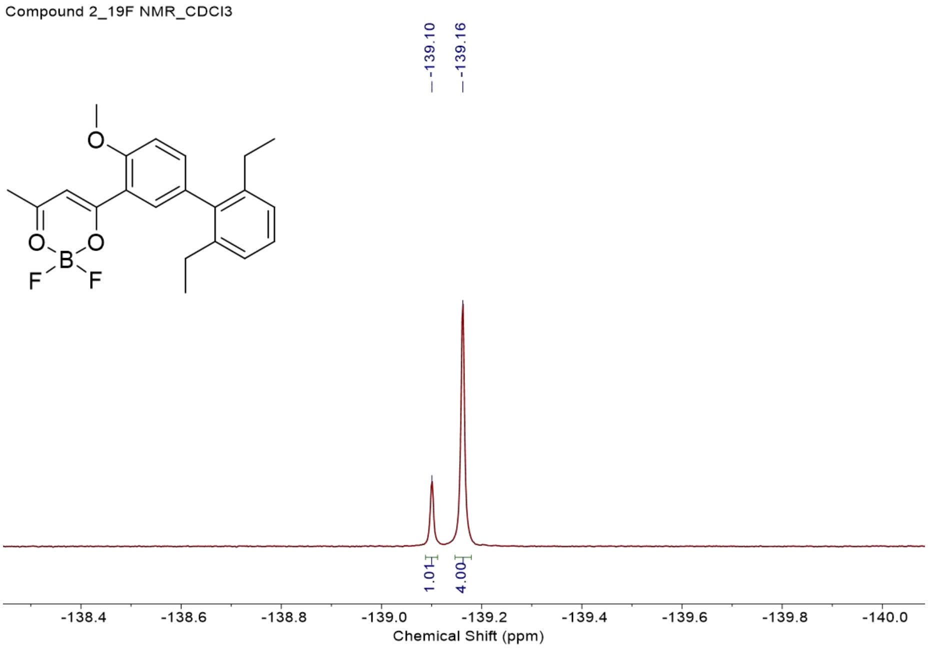

Figure S51. ^{19}F NMR spectra of compound **2**.

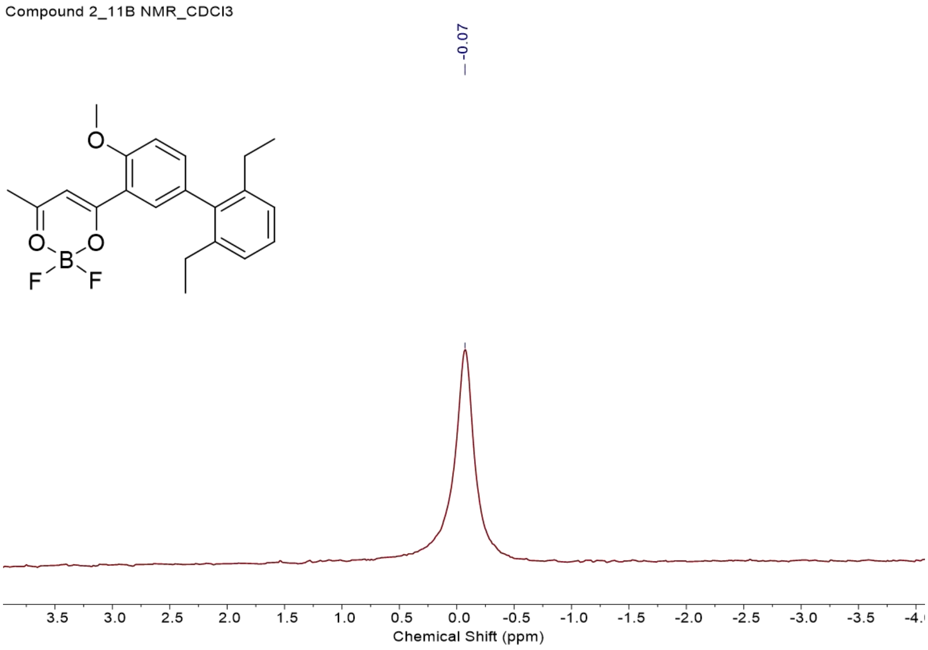

Figure S52. ^{11}B NMR spectra of compound **2**.

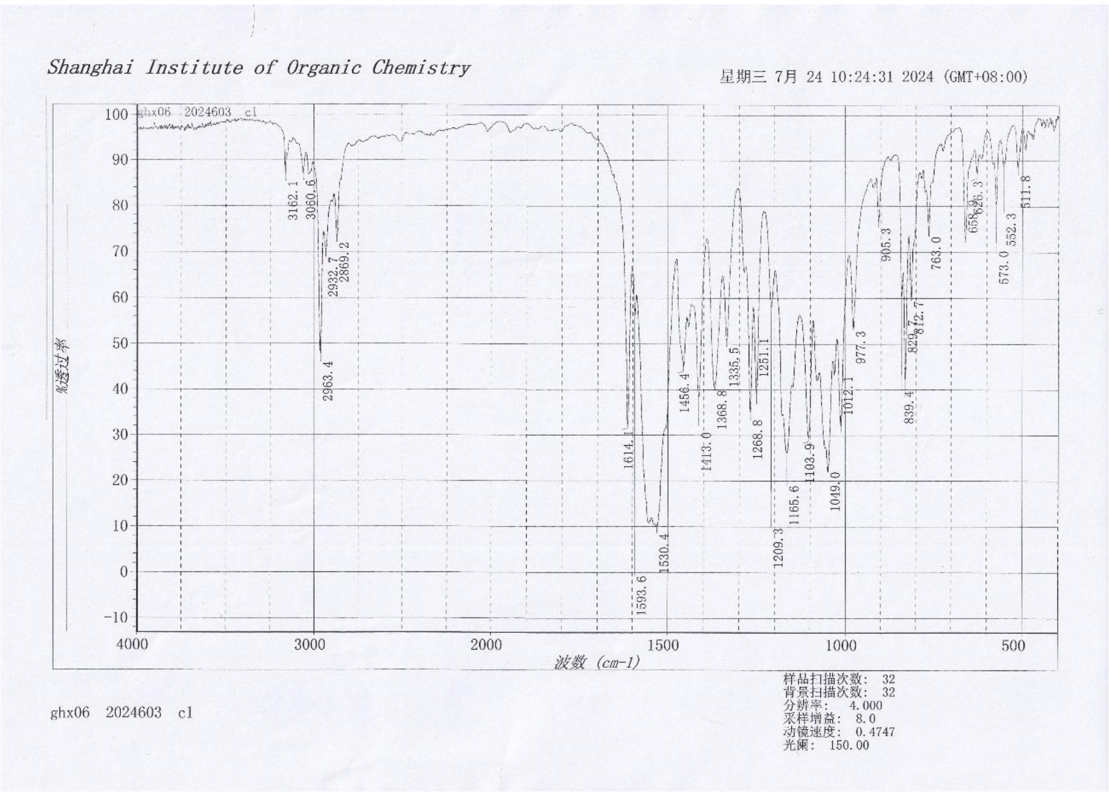

Figure S53. FT-IR spectrum of compound **2**.

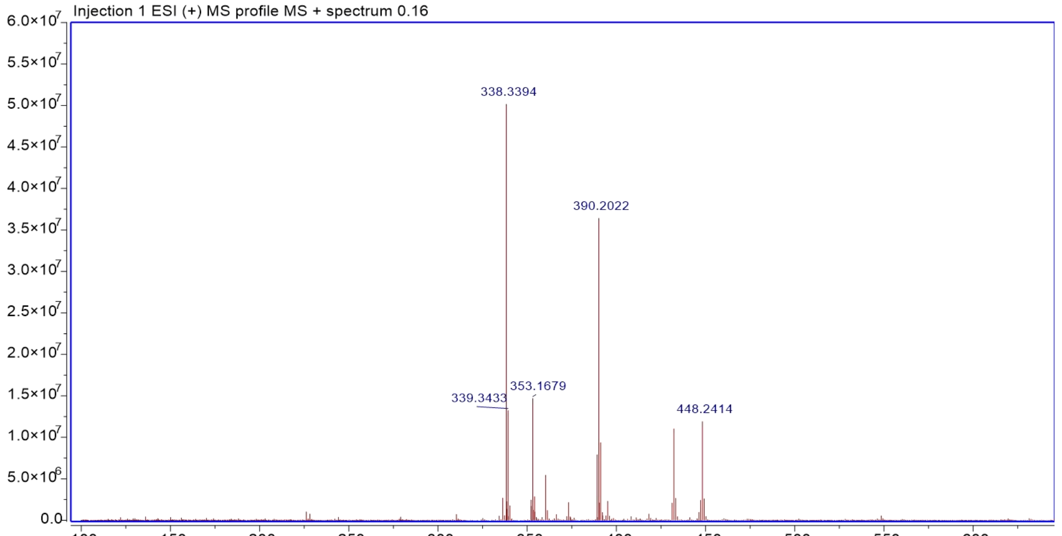

Figure S54. HRMS spectra of compound **2**. The additional signal peaks that 58 units higher than the $[M+NH_4]^+$ peak can be assigned to $[M+NH_4]^+$ with acetone.

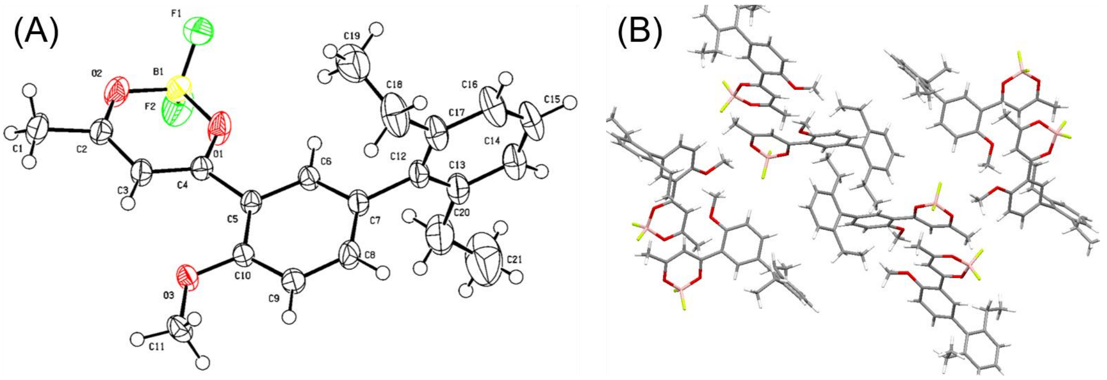

Figure S55. Single crystal structures of compound **2**: (A) monomer, (B) lattice structure; its depository number in CCDC is 2391572.

Table S6. Crystal data and structure refinement for compound **2**.

Identification code	mj24550_0m	
Empirical formula	C21 H23 B F2 O3	
Formula weight	372.20	
Temperature	170.00 K	
Wavelength	1.34139 Å	
Crystal system	Orthorhombic	
Space group	Pbca	
Unit cell dimensions	a = 8.22360(10) Å	a= 90°.
	b = 17.8594(3) Å	b= 90°.
	c = 26.4002(4) Å	g = 90°.
Volume	3877.36(10) Å^3	
Z	8	
Density (calculated)	1.275 Mg/m^3	
Absorption coefficient	0.507 mm^{-1}	
F(000)	1568	
Crystal size	0.17 x 0.17 x 0.05 mm^3	
Theta range for data collection	4.307 to 61.982°.	
Index ranges	-10<=h<=10, -17<=k<=23, -34<=l<=34	
Reflections collected	42212	
Independent reflections	4537 [R(int) = 0.0563]	
Completeness to theta = 53.594°	99.7 %	
Absorption correction	Semi-empirical from equivalents	
Max. and min. transmission	0.7521 and 0.6605	
Refinement method	Full-matrix least-squares on F^2	
Data / restraints / parameters	4537 / 0 / 248	

Goodness-of-fit on F^2	1.059
Final R indices [I>2sigma(I)]	R1 = 0.0536, wR2 = 0.1373
R indices (all data)	R1 = 0.0682, wR2 = 0.1485
Extinction coefficient	n/a
Largest diff. peak and hole	0.454 and -0.527 e.Å^{-3}

Table S7. Atomic coordinates (x 10^4) and equivalent isotropic displacement parameters (Å^{2}x 10^3) for compound **2**. U(eq) is defined as one third of the trace of the orthogonalized U^{ij} tensor.

	x	y	z	U(eq)
F(1)	7170(1)	3514(1)	3226(1)	54(1)
F(2)	4548(2)	3178(1)	3152(1)	58(1)
O(1)	5196(2)	4428(1)	3225(1)	46(1)
O(2)	5858(2)	3710(1)	2466(1)	45(1)
O(3)	2889(1)	6232(1)	2546(1)	31(1)
C(1)	5612(2)	4202(1)	1644(1)	46(1)
C(2)	5372(2)	4274(1)	2201(1)	33(1)
C(3)	4722(2)	4911(1)	2421(1)	33(1)
C(4)	4700(2)	4980(1)	2943(1)	26(1)
C(5)	4272(2)	5658(1)	3232(1)	26(1)
C(6)	4837(2)	5698(1)	3734(1)	28(1)
C(7)	4637(2)	6330(1)	4030(1)	33(1)
C(8)	3792(2)	6929(1)	3820(1)	43(1)
C(9)	3178(2)	6906(1)	3332(1)	38(1)
C(10)	3424(2)	6276(1)	3030(1)	27(1)
C(11)	2131(2)	6883(1)	2334(1)	37(1)
C(12)	5341(2)	6376(1)	4554(1)	34(1)
C(13)	4435(2)	6122(1)	4971(1)	41(1)
C(14)	5114(3)	6180(1)	5453(1)	47(1)
C(15)	6644(3)	6472(1)	5519(1)	54(1)
C(16)	7532(3)	6724(1)	5108(1)	58(1)
C(17)	6897(2)	6681(1)	4618(1)	44(1)
C(18)	7925(3)	6948(1)	4176(1)	63(1)
C(19)	8927(3)	6323(2)	3931(1)	74(1)

C(20)	2735(3)	5816(2)	4899(1)	72(1)
C(21)	1712(4)	5736(3)	5323(1)	124(2)
B(1)	5691(2)	3691(1)	3027(1)	33(1)

Table S8. Bond lengths [Å] and angles [°] for compound **2**.

F(1)-B(1)	1.363(2)
F(2)-B(1)	1.353(2)
O(1)-C(4)	1.3006(18)
O(1)-B(1)	1.4747(19)
O(2)-C(2)	1.289(2)
O(2)-B(1)	1.488(2)
O(3)-C(10)	1.3554(18)
O(3)-C(11)	1.4321(18)
C(1)-H(1A)	0.9800
C(1)-H(1B)	0.9800
C(1)-H(1C)	0.9800
C(1)-C(2)	1.491(2)
C(2)-C(3)	1.384(2)
C(3)-H(3)	0.9500
C(3)-C(4)	1.383(2)
C(4)-C(5)	1.4729(19)
C(5)-C(6)	1.405(2)
C(5)-C(10)	1.412(2)
C(6)-H(6)	0.9500
C(6)-C(7)	1.383(2)
C(7)-C(8)	1.392(2)
C(7)-C(12)	1.500(2)
C(8)-H(8)	0.9500
C(8)-C(9)	1.385(2)
C(9)-H(9)	0.9500
C(9)-C(10)	1.392(2)
C(11)-H(11A)	0.9800
C(11)-H(11B)	0.9800
C(11)-H(11C)	0.9800
C(12)-C(13)	1.404(3)
C(12)-C(17)	1.401(3)

C(13)-C(14)	1.394(2)
C(13)-C(20)	1.513(3)
C(14)-H(14)	0.9500
C(14)-C(15)	1.374(3)
C(15)-H(15)	0.9500
C(15)-C(16)	1.382(3)
C(16)-H(16)	0.9500
C(16)-C(17)	1.397(2)
C(17)-C(18)	1.517(3)
C(18)-H(18A)	0.9900
C(18)-H(18B)	0.9900
C(18)-C(19)	1.531(4)
C(19)-H(19A)	0.9800
C(19)-H(19B)	0.9800
C(19)-H(19C)	0.9800
C(20)-H(20A)	0.9900
C(20)-H(20B)	0.9900
C(20)-C(21)	1.408(4)
C(21)-H(21A)	0.9800
C(21)-H(21B)	0.9800
C(21)-H(21C)	0.9800

C(4)-O(1)-B(1)	124.14(13)
C(2)-O(2)-B(1)	121.85(12)
C(10)-O(3)-C(11)	117.53(11)
H(1A)-C(1)-H(1B)	109.5
H(1A)-C(1)-H(1C)	109.5
H(1B)-C(1)-H(1C)	109.5
C(2)-C(1)-H(1A)	109.5
C(2)-C(1)-H(1B)	109.5
C(2)-C(1)-H(1C)	109.5
O(2)-C(2)-C(1)	115.17(14)
O(2)-C(2)-C(3)	122.38(15)
C(3)-C(2)-C(1)	122.42(16)
C(2)-C(3)-H(3)	120.1
C(4)-C(3)-C(2)	119.73(14)
C(4)-C(3)-H(3)	120.1
O(1)-C(4)-C(3)	119.84(13)

O(1)-C(4)-C(5)	113.64(13)
C(3)-C(4)-C(5)	126.35(13)
C(6)-C(5)-C(4)	116.85(13)
C(6)-C(5)-C(10)	118.61(13)
C(10)-C(5)-C(4)	124.44(13)
C(5)-C(6)-H(6)	118.8
C(7)-C(6)-C(5)	122.41(13)
C(7)-C(6)-H(6)	118.8
C(6)-C(7)-C(8)	117.46(14)
C(6)-C(7)-C(12)	121.36(13)
C(8)-C(7)-C(12)	121.17(13)
C(7)-C(8)-H(8)	119.0
C(9)-C(8)-C(7)	122.00(14)
C(9)-C(8)-H(8)	119.0
C(8)-C(9)-H(9)	119.9
C(8)-C(9)-C(10)	120.27(14)
C(10)-C(9)-H(9)	119.9
O(3)-C(10)-C(5)	118.11(12)
O(3)-C(10)-C(9)	122.69(13)
C(9)-C(10)-C(5)	119.20(13)
O(3)-C(11)-H(11A)	109.5
O(3)-C(11)-H(11B)	109.5
O(3)-C(11)-H(11C)	109.5
H(11A)-C(11)-H(11B)	109.5
H(11A)-C(11)-H(11C)	109.5
H(11B)-C(11)-H(11C)	109.5
C(13)-C(12)-C(7)	119.93(15)
C(17)-C(12)-C(7)	119.08(15)
C(17)-C(12)-C(13)	120.99(15)
C(12)-C(13)-C(20)	120.62(16)
C(14)-C(13)-C(12)	118.65(18)
C(14)-C(13)-C(20)	120.71(18)
C(13)-C(14)-H(14)	119.6
C(15)-C(14)-C(13)	120.78(18)
C(15)-C(14)-H(14)	119.6
C(14)-C(15)-H(15)	119.8
C(14)-C(15)-C(16)	120.46(17)
C(16)-C(15)-H(15)	119.8

C(15)-C(16)-H(16)	119.6
C(15)-C(16)-C(17)	120.8(2)
C(17)-C(16)-H(16)	119.6
C(12)-C(17)-C(18)	122.51(16)
C(16)-C(17)-C(12)	118.34(18)
C(16)-C(17)-C(18)	119.13(19)
C(17)-C(18)-H(18A)	108.9
C(17)-C(18)-H(18B)	108.9
C(17)-C(18)-C(19)	113.3(2)
H(18A)-C(18)-H(18B)	107.7
C(19)-C(18)-H(18A)	108.9
C(19)-C(18)-H(18B)	108.9
C(18)-C(19)-H(19A)	109.5
C(18)-C(19)-H(19B)	109.5
C(18)-C(19)-H(19C)	109.5
H(19A)-C(19)-H(19B)	109.5
H(19A)-C(19)-H(19C)	109.5
H(19B)-C(19)-H(19C)	109.5
C(13)-C(20)-H(20A)	107.5
C(13)-C(20)-H(20B)	107.5
H(20A)-C(20)-H(20B)	107.0
C(21)-C(20)-C(13)	119.3(2)
C(21)-C(20)-H(20A)	107.5
C(21)-C(20)-H(20B)	107.5
C(20)-C(21)-H(21A)	109.5
C(20)-C(21)-H(21B)	109.5
C(20)-C(21)-H(21C)	109.5
H(21A)-C(21)-H(21B)	109.5
H(21A)-C(21)-H(21C)	109.5
H(21B)-C(21)-H(21C)	109.5
F(1)-B(1)-O(1)	108.41(14)
F(1)-B(1)-O(2)	107.99(15)
F(2)-B(1)-F(1)	111.66(15)
F(2)-B(1)-O(1)	109.06(15)
F(2)-B(1)-O(2)	108.77(14)
O(1)-B(1)-O(2)	110.96(13)

Symmetry transformations used to generate equivalent atoms:

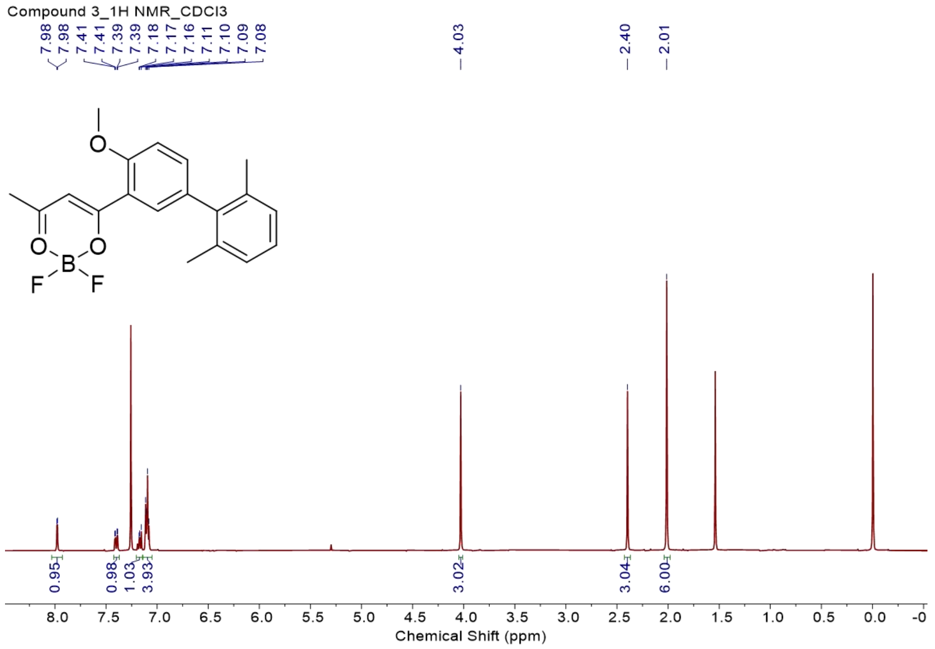

Figure S56. ^{1}H NMR spectra of compound **3**.

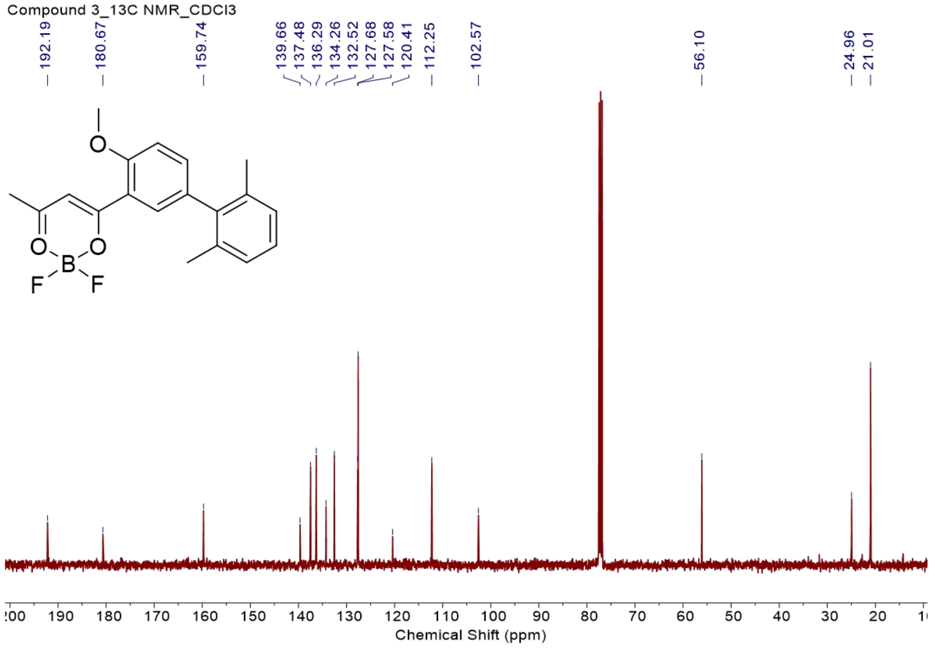

Figure S57. ^{13}C NMR spectra of compound **3**.

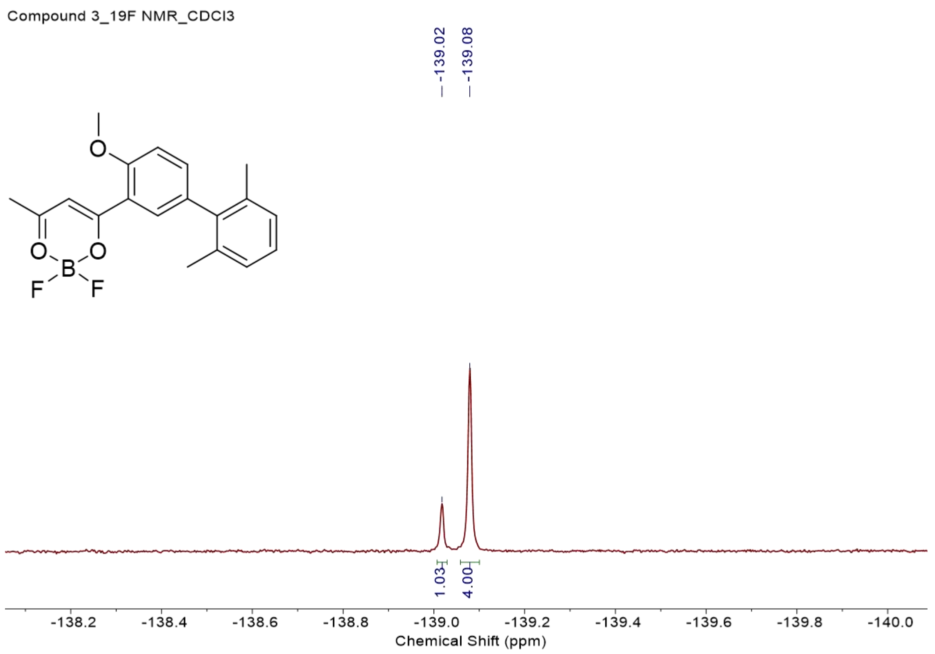

Figure S58. ^{19}F NMR spectra of compound **3**.

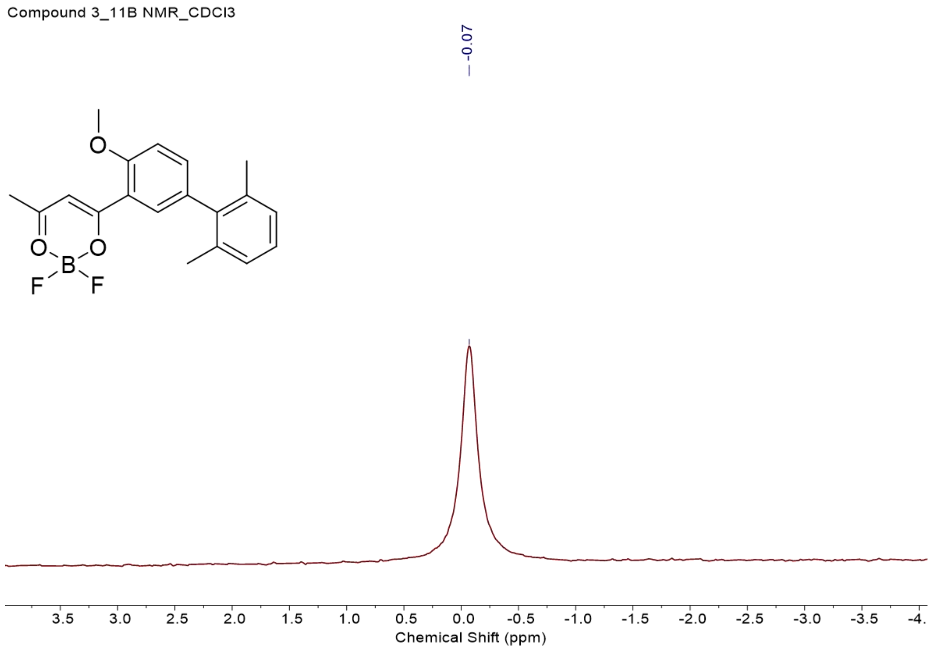

Figure S59. ^{11}B NMR spectra of compound **3**.

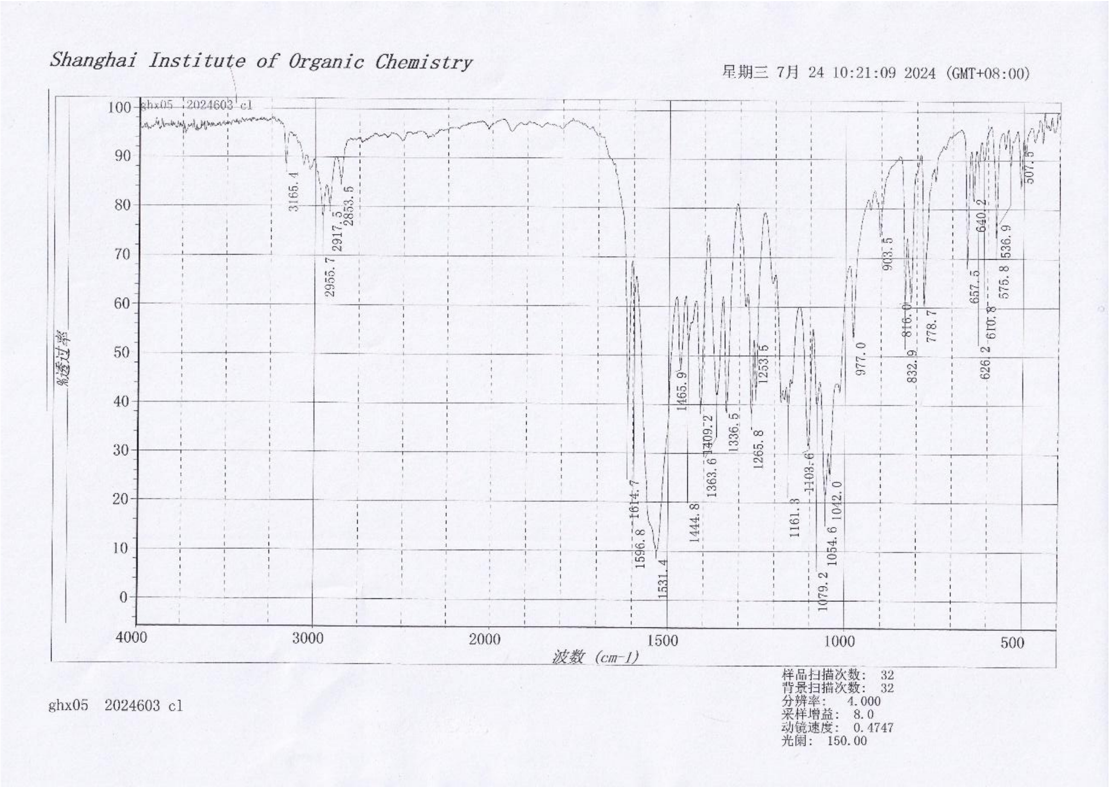

Figure S60. FT-IR spectrum of compound **3**.

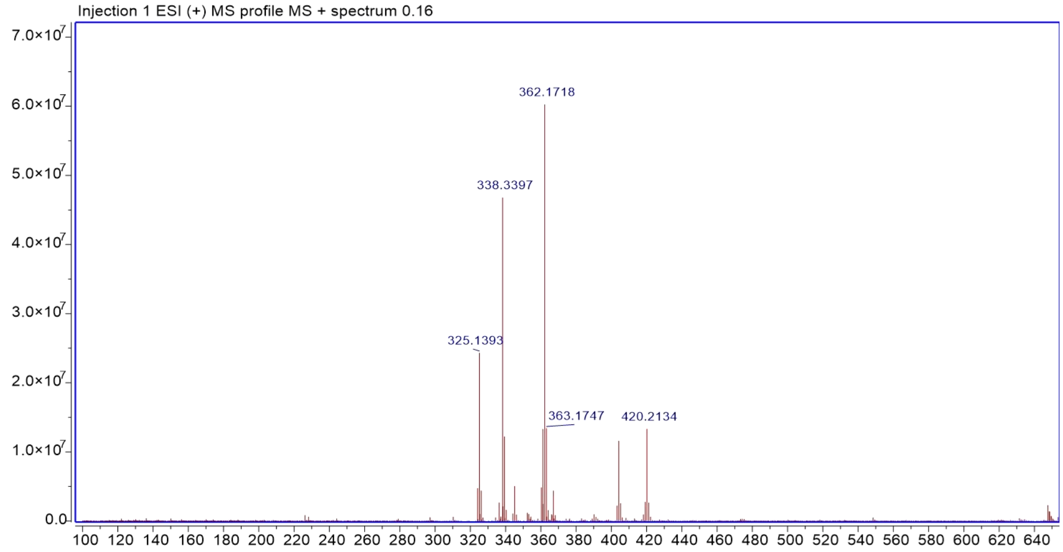

Figure S61. HRMS spectra of compound **3**. The additional signal peaks that 58 units higher than the $[M+NH_4]^+$ peak can be assigned to $[M+NH_4]^+$ with acetone.

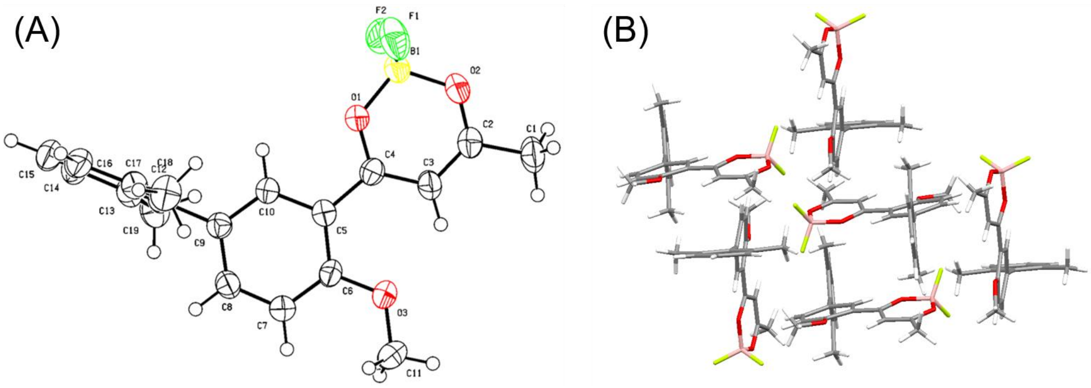

Figure S62. Single crystal structures of compound **3**: (A) monomer, (B) lattice structure; its depository number in CCDC is 2391573.

Table S9. Crystal data and structure refinement for compound **3**.

Identification code	mj24519_0m	
Empirical formula	C19 H19 B F2 O3	
Formula weight	344.15	
Temperature	170.00 K	
Wavelength	1.34139 Å	
Crystal system	Monoclinic	
Space group	P 1 21/c 1	
Unit cell dimensions	a = 10.5752(3) Å	a= 90°.
	b = 10.0203(3) Å	b= 90.864(2)°.
	c = 16.1825(4) Å	g = 90°.
Volume	1714.61(8) Å^3	
Z	4	
Density (calculated)	1.333 Mg/m^3	
Absorption coefficient	0.546 mm^{-1}	
F(000)	720	
Crystal size	0.17 x 0.17 x 0.05 mm^3	
Theta range for data collection	3.637 to 55.099°.	
Index ranges	-12<=h<=12, -12<=k<=12, -19<=l<=18	
Reflections collected	13668	
Independent reflections	3261 [R(int) = 0.0882]	
Completeness to theta = 53.594°	99.8 %	
Absorption correction	Semi-empirical from equivalents	
Max. and min. transmission	0.7508 and 0.3595	
Refinement method	Full-matrix least-squares on F^2	
Data / restraints / parameters	3261 / 0 / 231	

Goodness-of-fit on F^2	1.000
Final R indices [I>2sigma(I)]	R1 = 0.0811, wR2 = 0.2009
R indices (all data)	R1 = 0.1267, wR2 = 0.2405
Extinction coefficient	0.010(2)
Largest diff. peak and hole	0.302 and -0.538 e.Å^{-3}

Table S10. Atomic coordinates (x 10^4) and equivalent isotropic displacement parameters (Å^{2}x 10^3) for compound **3**. U(eq) is defined as one third of the trace of the orthogonalized U^{ij} tensor.

	x	y	z	U(eq)
F(2)	1640(2)	769(2)	3649(1)	67(1)
F(1)	304(2)	2392(2)	4035(1)	73(1)
O(3)	2288(2)	4912(2)	931(1)	51(1)
O(1)	1912(2)	2881(2)	3139(1)	58(1)
O(2)	110(2)	1493(2)	2725(1)	56(1)
C(4)	1979(3)	3334(3)	2389(2)	37(1)
C(5)	3043(3)	4247(3)	2247(2)	36(1)
C(12)	5979(3)	5311(3)	3499(2)	38(1)
C(10)	3964(3)	4384(3)	2882(2)	37(1)
C(6)	3225(3)	4961(3)	1513(2)	39(1)
C(9)	5042(3)	5154(3)	2794(2)	37(1)
C(17)	5746(3)	6250(3)	4118(2)	43(1)
C(8)	5221(3)	5775(3)	2044(2)	42(1)
C(2)	204(3)	1995(3)	1994(2)	45(1)
C(13)	7094(3)	4546(3)	3519(2)	44(1)
C(7)	4326(3)	5700(3)	1411(2)	45(1)
C(14)	7936(3)	4728(3)	4183(2)	51(1)
C(16)	6637(3)	6418(3)	4753(2)	49(1)
C(19)	7387(3)	3571(3)	2850(2)	55(1)
C(15)	7718(3)	5662(3)	4777(2)	54(1)
C(3)	1111(3)	2907(3)	1802(2)	54(1)
C(18)	4561(3)	7088(4)	4107(2)	56(1)
C(11)	2356(4)	5767(4)	231(2)	61(1)
C(1)	-752(3)	1497(4)	1383(2)	61(1)

B(1)	992(3)	1867(4)	3409(2)	45(1)

Table S11. Bond lengths [Å] and angles [°] for compound **3**.

F(2)-B(1)	1.350(4)
F(1)-B(1)	1.362(4)
O(3)-C(6)	1.359(3)
O(3)-C(11)	1.423(4)
O(1)-C(4)	1.298(3)
O(1)-B(1)	1.477(4)
O(2)-C(2)	1.291(4)
O(2)-B(1)	1.485(4)
C(4)-C(5)	1.471(4)
C(4)-C(3)	1.379(4)
C(5)-C(10)	1.411(4)
C(5)-C(6)	1.403(4)
C(12)-C(9)	1.507(4)
C(12)-C(17)	1.400(4)
C(12)-C(13)	1.406(4)
C(10)-H(10)	0.9500
C(10)-C(9)	1.386(4)
C(6)-C(7)	1.391(4)
C(9)-C(8)	1.380(4)
C(17)-C(16)	1.393(4)
C(17)-C(18)	1.509(4)
C(8)-H(8)	0.9500
C(8)-C(7)	1.386(4)
C(2)-C(3)	1.364(4)
C(2)-C(1)	1.489(4)
C(13)-C(14)	1.397(4)
C(13)-C(19)	1.493(4)
C(7)-H(7)	0.9500
C(14)-H(14)	0.9500
C(14)-C(15)	1.364(5)
C(16)-H(16)	0.9500
C(16)-C(15)	1.372(5)
C(19)-H(19A)	0.9800

C(19)-H(19B)	0.9800
C(19)-H(19C)	0.9800
C(15)-H(15)	0.9500
C(3)-H(3)	0.9500
C(18)-H(18A)	0.9800
C(18)-H(18B)	0.9800
C(18)-H(18C)	0.9800
C(11)-H(11A)	0.9800
C(11)-H(11B)	0.9800
C(11)-H(11C)	0.9800
C(1)-H(1A)	0.9800
C(1)-H(1B)	0.9800
C(1)-H(1C)	0.9800

C(6)-O(3)-C(11)	119.0(2)
C(4)-O(1)-B(1)	124.2(2)
C(2)-O(2)-B(1)	121.9(2)
O(1)-C(4)-C(5)	114.5(2)
O(1)-C(4)-C(3)	119.4(3)
C(3)-C(4)-C(5)	126.1(3)
C(10)-C(5)-C(4)	117.9(2)
C(6)-C(5)-C(4)	124.3(2)
C(6)-C(5)-C(10)	117.7(3)
C(17)-C(12)-C(9)	119.4(3)
C(17)-C(12)-C(13)	120.4(3)
C(13)-C(12)-C(9)	120.1(3)
C(5)-C(10)-H(10)	118.7
C(9)-C(10)-C(5)	122.6(3)
C(9)-C(10)-H(10)	118.7
O(3)-C(6)-C(5)	117.5(3)
O(3)-C(6)-C(7)	122.7(3)
C(7)-C(6)-C(5)	119.8(3)
C(10)-C(9)-C(12)	120.8(3)
C(8)-C(9)-C(12)	121.4(3)
C(8)-C(9)-C(10)	117.8(3)
C(12)-C(17)-C(18)	121.4(3)
C(16)-C(17)-C(12)	119.0(3)
C(16)-C(17)-C(18)	119.6(3)

C(9)-C(8)-H(8)	119.2
C(9)-C(8)-C(7)	121.6(3)
C(7)-C(8)-H(8)	119.2
O(2)-C(2)-C(3)	122.3(3)
O(2)-C(2)-C(1)	114.6(3)
C(3)-C(2)-C(1)	123.1(3)
C(12)-C(13)-C(19)	121.5(3)
C(14)-C(13)-C(12)	118.1(3)
C(14)-C(13)-C(19)	120.3(3)
C(6)-C(7)-H(7)	119.8
C(8)-C(7)-C(6)	120.3(3)
C(8)-C(7)-H(7)	119.8
C(13)-C(14)-H(14)	119.4
C(15)-C(14)-C(13)	121.3(3)
C(15)-C(14)-H(14)	119.4
C(17)-C(16)-H(16)	119.7
C(15)-C(16)-C(17)	120.5(3)
C(15)-C(16)-H(16)	119.7
C(13)-C(19)-H(19A)	109.5
C(13)-C(19)-H(19B)	109.5
C(13)-C(19)-H(19C)	109.5
H(19A)-C(19)-H(19B)	109.5
H(19A)-C(19)-H(19C)	109.5
H(19B)-C(19)-H(19C)	109.5
C(14)-C(15)-C(16)	120.6(3)
C(14)-C(15)-H(15)	119.7
C(16)-C(15)-H(15)	119.7
C(4)-C(3)-H(3)	119.5
C(2)-C(3)-C(4)	120.9(3)
C(2)-C(3)-H(3)	119.5
C(17)-C(18)-H(18A)	109.5
C(17)-C(18)-H(18B)	109.5
C(17)-C(18)-H(18C)	109.5
H(18A)-C(18)-H(18B)	109.5
H(18A)-C(18)-H(18C)	109.5
H(18B)-C(18)-H(18C)	109.5
O(3)-C(11)-H(11A)	109.5
O(3)-C(11)-H(11B)	109.5

O(3)-C(11)-H(11C)	109.5
H(11A)-C(11)-H(11B)	109.5
H(11A)-C(11)-H(11C)	109.5
H(11B)-C(11)-H(11C)	109.5
C(2)-C(1)-H(1A)	109.5
C(2)-C(1)-H(1B)	109.5
C(2)-C(1)-H(1C)	109.5
H(1A)-C(1)-H(1B)	109.5
H(1A)-C(1)-H(1C)	109.5
H(1B)-C(1)-H(1C)	109.5
F(2)-B(1)-F(1)	112.0(3)
F(2)-B(1)-O(1)	108.2(3)
F(2)-B(1)-O(2)	108.6(3)
F(1)-B(1)-O(1)	108.5(3)
F(1)-B(1)-O(2)	108.3(3)
O(1)-B(1)-O(2)	111.2(3)

Symmetry transformations used to generate equivalent atoms:

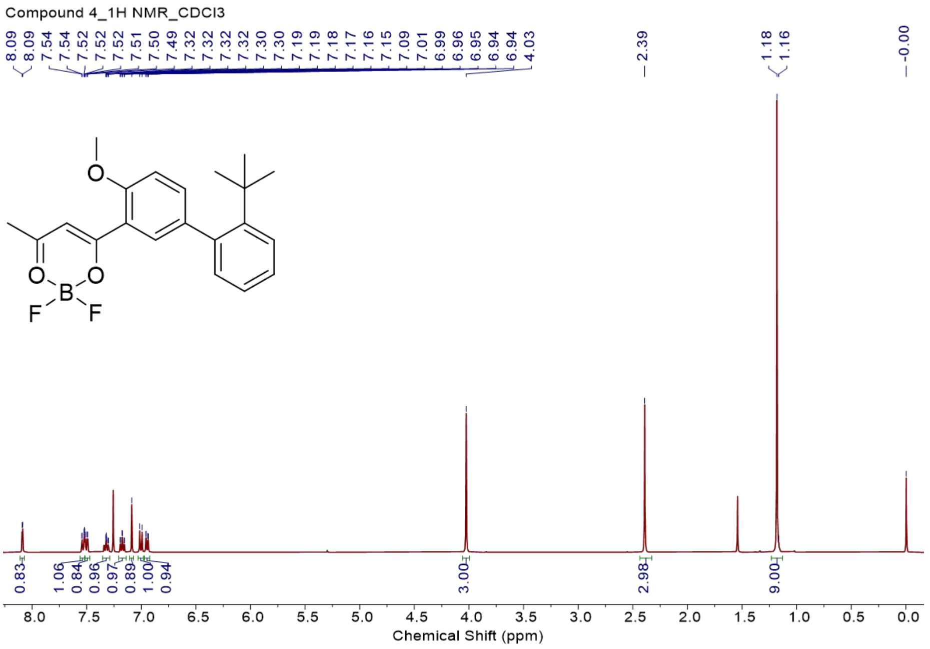

Figure S63. ^{1}H NMR spectra of compound **4**.

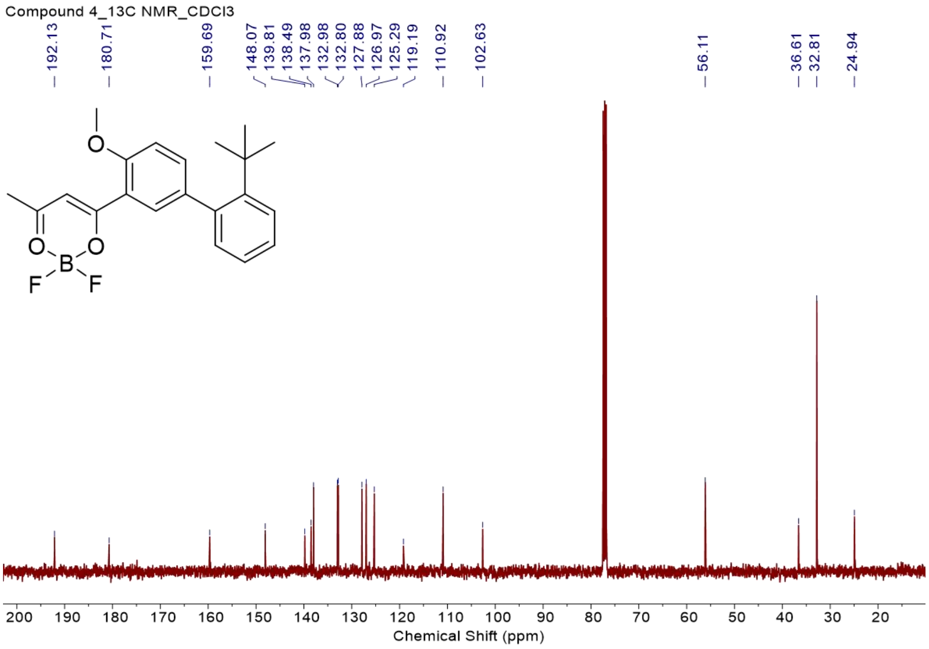

Figure S64. ^{13}C NMR spectra of compound **4**.

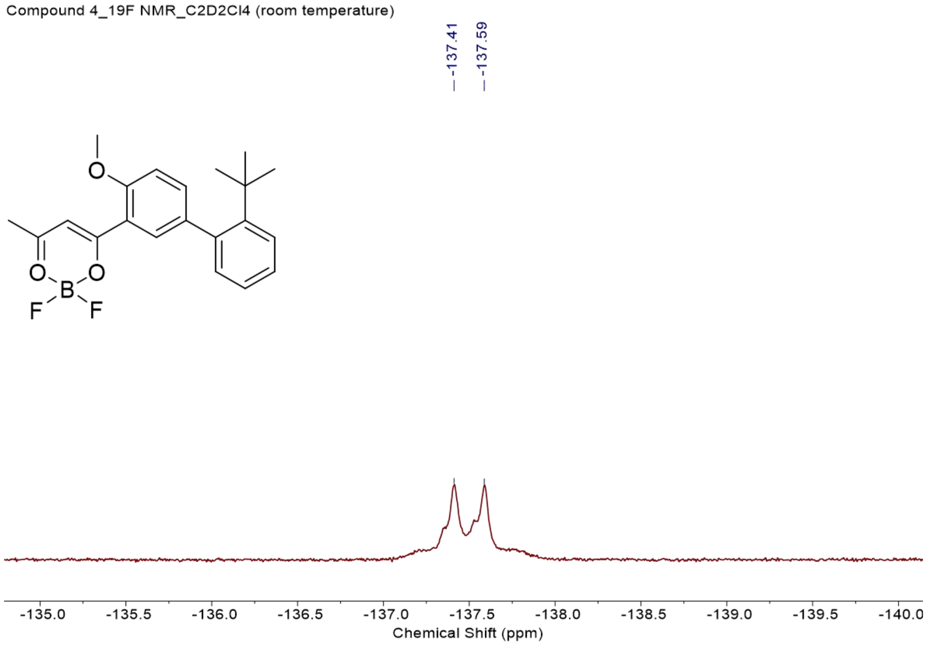

Figure S65. ^{19}F NMR spectra of compound **4** at room temperature. This specific ^{19}F NMR pattern should be induced by the conformation of **4**, rather than impurity.

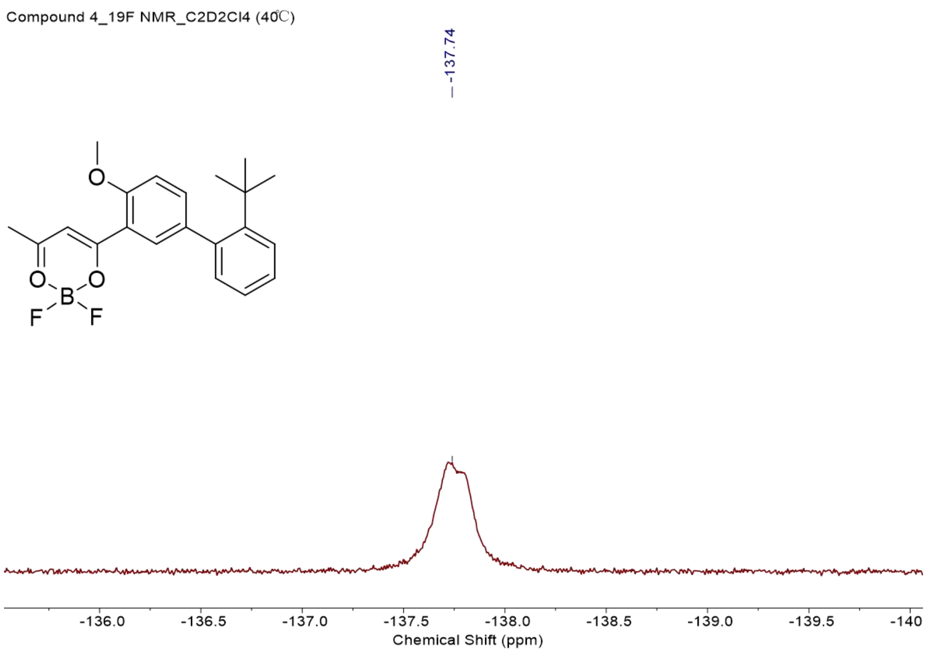

Figure S66. ^{19}F NMR spectra of compound **4** at 40 °C.

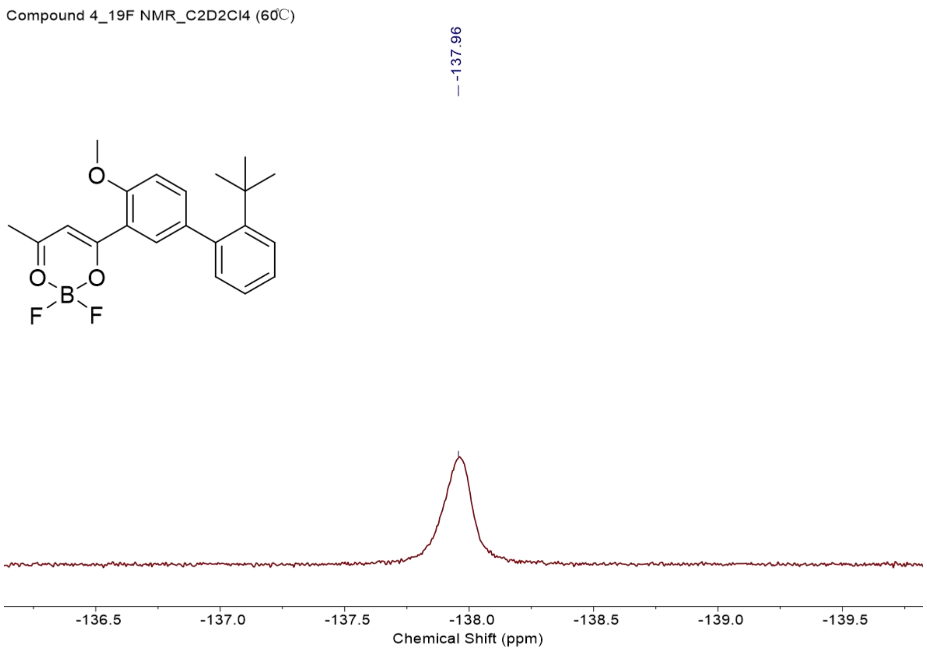

Figure S67. ^{19}F NMR spectra of compound **4** at 60 °C.

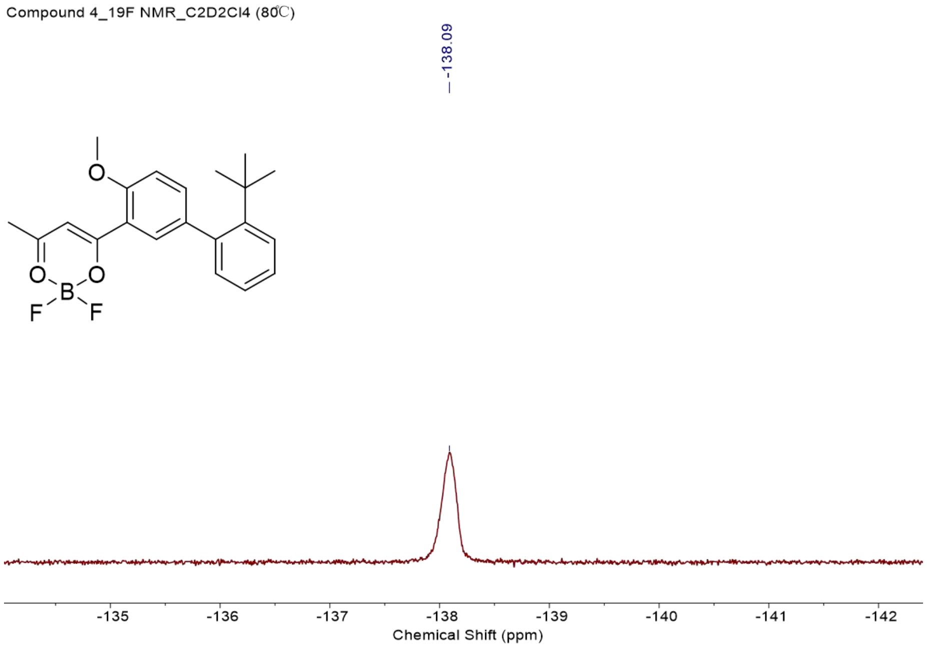

Figure S68. ^{19}F NMR spectra of compound **4** at 80 °C.

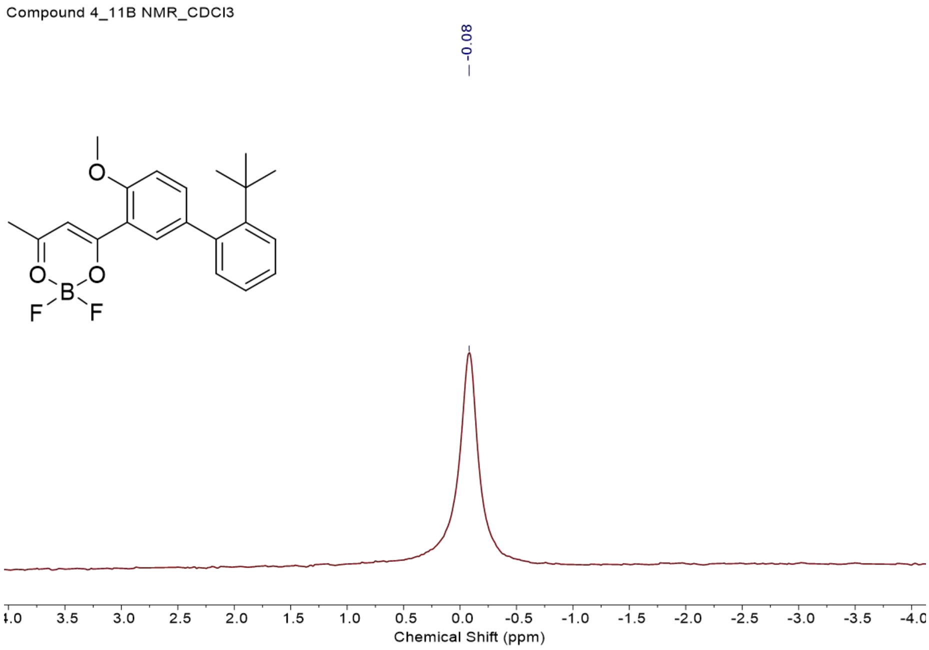

Figure S69. ^{11}B NMR spectra of compound **4**.

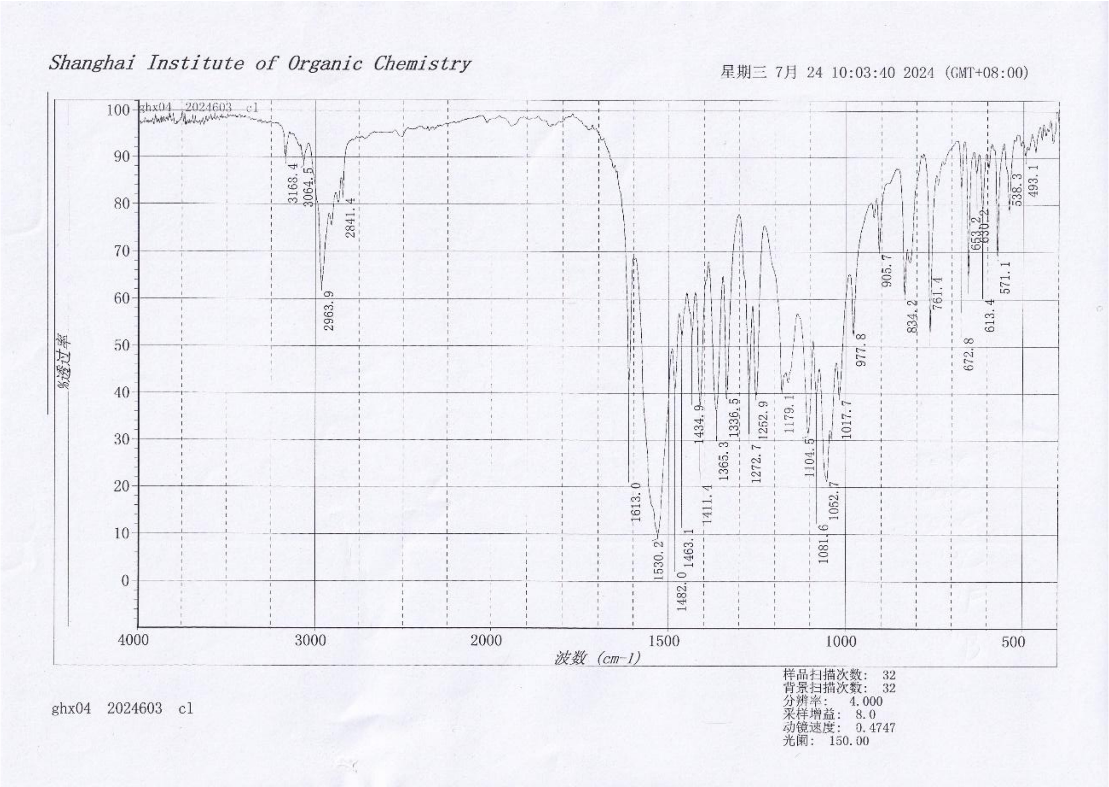

Figure S70. FT-IR spectrum of compound **4**.

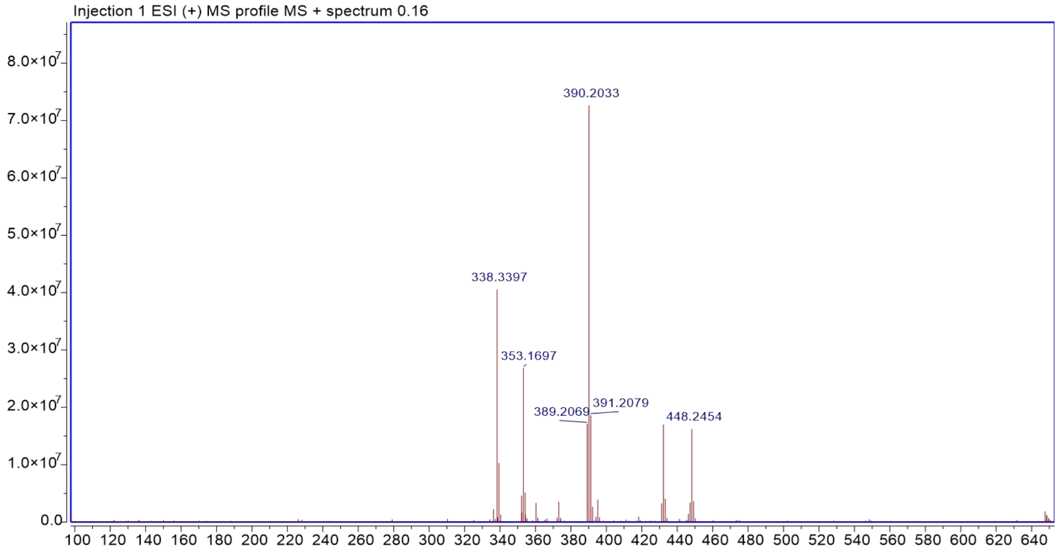

Figure S71. HRMS spectra of compound **4**. The additional signal peaks that 58 units higher than the $[M+NH_4]^+$ peak can be assigned to $[M+NH_4]^+$ with acetone.

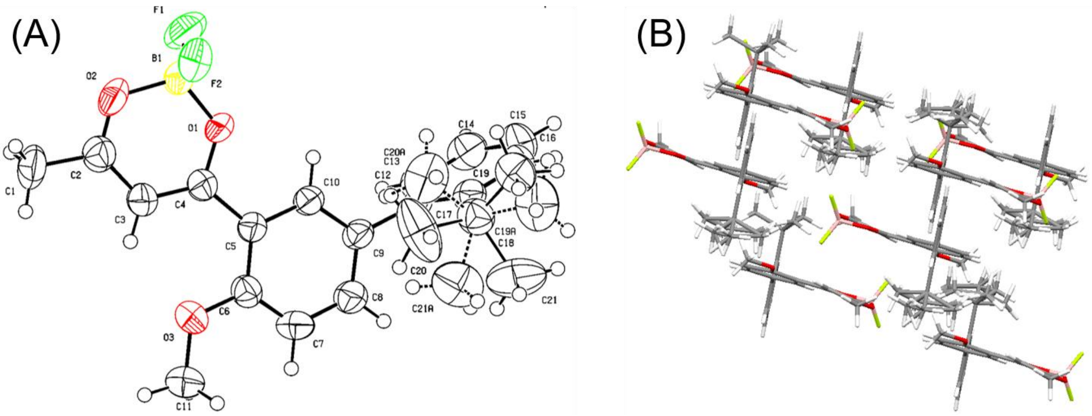

Figure S72. Single crystal structures of compound **4**: (A) monomer, (B) lattice structure; its depository number in CCDC is 2391574.

Table S12. Crystal data and structure refinement for compound **4**.

Identification code	mj24828_0m	
Empirical formula	C21 H23 B F2 O3	
Formula weight	372.20	
Temperature	170.00 K	
Wavelength	1.34139 Å	
Crystal system	Triclinic	
Space group	P-1	
Unit cell dimensions	a = 8.9896(12) Å	a= 99.285(5)°.
	b = 9.9871(13) Å	b= 90.106(5)°.
	c = 12.0187(16) Å	g = 114.799(5)°.
Volume	963.8(2) $Å^3$	
Z	2	
Density (calculated)	1.283 Mg/m^3	
Absorption coefficient	0.510 mm^{-1}	
F(000)	392	
Crystal size	0.17 x 0.17 x 0.05 mm^3	
Theta range for data collection	3.251 to 54.834°.	
Index ranges	-10<=h<=10, -11<=k<=12, -14<=l<=14	
Reflections collected	11469	
Independent reflections	3624 [R(int) = 0.0882]	
Completeness to theta = 53.594°	99.5 %	
Absorption correction	Semi-empirical from equivalents	
Max. and min. transmission	0.7508 and 0.4749	
Refinement method	Full-matrix least-squares on F^2	

Data / restraints / parameters	3624 / 75 / 280
Goodness-of-fit on F^2	1.026
Final R indices [I>2sigma(I)]	R1 = 0.0943, wR2 = 0.2401
R indices (all data)	R1 = 0.1615, wR2 = 0.2869
Extinction coefficient	n/a
Largest diff. peak and hole	0.410 and -0.296 e.Å^{-3}

Table S13. Atomic coordinates (x 10^4) and equivalent isotropic displacement parameters (Å^{2}x 10^3) for compound **4**. U(eq) is defined as one third of the trace of the orthogonalized U^{ij} tensor.

	x	y	z	U(eq)
F(1)	5553(5)	9776(3)	3446(3)	115(1)
F(2)	8216(5)	10435(4)	3051(3)	108(1)
O(1)	6717(5)	8161(4)	3608(3)	97(1)
O(2)	7575(5)	10537(4)	4920(3)	87(1)
O(3)	8230(4)	5882(3)	5656(2)	65(1)
C(1)	8698(7)	10924(5)	6766(4)	78(2)
C(2)	8073(6)	9958(5)	5629(4)	61(1)
C(3)	8008(6)	8535(5)	5365(4)	59(1)
C(4)	7228(5)	7614(4)	4363(3)	52(1)
C(5)	6834(5)	6001(4)	4017(3)	48(1)
C(6)	7256(5)	5137(5)	4664(3)	51(1)
C(7)	6732(5)	3622(5)	4281(4)	61(1)
C(8)	5813(5)	2940(5)	3250(4)	58(1)
C(9)	5412(5)	3765(4)	2581(3)	49(1)
C(10)	5906(5)	5269(4)	2981(3)	49(1)
C(11)	8633(7)	5012(6)	6330(4)	78(2)
C(12)	4361(5)	3013(4)	1486(4)	48(1)
C(13)	2678(6)	2561(5)	1596(4)	63(1)
C(14)	1520(6)	1874(5)	682(5)	73(1)
C(15)	2060(6)	1599(6)	-369(5)	77(2)
C(16)	3709(6)	2033(5)	-491(4)	69(1)
C(17)	4913(5)	2739(4)	415(4)	54(1)
C(18)	6732(6)	3168(5)	168(4)	68(1)

C(19)	7091(12)	3601(11)	-1004(7)	77(3)
C(19A)	6930(30)	2690(30)	-1031(14)	109(6)
C(20)	8064(11)	4338(16)	993(8)	121(4)
C(20A)	7540(20)	4919(14)	459(19)	89(5)
C(21)	7013(13)	1675(12)	38(13)	116(3)
C(21A)	7590(20)	2630(30)	977(17)	101(5)
B(1)	7011(11)	9731(7)	3733(6)	87(2)

Table S14. Bond lengths [Å] and angles [°] for compound **4**.

F(1)-B(1)	1.375(7)
F(2)-B(1)	1.374(9)
O(1)-C(4)	1.304(5)
O(1)-B(1)	1.459(7)
O(2)-C(2)	1.279(5)
O(2)-B(1)	1.492(7)
O(3)-C(6)	1.380(5)
O(3)-C(11)	1.425(5)
C(1)-H(1A)	0.9800
C(1)-H(1B)	0.9800
C(1)-H(1C)	0.9800
C(1)-C(2)	1.493(6)
C(2)-C(3)	1.381(6)
C(3)-H(3)	0.9500
C(3)-C(4)	1.374(6)
C(4)-C(5)	1.483(5)
C(5)-C(6)	1.408(5)
C(5)-C(10)	1.404(5)
C(6)-C(7)	1.378(6)
C(7)-H(7)	0.9500
C(7)-C(8)	1.391(6)
C(8)-H(8)	0.9500
C(8)-C(9)	1.384(5)
C(9)-C(10)	1.376(5)
C(9)-C(12)	1.507(5)
C(10)-H(10)	0.9500
C(11)-H(11A)	0.9800
C(11)-H(11B)	0.9800

C(11)-H(11C)	0.9800
C(12)-C(13)	1.399(6)
C(12)-C(17)	1.407(6)
C(13)-H(13)	0.9500
C(13)-C(14)	1.384(6)
C(14)-H(14)	0.9500
C(14)-C(15)	1.381(7)
C(15)-H(15)	0.9500
C(15)-C(16)	1.374(7)
C(16)-H(16)	0.9500
C(16)-C(17)	1.401(6)
C(17)-C(18)	1.550(6)
C(18)-C(19)	1.535(9)
C(18)-C(19A)	1.477(15)
C(18)-C(20)	1.493(9)
C(18)-C(20A)	1.563(13)
C(18)-C(21)	1.597(10)
C(18)-C(21A)	1.528(14)
C(19)-H(19A)	0.9800
C(19)-H(19B)	0.9800
C(19)-H(19C)	0.9800
C(19A)-H(19D)	0.9800
C(19A)-H(19E)	0.9800
C(19A)-H(19F)	0.9800
C(20)-H(20A)	0.9800
C(20)-H(20B)	0.9800
C(20)-H(20C)	0.9800
C(20A)-H(20D)	0.9800
C(20A)-H(20E)	0.9800
C(20A)-H(20F)	0.9800
C(21)-H(21A)	0.9800
C(21)-H(21B)	0.9800
C(21)-H(21C)	0.9800
C(21A)-H(21D)	0.9800
C(21A)-H(21E)	0.9800
C(21A)-H(21F)	0.9800
C(4)-O(1)-B(1)	123.8(4)
C(2)-O(2)-B(1)	120.5(4)

C(6)-O(3)-C(11)	117.5(3)
H(1A)-C(1)-H(1B)	109.5
H(1A)-C(1)-H(1C)	109.5
H(1B)-C(1)-H(1C)	109.5
C(2)-C(1)-H(1A)	109.5
C(2)-C(1)-H(1B)	109.5
C(2)-C(1)-H(1C)	109.5
O(2)-C(2)-C(1)	114.9(4)
O(2)-C(2)-C(3)	122.3(4)
C(3)-C(2)-C(1)	122.7(4)
C(2)-C(3)-H(3)	119.7
C(4)-C(3)-C(2)	120.6(4)
C(4)-C(3)-H(3)	119.7
O(1)-C(4)-C(3)	118.9(4)
O(1)-C(4)-C(5)	113.5(3)
C(3)-C(4)-C(5)	127.6(4)
C(6)-C(5)-C(4)	125.3(3)
C(10)-C(5)-C(4)	116.9(3)
C(10)-C(5)-C(6)	117.7(3)
O(3)-C(6)-C(5)	117.1(3)
C(7)-C(6)-O(3)	123.0(4)
C(7)-C(6)-C(5)	119.8(4)
C(6)-C(7)-H(7)	119.7
C(6)-C(7)-C(8)	120.6(4)
C(8)-C(7)-H(7)	119.7
C(7)-C(8)-H(8)	119.5
C(9)-C(8)-C(7)	121.0(4)
C(9)-C(8)-H(8)	119.5
C(8)-C(9)-C(12)	120.9(3)
C(10)-C(9)-C(8)	118.1(4)
C(10)-C(9)-C(12)	120.9(3)
C(5)-C(10)-H(10)	118.6
C(9)-C(10)-C(5)	122.8(4)
C(9)-C(10)-H(10)	118.6
O(3)-C(11)-H(11A)	109.5
O(3)-C(11)-H(11B)	109.5
O(3)-C(11)-H(11C)	109.5
H(11A)-C(11)-H(11B)	109.5

H(11A)-C(11)-H(11C)	109.5
H(11B)-C(11)-H(11C)	109.5
C(13)-C(12)-C(9)	113.9(4)
C(13)-C(12)-C(17)	119.5(4)
C(17)-C(12)-C(9)	126.6(4)
C(12)-C(13)-H(13)	118.8
C(14)-C(13)-C(12)	122.5(5)
C(14)-C(13)-H(13)	118.8
C(13)-C(14)-H(14)	121.0
C(15)-C(14)-C(13)	118.1(5)
C(15)-C(14)-H(14)	121.0
C(14)-C(15)-H(15)	119.9
C(16)-C(15)-C(14)	120.2(4)
C(16)-C(15)-H(15)	119.9
C(15)-C(16)-H(16)	118.4
C(15)-C(16)-C(17)	123.1(5)
C(17)-C(16)-H(16)	118.4
C(12)-C(17)-C(18)	125.1(4)
C(16)-C(17)-C(12)	116.6(4)
C(16)-C(17)-C(18)	118.2(4)
C(17)-C(18)-C(20A)	103.3(7)
C(17)-C(18)-C(21)	107.3(5)
C(19)-C(18)-C(17)	112.2(5)
C(19)-C(18)-C(21)	102.1(7)
C(19A)-C(18)-C(17)	112.8(9)
C(19A)-C(18)-C(20A)	109.1(12)
C(19A)-C(18)-C(21A)	112.7(13)
C(20)-C(18)-C(17)	119.3(6)
C(20)-C(18)-C(19)	107.3(6)
C(20)-C(18)-C(21)	107.1(8)
C(21A)-C(18)-C(17)	111.7(7)
C(21A)-C(18)-C(20A)	106.7(12)
C(18)-C(19)-H(19A)	109.5
C(18)-C(19)-H(19B)	109.5
C(18)-C(19)-H(19C)	109.5
H(19A)-C(19)-H(19B)	109.5
H(19A)-C(19)-H(19C)	109.5
H(19B)-C(19)-H(19C)	109.5

C(18)-C(19A)-H(19D)	109.5
C(18)-C(19A)-H(19E)	109.5
C(18)-C(19A)-H(19F)	109.5
H(19D)-C(19A)-H(19E)	109.5
H(19D)-C(19A)-H(19F)	109.5
H(19E)-C(19A)-H(19F)	109.5
C(18)-C(20)-H(20A)	109.5
C(18)-C(20)-H(20B)	109.5
C(18)-C(20)-H(20C)	109.5
H(20A)-C(20)-H(20B)	109.5
H(20A)-C(20)-H(20C)	109.5
H(20B)-C(20)-H(20C)	109.5
C(18)-C(20A)-H(20D)	109.5
C(18)-C(20A)-H(20E)	109.5
C(18)-C(20A)-H(20F)	109.5
H(20D)-C(20A)-H(20E)	109.5
H(20D)-C(20A)-H(20F)	109.5
H(20E)-C(20A)-H(20F)	109.5
C(18)-C(21)-H(21A)	109.5
C(18)-C(21)-H(21B)	109.5
C(18)-C(21)-H(21C)	109.5
H(21A)-C(21)-H(21B)	109.5
H(21A)-C(21)-H(21C)	109.5
H(21B)-C(21)-H(21C)	109.5
C(18)-C(21A)-H(21D)	109.5
C(18)-C(21A)-H(21E)	109.5
C(18)-C(21A)-H(21F)	109.5
H(21D)-C(21A)-H(21E)	109.5
H(21D)-C(21A)-H(21F)	109.5
H(21E)-C(21A)-H(21F)	109.5
F(1)-B(1)-O(1)	107.9(5)
F(1)-B(1)-O(2)	108.5(5)
F(2)-B(1)-F(1)	111.6(6)
F(2)-B(1)-O(1)	109.4(6)
F(2)-B(1)-O(2)	107.8(5)
O(1)-B(1)-O(2)	111.7(5)

Symmetry transformations used to generate equivalent atoms:

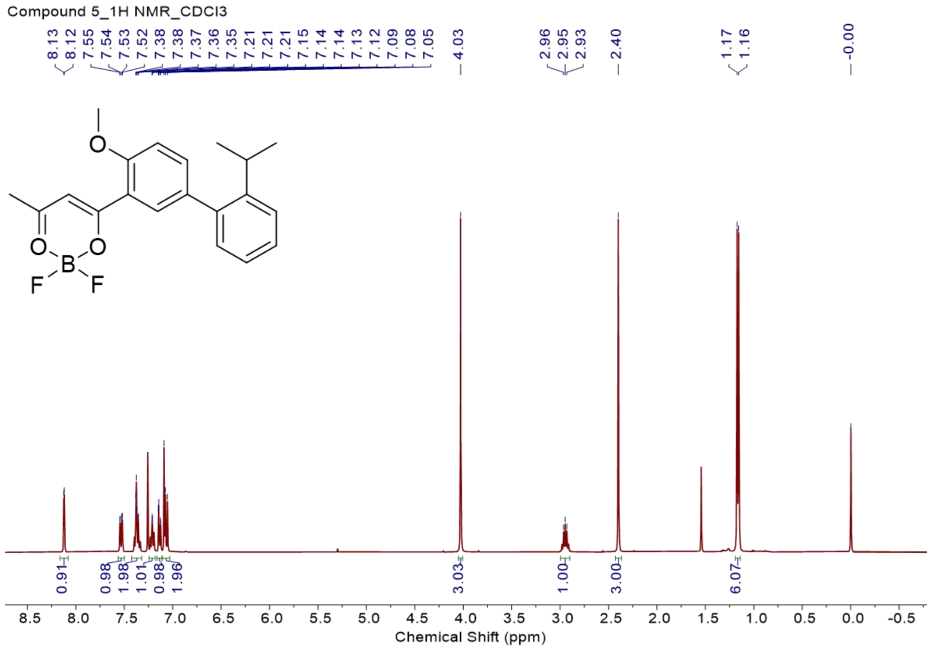

Figure S73. ^{1}H NMR spectra of compound **5**.

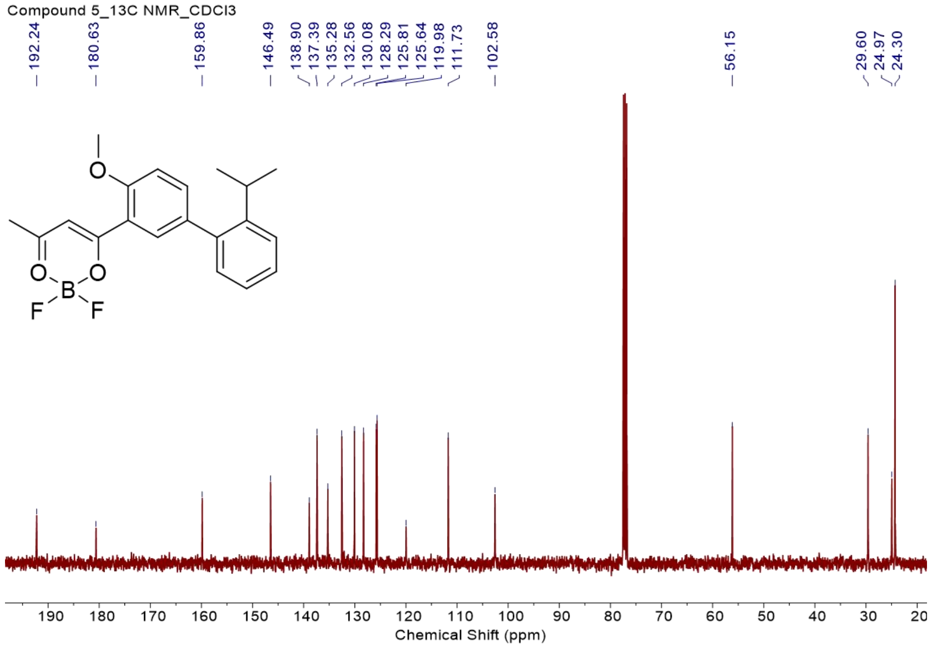

Figure S74. ^{13}C NMR spectra of compound **5**.

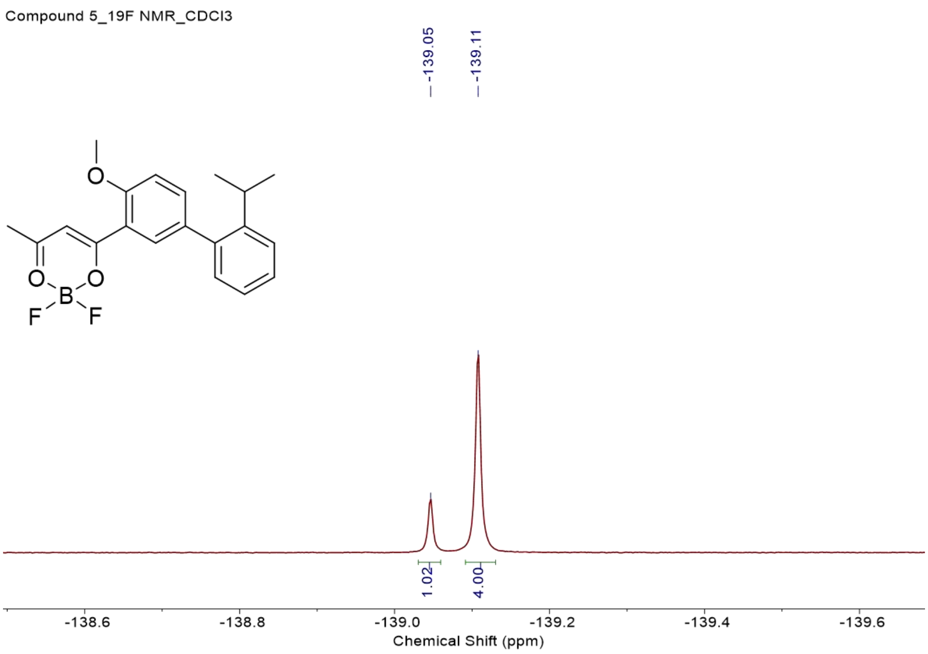

Figure S75. ^{19}F NMR spectra of compound **5**.

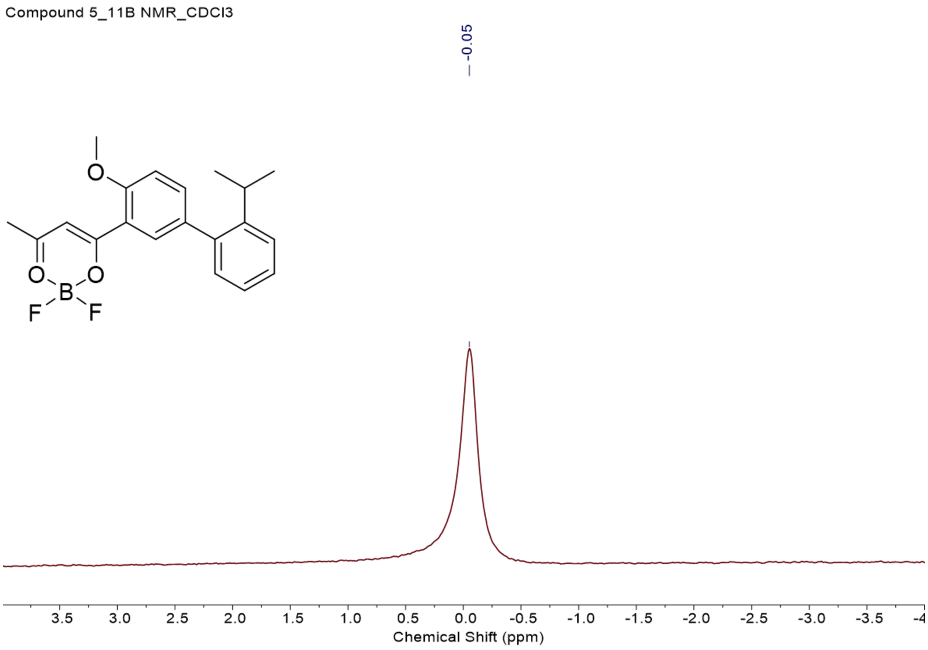

Figure S76. ^{11}B NMR spectra of compound **5**.

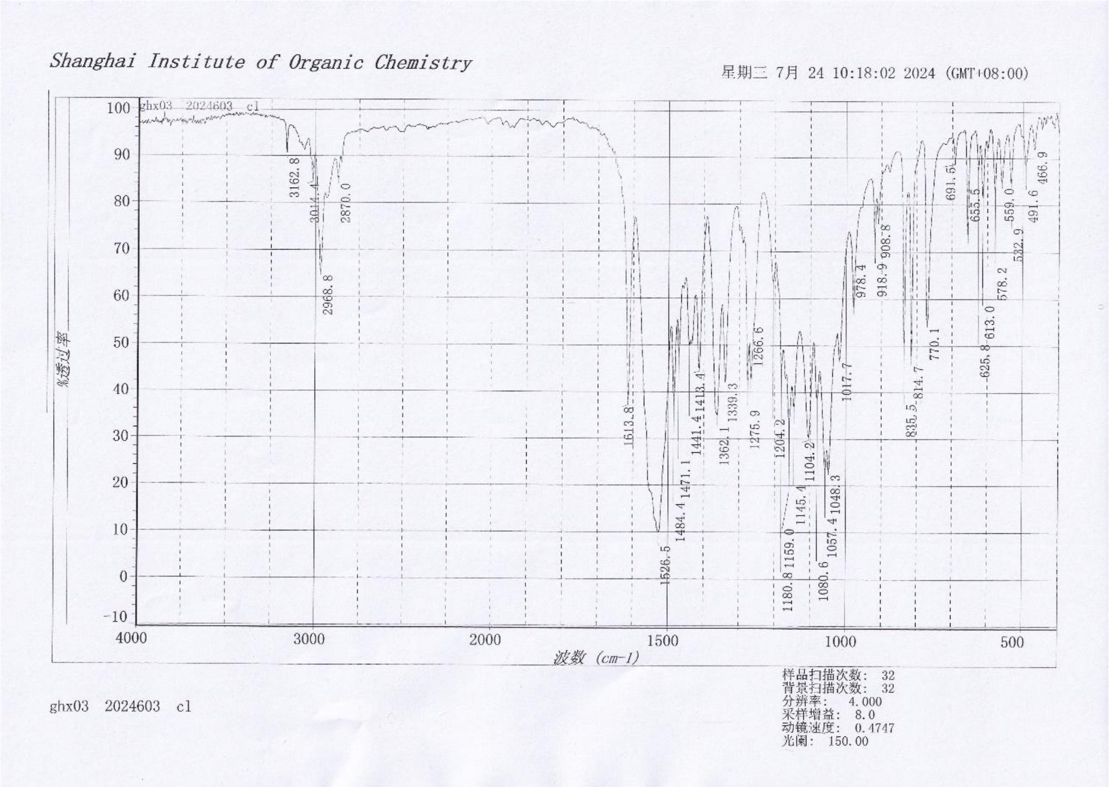

Figure S77. FT-IR spectrum of compound **5**.

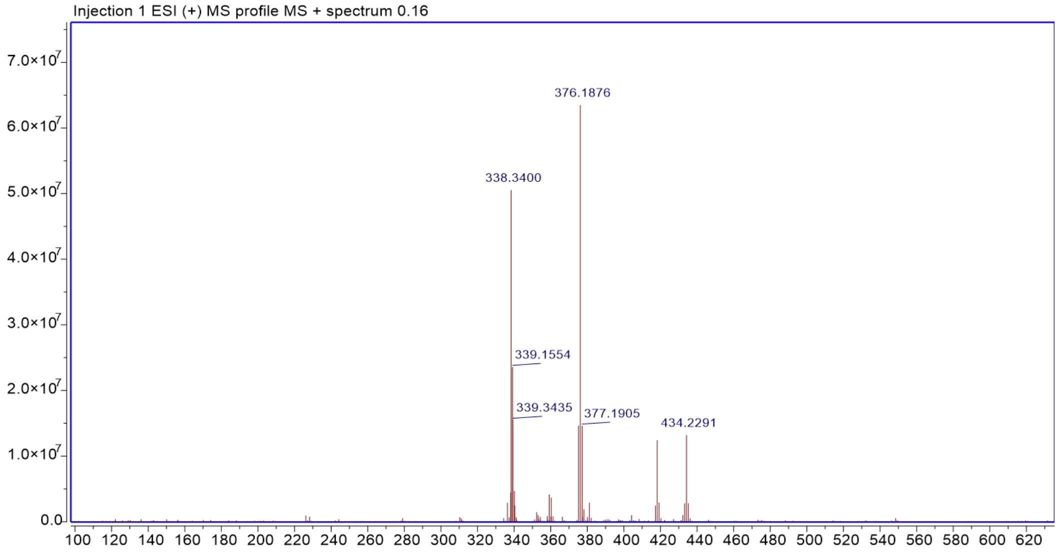

Figure S78. HRMS spectra of compound **5**. The additional signal peaks that 58 units higher than the $[M+NH_4]^+$ peak can be assigned to $[M+NH_4]^+$ with acetone.

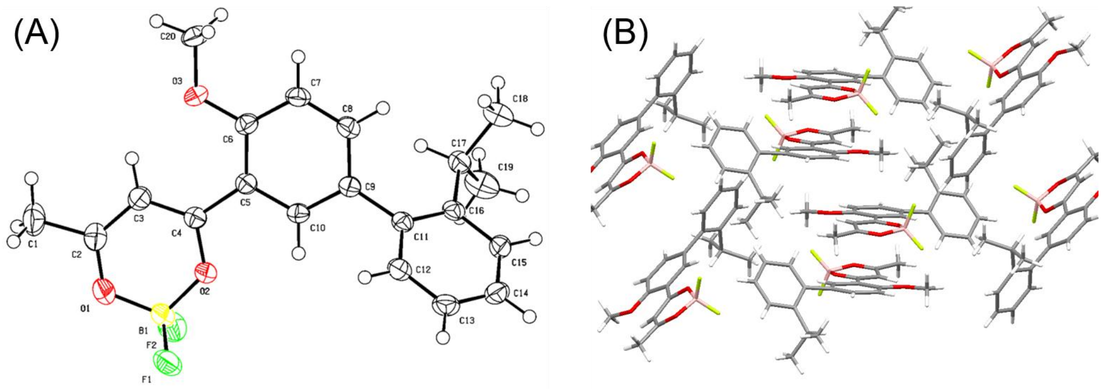

Figure S79. Single crystal structures of compound **5**: (A) monomer, (B) lattice structure; its depository number in CCDC is 2391575.

Table S15. Crystal data and structure refinement for compound **5**.

Identification code	mj24503_0m	
Empirical formula	C20 H21 B F2 O3	
Formula weight	358.18	
Temperature	170.00 K	
Wavelength	1.34139 Å	
Crystal system	Monoclinic	
Space group	P 1 21/c 1	
Unit cell dimensions	a = 10.2839(2) Å	a= 90°.
	b = 7.69780(10) Å	b= 92.0850(10)°.
	c = 23.1459(4) Å	g = 90°.
Volume	1831.10(5) Å^3	
Z	4	
Density (calculated)	1.299 Mg/m^3	
Absorption coefficient	0.524 mm^{-1}	
F(000)	752	
Crystal size	0.17 x 0.17 x 0.05 mm^3	
Theta range for data collection	3.742 to 54.861°.	
Index ranges	-12<=h<=12, -4<=k<=9, -28<=l<=28	
Reflections collected	19621	
Independent reflections	3465 [R(int) = 0.0460]	
Completeness to theta = 53.594°	99.3 %	
Absorption correction	Semi-empirical from equivalents	
Max. and min. transmission	0.7508 and 0.6359	
Refinement method	Full-matrix least-squares on F^2	
Data / restraints / parameters	3465 / 0 / 239	

Goodness-of-fit on F^2	1.045
Final R indices [I>2sigma(I)]	R1 = 0.0420, wR2 = 0.1053
R indices (all data)	R1 = 0.0537, wR2 = 0.1134
Extinction coefficient	n/a
Largest diff. peak and hole	0.339 and -0.271 e.Å^{-3}

Table S16. Atomic coordinates (x 10^4) and equivalent isotropic displacement parameters (Å^{2}x 10^3) for compound **5**. U(eq) is defined as one third of the trace of the orthogonalized U^{ij} tensor.

	x	y	z	U(eq)
F(1)	727(1)	4495(1)	5727(1)	46(1)
F(2)	1000(1)	1627(1)	5905(1)	46(1)
O(1)	940(1)	2599(2)	4956(1)	43(1)
O(2)	2757(1)	3287(2)	5625(1)	37(1)
O(3)	5801(1)	1967(2)	4555(1)	37(1)
C(1)	1229(2)	1615(3)	4008(1)	50(1)
C(2)	1787(2)	2114(2)	4588(1)	34(1)
C(3)	3109(2)	2101(2)	4721(1)	33(1)
C(4)	3583(1)	2768(2)	5243(1)	26(1)
C(5)	4956(1)	3028(2)	5428(1)	26(1)
C(6)	6039(2)	2644(2)	5091(1)	28(1)
C(7)	7295(2)	2969(2)	5315(1)	31(1)
C(8)	7490(1)	3661(2)	5862(1)	31(1)
C(9)	6445(1)	4071(2)	6207(1)	26(1)
C(10)	5200(1)	3748(2)	5979(1)	26(1)
C(11)	6606(1)	4926(2)	6784(1)	26(1)
C(12)	5914(1)	6463(2)	6870(1)	32(1)
C(13)	5990(2)	7346(2)	7390(1)	38(1)
C(14)	6791(2)	6704(2)	7835(1)	37(1)
C(15)	7469(1)	5179(2)	7758(1)	31(1)
C(16)	7389(1)	4243(2)	7241(1)	26(1)
C(17)	8102(1)	2520(2)	7202(1)	32(1)
C(18)	9571(2)	2768(3)	7192(1)	45(1)
C(19)	7768(2)	1306(3)	7699(1)	50(1)

C(20)	6889(2)	1709(3)	4193(1)	43(1)
B(1)	1333(2)	3013(3)	5565(1)	34(1)

Table S17. Bond lengths [Å] and angles [°] for compound **5**.

F(1)-B(1)	1.359(2)
F(2)-B(1)	1.377(2)
O(1)-C(2)	1.295(2)
O(1)-B(1)	1.487(2)
O(2)-C(4)	1.3109(18)
O(2)-B(1)	1.481(2)
O(3)-C(6)	1.3585(18)
O(3)-C(20)	1.4364(19)
C(1)-H(1A)	0.9800
C(1)-H(1B)	0.9800
C(1)-H(1C)	0.9800
C(1)-C(2)	1.490(2)
C(2)-C(3)	1.383(2)
C(3)-H(3)	0.9500
C(3)-C(4)	1.385(2)
C(4)-C(5)	1.474(2)
C(5)-C(6)	1.414(2)
C(5)-C(10)	1.405(2)
C(6)-C(7)	1.397(2)
C(7)-H(7)	0.9500
C(7)-C(8)	1.382(2)
C(8)-H(8)	0.9500
C(8)-C(9)	1.398(2)
C(9)-C(10)	1.389(2)
C(9)-C(11)	1.494(2)
C(10)-H(10)	0.9500
C(11)-C(12)	1.398(2)
C(11)-C(16)	1.406(2)
C(12)-H(12)	0.9500
C(12)-C(13)	1.383(2)
C(13)-H(13)	0.9500
C(13)-C(14)	1.386(2)
C(14)-H(14)	0.9500

C(14)-C(15)	1.380(2)
C(15)-H(15)	0.9500
C(15)-C(16)	1.398(2)
C(16)-C(17)	1.520(2)
C(17)-H(17)	1.0000
C(17)-C(18)	1.523(2)
C(17)-C(19)	1.531(2)
C(18)-H(18A)	0.9800
C(18)-H(18B)	0.9800
C(18)-H(18C)	0.9800
C(19)-H(19A)	0.9800
C(19)-H(19B)	0.9800
C(19)-H(19C)	0.9800
C(20)-H(20A)	0.9800
C(20)-H(20B)	0.9800
C(20)-H(20C)	0.9800

C(2)-O(1)-B(1)	121.28(12)
C(4)-O(2)-B(1)	123.79(13)
C(6)-O(3)-C(20)	117.89(13)
H(1A)-C(1)-H(1B)	109.5
H(1A)-C(1)-H(1C)	109.5
H(1B)-C(1)-H(1C)	109.5
C(2)-C(1)-H(1A)	109.5
C(2)-C(1)-H(1B)	109.5
C(2)-C(1)-H(1C)	109.5
O(1)-C(2)-C(1)	114.83(15)
O(1)-C(2)-C(3)	122.30(14)
C(3)-C(2)-C(1)	122.86(16)
C(2)-C(3)-H(3)	119.9
C(2)-C(3)-C(4)	120.27(15)
C(4)-C(3)-H(3)	119.9
O(2)-C(4)-C(3)	119.03(13)
O(2)-C(4)-C(5)	113.63(13)
C(3)-C(4)-C(5)	127.31(14)
C(6)-C(5)-C(4)	125.26(13)
C(10)-C(5)-C(4)	117.02(13)
C(10)-C(5)-C(6)	117.70(13)

O(3)-C(6)-C(5)	117.68(13)
O(3)-C(6)-C(7)	122.70(13)
C(7)-C(6)-C(5)	119.62(13)
C(6)-C(7)-H(7)	119.7
C(8)-C(7)-C(6)	120.69(14)
C(8)-C(7)-H(7)	119.7
C(7)-C(8)-H(8)	119.3
C(7)-C(8)-C(9)	121.42(14)
C(9)-C(8)-H(8)	119.3
C(8)-C(9)-C(11)	123.20(13)
C(10)-C(9)-C(8)	117.37(13)
C(10)-C(9)-C(11)	119.32(13)
C(5)-C(10)-H(10)	118.4
C(9)-C(10)-C(5)	123.19(13)
C(9)-C(10)-H(10)	118.4
C(12)-C(11)-C(9)	117.30(13)
C(12)-C(11)-C(16)	119.37(14)
C(16)-C(11)-C(9)	123.32(13)
C(11)-C(12)-H(12)	119.1
C(13)-C(12)-C(11)	121.74(15)
C(13)-C(12)-H(12)	119.1
C(12)-C(13)-H(13)	120.5
C(12)-C(13)-C(14)	119.03(15)
C(14)-C(13)-H(13)	120.5
C(13)-C(14)-H(14)	120.1
C(15)-C(14)-C(13)	119.81(15)
C(15)-C(14)-H(14)	120.1
C(14)-C(15)-H(15)	118.9
C(14)-C(15)-C(16)	122.26(15)
C(16)-C(15)-H(15)	118.9
C(11)-C(16)-C(17)	123.11(13)
C(15)-C(16)-C(11)	117.76(14)
C(15)-C(16)-C(17)	119.10(13)
C(16)-C(17)-H(17)	107.8
C(16)-C(17)-C(18)	111.86(14)
C(16)-C(17)-C(19)	111.54(13)
C(18)-C(17)-H(17)	107.8
C(18)-C(17)-C(19)	109.73(14)

C(19)-C(17)-H(17)	107.8
C(17)-C(18)-H(18A)	109.5
C(17)-C(18)-H(18B)	109.5
C(17)-C(18)-H(18C)	109.5
H(18A)-C(18)-H(18B)	109.5
H(18A)-C(18)-H(18C)	109.5
H(18B)-C(18)-H(18C)	109.5
C(17)-C(19)-H(19A)	109.5
C(17)-C(19)-H(19B)	109.5
C(17)-C(19)-H(19C)	109.5
H(19A)-C(19)-H(19B)	109.5
H(19A)-C(19)-H(19C)	109.5
H(19B)-C(19)-H(19C)	109.5
O(3)-C(20)-H(20A)	109.5
O(3)-C(20)-H(20B)	109.5
O(3)-C(20)-H(20C)	109.5
H(20A)-C(20)-H(20B)	109.5
H(20A)-C(20)-H(20C)	109.5
H(20B)-C(20)-H(20C)	109.5
F(1)-B(1)-F(2)	111.48(14)
F(1)-B(1)-O(1)	109.30(14)
F(1)-B(1)-O(2)	108.43(14)
F(2)-B(1)-O(1)	108.15(14)
F(2)-B(1)-O(2)	108.80(14)
O(2)-B(1)-O(1)	110.69(13)

Symmetry transformations used to generate equivalent atoms:

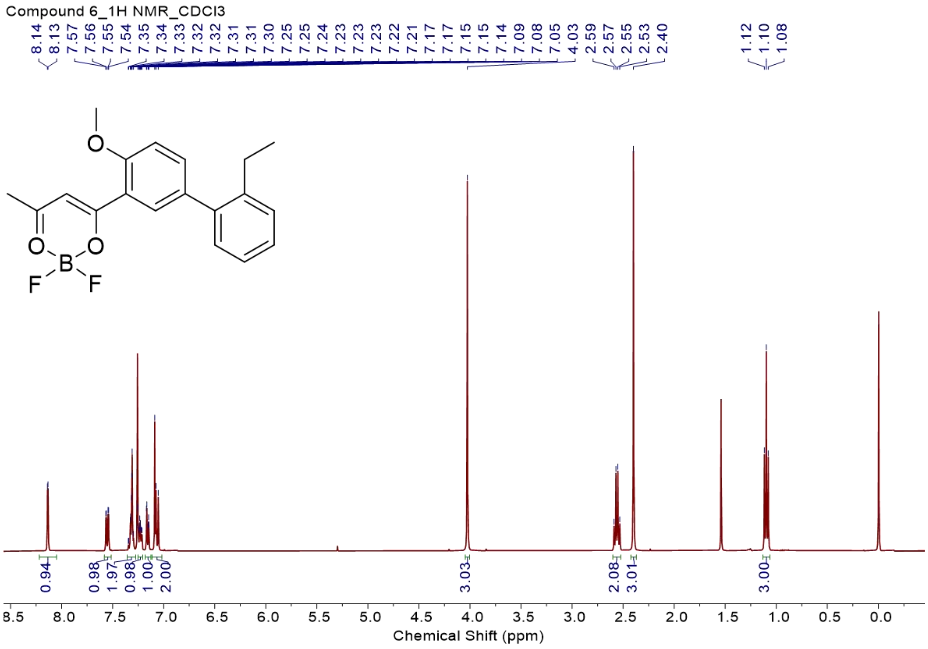

Figure S80. ^{1}H NMR spectra of compound **6**.

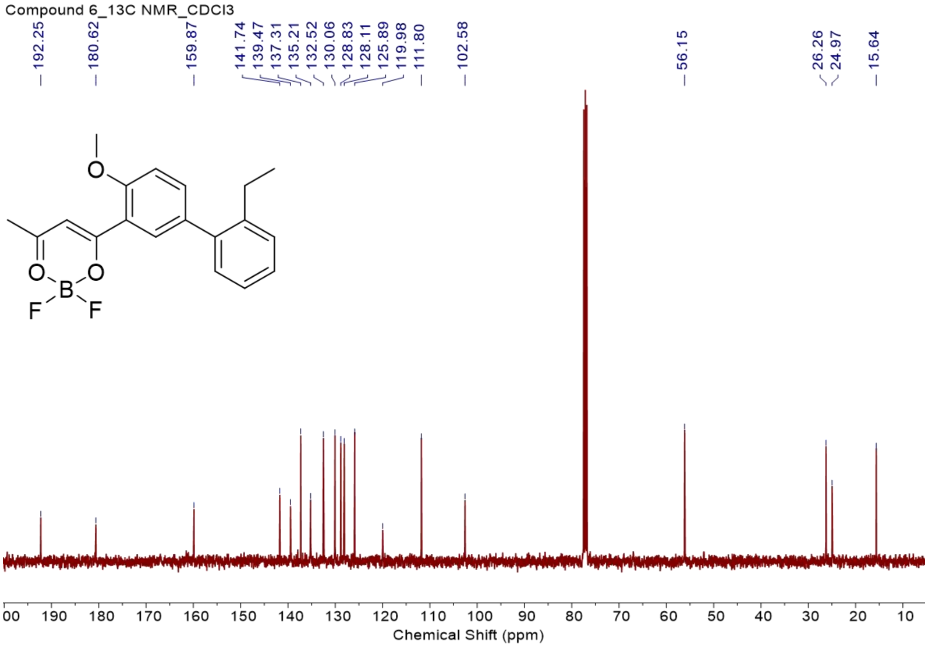

Figure S81. ^{13}C NMR spectra of compound **6**.

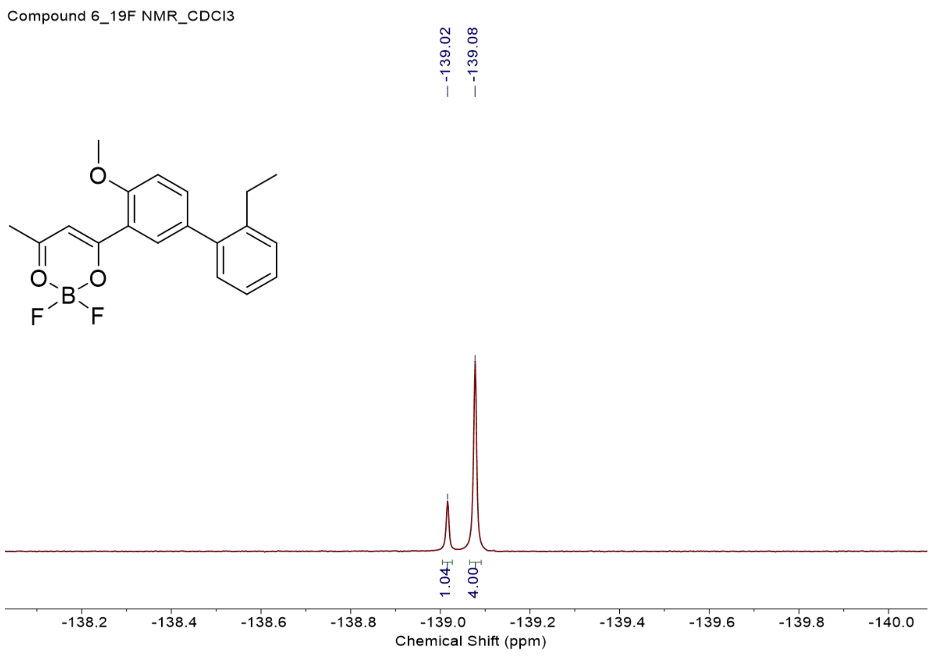

Figure S82. ^{19}F NMR spectra of compound **6**.

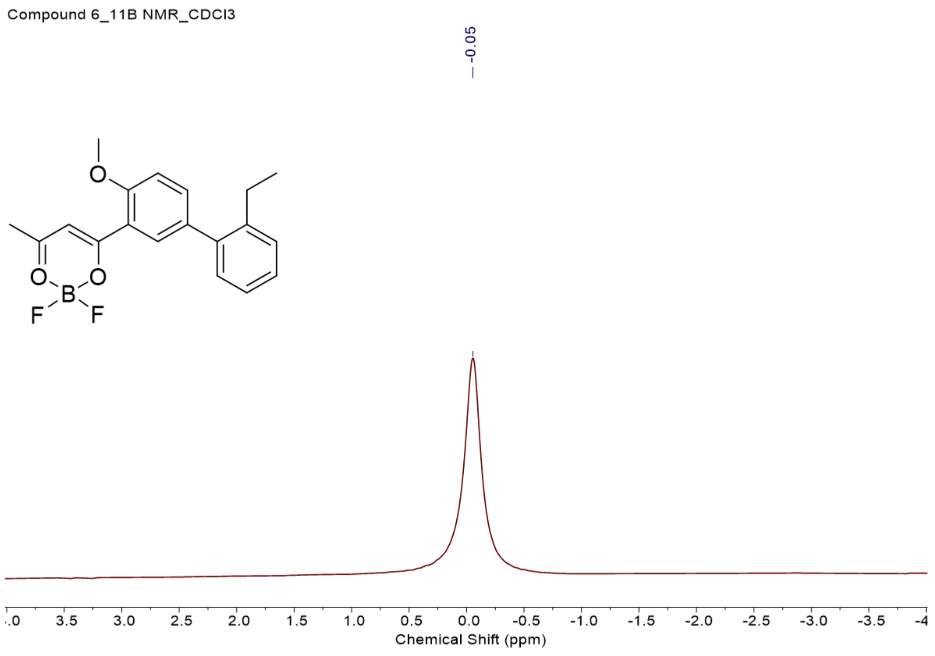

Figure S83. ^{11}B NMR spectra of compound **6**.

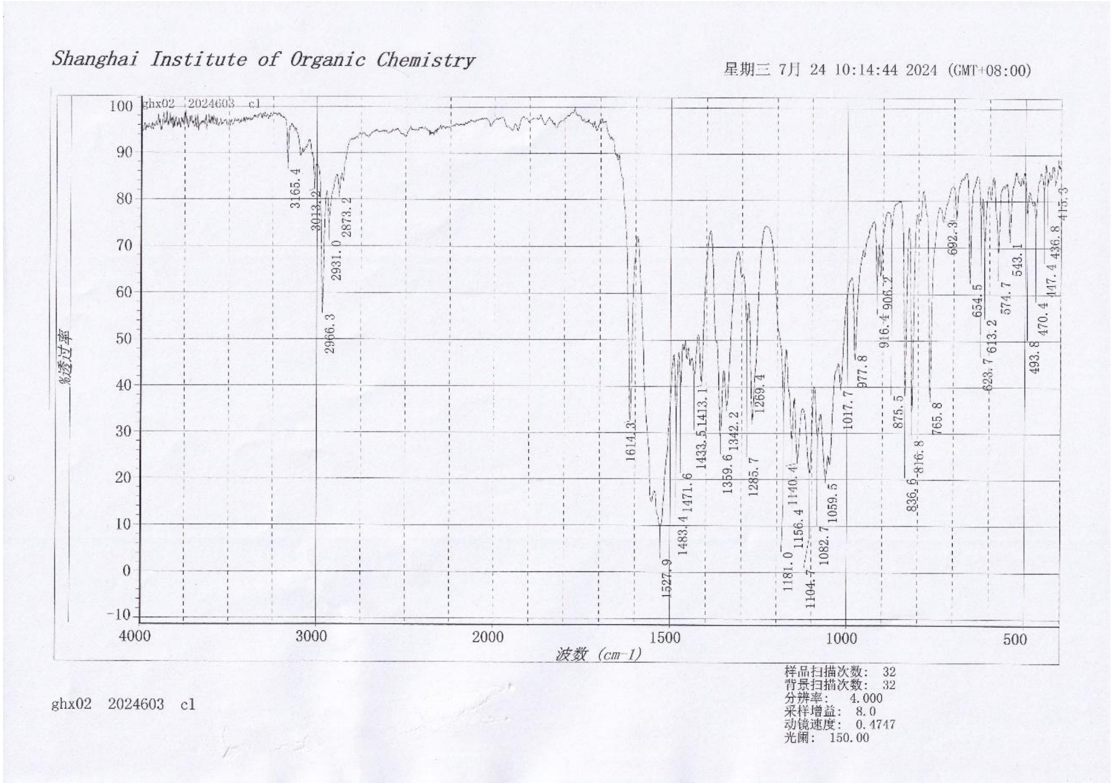

Figure S84. FT-IR spectrum of compound **6**.

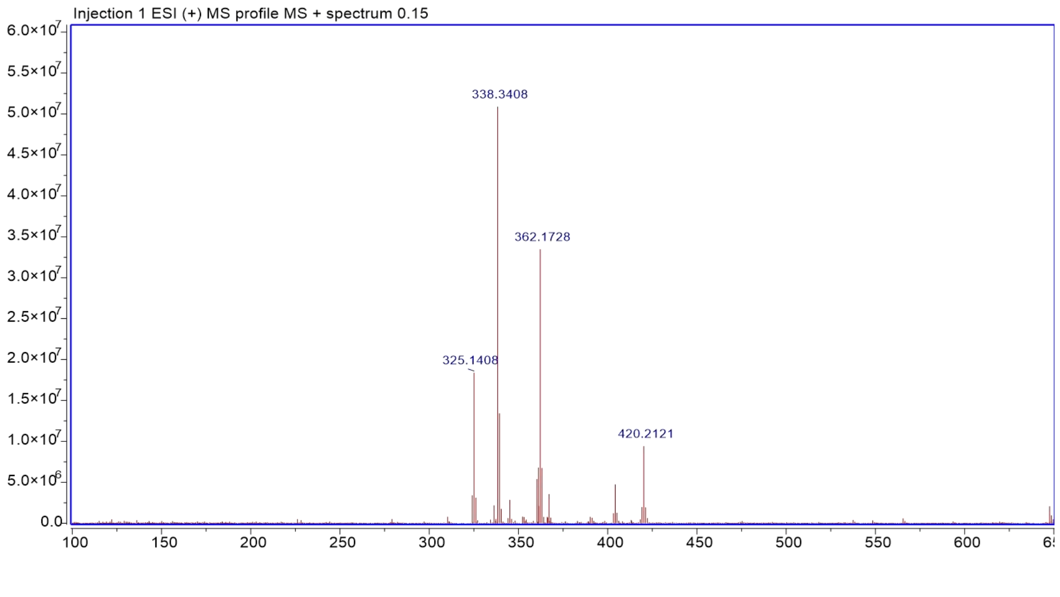

Figure S85. HRMS spectra of compound **6**. The additional signal peaks that 58 units higher than the $[M+NH_4]^+$ peak can be assigned to $[M+NH_4]^+$ with acetone.

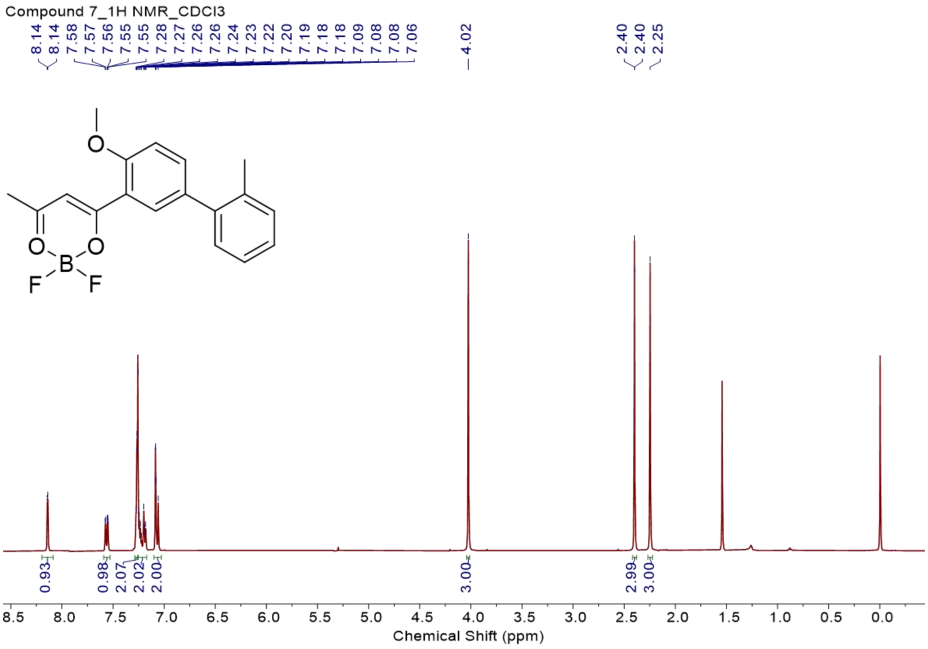

Figure S86. ^{1}H NMR spectra of compound **7**.

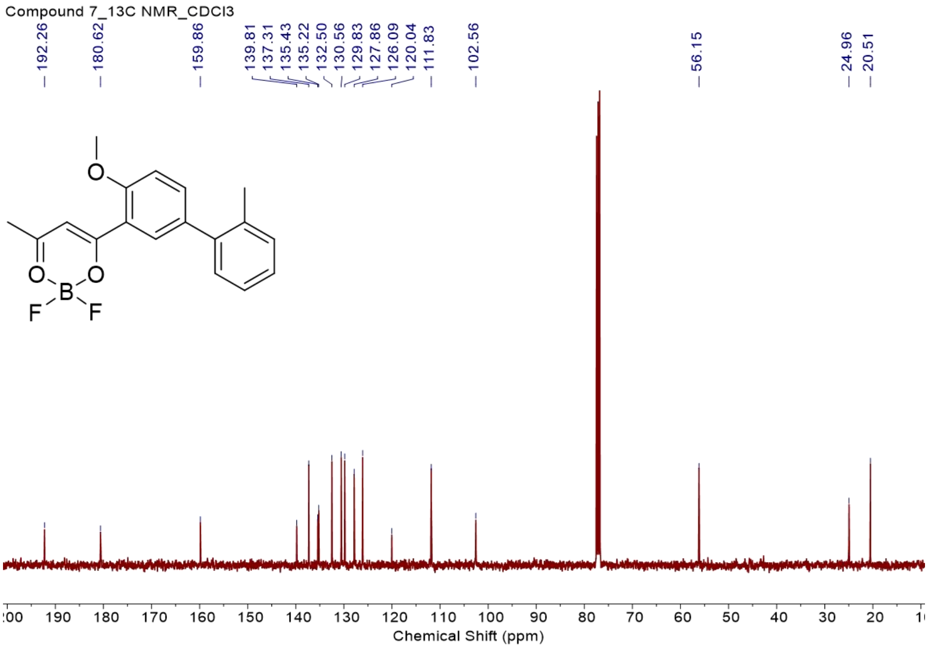

Figure S87. ^{13}C NMR spectra of compound **7**.

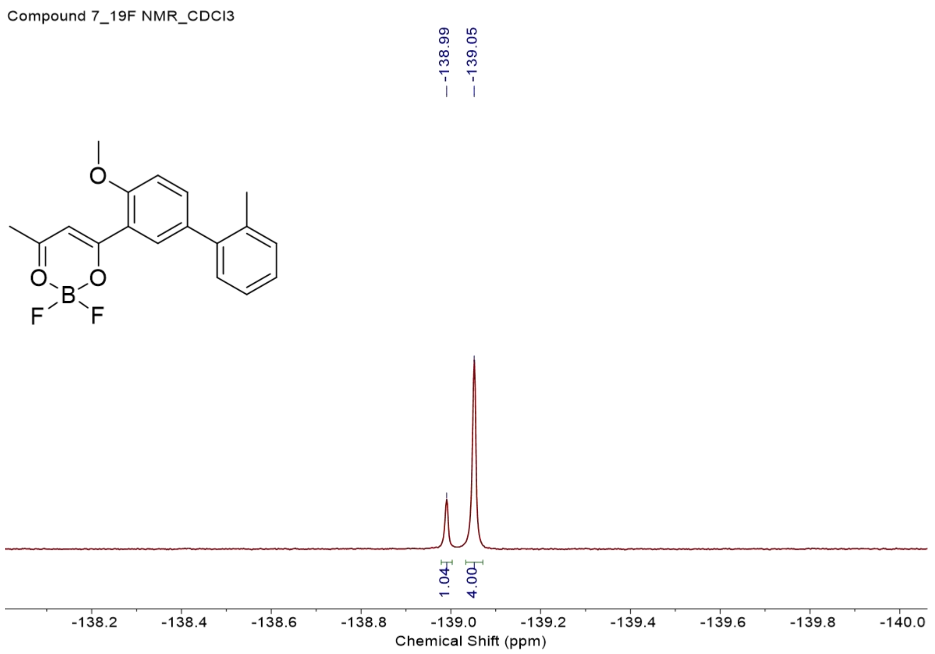

Figure S88. ^{19}F NMR spectra of compound **7**.

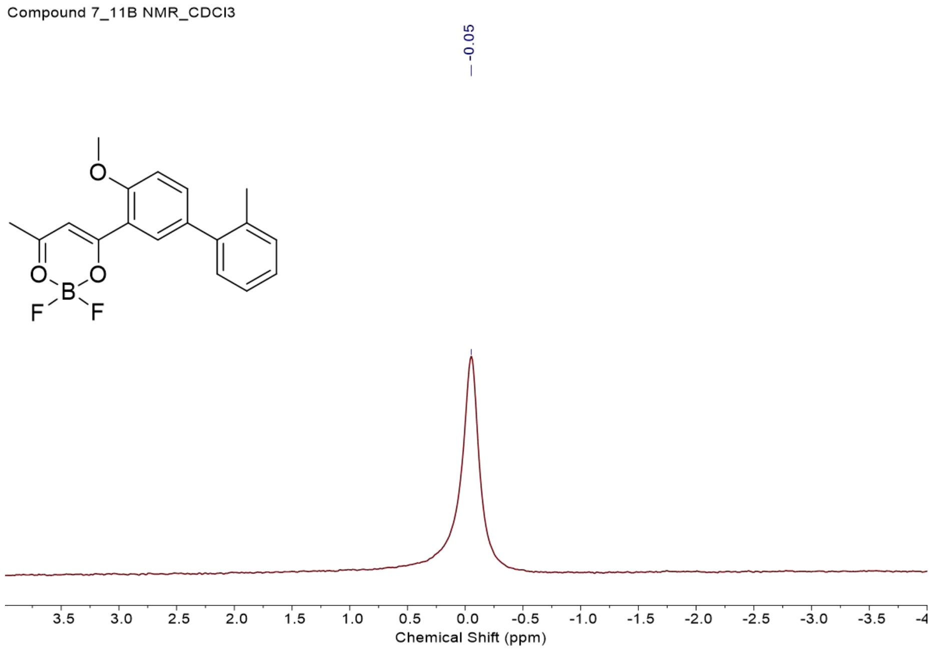

Figure S89. ^{11}B NMR spectra of compound **7**.

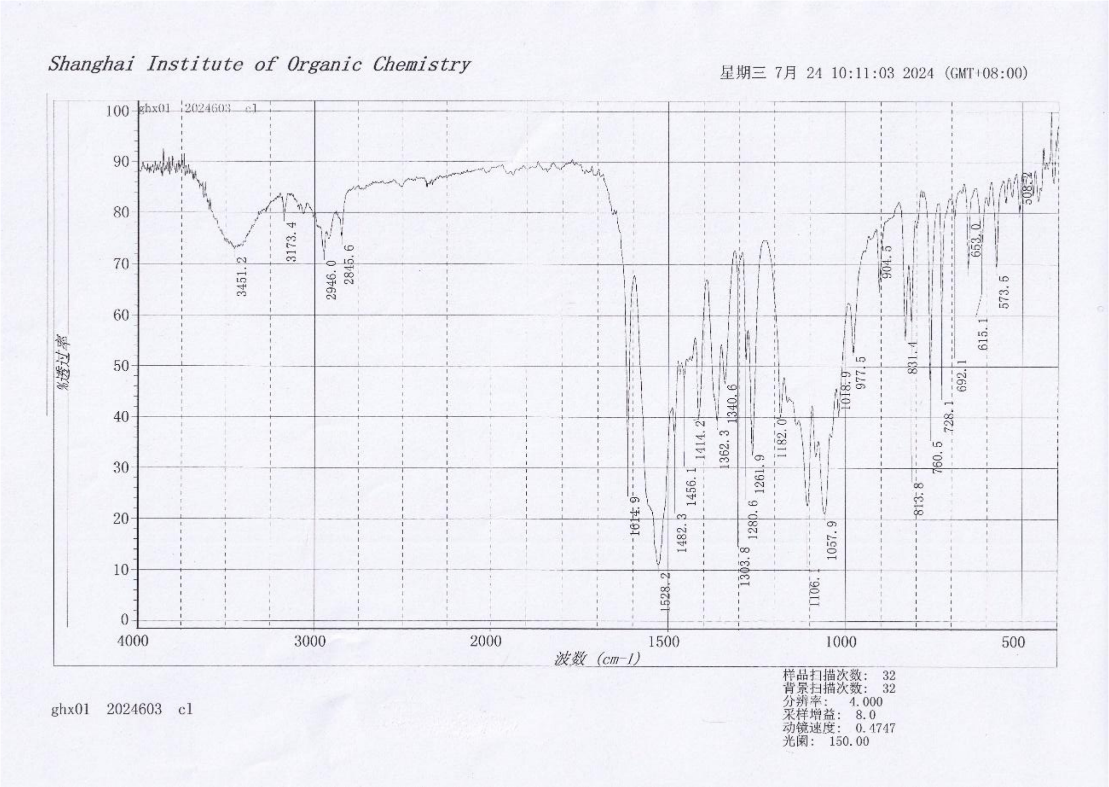

Figure S90. FT-IR spectrum of compound **7**.

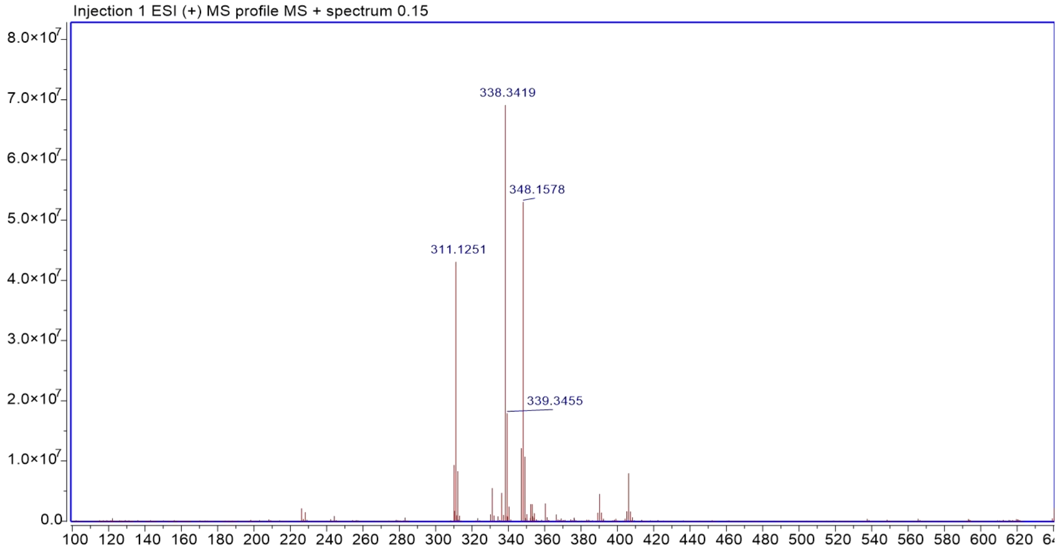

Figure S91. HRMS spectra of compound **7**.

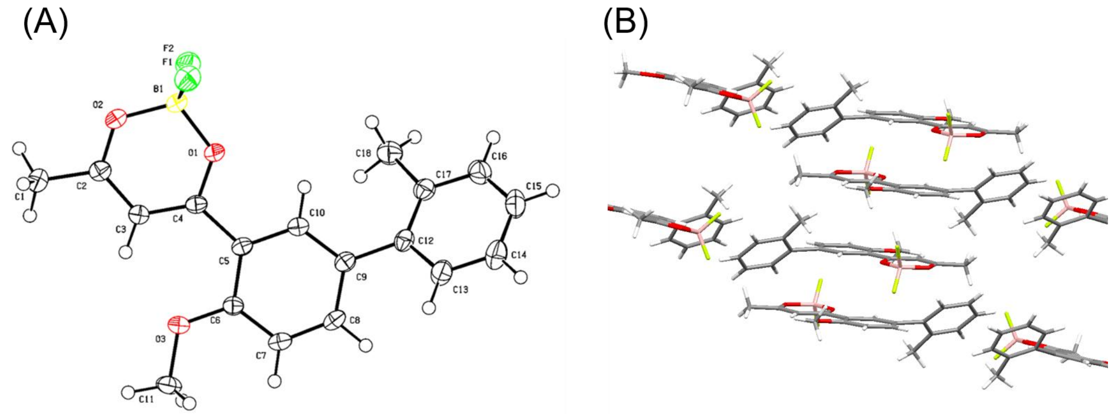

Figure S92. Single crystal structures of compound **7**: (A) monomer, (B) lattice structure; its depository number in CCDC is 2391576.

Table S18. Crystal data and structure refinement for compound **7**.

Identification code	mj24637_0m	
Empirical formula	C18 H17 B F2 O3	
Formula weight	330.12	
Temperature	170.00 K	
Wavelength	1.34139 Å	
Crystal system	Monoclinic	
Space group	P 1 21/c 1	
Unit cell dimensions	a = 7.5499(2) Å	a= 90°.
	b = 21.6676(4) Å	b= 107.2250(10)°.
	c = 10.1346(2) Å	g = 90°.
Volume	1583.54(6) Å^3	
Z	4	
Density (calculated)	1.385 Mg/m^3	
Absorption coefficient	0.576 mm^{-1}	
F(000)	688	
Crystal size	0.17 x 0.17 x 0.05 mm^3	
Theta range for data collection	4.352 to 54.854°.	
Index ranges	-9<=h<=9, -26<=k<=24, -12<=l<=12	
Reflections collected	16470	
Independent reflections	2996 [R(int) = 0.0523]	
Completeness to theta = 53.594°	99.4 %	
Absorption correction	Semi-empirical from equivalents	
Max. and min. transmission	0.7508 and 0.6614	
Refinement method	Full-matrix least-squares on F^2	

Data / restraints / parameters	2996 / 0 / 220
Goodness-of-fit on F^2	1.052
Final R indices [I>2sigma(I)]	R1 = 0.0425, wR2 = 0.1012
R indices (all data)	R1 = 0.0587, wR2 = 0.1107
Extinction coefficient	n/a
Largest diff. peak and hole	0.234 and -0.234 e.Å^{-3}

Table S19. Atomic coordinates (x 10^4) and equivalent isotropic displacement parameters (Å^{2}x 10^3) for compound **7**. U(eq) is defined as one third of the trace of the orthogonalized U^{ij} tensor.

	x	y	z	U(eq)
F(1)	4004(1)	5673(1)	9931(1)	36(1)
F(2)	901(1)	5784(1)	9149(1)	35(1)
O(1)	2504(2)	5497(1)	7616(1)	38(1)
O(2)	2141(2)	4796(1)	9418(1)	30(1)
O(3)	2284(2)	4161(1)	4591(1)	29(1)
C(1)	1782(3)	3722(1)	9081(2)	32(1)
C(2)	2011(2)	4346(1)	8554(2)	24(1)
C(3)	2094(2)	4443(1)	7220(2)	24(1)
C(4)	2349(2)	5028(1)	6771(2)	22(1)
C(5)	2466(2)	5199(1)	5391(2)	21(1)
C(6)	2439(2)	4771(1)	4326(2)	22(1)
C(7)	2577(2)	4989(1)	3065(2)	26(1)
C(8)	2755(2)	5611(1)	2853(2)	26(1)
C(9)	2808(2)	6049(1)	3884(2)	23(1)
C(10)	2652(2)	5828(1)	5130(2)	22(1)
C(11)	2445(3)	3715(1)	3586(2)	32(1)
C(12)	3150(2)	6713(1)	3666(2)	25(1)
C(13)	4496(2)	6856(1)	3009(2)	33(1)
C(14)	4980(3)	7458(1)	2827(2)	40(1)
C(15)	4120(3)	7939(1)	3296(2)	38(1)
C(16)	2755(3)	7809(1)	3915(2)	35(1)
C(17)	2232(2)	7204(1)	4101(2)	27(1)
C(18)	669(3)	7114(1)	4736(2)	36(1)

B(1)	2397(3)	5444(1)	9044(2)	26(1)

Table S20. Bond lengths [Å] and angles [°] for compound **7**.

F(1)-B(1)	1.371(2)
F(2)-B(1)	1.379(2)
O(1)-C(4)	1.3109(19)
O(1)-B(1)	1.477(2)
O(2)-C(2)	1.294(2)
O(2)-B(1)	1.481(2)
O(3)-C(6)	1.361(2)
O(3)-C(11)	1.4341(19)
C(1)-H(1A)	0.9800
C(1)-H(1B)	0.9800
C(1)-H(1C)	0.9800
C(1)-C(2)	1.484(2)
C(2)-C(3)	1.388(2)
C(3)-H(3)	0.9500
C(3)-C(4)	1.379(2)
C(4)-C(5)	1.474(2)
C(5)-C(6)	1.419(2)
C(5)-C(10)	1.404(2)
C(6)-C(7)	1.394(2)
C(7)-H(7)	0.9500
C(7)-C(8)	1.378(2)
C(8)-H(8)	0.9500
C(8)-C(9)	1.404(2)
C(9)-C(10)	1.388(2)
C(9)-C(12)	1.489(2)
C(10)-H(10)	0.9500
C(11)-H(11A)	0.9800
C(11)-H(11B)	0.9800
C(11)-H(11C)	0.9800
C(12)-C(13)	1.405(2)
C(12)-C(17)	1.411(2)
C(13)-H(13)	0.9500
C(13)-C(14)	1.383(3)

C(14)-H(14)	0.9500
C(14)-C(15)	1.383(3)
C(15)-H(15)	0.9500
C(15)-C(16)	1.385(3)
C(16)-H(16)	0.9500
C(16)-C(17)	1.396(3)
C(17)-C(18)	1.515(2)
C(18)-H(18A)	0.9800
C(18)-H(18B)	0.9800
C(18)-H(18C)	0.9800

C(4)-O(1)-B(1)	124.07(14)
C(2)-O(2)-B(1)	121.94(13)
C(6)-O(3)-C(11)	118.90(13)
H(1A)-C(1)-H(1B)	109.5
H(1A)-C(1)-H(1C)	109.5
H(1B)-C(1)-H(1C)	109.5
C(2)-C(1)-H(1A)	109.5
C(2)-C(1)-H(1B)	109.5
C(2)-C(1)-H(1C)	109.5
O(2)-C(2)-C(1)	115.89(14)
O(2)-C(2)-C(3)	121.97(15)
C(3)-C(2)-C(1)	122.14(15)
C(2)-C(3)-H(3)	119.6
C(4)-C(3)-C(2)	120.72(15)
C(4)-C(3)-H(3)	119.6
O(1)-C(4)-C(3)	119.34(14)
O(1)-C(4)-C(5)	114.01(14)
C(3)-C(4)-C(5)	126.64(14)
C(6)-C(5)-C(4)	124.49(15)
C(10)-C(5)-C(4)	117.38(14)
C(10)-C(5)-C(6)	118.12(14)
O(3)-C(6)-C(5)	117.86(14)
O(3)-C(6)-C(7)	122.95(14)
C(7)-C(6)-C(5)	119.19(15)
C(6)-C(7)-H(7)	119.6
C(8)-C(7)-C(6)	120.78(15)
C(8)-C(7)-H(7)	119.6

C(7)-C(8)-H(8)	119.1
C(7)-C(8)-C(9)	121.84(15)
C(9)-C(8)-H(8)	119.1
C(8)-C(9)-C(12)	120.71(14)
C(10)-C(9)-C(8)	116.93(15)
C(10)-C(9)-C(12)	122.22(14)
C(5)-C(10)-H(10)	118.4
C(9)-C(10)-C(5)	123.13(15)
C(9)-C(10)-H(10)	118.4
O(3)-C(11)-H(11A)	109.5
O(3)-C(11)-H(11B)	109.5
O(3)-C(11)-H(11C)	109.5
H(11A)-C(11)-H(11B)	109.5
H(11A)-C(11)-H(11C)	109.5
H(11B)-C(11)-H(11C)	109.5
C(13)-C(12)-C(9)	117.73(15)
C(13)-C(12)-C(17)	118.23(16)
C(17)-C(12)-C(9)	124.03(15)
C(12)-C(13)-H(13)	119.1
C(14)-C(13)-C(12)	121.87(17)
C(14)-C(13)-H(13)	119.1
C(13)-C(14)-H(14)	120.1
C(13)-C(14)-C(15)	119.73(18)
C(15)-C(14)-H(14)	120.1
C(14)-C(15)-H(15)	120.3
C(14)-C(15)-C(16)	119.33(17)
C(16)-C(15)-H(15)	120.3
C(15)-C(16)-H(16)	119.0
C(15)-C(16)-C(17)	122.04(18)
C(17)-C(16)-H(16)	119.0
C(12)-C(17)-C(18)	123.49(16)
C(16)-C(17)-C(12)	118.74(16)
C(16)-C(17)-C(18)	117.75(16)
C(17)-C(18)-H(18A)	109.5
C(17)-C(18)-H(18B)	109.5
C(17)-C(18)-H(18C)	109.5
H(18A)-C(18)-H(18B)	109.5
H(18A)-C(18)-H(18C)	109.5

H(18B)-C(18)-H(18C)	109.5
F(1)-B(1)-F(2)	109.94(14)
F(1)-B(1)-O(1)	108.68(14)
F(1)-B(1)-O(2)	109.45(14)
F(2)-B(1)-O(1)	108.55(14)
F(2)-B(1)-O(2)	108.26(14)
O(1)-B(1)-O(2)	111.93(14)

Symmetry transformations used to generate equivalent atoms:

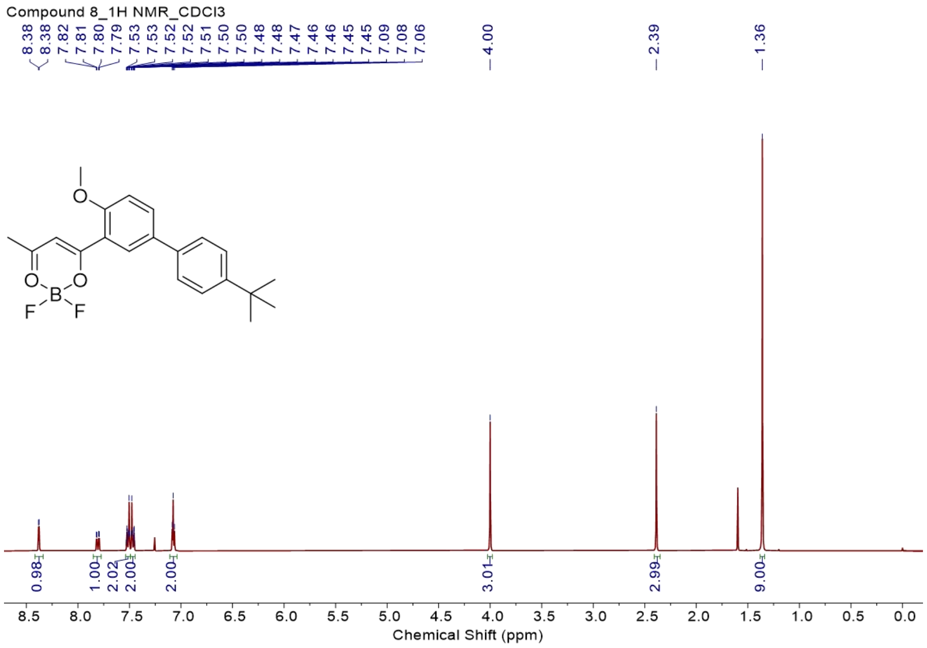

Figure S93. ^{1}H NMR spectra of compound **8**.

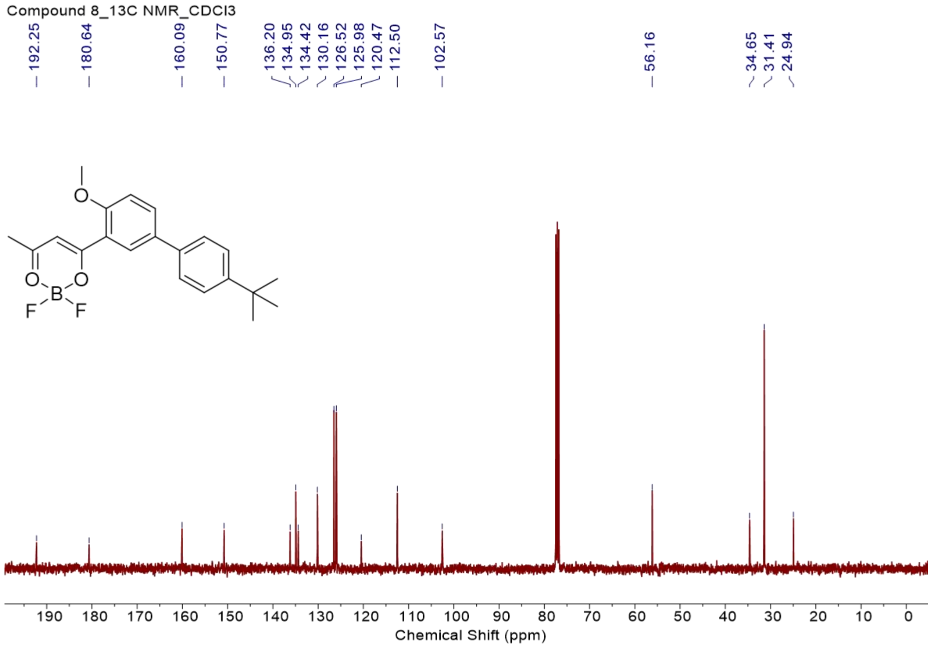

Figure S94. ^{13}C NMR spectra of compound **8**.

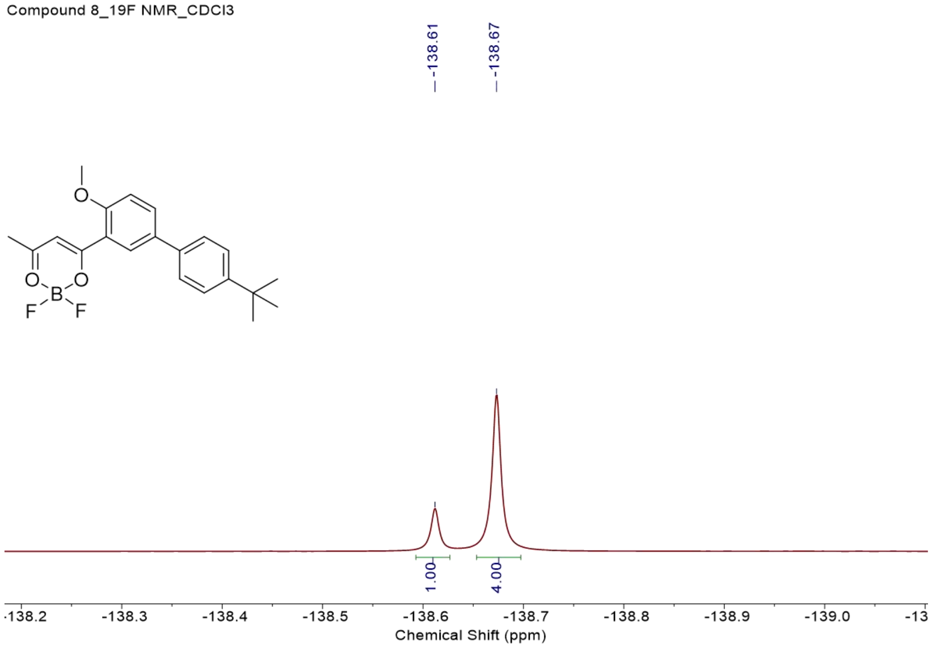

Figure S95. ^{19}F NMR spectra of compound **8**.

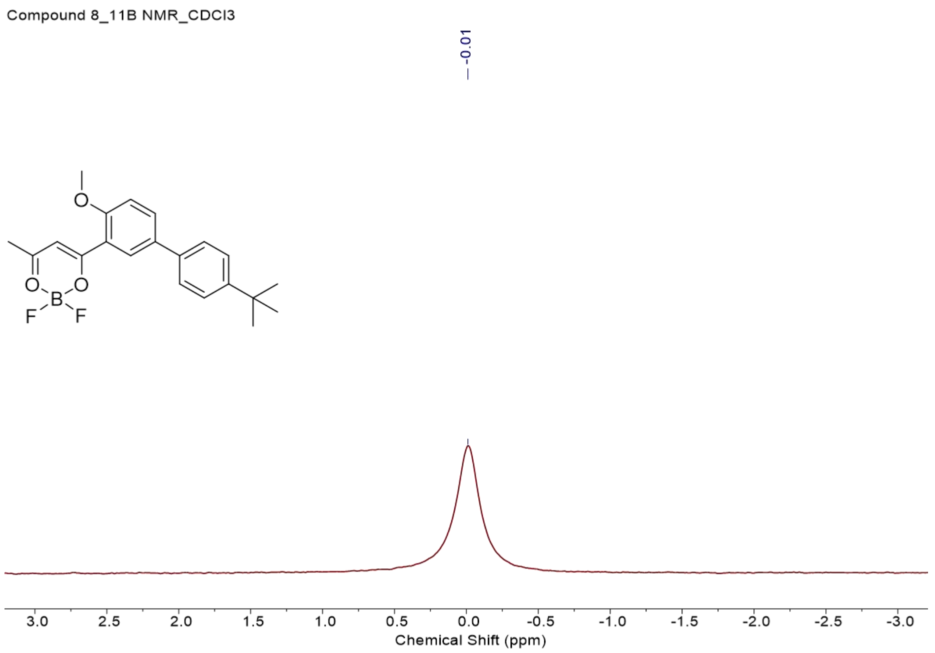

Figure S96. ^{11}B NMR spectra of compound **8**.

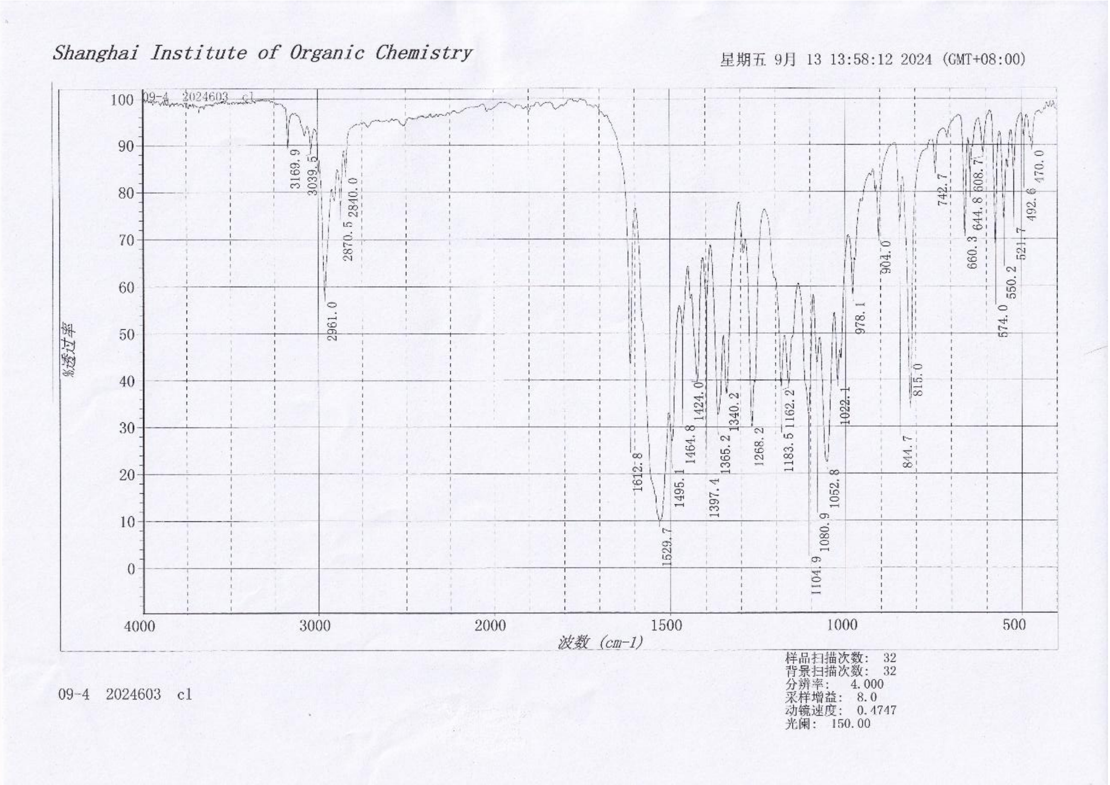

Figure S97. FT-IR spectrum of compound **8**.

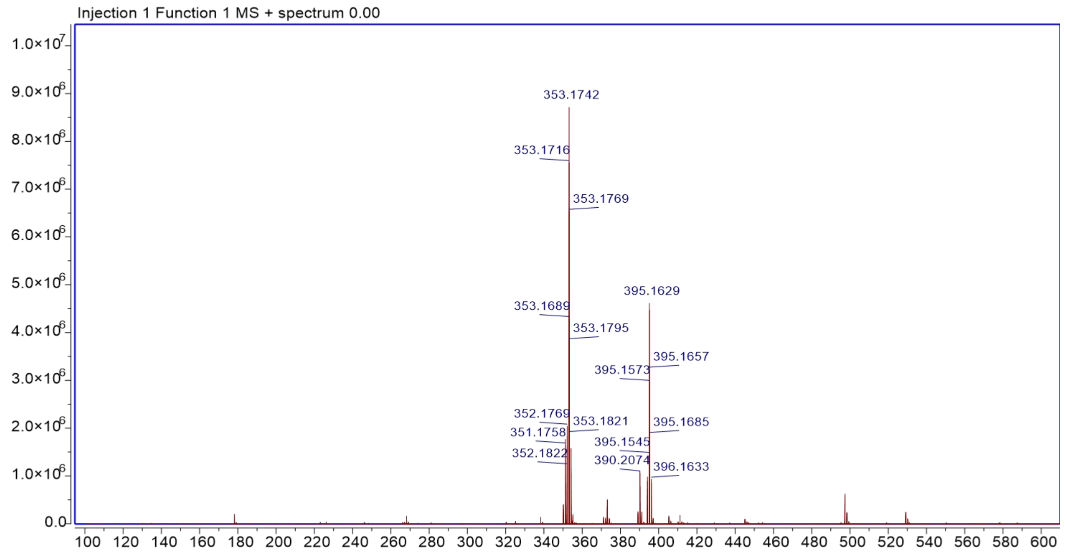

Figure S98. HRMS spectra of compound **8**. The additional signal peaks that are 42 units higher than the $[M\text{-}F]^{+}$ peak can be assigned to $[M\text{-}F]^{+}$ with acetonitrile.

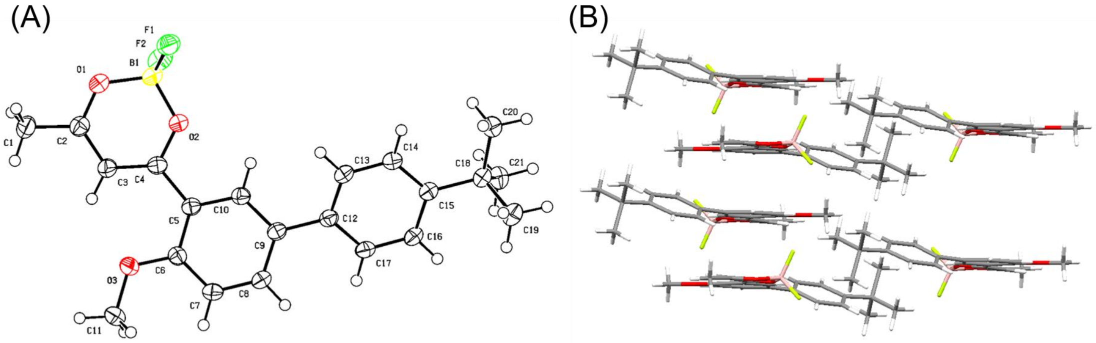

Figure S99. Single crystal structures of compound **8**: (A) monomer, (B) lattice structure; its depository number in CCDC is 2391577.

Table S21. Crystal data and structure refinement for compound **8**.

Identification code	mj24893_0m	
Empirical formula	C21 H23 B F2 O3	
Formula weight	372.20	
Temperature	170.00 K	
Wavelength	1.34139 Å	
Crystal system	Triclinic	
Space group	P-1	
Unit cell dimensions	a = 8.1245(2) Å	a= 85.0110(10)°.
	b = 10.2910(3) Å	b= 84.7540(10)°.
	c = 11.7517(3) Å	g = 75.4220(10)°.

Volume	944.85(4) Å^3
Z	2
Density (calculated)	1.308 Mg/m^3
Absorption coefficient	0.520 mm^{-1}
F(000)	392
Crystal size	0.17 x 0.17 x 0.05 mm^3
Theta range for data collection	3.870 to 54.860°.
Index ranges	-9<=h<=9, -12<=k<=12, -14<=l<=14
Reflections collected	12246
Independent reflections	3525 [R(int) = 0.0330]
Completeness to theta = 53.594°	98.4 %
Absorption correction	Semi-empirical from equivalents
Max. and min. transmission	0.7508 and 0.6552
Refinement method	Full-matrix least-squares on F^2
Data / restraints / parameters	3525 / 0 / 249
Goodness-of-fit on F^2	1.055
Final R indices [I>2sigma(I)]	R1 = 0.0433, wR2 = 0.1117
R indices (all data)	R1 = 0.0489, wR2 = 0.1165
Extinction coefficient	n/a
Largest diff. peak and hole	0.324 and -0.237 e.Å^{-3}

Table S22. Atomic coordinates (x 10^4) and equivalent isotropic displacement parameters (Å^{2}x 10^3) for compound **8**. U(eq) is defined as one third of the trace of the orthogonalized U^{ij} tensor.

	x	y	z	U(eq)
F(1)	12178(1)	1339(1)	7340(1)	41(1)
F(2)	10224(1)	262(1)	6916(1)	44(1)
O(1)	12349(2)	588(1)	5521(1)	42(1)
O(2)	10044(1)	2461(1)	6178(1)	40(1)
O(3)	8980(1)	4424(1)	2962(1)	33(1)
C(1)	13244(2)	329(2)	3578(1)	39(1)
C(2)	12047(2)	1052(2)	4481(1)	31(1)
C(3)	10747(2)	2173(2)	4237(1)	32(1)
C(4)	9784(2)	2887(1)	5114(1)	26(1)

C(5)	8460(2)	4160(1)	4988(1)	25(1)
C(6)	8045(2)	4890(1)	3930(1)	26(1)
C(7)	6736(2)	6060(1)	3918(1)	29(1)
C(8)	5899(2)	6552(1)	4928(1)	28(1)
C(9)	6317(2)	5894(1)	5994(1)	26(1)
C(10)	7575(2)	4692(1)	5990(1)	26(1)
C(11)	8511(2)	5122(2)	1886(1)	38(1)
C(12)	5498(2)	6465(1)	7081(1)	27(1)
C(13)	6350(2)	6191(2)	8083(1)	33(1)
C(14)	5586(2)	6694(2)	9114(1)	35(1)
C(15)	3932(2)	7511(1)	9191(1)	30(1)
C(16)	3105(2)	7826(1)	8175(1)	31(1)
C(17)	3857(2)	7317(1)	7149(1)	29(1)
C(18)	2994(2)	8033(2)	10315(1)	34(1)
C(19)	2459(2)	9582(2)	10214(2)	42(1)
C(20)	4086(2)	7577(2)	11340(1)	43(1)
C(21)	1400(2)	7478(2)	10546(1)	42(1)
B(1)	11195(2)	1145(2)	6519(2)	34(1)

Table S23. Bond lengths [Å] and angles [°] for compound **8**.

F(1)-B(1)	1.365(2)
F(2)-B(1)	1.374(2)
O(1)-C(2)	1.2949(18)
O(1)-B(1)	1.492(2)
O(2)-C(4)	1.3064(17)
O(2)-B(1)	1.4855(19)
O(3)-C(6)	1.3618(17)
O(3)-C(11)	1.4329(17)
C(1)-H(1A)	0.9800
C(1)-H(1B)	0.9800
C(1)-H(1C)	0.9800
C(1)-C(2)	1.487(2)
C(2)-C(3)	1.383(2)
C(3)-H(3)	0.9500
C(3)-C(4)	1.382(2)
C(4)-C(5)	1.4766(19)
C(5)-C(6)	1.4174(19)

C(5)-C(10)	1.4003(19)
C(6)-C(7)	1.392(2)
C(7)-H(7)	0.9500
C(7)-C(8)	1.382(2)
C(8)-H(8)	0.9500
C(8)-C(9)	1.400(2)
C(9)-C(10)	1.3926(19)
C(9)-C(12)	1.4827(19)
C(10)-H(10)	0.9500
C(11)-H(11A)	0.9800
C(11)-H(11B)	0.9800
C(11)-H(11C)	0.9800
C(12)-C(13)	1.392(2)
C(12)-C(17)	1.3992(19)
C(13)-H(13)	0.9500
C(13)-C(14)	1.390(2)
C(14)-H(14)	0.9500
C(14)-C(15)	1.394(2)
C(15)-C(16)	1.397(2)
C(15)-C(18)	1.532(2)
C(16)-H(16)	0.9500
C(16)-C(17)	1.385(2)
C(17)-H(17)	0.9500
C(18)-C(19)	1.540(2)
C(18)-C(20)	1.532(2)
C(18)-C(21)	1.536(2)
C(19)-H(19A)	0.9800
C(19)-H(19B)	0.9800
C(19)-H(19C)	0.9800
C(20)-H(20A)	0.9800
C(20)-H(20B)	0.9800
C(20)-H(20C)	0.9800
C(21)-H(21A)	0.9800
C(21)-H(21B)	0.9800
C(21)-H(21C)	0.9800
C(2)-O(1)-B(1)	121.55(12)
C(4)-O(2)-B(1)	123.45(12)

C(6)-O(3)-C(11)	118.17(11)
H(1A)-C(1)-H(1B)	109.5
H(1A)-C(1)-H(1C)	109.5
H(1B)-C(1)-H(1C)	109.5
C(2)-C(1)-H(1A)	109.5
C(2)-C(1)-H(1B)	109.5
C(2)-C(1)-H(1C)	109.5
O(1)-C(2)-C(1)	115.10(13)
O(1)-C(2)-C(3)	122.11(13)
C(3)-C(2)-C(1)	122.74(14)
C(2)-C(3)-H(3)	119.9
C(4)-C(3)-C(2)	120.27(14)
C(4)-C(3)-H(3)	119.9
O(2)-C(4)-C(3)	119.79(13)
O(2)-C(4)-C(5)	113.72(12)
C(3)-C(4)-C(5)	126.47(13)
C(6)-C(5)-C(4)	124.68(12)
C(10)-C(5)-C(4)	117.40(12)
C(10)-C(5)-C(6)	117.91(12)
O(3)-C(6)-C(5)	118.06(12)
O(3)-C(6)-C(7)	122.56(12)
C(7)-C(6)-C(5)	119.37(13)
C(6)-C(7)-H(7)	119.6
C(8)-C(7)-C(6)	120.75(13)
C(8)-C(7)-H(7)	119.6
C(7)-C(8)-H(8)	119.1
C(7)-C(8)-C(9)	121.73(13)
C(9)-C(8)-H(8)	119.1
C(8)-C(9)-C(12)	121.80(12)
C(10)-C(9)-C(8)	116.85(13)
C(10)-C(9)-C(12)	121.33(12)
C(5)-C(10)-H(10)	118.4
C(9)-C(10)-C(5)	123.28(13)
C(9)-C(10)-H(10)	118.4
O(3)-C(11)-H(11A)	109.5
O(3)-C(11)-H(11B)	109.5
O(3)-C(11)-H(11C)	109.5
H(11A)-C(11)-H(11B)	109.5

H(11A)-C(11)-H(11C)	109.5
H(11B)-C(11)-H(11C)	109.5
C(13)-C(12)-C(9)	121.10(12)
C(13)-C(12)-C(17)	116.86(13)
C(17)-C(12)-C(9)	122.03(12)
C(12)-C(13)-H(13)	119.1
C(14)-C(13)-C(12)	121.77(13)
C(14)-C(13)-H(13)	119.1
C(13)-C(14)-H(14)	119.3
C(13)-C(14)-C(15)	121.45(14)
C(15)-C(14)-H(14)	119.3
C(14)-C(15)-C(16)	116.63(13)
C(14)-C(15)-C(18)	123.66(13)
C(16)-C(15)-C(18)	119.69(12)
C(15)-C(16)-H(16)	119.0
C(17)-C(16)-C(15)	122.03(13)
C(17)-C(16)-H(16)	119.0
C(12)-C(17)-H(17)	119.4
C(16)-C(17)-C(12)	121.20(13)
C(16)-C(17)-H(17)	119.4
C(15)-C(18)-C(19)	109.85(13)
C(15)-C(18)-C(20)	112.52(12)
C(15)-C(18)-C(21)	108.47(13)
C(20)-C(18)-C(19)	108.64(13)
C(20)-C(18)-C(21)	107.79(14)
C(21)-C(18)-C(19)	109.52(13)
C(18)-C(19)-H(19A)	109.5
C(18)-C(19)-H(19B)	109.5
C(18)-C(19)-H(19C)	109.5
H(19A)-C(19)-H(19B)	109.5
H(19A)-C(19)-H(19C)	109.5
H(19B)-C(19)-H(19C)	109.5
C(18)-C(20)-H(20A)	109.5
C(18)-C(20)-H(20B)	109.5
C(18)-C(20)-H(20C)	109.5
H(20A)-C(20)-H(20B)	109.5
H(20A)-C(20)-H(20C)	109.5
H(20B)-C(20)-H(20C)	109.5

C(18)-C(21)-H(21A)	109.5
C(18)-C(21)-H(21B)	109.5
C(18)-C(21)-H(21C)	109.5
H(21A)-C(21)-H(21B)	109.5
H(21A)-C(21)-H(21C)	109.5
H(21B)-C(21)-H(21C)	109.5
F(1)-B(1)-F(2)	111.49(13)
F(1)-B(1)-O(1)	108.23(13)
F(1)-B(1)-O(2)	108.52(13)
F(2)-B(1)-O(1)	109.14(13)
F(2)-B(1)-O(2)	108.84(13)
O(2)-B(1)-O(1)	110.63(12)

Symmetry transformations used to generate equivalent atoms:

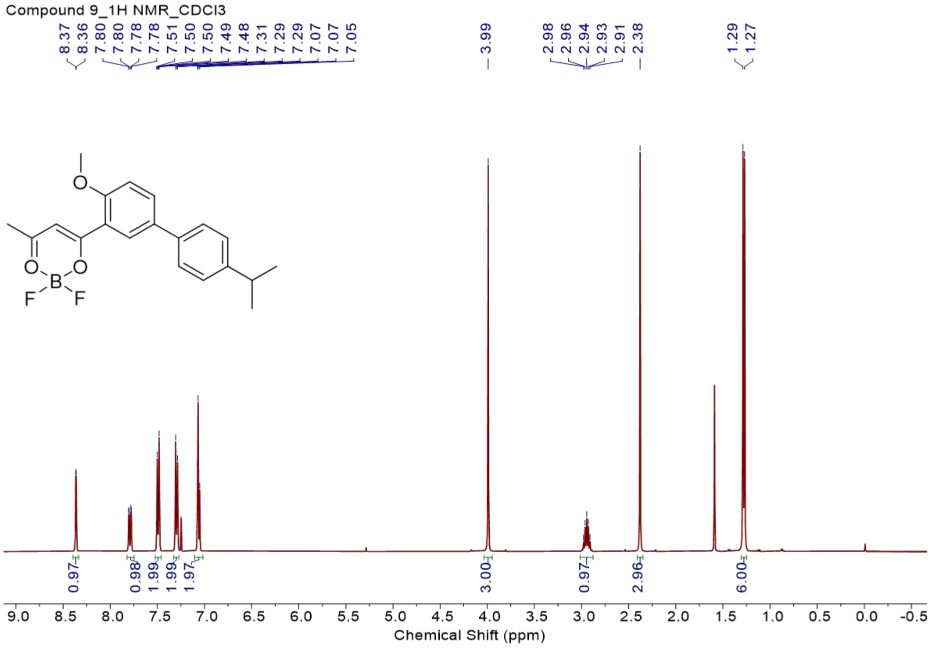

Figure S100. ^{1}H NMR spectra of compound **9**.

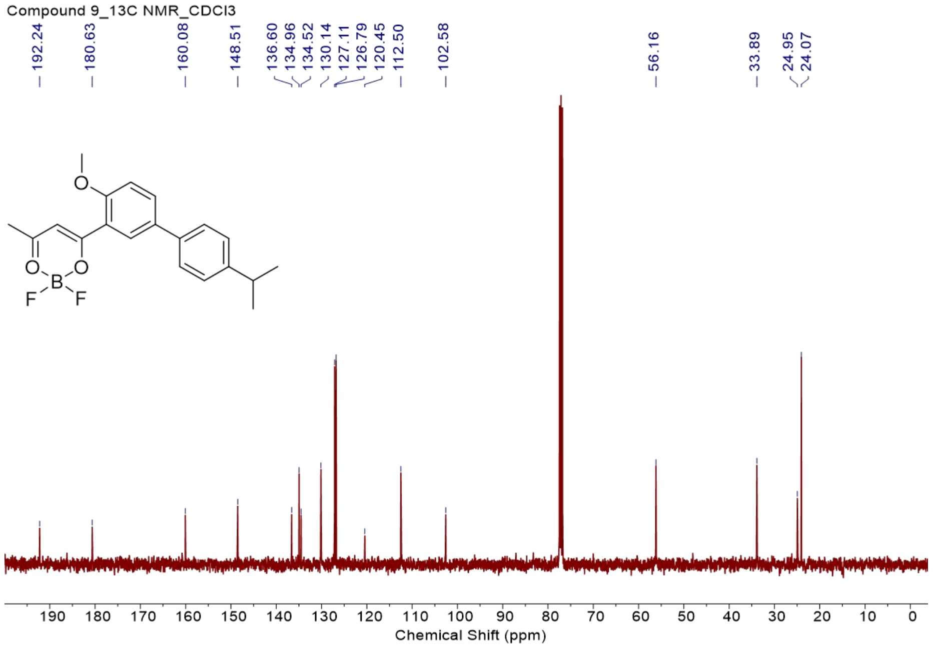

Figure S101. ^{13}C NMR spectra of compound **9**.

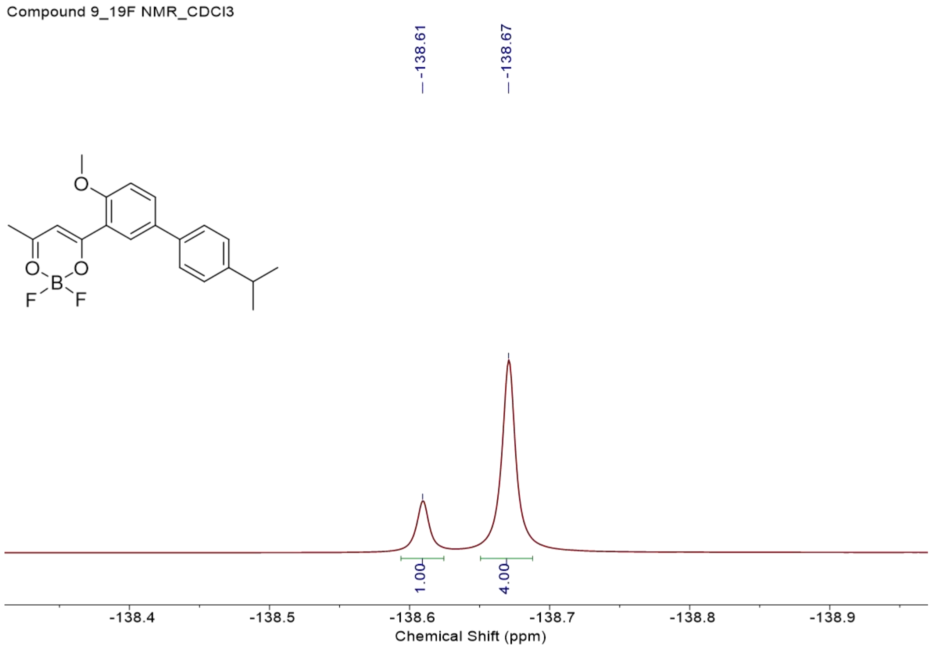

Figure S102. ^{19}F NMR spectra of compound **9**.

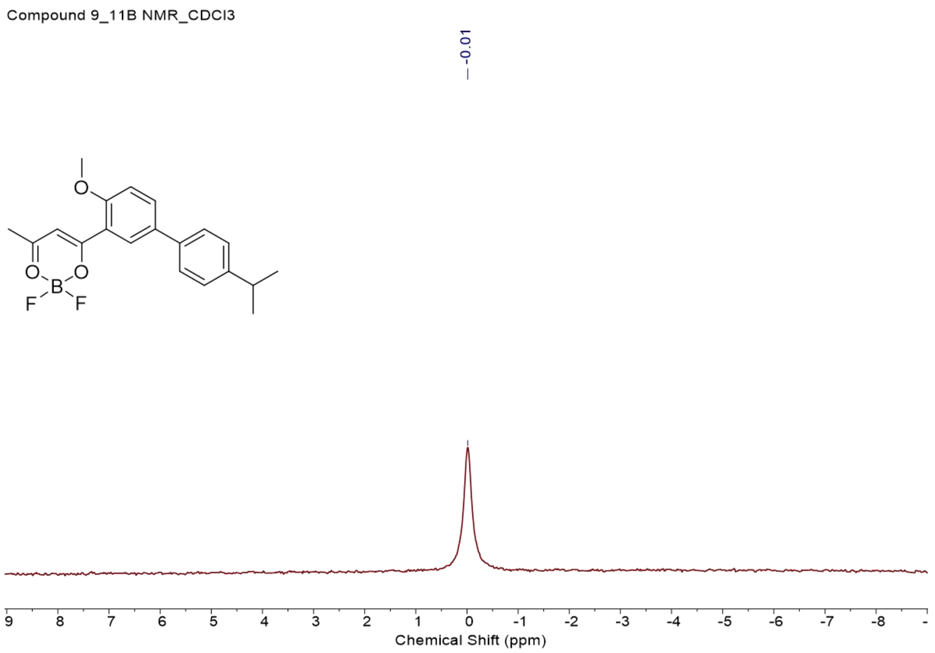

Figure S103. ^{11}B NMR spectra of compound **9**.

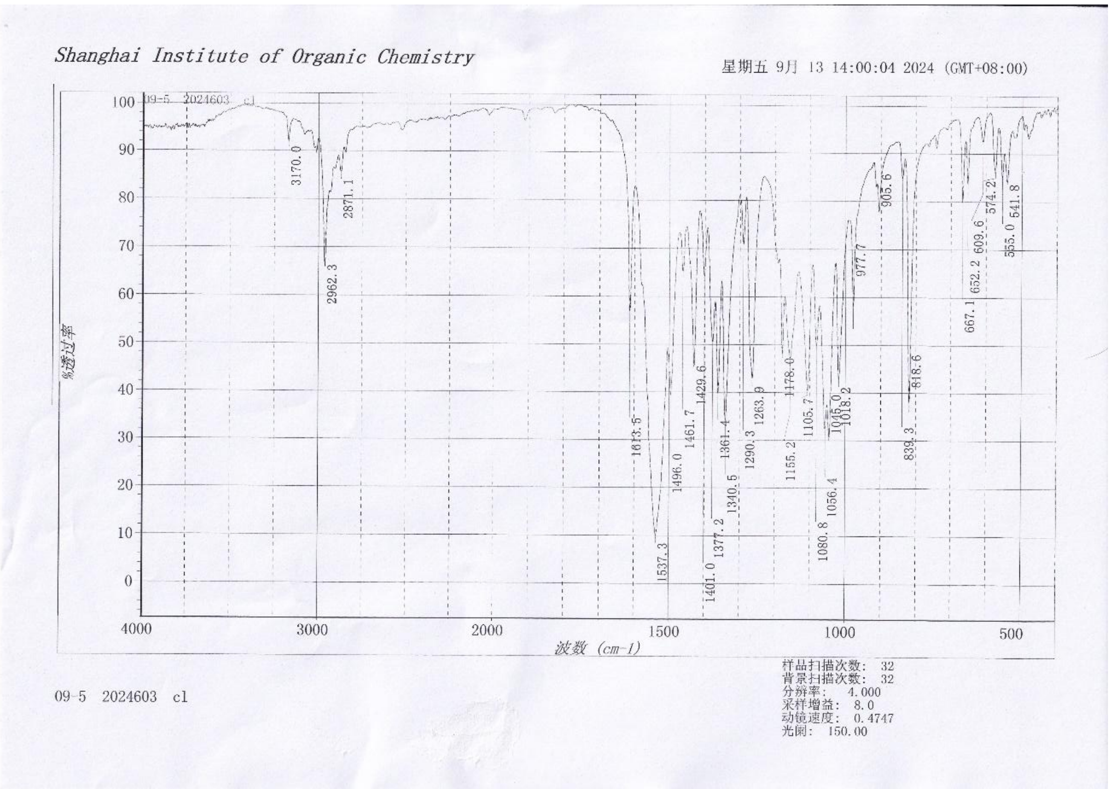

Figure S104. FT-IR spectrum of compound **9**.

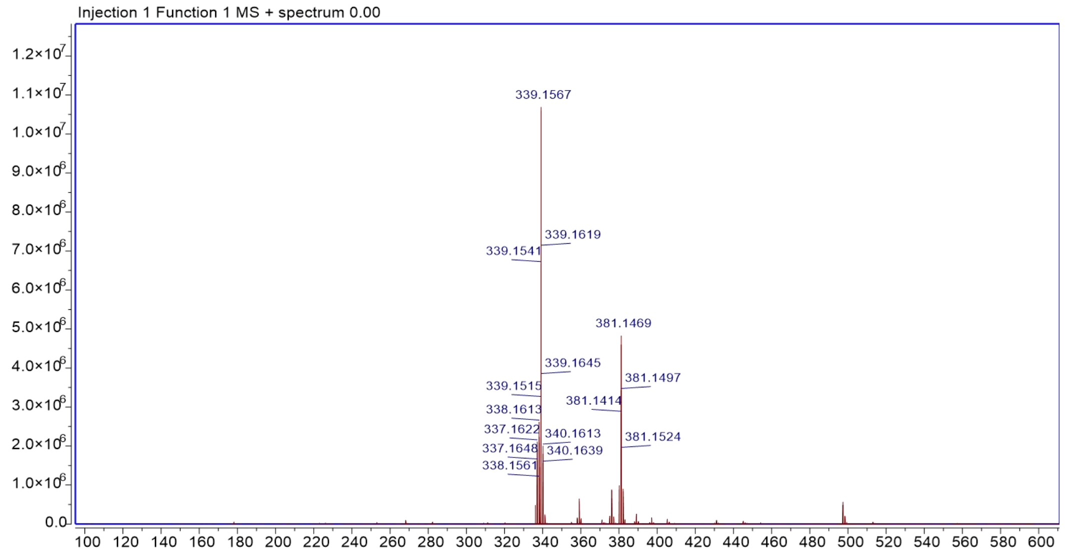

Figure S105. HRMS spectra of compound **9**. The additional signal peaks that are 42 units higher than the [M-F]$^+$ peak can be assigned to [M-F]$^+$ with acetonitrile.

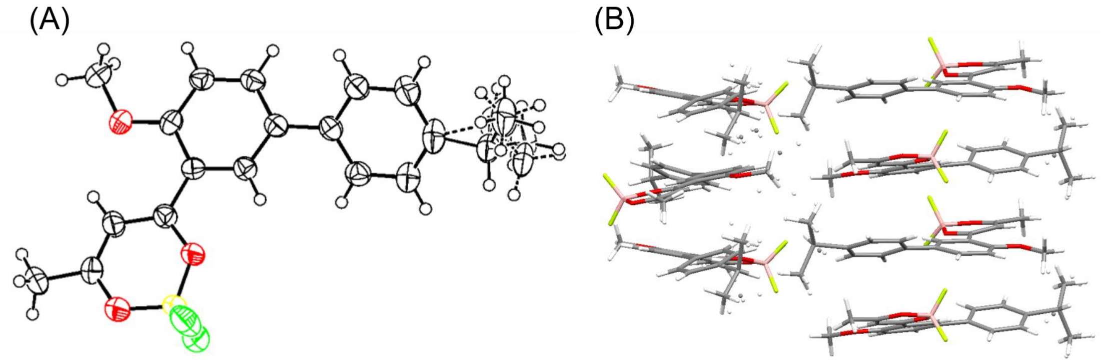

Figure S106. Single crystal structures of compound **9**: (A) monomer, (B) lattice structure; its depository number in CCDC is 2391578.

Table S24. Crystal data and structure refinement for compound **9**.

Identification code	mj24875_0m_4	
Empirical formula	C20 H21 B F2 O3	
Formula weight	358.18	
Temperature	170.00 K	
Wavelength	1.34139 Å	
Crystal system	Monoclinic	
Space group	P 1 21 1	
Unit cell dimensions	a = 7.2858(3) Å	a= 90°.
	b = 13.2573(5) Å	b= 91.570(2)°.
	c = 18.4680(7) Å	g = 90°.

Volume	1783.16(12) Å^3
Z	4
Density (calculated)	1.334 Mg/m^3
Absorption coefficient	0.538 mm^{-1}
F(000)	752
Crystal size	0.17 x 0.17 x 0.05 mm^3
Theta range for data collection	3.571 to 54.957°.
Index ranges	-8<=h<=8, 0<=k<=16, 0<=l<=22
Reflections collected	3436
Independent reflections	3436 [R(int) = ?]
Completeness to theta = 53.594°	97.0 %
Absorption correction	None
Refinement method	Full-matrix least-squares on F^2
Data / restraints / parameters	3436 / 107 / 518
Goodness-of-fit on F^2	1.059
Final R indices [I>2sigma(I)]	R1 = 0.0532, wR2 = 0.1377
R indices (all data)	R1 = 0.0693, wR2 = 0.1542
Absolute structure parameter	-0.2(2)
Extinction coefficient	n/a
Largest diff. peak and hole	0.189 and -0.245 e.Å^{-3}

Table S25. Atomic coordinates (x 10^4) and equivalent isotropic displacement parameters (Å^{2}x 10^3) for compound **9**. U(eq) is defined as one third of the trace of the orthogonalized U^{ij} tensor.

	x	y	z	U(eq)
F(1)	2355(5)	5697(3)	6290(2)	74(1)
F(2)	5386(6)	5945(3)	6118(2)	72(1)
O(1)	4051(6)	6651(3)	7116(2)	64(1)
O(2)	4498(6)	4830(3)	7004(2)	60(1)
O(3)	5168(4)	4207(3)	9202(2)	46(1)
C(1)	3852(9)	7475(5)	8242(3)	59(1)
C(2)	4001(7)	6522(4)	7815(3)	46(1)
C(3)	4163(7)	5586(4)	8120(3)	50(1)
C(4)	4456(6)	4746(4)	7703(2)	38(1)

C(5)	4790(6)	3706(4)	7967(2)	37(1)
C(6)	5141(6)	3452(4)	8701(2)	40(1)
C(7)	5397(6)	2443(4)	8884(3)	43(1)
C(8)	5348(6)	1698(4)	8364(3)	44(1)
C(9)	4998(6)	1919(4)	7626(3)	39(1)
C(10)	4756(6)	2935(4)	7448(2)	39(1)
C(11)	5511(8)	3944(5)	9947(3)	55(1)
C(12)	4937(6)	1129(4)	7065(2)	39(1)
C(13)	5427(7)	1342(4)	6353(3)	47(1)
C(14)	5325(7)	613(5)	5816(3)	56(2)
C(15)	4748(7)	-355(5)	5964(3)	64(2)
C(16)	4309(8)	-582(5)	6672(3)	60(2)
C(17)	4380(7)	152(4)	7215(3)	50(1)
C(18)	4489(14)	-962(9)	5242(6)	53(2)
C(19)	6335(9)	-1373(6)	5014(4)	74(2)
C(20)	2997(15)	-1765(8)	5359(6)	49(3)
B(1)	4057(10)	5782(5)	6615(3)	53(2)
F(1A)	2660(5)	4186(3)	1251(2)	71(1)
F(2A)	-420(5)	3956(3)	1164(2)	69(1)
O(1A)	1232(6)	3250(3)	2128(2)	64(1)
O(2A)	805(6)	5077(3)	2005(2)	60(1)
O(3A)	-206(5)	5691(3)	4183(2)	47(1)
C(1A)	1001(9)	2406(5)	3237(3)	59(2)
C(2A)	911(7)	3363(4)	2815(3)	45(1)
C(3A)	545(7)	4279(4)	3118(3)	49(1)
C(4A)	505(6)	5149(4)	2701(3)	40(1)
C(5A)	171(6)	6179(4)	2955(2)	35(1)
C(6A)	-167(6)	6439(4)	3683(2)	40(1)
C(7A)	-406(6)	7453(4)	3861(2)	41(1)
C(8A)	-323(6)	8199(4)	3339(3)	42(1)
C(9A)	24(6)	7974(4)	2611(3)	39(1)
C(10A)	249(6)	6958(4)	2443(2)	38(1)
C(11A)	-487(8)	5957(5)	4923(3)	58(1)
C(12A)	115(6)	8758(4)	2043(2)	40(1)
C(13A)	-424(6)	8539(4)	1326(3)	46(1)
C(14A)	-276(7)	9243(5)	786(3)	52(1)
C(15A)	409(7)	10210(5)	929(3)	59(2)
C(16A)	903(7)	10437(5)	1643(3)	56(1)

C(17A)	761(7)	9726(4)	2190(3)	48(1)
C(18A)	664(11)	10871(7)	255(5)	51(2)
C(19A)	-1176(17)	11393(11)	97(8)	67(4)
C(20A)	2146(9)	11603(6)	336(4)	76(2)
B(1A)	1072(9)	4136(5)	1613(3)	51(1)
C(21)	4813(16)	-1383(10)	5518(7)	53(3)
C(1B)	2931(19)	-1344(15)	5061(9)	76(4)
C(18B)	300(20)	11248(16)	511(12)	61(4)
C(19B)	-1100(40)	11150(20)	-98(15)	58(6)

Table S26. Bond lengths [Å] and angles [°] for compound **9**.

F(1)-B(1)	1.368(7)
F(2)-B(1)	1.371(8)
O(1)-C(2)	1.304(6)
O(1)-B(1)	1.478(7)
O(2)-C(4)	1.296(6)
O(2)-B(1)	1.483(7)
O(3)-C(6)	1.363(6)
O(3)-C(11)	1.436(6)
C(1)-H(1A)	0.9800
C(1)-H(1B)	0.9800
C(1)-H(1C)	0.9800
C(1)-C(2)	1.494(8)
C(2)-C(3)	1.366(8)
C(3)-H(3)	0.9500
C(3)-C(4)	1.375(7)
C(4)-C(5)	1.480(7)
C(5)-C(6)	1.413(6)
C(5)-C(10)	1.402(7)
C(6)-C(7)	1.390(7)
C(7)-H(7)	0.9500
C(7)-C(8)	1.376(7)
C(8)-H(8)	0.9500
C(8)-C(9)	1.411(7)
C(9)-C(10)	1.397(7)
C(9)-C(12)	1.473(7)
C(10)-H(10)	0.9500

C(11)-H(11A)	0.9800
C(11)-H(11B)	0.9800
C(11)-H(11C)	0.9800
C(12)-C(13)	1.401(7)
C(12)-C(17)	1.387(8)
C(13)-H(13)	0.9500
C(13)-C(14)	1.385(7)
C(14)-H(14)	0.9500
C(14)-C(15)	1.380(10)
C(15)-C(16)	1.387(9)
C(15)-C(18)	1.564(11)
C(15)-C(21)	1.594(12)
C(16)-H(16)	0.9500
C(16)-C(17)	1.397(8)
C(17)-H(17)	0.9500
C(18)-H(18)	1.0000
C(18)-C(19)	1.521(11)
C(18)-C(20)	1.541(12)
C(19)-H(19A)	0.9800
C(19)-H(19B)	0.9800
C(19)-H(19C)	0.9800
C(19)-H(19D)	0.9800
C(19)-H(19E)	0.9800
C(19)-H(19F)	0.9800
C(19)-C(21)	1.467(12)
C(20)-H(20A)	0.9800
C(20)-H(20B)	0.9800
C(20)-H(20C)	0.9800
F(1A)-B(1A)	1.353(7)
F(2A)-B(1A)	1.370(8)
O(1A)-C(2A)	1.305(6)
O(1A)-B(1A)	1.514(8)
O(2A)-C(4A)	1.314(6)
O(2A)-B(1A)	1.457(7)
O(3A)-C(6A)	1.356(6)
O(3A)-C(11A)	1.431(6)
C(1A)-H(1AA)	0.9800
C(1A)-H(1AB)	0.9800

C(1A)-H(1AC)	0.9800
C(1A)-C(2A)	1.489(7)
C(2A)-C(3A)	1.367(8)
C(3A)-H(3A)	0.9500
C(3A)-C(4A)	1.387(7)
C(4A)-C(5A)	1.466(7)
C(5A)-C(6A)	1.417(6)
C(5A)-C(10A)	1.401(7)
C(6A)-C(7A)	1.395(7)
C(7A)-H(7A)	0.9500
C(7A)-C(8A)	1.383(7)
C(8A)-H(8A)	0.9500
C(8A)-C(9A)	1.407(7)
C(9A)-C(10A)	1.394(7)
C(9A)-C(12A)	1.479(7)
C(10A)-H(10A)	0.9500
C(11A)-H(11D)	0.9800
C(11A)-H(11E)	0.9800
C(11A)-H(11F)	0.9800
C(12A)-C(13A)	1.401(7)
C(12A)-C(17A)	1.391(8)
C(13A)-H(13A)	0.9500
C(13A)-C(14A)	1.373(7)
C(14A)-H(14A)	0.9500
C(14A)-C(15A)	1.397(9)
C(15A)-C(16A)	1.391(9)
C(15A)-C(18A)	1.538(9)
C(15A)-C(18B)	1.580(17)
C(16A)-H(16A)	0.9500
C(16A)-C(17A)	1.387(8)
C(17A)-H(17A)	0.9500
C(18A)-H(18A)	1.0000
C(18A)-C(19A)	1.529(12)
C(18A)-C(20A)	1.457(10)
C(19A)-H(19G)	0.9800
C(19A)-H(19H)	0.9800
C(19A)-H(19I)	0.9800
C(20A)-H(20D)	0.9800

C(20A)-H(20E)	0.9800
C(20A)-H(20F)	0.9800
C(20A)-H(20G)	0.9800
C(20A)-H(20H)	0.9800
C(20A)-H(20I)	0.9800
C(20A)-C(18B)	1.473(17)
C(21)-H(21)	1.0000
C(21)-C(1B)	1.592(15)
C(1B)-H(1BA)	0.9800
C(1B)-H(1BB)	0.9800
C(1B)-H(1BC)	0.9800
C(18B)-H(18B)	1.0000
C(18B)-C(19B)	1.502(18)
C(19B)-H(19J)	0.9800
C(19B)-H(19K)	0.9800
C(19B)-H(19L)	0.9800

C(2)-O(1)-B(1)	121.2(4)
C(4)-O(2)-B(1)	123.0(4)
C(6)-O(3)-C(11)	118.1(4)
H(1A)-C(1)-H(1B)	109.5
H(1A)-C(1)-H(1C)	109.5
H(1B)-C(1)-H(1C)	109.5
C(2)-C(1)-H(1A)	109.5
C(2)-C(1)-H(1B)	109.5
C(2)-C(1)-H(1C)	109.5
O(1)-C(2)-C(1)	114.6(5)
O(1)-C(2)-C(3)	121.5(5)
C(3)-C(2)-C(1)	123.9(5)
C(2)-C(3)-H(3)	119.4
C(2)-C(3)-C(4)	121.2(5)
C(4)-C(3)-H(3)	119.4
O(2)-C(4)-C(3)	119.8(5)
O(2)-C(4)-C(5)	113.6(4)
C(3)-C(4)-C(5)	126.6(4)
C(6)-C(5)-C(4)	124.2(4)
C(10)-C(5)-C(4)	117.0(4)
C(10)-C(5)-C(6)	118.8(4)

O(3)-C(6)-C(5)	118.3(4)
O(3)-C(6)-C(7)	122.9(4)
C(7)-C(6)-C(5)	118.8(4)
C(6)-C(7)-H(7)	119.3
C(8)-C(7)-C(6)	121.3(4)
C(8)-C(7)-H(7)	119.3
C(7)-C(8)-H(8)	119.2
C(7)-C(8)-C(9)	121.7(5)
C(9)-C(8)-H(8)	119.2
C(8)-C(9)-C(12)	122.3(5)
C(10)-C(9)-C(8)	116.5(5)
C(10)-C(9)-C(12)	121.2(4)
C(5)-C(10)-H(10)	118.6
C(9)-C(10)-C(5)	122.8(4)
C(9)-C(10)-H(10)	118.6
O(3)-C(11)-H(11A)	109.5
O(3)-C(11)-H(11B)	109.5
O(3)-C(11)-H(11C)	109.5
H(11A)-C(11)-H(11B)	109.5
H(11A)-C(11)-H(11C)	109.5
H(11B)-C(11)-H(11C)	109.5
C(13)-C(12)-C(9)	120.9(5)
C(17)-C(12)-C(9)	121.8(4)
C(17)-C(12)-C(13)	117.3(5)
C(12)-C(13)-H(13)	119.3
C(14)-C(13)-C(12)	121.4(5)
C(14)-C(13)-H(13)	119.3
C(13)-C(14)-H(14)	119.4
C(15)-C(14)-C(13)	121.2(5)
C(15)-C(14)-H(14)	119.4
C(14)-C(15)-C(16)	117.9(5)
C(14)-C(15)-C(18)	109.9(6)
C(14)-C(15)-C(21)	132.8(7)
C(16)-C(15)-C(18)	131.7(7)
C(16)-C(15)-C(21)	108.2(7)
C(15)-C(16)-H(16)	119.3
C(15)-C(16)-C(17)	121.4(6)
C(17)-C(16)-H(16)	119.3

C(12)-C(17)-C(16)	120.8(5)
C(12)-C(17)-H(17)	119.6
C(16)-C(17)-H(17)	119.6
C(15)-C(18)-H(18)	108.1
C(19)-C(18)-C(15)	109.5(7)
C(19)-C(18)-H(18)	108.1
C(19)-C(18)-C(20)	115.1(9)
C(20)-C(18)-C(15)	107.8(8)
C(20)-C(18)-H(18)	108.1
C(18)-C(19)-H(19A)	109.5
C(18)-C(19)-H(19B)	109.5
C(18)-C(19)-H(19C)	109.5
H(19A)-C(19)-H(19B)	109.5
H(19A)-C(19)-H(19C)	109.5
H(19B)-C(19)-H(19C)	109.5
H(19D)-C(19)-H(19E)	109.5
H(19D)-C(19)-H(19F)	109.5
H(19E)-C(19)-H(19F)	109.5
C(21)-C(19)-H(19D)	109.5
C(21)-C(19)-H(19E)	109.5
C(21)-C(19)-H(19F)	109.5
C(18)-C(20)-H(20A)	109.5
C(18)-C(20)-H(20B)	109.5
C(18)-C(20)-H(20C)	109.5
H(20A)-C(20)-H(20B)	109.5
H(20A)-C(20)-H(20C)	109.5
H(20B)-C(20)-H(20C)	109.5
F(1)-B(1)-F(2)	111.6(5)
F(1)-B(1)-O(1)	108.7(5)
F(1)-B(1)-O(2)	108.9(5)
F(2)-B(1)-O(1)	108.1(5)
F(2)-B(1)-O(2)	108.1(5)
O(1)-B(1)-O(2)	111.4(4)
C(2A)-O(1A)-B(1A)	120.6(5)
C(4A)-O(2A)-B(1A)	125.3(4)
C(6A)-O(3A)-C(11A)	118.4(4)
H(1AA)-C(1A)-H(1AB)	109.5
H(1AA)-C(1A)-H(1AC)	109.5

H(1AB)-C(1A)-H(1AC)	109.5
C(2A)-C(1A)-H(1AA)	109.5
C(2A)-C(1A)-H(1AB)	109.5
C(2A)-C(1A)-H(1AC)	109.5
O(1A)-C(2A)-C(1A)	113.9(5)
O(1A)-C(2A)-C(3A)	122.8(5)
C(3A)-C(2A)-C(1A)	123.3(5)
C(2A)-C(3A)-H(3A)	119.5
C(2A)-C(3A)-C(4A)	120.9(4)
C(4A)-C(3A)-H(3A)	119.5
O(2A)-C(4A)-C(3A)	118.7(5)
O(2A)-C(4A)-C(5A)	114.5(4)
C(3A)-C(4A)-C(5A)	126.8(4)
C(6A)-C(5A)-C(4A)	124.4(4)
C(10A)-C(5A)-C(4A)	117.5(4)
C(10A)-C(5A)-C(6A)	118.2(4)
O(3A)-C(6A)-C(5A)	118.4(4)
O(3A)-C(6A)-C(7A)	122.6(4)
C(7A)-C(6A)-C(5A)	118.9(4)
C(6A)-C(7A)-H(7A)	119.4
C(8A)-C(7A)-C(6A)	121.2(4)
C(8A)-C(7A)-H(7A)	119.4
C(7A)-C(8A)-H(8A)	119.1
C(7A)-C(8A)-C(9A)	121.7(5)
C(9A)-C(8A)-H(8A)	119.1
C(8A)-C(9A)-C(12A)	122.8(5)
C(10A)-C(9A)-C(8A)	116.3(5)
C(10A)-C(9A)-C(12A)	120.9(4)
C(5A)-C(10A)-H(10A)	118.2
C(9A)-C(10A)-C(5A)	123.7(4)
C(9A)-C(10A)-H(10A)	118.2
O(3A)-C(11A)-H(11D)	109.5
O(3A)-C(11A)-H(11E)	109.5
O(3A)-C(11A)-H(11F)	109.5
H(11D)-C(11A)-H(11E)	109.5
H(11D)-C(11A)-H(11F)	109.5
H(11E)-C(11A)-H(11F)	109.5
C(13A)-C(12A)-C(9A)	120.5(5)

C(17A)-C(12A)-C(9A)	122.1(4)
C(17A)-C(12A)-C(13A)	117.4(5)
C(12A)-C(13A)-H(13A)	119.3
C(14A)-C(13A)-C(12A)	121.3(5)
C(14A)-C(13A)-H(13A)	119.3
C(13A)-C(14A)-H(14A)	119.3
C(13A)-C(14A)-C(15A)	121.4(5)
C(15A)-C(14A)-H(14A)	119.3
C(14A)-C(15A)-C(18A)	114.9(6)
C(14A)-C(15A)-C(18B)	133.9(9)
C(16A)-C(15A)-C(14A)	117.3(5)
C(16A)-C(15A)-C(18A)	127.6(6)
C(16A)-C(15A)-C(18B)	106.5(10)
C(15A)-C(16A)-H(16A)	119.3
C(17A)-C(16A)-C(15A)	121.4(6)
C(17A)-C(16A)-H(16A)	119.3
C(12A)-C(17A)-H(17A)	119.5
C(16A)-C(17A)-C(12A)	121.1(5)
C(16A)-C(17A)-H(17A)	119.5
C(15A)-C(18A)-H(18A)	108.3
C(19A)-C(18A)-C(15A)	106.8(8)
C(19A)-C(18A)-H(18A)	108.3
C(20A)-C(18A)-C(15A)	113.8(6)
C(20A)-C(18A)-H(18A)	108.3
C(20A)-C(18A)-C(19A)	111.2(10)
C(18A)-C(19A)-H(19G)	109.5
C(18A)-C(19A)-H(19H)	109.5
C(18A)-C(19A)-H(19I)	109.5
H(19G)-C(19A)-H(19H)	109.5
H(19G)-C(19A)-H(19I)	109.5
H(19H)-C(19A)-H(19I)	109.5
C(18A)-C(20A)-H(20D)	109.5
C(18A)-C(20A)-H(20E)	109.5
C(18A)-C(20A)-H(20F)	109.5
H(20D)-C(20A)-H(20E)	109.5
H(20D)-C(20A)-H(20F)	109.5
H(20E)-C(20A)-H(20F)	109.5
H(20G)-C(20A)-H(20H)	109.5

H(20G)-C(20A)-H(20I)	109.5
H(20H)-C(20A)-H(20I)	109.5
C(18B)-C(20A)-H(20G)	109.5
C(18B)-C(20A)-H(20H)	109.5
C(18B)-C(20A)-H(20I)	109.5
F(1A)-B(1A)-F(2A)	112.6(5)
F(1A)-B(1A)-O(1A)	107.2(5)
F(1A)-B(1A)-O(2A)	109.4(5)
F(2A)-B(1A)-O(1A)	106.9(5)
F(2A)-B(1A)-O(2A)	109.6(5)
O(2A)-B(1A)-O(1A)	111.1(4)
C(15)-C(21)-H(21)	111.7
C(19)-C(21)-C(15)	110.7(8)
C(19)-C(21)-H(21)	111.7
C(19)-C(21)-C(1B)	108.5(11)
C(1B)-C(21)-C(15)	102.0(10)
C(1B)-C(21)-H(21)	111.7
C(21)-C(1B)-H(1BA)	109.5
C(21)-C(1B)-H(1BB)	109.5
C(21)-C(1B)-H(1BC)	109.5
H(1BA)-C(1B)-H(1BB)	109.5
H(1BA)-C(1B)-H(1BC)	109.5
H(1BB)-C(1B)-H(1BC)	109.5
C(15A)-C(18B)-H(18B)	106.5
C(20A)-C(18B)-C(15A)	110.5(12)
C(20A)-C(18B)-H(18B)	106.5
C(20A)-C(18B)-C(19B)	118.0(19)
C(19B)-C(18B)-C(15A)	108.2(17)
C(19B)-C(18B)-H(18B)	106.5
C(18B)-C(19B)-H(19J)	109.5
C(18B)-C(19B)-H(19K)	109.5
C(18B)-C(19B)-H(19L)	109.5
H(19J)-C(19B)-H(19K)	109.5
H(19J)-C(19B)-H(19L)	109.5
H(19K)-C(19B)-H(19L)	109.5

Symmetry transformations used to generate equivalent atoms:

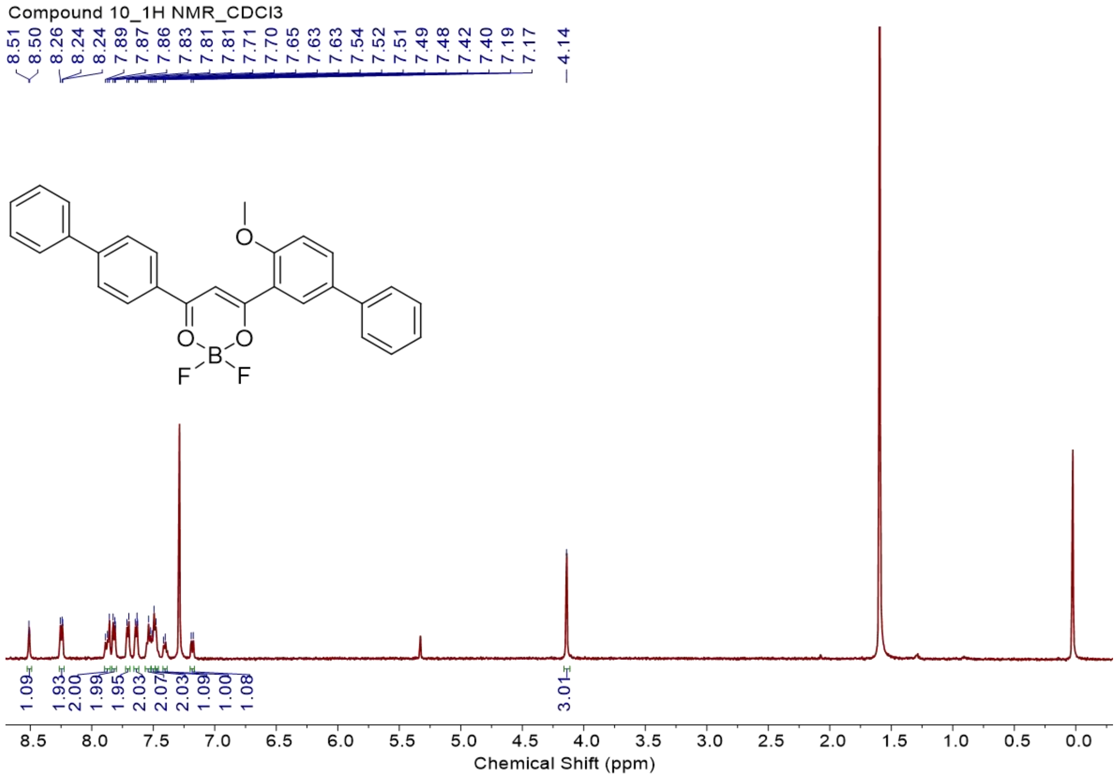

Figure S107. ^{1}H NMR spectra of compound **10**.

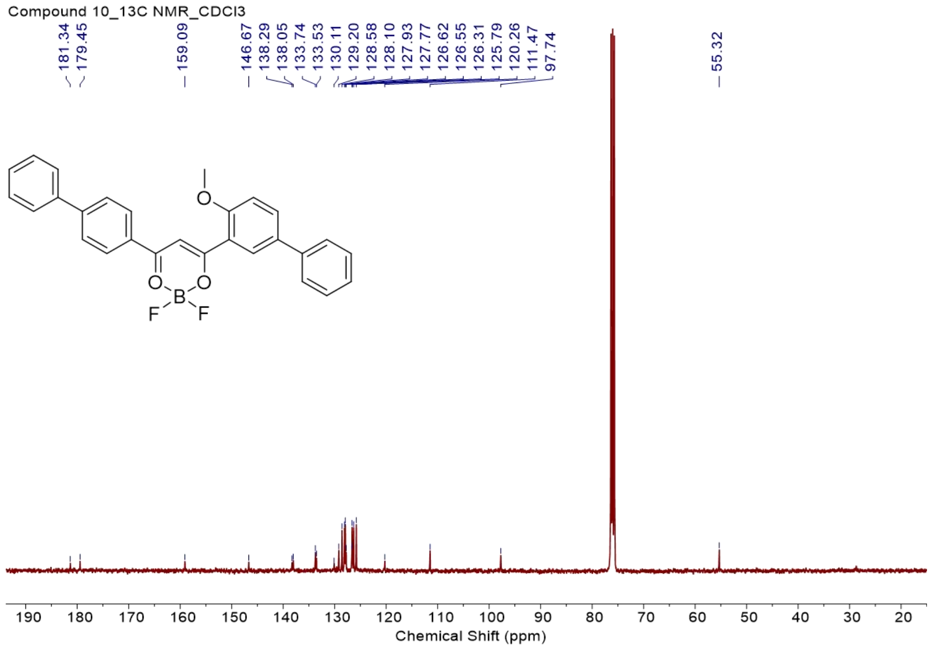

Figure S108. ^{13}C NMR spectra of compound **10**.

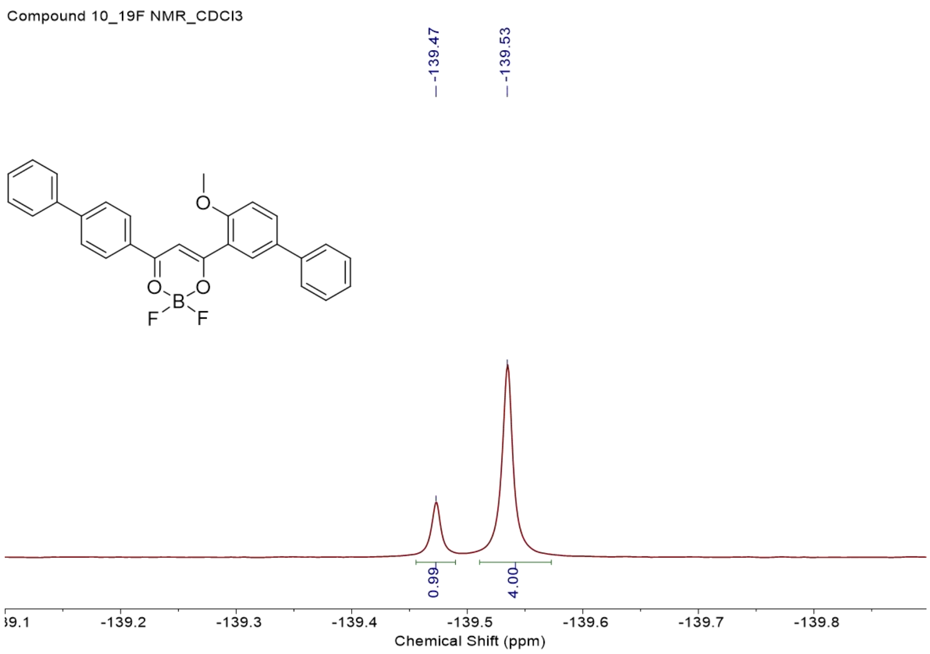

Figure S109. ^{19}F NMR spectra of compound **10**.

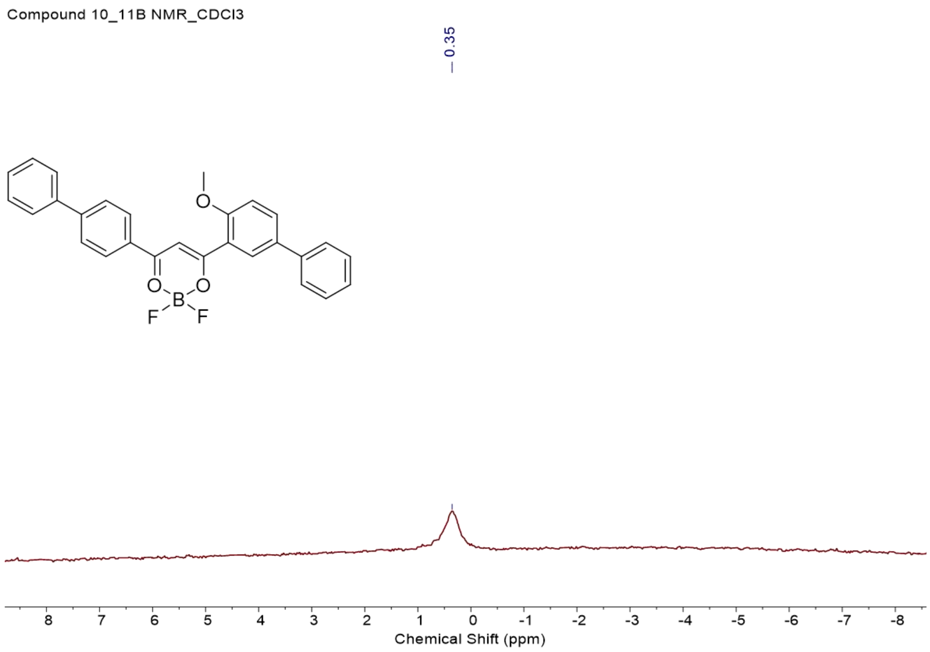

Figure S110. ^{11}B NMR spectra of compound **10**.

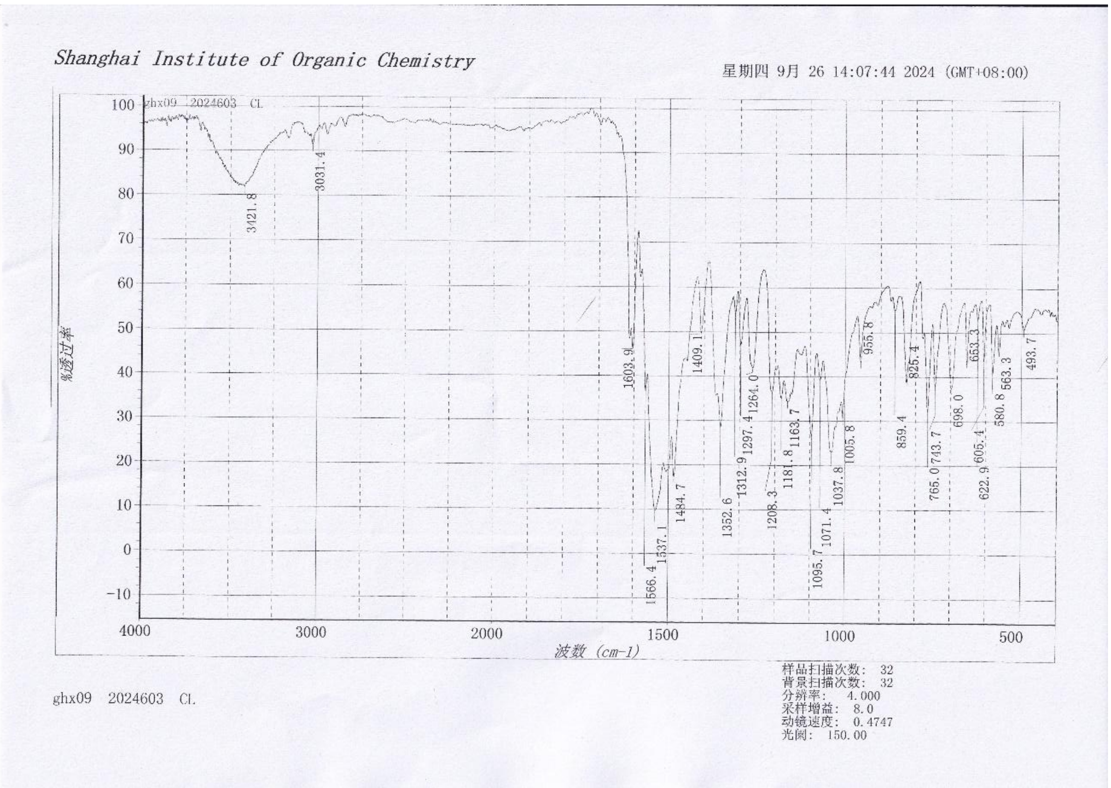

Figure S111. FT-IR spectrum of compound **10**.

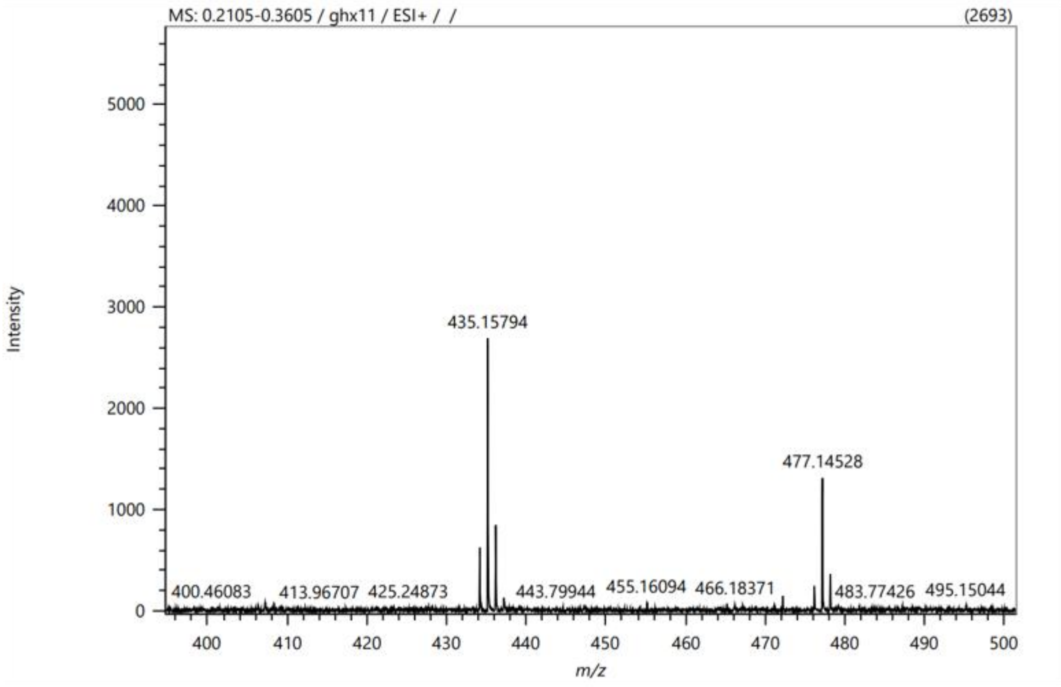

Figure S112. HRMS spectra of compound **10**. The additional signal peaks that are 42 units higher than the $[M-F]^+$ peak can be assigned to $[M-F]^+$ with acetonitrile.

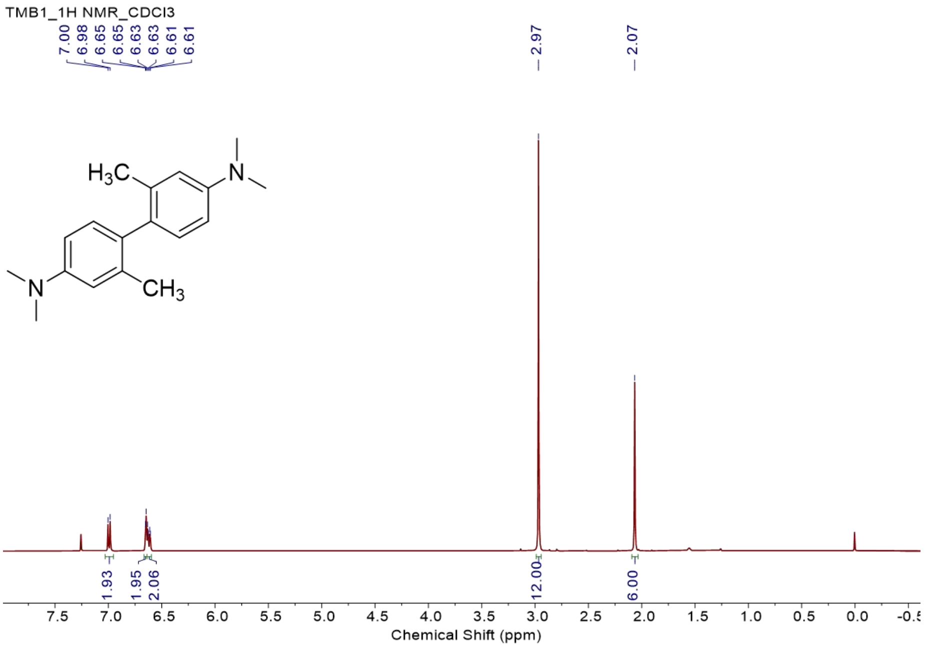

Figure S113. ^{1}H NMR spectra of compound **TMB1**.

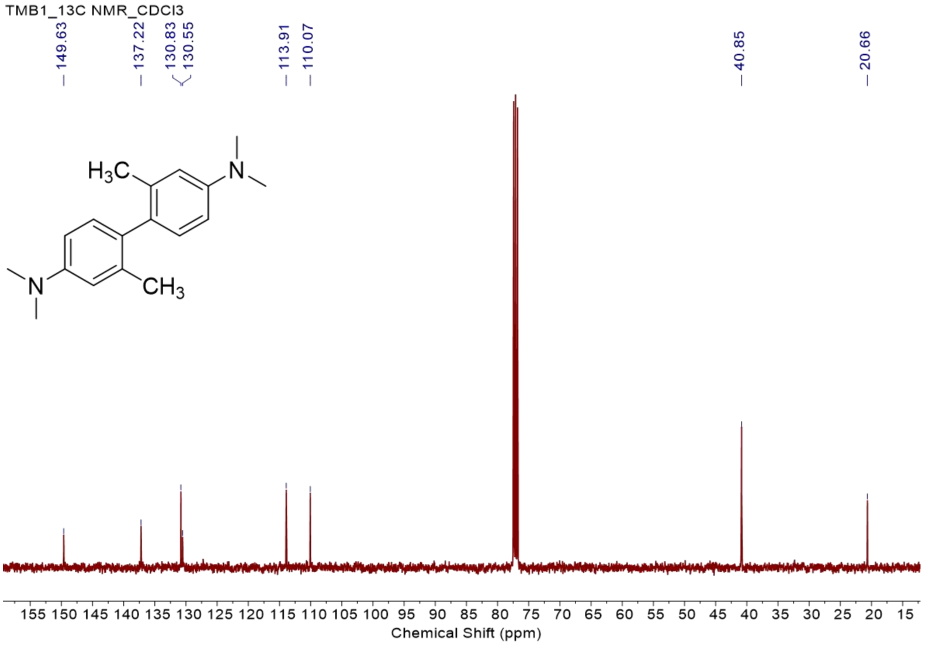

Figure S114. ^{13}C NMR spectra of compound **TMB1**.

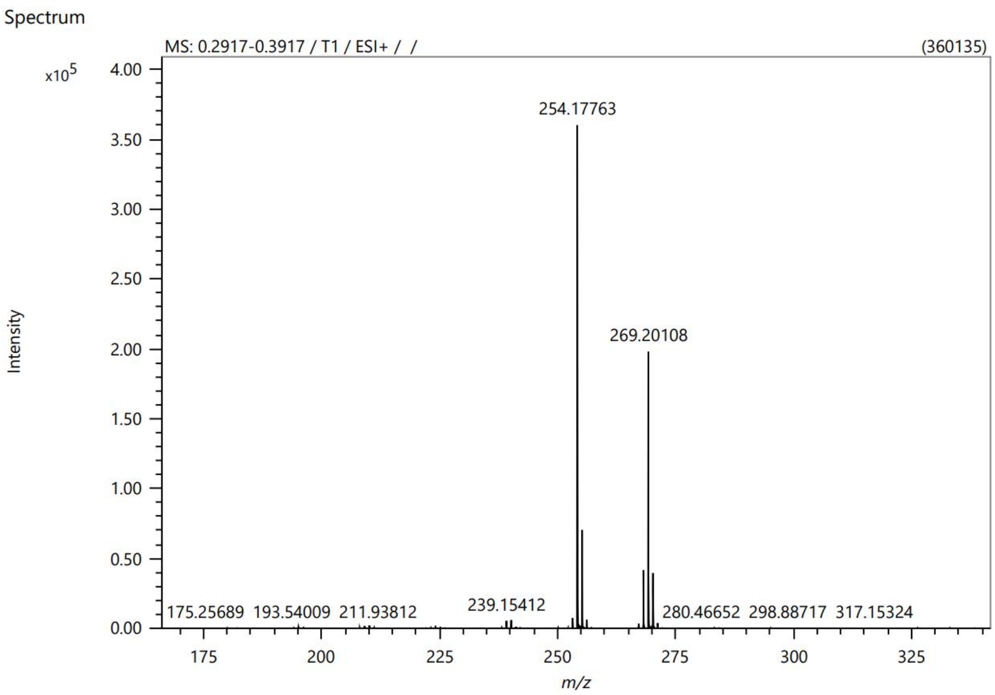

Figure S115. HRMS spectra of compound **TMB1**.

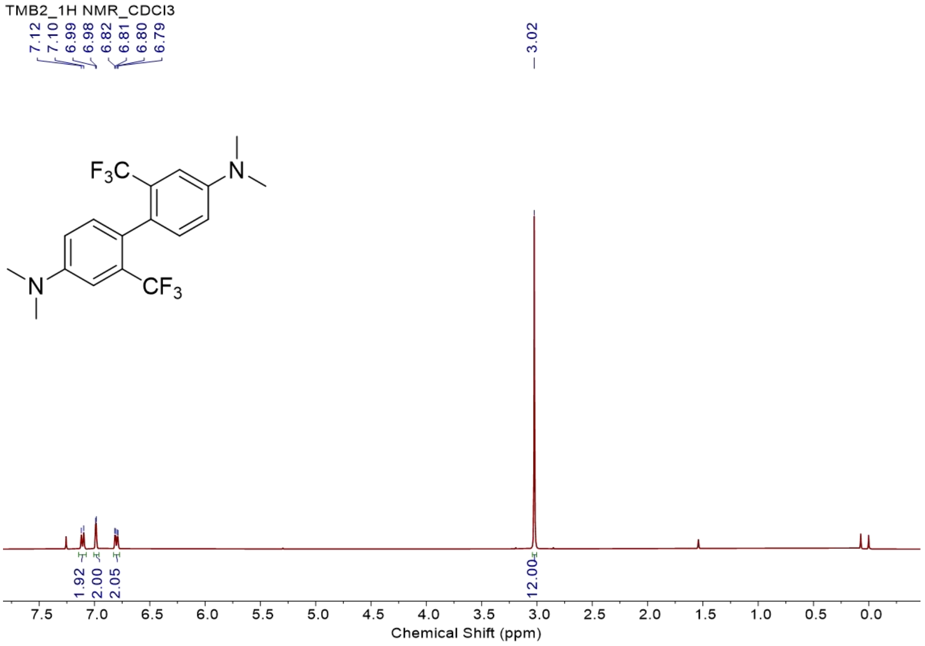

Figure S116. ^{1}H NMR spectra of compound **TMB2**.

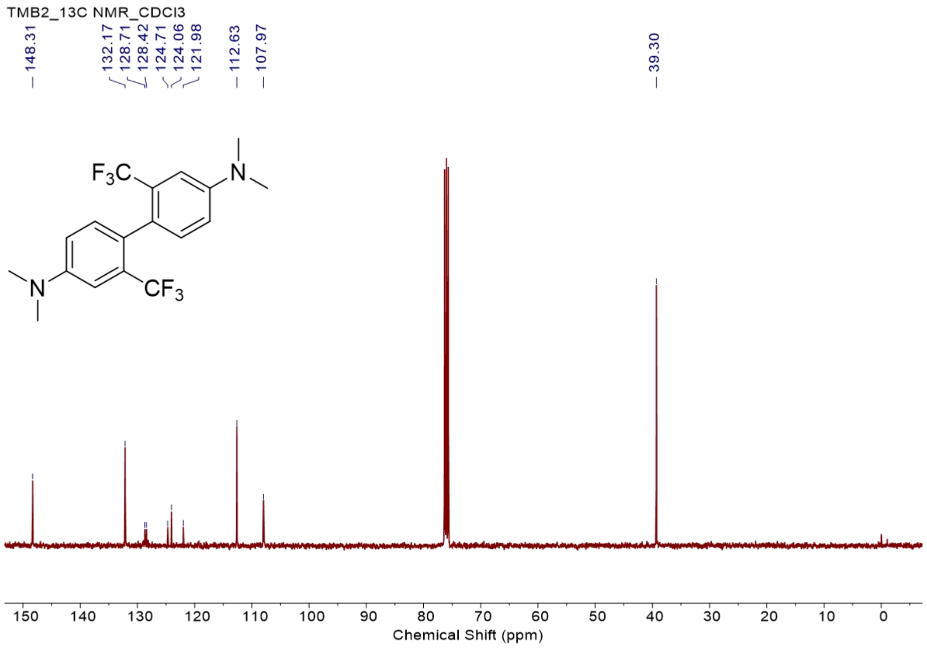

Figure S117. ^{13}C NMR spectra of compound **TMB2**.

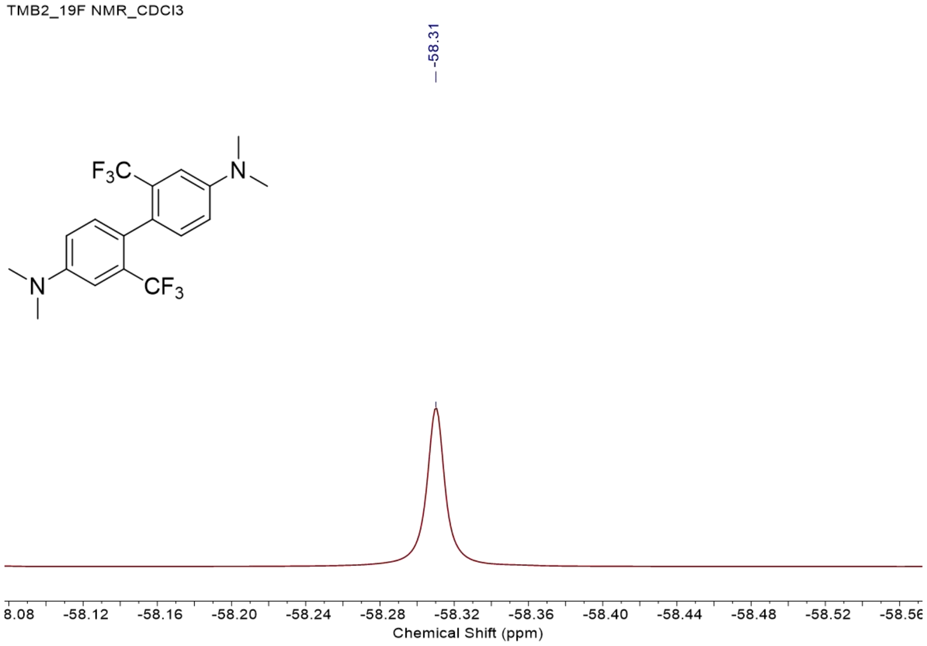

Figure S118. ^{19}F NMR spectra of compound **TMB2**.

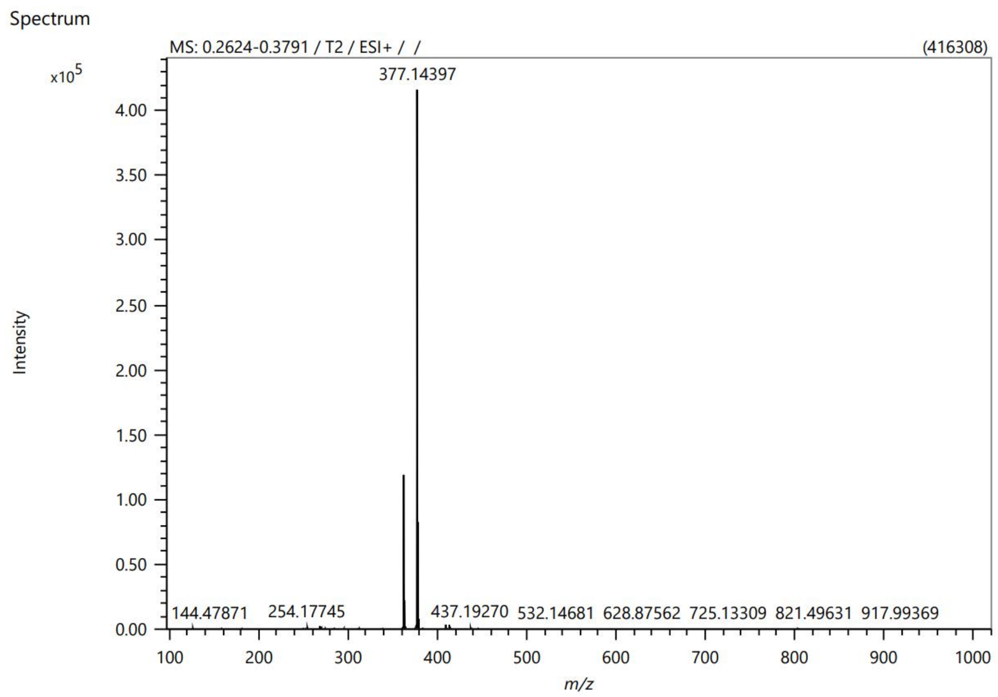

Figure S119. HRMS spectra of compound **TMB2**.
